# Use of Aldehyde–Alkyne–Amine Couplings to Generate Medicinal Chemistry-Relevant Linkers

**DOI:** 10.1021/acsmedchemlett.4c00531

**Published:** 2025-01-25

**Authors:** Andrew McGown, Vesna Vetma, Damien Crepin, Yan Lin, Claire Adcock, Conner Craigon, Jordan Nafie, Daniel von Emloh, Léa Sutton, Kiera Bailey, Lewis Edmunds, Manvendra Sharma, Jonathan D. Wilden, Simon J. Coles, Graham J. Tizzard, William Farnaby, Alessio Ciulli, George E. Kostakis, John Spencer

**Affiliations:** † Sussex Drug Discovery Centre, School of Life Sciences, University of Sussex, Falmer BN1 9QJ, U.K.; ‡ Centre for Targeted Protein Degradation, School of Life Sciences, University of Dundee, 1 James Lindsay Place, Dundee DD1 5JJ, U.K.; § Biotools, Inc., 17546 Beeline Highway, Jupiter, Florida 33458, United States; ∥ Reach Separations, Biocity, Pennyfoot Lane, Nottingham NG1 1GF, U.K.; ⊥ Chemistry Department, School of Life Sciences, University of Sussex, Falmer BN1 9QJ, U.K.; # National Crystallography Service Chemistry, University of Southampton, Southampton SO171BJ, U.K.

**Keywords:** PROTACs, linkers, chiral separation, multicomponent reactions, bromodomains

## Abstract

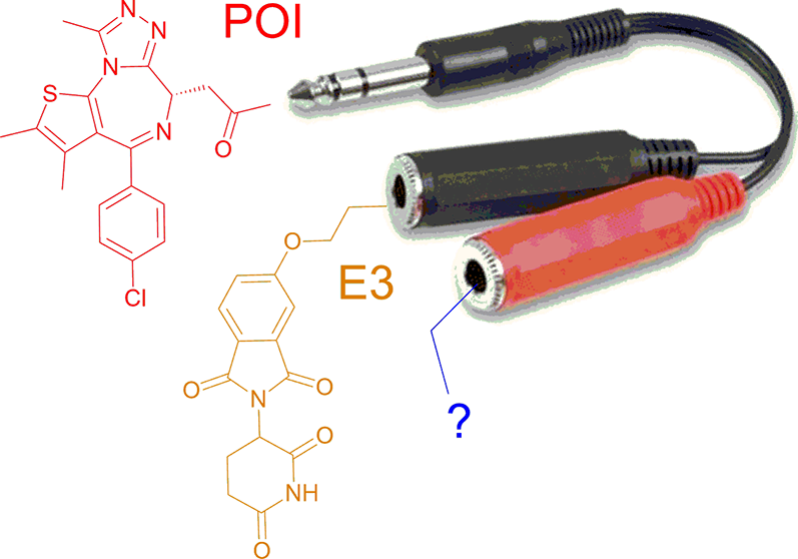

Copper catalyzed aldehyde–alkyne–amine (A^3^) couplings lead to multifunctional, racemic, propargylic amines, many on a multigram scale. As part of an industrial collaboration, a selection of linkers was purified by chiral HPLC to afford single enantiomers, the absolute configuration of which was determined by vibrational circular dichroism (vCD). To show medicinal chemistry applications, selected linkers were further derivatized into potential cellular probes and (+)-JQ1 containing PROTACs (proteolysis targeting chimeras), which degraded their target protein BRD4.

## Introduction

PROTACs (proteolysis targeting chimeras, degraders) are heterobifunctional molecules that comprise a POI (protein of interest) binding ligand and a terminal ligand capable of engaging an E3 ligase separated by a linker group, enabling the formation of a ternary complex for subsequent proteasomal degradation of the POI.^1−5^ Whereas the nature of the POI ligand is dictated by its protein target and the E3 ligase choice is somewhat limited, linker design (*linkerology*) offers an opportunity to influence many suboptimal PROTAC properties, such as target engagement, selectivity, bioavailability, solubility, polar surface area, number of rotatable bonds, and logP, via fine-tuning of, e.g., flexibility, rigidity, chirality, and heteroatom and hydrogen bond donor count, all of which can contribute to degrader success.^6−14^

The aldehyde–alkyne–amine (A^3^) coupling reaction is a powerful transformation due to its atom economical nature and the possibility for assembling molecules with high levels of diversity and complexity, e.g., in library design for medicinal chemistry.^15−17^ For example, we recently described a late-stage A^3^ coupling of the (+)-JQ1^18^ containing alkyne derivative **1a** to afford an A^3^ product.^19^ We now disclose facile gram-scale synthesis of A^3^-derived racemic linkers, chiral separation of selected examples, and uses in PROTAC synthesis to demonstrate synthetic scope and applicability.

## Results and Discussion

**Scheme 1:**
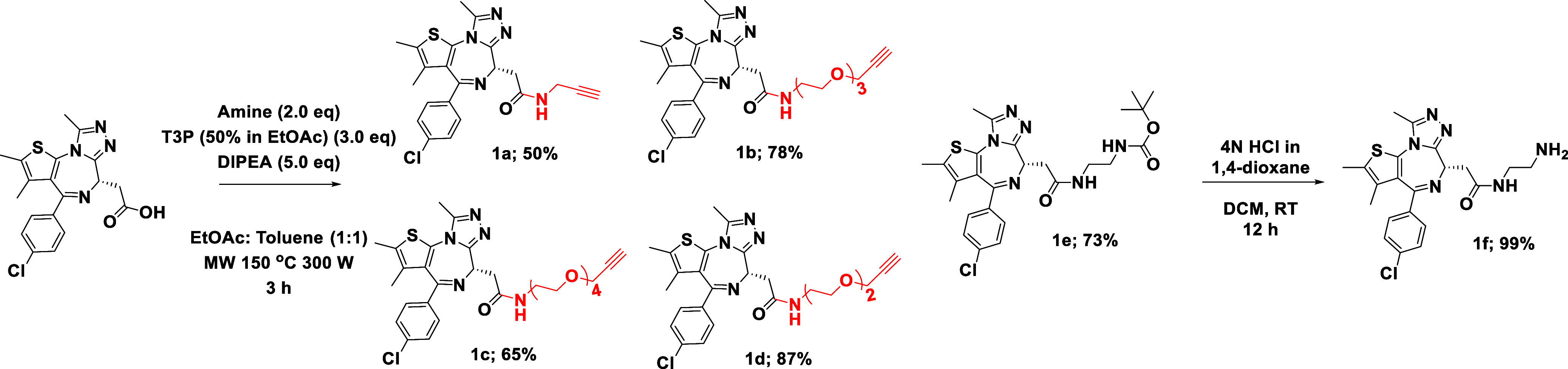
Modified (+)-JQ1 Scaffolds

To broaden synthetic scope, we have expanded the range of (+)-JQ1-containing alkynes (**1a**–**d**), known to have applications in click chemistry and proteomics,^20^ and amines for further functionalization into PROTACs (**1e**, **1f**) (*vide infra*) (Scheme 1).

**Scheme 2:**
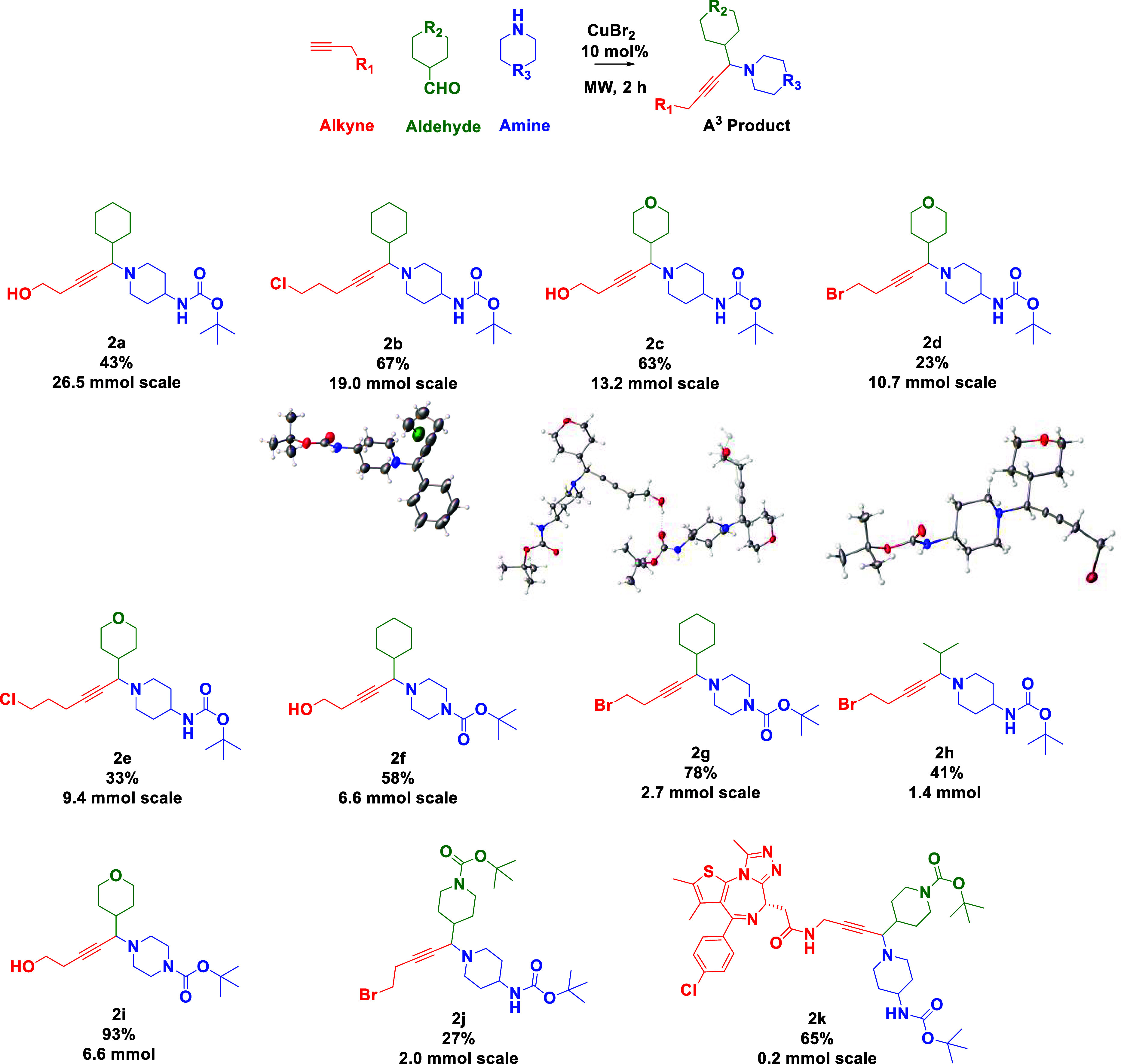
Range of Products from the A^3^ Reaction (**2a**–**2k**)

To enable synthetic flexibility and scope toward -molecules such as PROTACs beyond (+)-JQ1, which is an inhibitor of BRD4, we opted to perform this reaction on substrates that could be coupled to different POI ligands at a later stage. In our hands, the unoptimized A^3^ reaction was successfully performed (Scheme 2) to afford a range of propargylic amines, **2**, which were generally obtained in moderate to good yields, on a relatively high scale in a microwave using simple copper salts.^21,22^ The reaction is tolerant of halo (e.g., **2b**, **2d**) and alcohol (e.g., **2a**, **2c**, **2f**) “handles”, as well as Boc protecting groups (**2a**–**2k**) for further potential functionalization. Moreover, it is tolerant of short or long hydrocarbon chains, hydrophobic, including cyclohexyl and *i*-Pr (e.g., **2a**, **2b**, **2f**, **2h**), or hydrophilic (from the aldehyde precursor, e.g., **2c**, **2d**) side groups, which are important in chimeric drug *linkerology* since these may form interactions with targeted proteins in the context of binary (i.e., between molecule and a single protein) or ternary (i.e., between molecule and two different proteins) interactions. Linker physicochemical properties may also influence and tune overall molecule/drug properties such as solubility, nonspecific binding, and biological stability. A (*+*)-JQ1 analogue, **2k**, again demonstrates that late-stage functionalization is possible on a bioactive core. Product structures were confirmed by a series of NMR spectroscopic experiments, where notable ^13^C NMR peaks were found at ca. δ = 30, 36, and 45 ppm for the piperidine ring, δ = 32, 62, and 67 ppm for the tetrahydropyran group, and δ = 77 and 79 ppm for the alkyne signals. A HSQC (Heteronuclear Single Quantum Coherence) experiment on **2d** located the CH bond at the newly formed stereogenic center to be around δ = 3.4 ppm and at δ = 45 ppm in its respective ^1^H and ^13^C NMR spectra (Figure S4). Moreover, X-ray crystallography established the correct atom connectivity for three of the propargylic amines in the solid state (Scheme 2).^23^

Compounds **2a** and **2b** were analyzed and separable by analytical chiral HPLC, and the racemates were readily separated by preparative chiral HPLC, to >95% ee. Next, the absolute configuration of the separated enantiomers was determined by vibrational circular dichroism (Figures S1 and S2).^24^ For example, a 2.1 g sample of *rac*-**2a** yielded (*R*)-**2a** (710 mg) and (*S*)-**2a** (574 mg), both in >98% ee, demonstrating that this is also amenable to providing enantiopure compounds to scale and to demand. Practically, in our hands, having a method to afford gram quantities of racemate was a more attractive proposition than the stereoselective synthesis of one enantiomer (Figure S2).

**Scheme 3:**
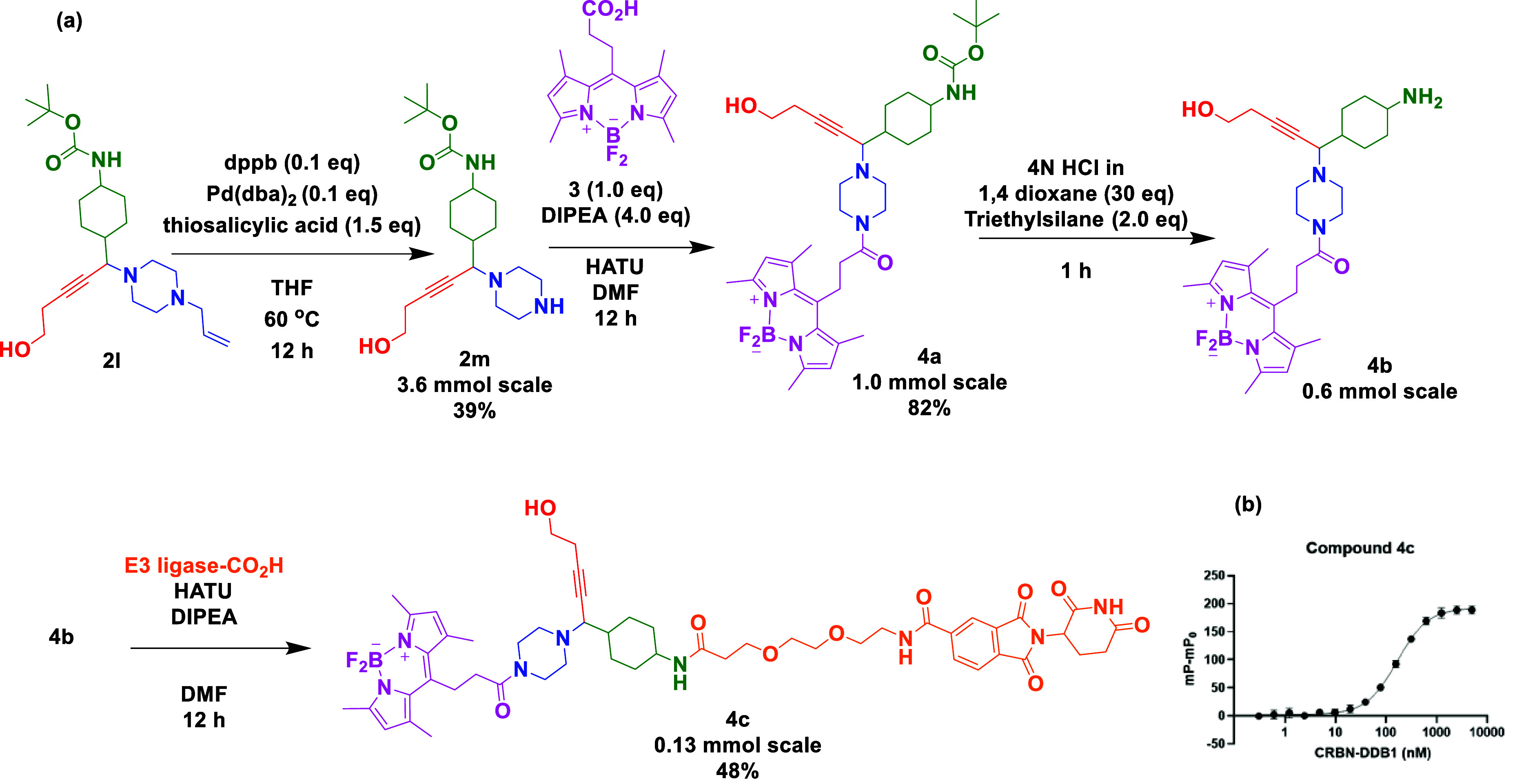
(a) An A^3^ Product **4c** Decorated with Representative Bodipy, E3 ligase, and a Free Handle for Late-Stage Incorporation of a POI Ligand; (b) Fluorescent Polarization CRBN-Binding Assay for **4c**

Medicinal chemistry scope was expanded by the synthesis of a few representative linkers, which were elaborated to modalities adorned with a cellular marker, an E3 ligase motif, and a free alcohol function. Hence, the allyl, Boc-protected A^3^ product **2l** was synthesized on a multigram scale in 92% yield and selected as an orthogonally protected linker with three potential handles for functionalization. Initially, treatment with Pd(dba)_2_, dppb, and thiosalicylic acid removed the allyl protecting group to give **2m** in 39% yield. The resulting secondary amine was coupled to a bodipy-containing carboxylic acid **3**(25) to afford the Boc-protected linker **4a** (82% yield, Scheme 3). Simple Boc removal with acid exposed the secondary amine **4b** intermediate, which was coupled to an acid comprising E3 ligand affording **4c**. Such modalities have a free “handle” that could be added to a POI-binding ligand of choice, e.g., by substitution chemistry, esterification, or ether formation. Compound **4c** was selected as an exemplar with many permutations possible in terms of alkyne, amine, aldehyde substituents, cell probe motif, E3 ligand, and linker size and type, not to mention chirality (racemic, or (*R*)- or (*S*)-linker). Fluorescence polarization (FP) was performed to measure a dissociation constant (*K*_d_) of 165.7 ± 3.5 nM for the direct binding of compound **4c** to CRBN-DDB1 (cereblon DNA damage-binding protein 1 complex) (Scheme 3b).^26^

**Scheme 4:**
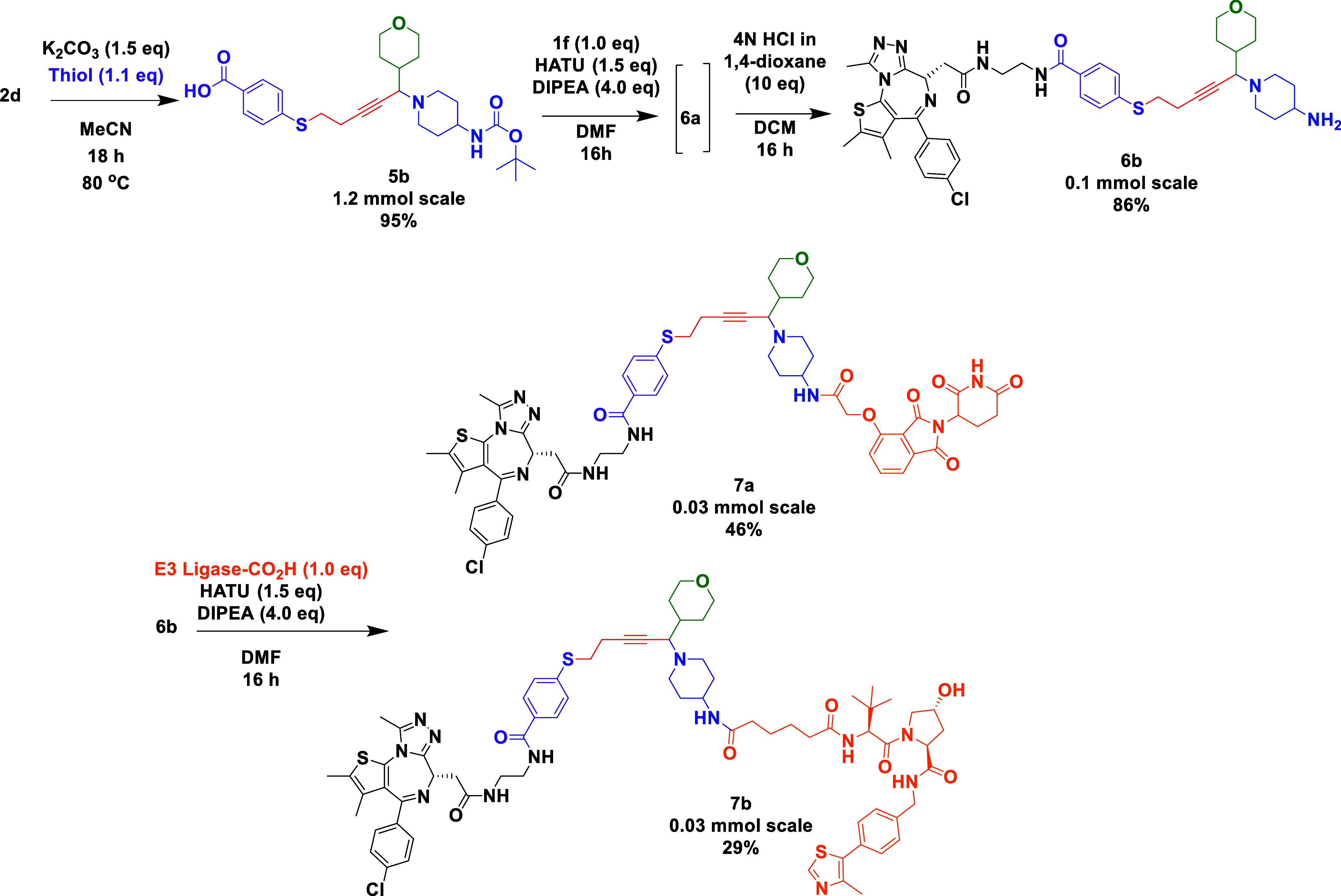
A^3^ Linked with a POI and E3 Ligase

Exploitation of the A^3^ chemistry toward PROTACs was also explored. We selected (+)-JQ1 as a POI ligand of choice to benchmark activity versus that of other PROTACs. Two final PROTAC candidates, **7a** and **7b**, were synthesized using a thiobenzoic acid linker attached to propargylic amine **2d** (Scheme 4).

**Scheme 5:**
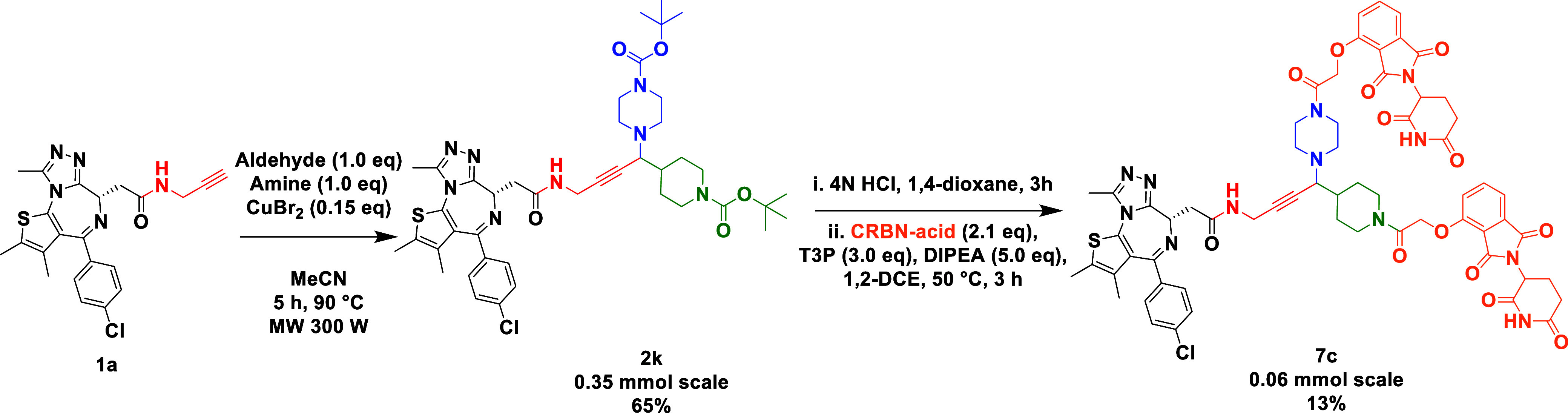
“Plug and Play” A^3^ Reaction Rapidly Leading to an Elaborated Trivalent PROTAC

An effective A^3^ coupling “plug and play” reaction^27−30^ afforded the double Boc-protected propargylic amine **2k** (Scheme 5). Given that both amine components have identical protecting groups, simple deprotection led to two similar secondary amines that were coupled with a CRBN E3 ligase ligand to afford a trivalent PROTAC containing two copies of an E3 ligase moiety.

The A^3^ generated PROTACs **7a**–**c** were examined for their BRD4 degradation capabilities using the Promega Nano-Glo HiBiT assay^31,32^ against known bromodomain degrader PROTACs MZ-1, dBET6, SIM1, SIM6, and AGB1.^33−36^ It was observed that, while compounds **7b** and **7c** were poor examples with DC_50_ values >1 μM, being attributed to their poor solubility and structural limitations, compound **7a** was identified as a potent BRD4 degrader with a DC_50_ value of 89.4 nM (vs *ca*. 20 nM for MZ-1), 18 h after dosage.

**Figure 1:**
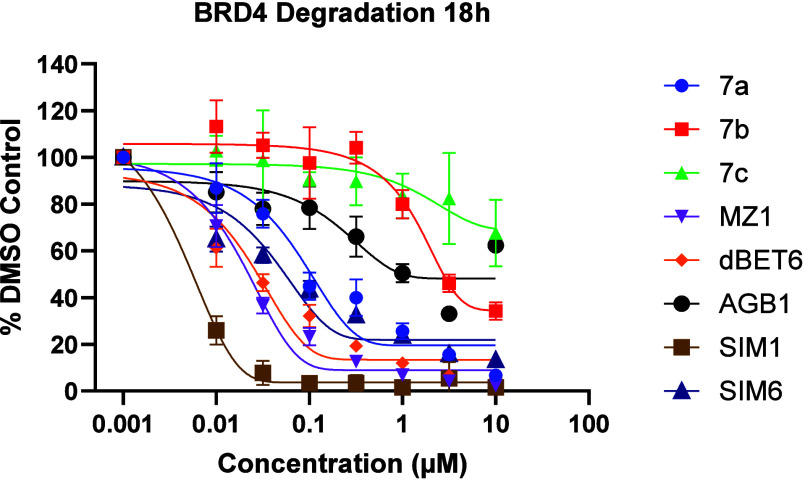
BRD4 degradation assay at 18 h in HEK293 CRISPR HiBiT BRD4 cell line compared with related (+)-JQ1-based PROTACs. Data plotted are average ± SD of *n* = 3 biological replicates.

The CRBN containing compounds **7a** and **7c** were tested for selected *in vitro* PK properties (**7b** was visibly poorly soluble and was not selected) (Figure 1). Both displayed low permeability and low solubility, with the latter demonstrating greater microsomal stability (**7c**: HLM, *t*_1/2_ 17.53 min; Cl_int_ at 79.07 mL/min/mg) compared with the high clearance of **7a** (*t*_1/2_ 6.64 min with Cl_int_ at 208.87 L/min/mg) (Figure S3). Moreover, the solubility of both final compounds was low (5% PBS buffer, saline). These examples were merely chosen to showcase the synthetic potential of the chemistry rather than a medicinal chemistry-focused PROTAC optimization approach for which scope remains to optimize PK properties in future heterofunctional molecules. For example, the alkyne functionality, present in the A^3^ products **2a** and **2c**, although present in a number of marketed, bioactive molecules,^37^ even PROTACs,^38^ acting as a rigid hydrophobic spacer, can be reduced by catalytic hydrogenation^39^ to afford a saturated, more flexible yet possibly less metabolically labile linker.

**Figure 2:**
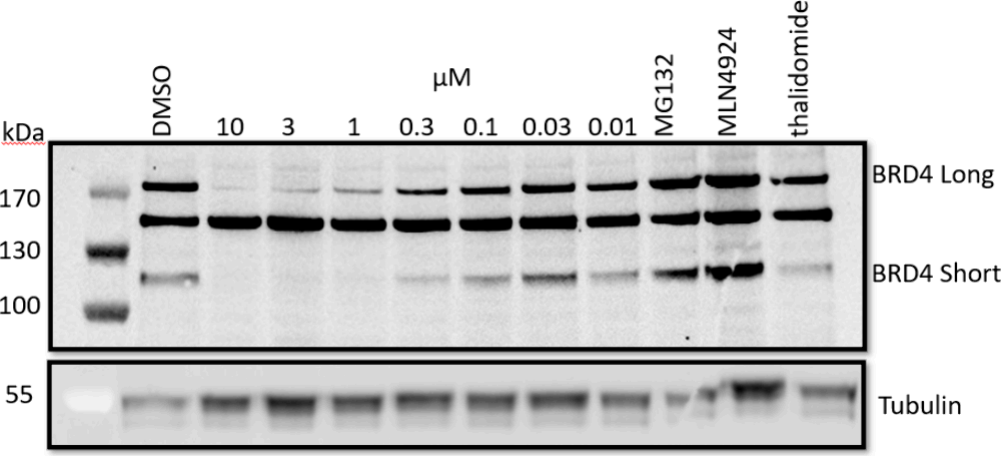
Representative Western blot of **7a** degradation of BRD4 performed in HEK293 cell line after 18 h treatment. The experiment was done as *n* = 3 independent biological replicates.

The final three potential PROTACs were tested against BRD4 in degradation assays using a HEK293 CRISPR HiBiT BRD4 cell line and HEK293 parental cell line (Figures 1 and 2).^40^

The best analogue, **7a**, was next shown, by Western blot, to degrade both BRD4 long and short in the 300–1000 nM range, reversed by the addition of either proteasome or neddylation inhibitors or by thalidomide.

It is encouraging that despite poor permeability we still observe degradation. The presence of basic centers and the potential to look at salts may help tune aqueous solubility and with rapid, but no doubt tunable, clearance, depending on the half-life of the POI, a quickly acting degrader might be desirable, in certain circumstances, to minimize off target toxicity.

In summary, we have applied the A^3^ coupling reaction to the gram scale synthesis of racemic linkers, which can be readily separated into single enantiomers or used in the design of PROTACs, one of which displays a DC_50_ < 100 nM vs BRD4. Additionally, due to the complementarity of using S-nucleophiles with such linkers, they might find applications in, e.g., antibody–drug conjugate linker chemistry.^41^ Of particular interest was a “plug and play” three-component A^3^ reaction leading to a POI-double E3 ligase targeting product, which should be amenable to a myriad of homo- and hetero-POI–E3 permutations^35,42,43^ and to automated array chemistry.^44^

### Safety Statement

All reactions were performed using the appropriate PPE, following rigorous health and safety protocols. Compounds were considered toxic and handled appropriately, such as weighing in vented hoods and correct disposal via approved contractors. Procedures were recorded and countersigned in electronic laboratory notebooks. Unless otherwise stated, reactions were either heated using a Radleys hot plate or via a CEM or Biotage microwave (high pressure and temperature) within a ventilated fume hood, with the sash lowered. No safety violations or accident or near-miss incidents were reported during this study.

## Supporting Information Available

Synthetic data for **1a**–**7c**, ^1^H and ^13^C NMR, HPLC purity, and HRMS; scanned spectra; purification and vCD for (***R***)-**2b**; in vitro PK for **7a** and **7c** (PDF)

## Abbreviations

CRBN: cereblon
dba: dibenzylideneacetone
dppb: 1,4-bis(diphenylphosphino)butane
E3 ligase: ubiquitin ligase
POI: protein of interest
PROTAC: Proteolysis-targeting chimera
vCD: vibrational circular dichroism
VHL: Von Hippel–Lindau
