# Supplementary material for: Use of Aldehyde–Alkyne–Amine Couplings to Generate Medicinal Chemistry-Relevant Linkers
Source: ACS Med Chem Lett. 2025 Jan 25;16(2):278–84. doi: 10.1021/acsmedchemlett.4c00531 (PMC11831382; doi:10.1021/acsmedchemlett.4c00531)
Supplement: Supplementary file 1 — ml4c00531_si_001.pdf [file ml4c00531_si_001.pdf]

Use of Aldehyde-Alkyne-Amine Couplings to Generate Medicinal Chemistry-Relevant Linkers

Andrew McGown,[#,*] Vesna Vetma,[~] Damien Crepin,[#] Yan Lin,[#] Claire Adcock,[#] Conner Craigon,[~] Jordan Nafie,[$] Daniel von Emloh,[&] Léa Sutton,[&] Kiera Bailey,[&] Lewis Edmunds,[&] Manvendra Sharma,[/] Jonathan D. Wilden,[/] Simon J. Coles,[≠] Graham J. Tizzard,[≠] William Farnaby,[~] Alessio Ciulli,[~] George E. Kostakis,[/] John Spencer[#,/,*].

[#]Sussex Drug Discovery Centre, School of Life Sciences, University of Sussex, Falmer, BN1 9QJ, UK. Corresponding author Email: a.mcgown@sussex.ac.uk. ORCID: 0000-0002-2187-3850. Email: j.spencer@sussex.ac.uk. ORCID: 0000-0001-5231-8836. [~]Centre for Targeted Protein Degradation, 1 James Lindsay Place, School of Life Sciences, University of Dundee, Dundee, DD1 5JJ, UK. [$]Biotools, Inc., 17546 Beeline Highway, Jupiter, Florida 33458, US. [&]Reach Separations, Biocity, Pennyfoot Lane, Nottingham, NG1 1GF, UK. [/] Chemistry Department, School of Life Sciences, University of Sussex, Falmer, BN1 9QJ, UK.[≠]National Crystallography Service Chemistry, University of Southampton, Southampton, SO171BJ, UK.

Table of Contents

Figure S1. Chiral separation/ vCD for (*R*)-**2a**.. S2

Figure S2. Chromatogram for *rac*- and ***(R)*-2b** and the latter's VCD..S3

Figure S3. PK data for **7a** and **7c**..S4

Figure S4. HSQC spectrum for **2d**...S5

Biological assays...S6

Synthetic Procedures, spectra and scanned spectra..S7

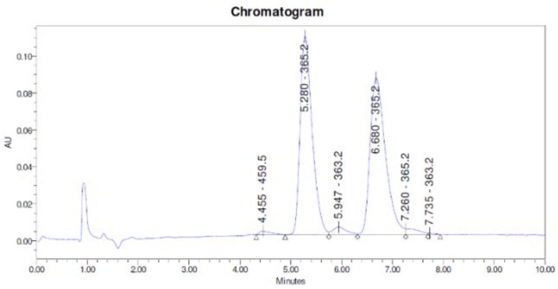

(a)

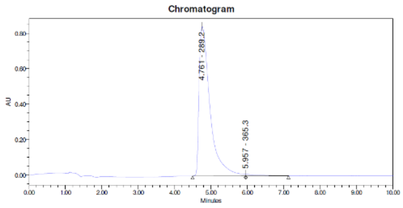

Peak Results

	Retention Time (min)	Area (µV*sec)	% Area	Width @ 50%
1	4.76	17659906	99.0	0.29523
2	5.96	178036	1.0	

(b)

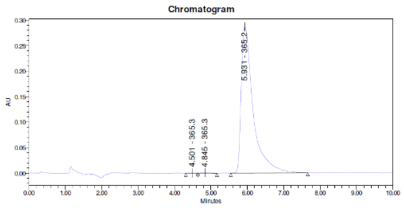

Peak Results

	Retention Time (min)	Area (µV*sec)	% Area	Width @ 50%
1	4.50	3626	0.1	
2	4.85	17000	0.3	0.23485
3	5.93	6577365	99.7	0.30850

(c)

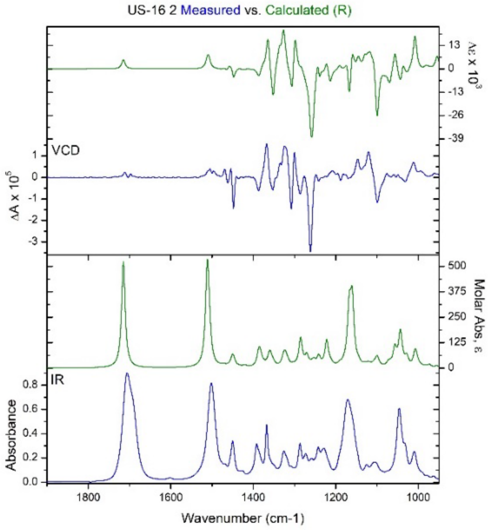

(d)

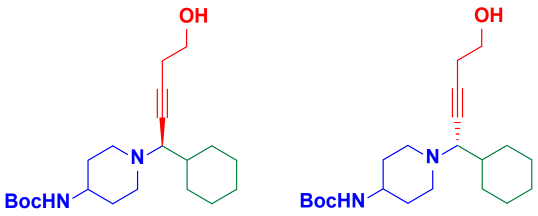

(e)

Figure S1. (a) Racemic, (b) separated (R)-, (c) separated (S)- **2a**. (d) Measured *vs* calculated vCD of (*R*)-**2a**. (e) (*R*)- and (*S*)- **2a**.

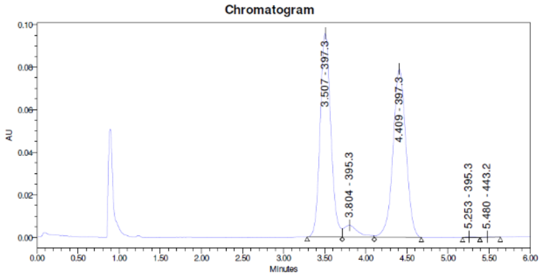

Peak Results

	Retention Time (min)	Area (µV*sec)	% Area	Width @ 50%
1	3.51	860018	47.8	0.13801
2	3.80	66149	3.7	
3	4.41	866683	48.2	0.16684
4	5.25	3165	0.2	0.11872
5	5.48	2057	0.1	0.10343

(a)

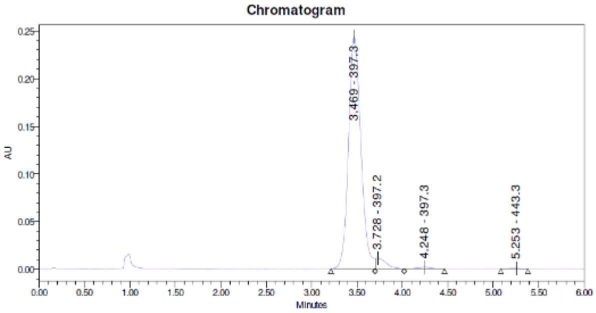

Peak Results

	Retention Time (min)	Area (µV*sec)	% Area	Width @ 50%
1	3.47	2308328	95.6	0.14436
2	3.73	87994	3.6	
3	4.25	14061	0.6	0.16248
[illegible]	[illegible]	[illegible]	[illegible]	[illegible]

(b)

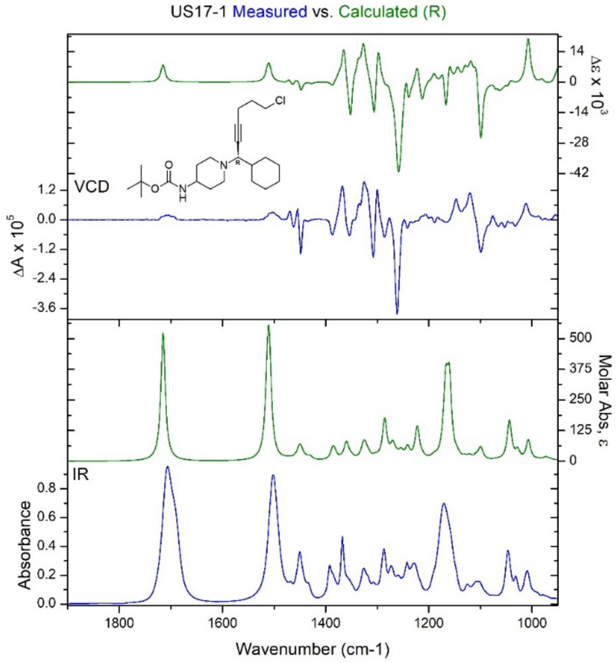

(c)

Figure S2. Chromatogram for (a) *rac-* and (b) ***(R)*-2b** and (c) VCD for the (*R*)-analogue.

Compound ID	Papp cm/sec, x10-6)		A-B Permeability	Efflux Ratio B-A/A-B	P-gp Substrate
	A-B	B-A			
UOS-00060104-002 (**7a**)	0.007	0.010	Low	1.4	Not-Substrate
AMPRO-233 (**7c**)	<0.1	<0.1	Low	-	-
Labetolol	2.6	18.2	Medium	7.0	Substrate

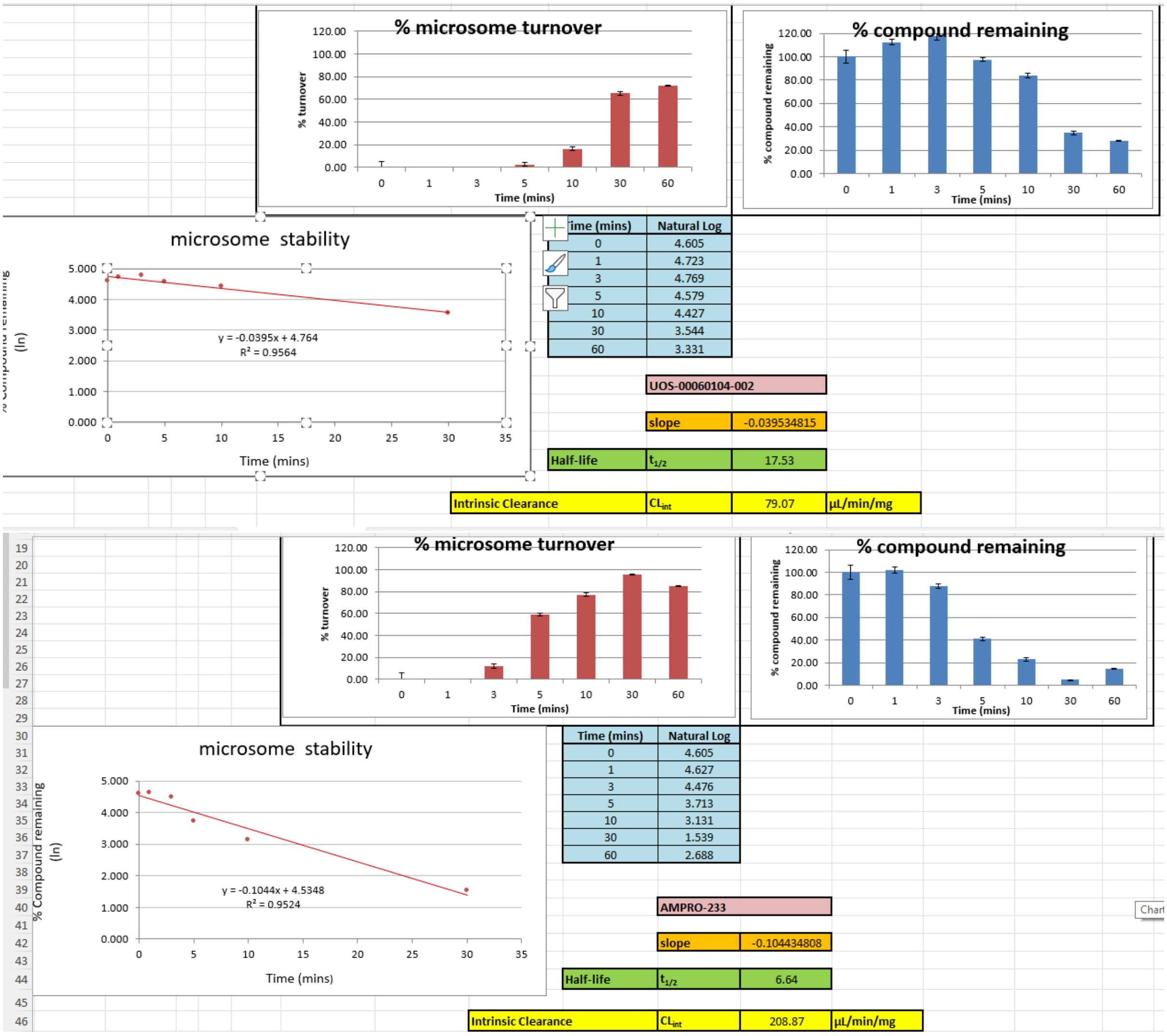

Figure S3. PK data for **7a** and **7c**.

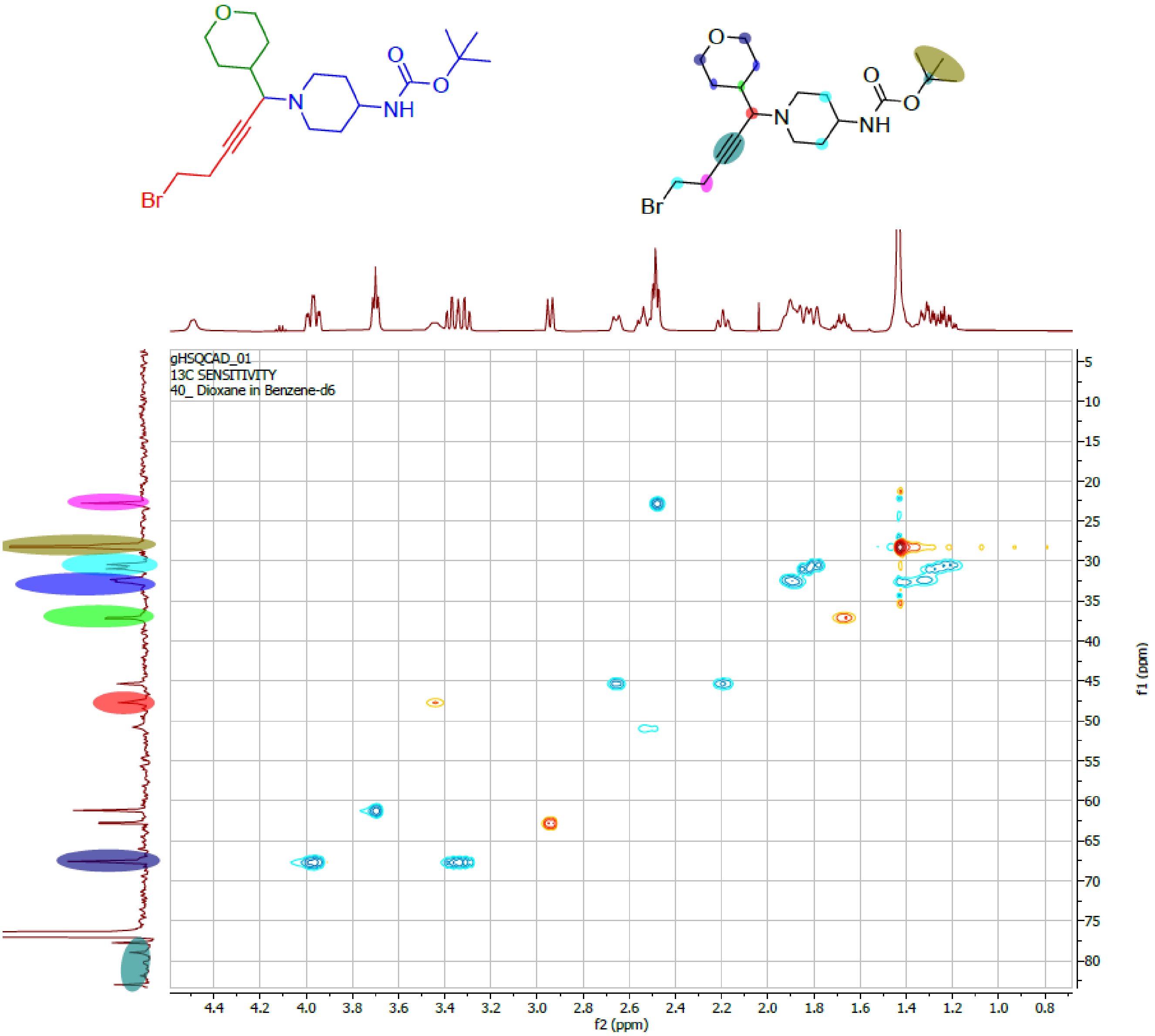

Figure S4. HSQC for **2d**.

Biological Assays.

Methods

Cell culture. HEK293 CRISPR HIBIT BRD4 cells were generated at CeTPD using CRISPR-CAS technology. Parental HEK293 and HEK293 CRISPR HIBIT BRD4 cell lines were cultured in DMEM media (Gibco), supplemented with 10% fetal bovine serum (FBS), and were kept at 37 °C, 5% CO_2 in a humidified incubator.

BRD4 Degradation Assays used a HiBiT Lytic assay.

HEK293 CRISPR HIBIT BRD4 cells were seeded at 5 X 10^5 cells per well in culture medium into white bottom opaque 96-well plates (Perkin Elmer cat no. 5680). Plates were incubated at 37 °C, 5% CO_2 in a humidified incubator overnight. Compounds (10 mM stock in DMSO) were diluted to a half-log scale and added to the cells. Plates were further incubated at 37 °C for 18 hours. Following incubation, 100 µL per well Promega Nano-Glo HiBiT lytic detection reagent mix (Promega Nano-Glo HiBiT Lytic Detection System #N3050), prepared according to the manufacturer´s instructions in the kit, were added. The plates were incubated on an orbital shaker for 10 min. Luminescence was measured using plate reader (BMG Labtech PHERAstar). Luminescence levels in the treatment wells were normalised to DMSO-treated samples and plotted as percentage of DMSO control in GraphPad Prism (10.2.3).Western blot

5x10^5 HEK293 CRISPR HIBIT BRD4 cells were seeded into 6-well plates 24 hours before treatment. Next day, wells were treated with range titration of compound **7a** or a combination treatment of 10 µM compound **7a** and 10 µM MG132 (Selleckchem #S2619), MLN4924 (Tocris #6499) and thalidomide (Flourochem #50-35-1).

Cells were treated for 18 hours as indicated, washed with PBS and lysed with lysis buffer (1% Triton X-100, 150 mM NaCl, 1 mM EDTA, 50 mM Tris pH 7.4, protease inhibitor cocktail (Roche), 50 units/mL benzonase nuclease (Sigma). Protein concentration was determined by BCA assay (Pierce) and the absorbance at 562 nm measured by spectrophotometry on a plate reader (BMG Labtech PHERAstar). Samples were separated by SDS-PAGE using 20 µg of protein per well of NuPAGE Novex 4-12% BIS-TRIS gels (Invitrogen) and transferred to 0.2 µm pore nitrocellulose membrane (Amersham) by wet transfer. Western blot images were obtained through detection of anti-rabbit BRD4 (1:1000, Abcam #128874), hFAB™ Rhodamine anti-tubulin (1:10000, Biorad #12004166) antibodies with donkey anti-rabbit IRDye 800CW secondary antibody (1:10000, LI-COR #926-32213) using a ChemiDoc MP imaging system (Bio-Rad). Western blots were quantified using Image Studio Lite (Licor, version 5.2) with normalisation to loading control and DMSO and further analysed using GraphPad Prism (version 10.2.3) and ImageJ (Fiji).

Fluorescence Polarization (FP). The experiment was performed at room temperature on a PHERAstar FS (BMG LABTECH), with fluorescence excitation wavelength at 485 nm and emission at 520 nm. FP binding assay was performed in triplicates in low volume 384-well plates (Corning 3820), with a total well volume of 20 uL. The assay buffer was 50 mM HEPES pH 7.4, 100 mM NaCl, 1 mM TCEP, 0.1 % (w/v) Tween20 and the final concentration of DMSO in all assay wells was 2% (v/v).

A direct titration of CRBN-DDB1 against the fluorescent compound **4c** was performed using 1 nM of compound **4c** and decreasing concentrations of CRBN DDB1 (15-point two-fold serial dilution from 5 µM to 0.3 nM, triplicate wells). Obtained data was fitted to 4PL non-logistic regression model in GraphPad Prism (version 10.2.3) and the dissociation constant (Kd) was derived.

Experimental Section. All reactions were carried out under air at room temperature unless otherwise stated, using commercial grade reagents and solvents. E3 ligase precursors were purchased from commercial vendors: thalidomide-5-(PEG2-acid; https://broadpharm.com/product/bp-28033), (S, R, S)-AHPC-pentanoic-acid (https://broadpharm.com/product/bp-24508) via their UK distributor, Tebubio. 2-((2-(2,6-Dioxopiperidin-3-yl)-1,3-dioxoisoindolin-4-yl)oxy)acetic acid, was acquired from BLD (https://www.bldpharm.com/products/1061605-21-7.html). The progress of all reactions was monitored by LC-MS (5 μm C18 110 Å column) and TLC using commercially available silica gel plates (60 Å, F254), with visualisation under UV or by $KMnO_4$ staining. All NMR samples were ran using a Varian NMR 600, 500 or 400 MHz spectrometer, stated per sample. Chemical shifts are reported in parts per million (ppm), with δ relative to the residual solvent peak of the solution for ^{1}H and ^{13}C. spectra Purifications were carried out using a Teledyne ISCO purification unit, either Combi Flash RF 75 PSI or Combi Flash RF 150 PSI, using either Teledyne or Biotage silica gel columns. LC-MS were performed on a Shimadzu 2020 Mass Directed Automated Purification (MDAP) system using a 30-minute method in water/acetonitrile with 0.1% formic acid (5 min at 5%, 5-95% over 20 min, 5 min at 95% or 5 min at 30%, 30-95% over 20 min, 5 min at 95%) with the UV set to 254 nm. All mass spectrometry was conducted by Dr. Ramon Gonzalez-Mendez at the University of Sussex. Calculations of compound m/z were performed using Chemdraw Ultra 12.0.2.1076.

(+)-JQ1-OH.

Data in concordance with the literature[1].

(S)-2-(4-(4-chlorophenyl)-2,3,9-trimethyl-6H-thieno[3,2-f][1,2,4]triazolo[4,3-a][1,4]diazepin-6-yl)acetic acid.

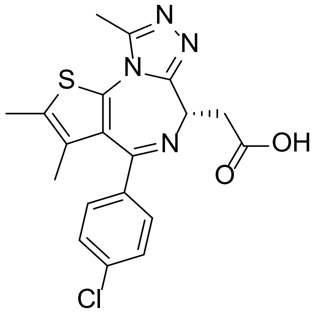

(+)-JQ1 (2000mg, 4.38mmol, 1.0eq) was dissolved in DCM (10mL) with stirring. To this was added TFA (3351μL, 43.8mmol, 10.0eq) dropwise over 5 minutes. The reaction mixture was stirred at room temperature overnight and, upon completion, the DCM was removed, by evaporation under reduced pressure, leading to a deep yellow oil. This oil was suspended in water (30mL) and triturated by sonication over 20 minutes to afford pure product as a yellow solid – this process may be repeated with the water being carefully decanted and replaced until all material is free flowing. The title compound (1750 mg, 4.28 mmol, 98%) was isolated in quantitative yield. ^{1}H NMR (600 MHz, DMSO-d_6) δ 12.45 (s, 1H), 7.48 (d, J = 8.4 Hz, 2H), 7.42 (d, J = 8.2 Hz, 2H), 4.42 (t, J = 7.1 Hz, 1H), 3.41, (dd, J = 16.8, 7.0 Hz, 1H), 3.30 (dd, J = 16.7, 7.5 Hz, 1H), 2.58 (s, 3H), 2.39 (s, 3H), 1.61 (s, 3H). LCMS RT = 5.643 min, %A = 98%, m/z [M+H] = 401.05, m/z [M-H] = 399.00.

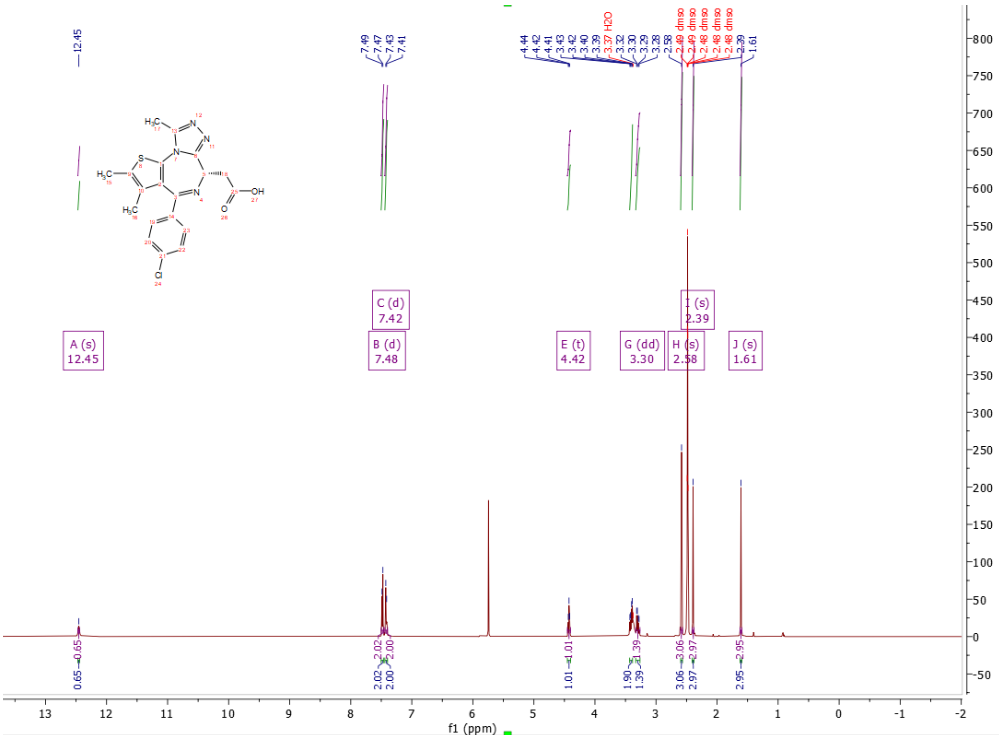

Figure S1 – ^{1}H Spectra for **(+)-JQ1-OH**.

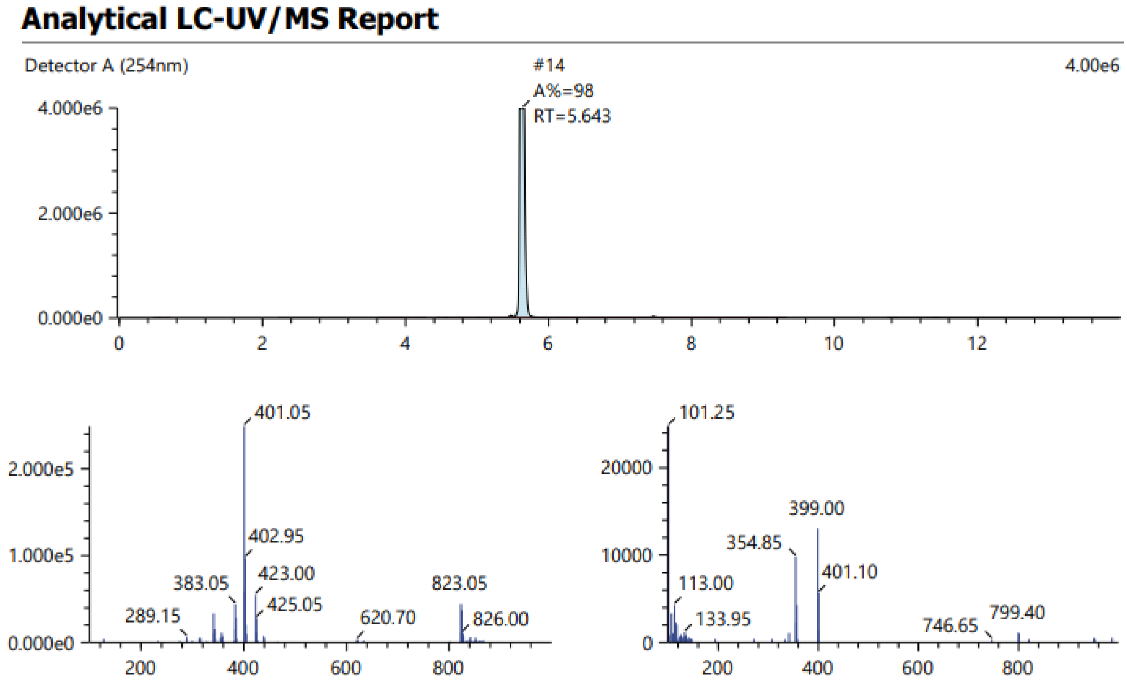

Figure S2 – LCMS report for **(+)-JQ1-OH**.

General Synthesis for (+)-JQ1-OH amide coupling (unless stated otherwise).

In a microwave vial, **(+)-JQ1-OH** (250 mg, 0.62 mmol, 1.0 eq) and amine (1.24 mmol, 2.0 eq) were dissolved in a mixture of ethyl acetate and toluene (1:1, 3.0 mL). The vessel was capped and flushed with nitrogen before anhydrous N,N-diisopropylethylamine (3.10 mmol, 5.0 eq) and propylphosphonic anhydride solution (50% v/v in ethyl acetate, 1.86 mmol, 3.0 eq) were added and the mixture was heated at 150 °C for 3 hours in a microwave reactor (300 W).

Upon completion, the reaction mixture was cooled to room temperature and diluted with EtOAc (5 mL). The organics were washed with aq. $NaHCO_3$ (3 x 10 mL), saturated brine (10 mL) and dried over $MgSO_4$ before being concentrated to a viscous gum. The latter was purified by column chromatography (SiO_2, 12g, DCM: MeOH) with the product eluting between 15-18% MeOH.

1a AMKV-107/AMPRO-325

(S)-2-(4-(4-chlorophenyl)-2,3,9-trimethyl-6H-thieno[3,2-f][1,2,4]triazolo[4,3-a][1,4]diazepin-6-yl)-N-(prop-2-yn-1-yl)acetamide.

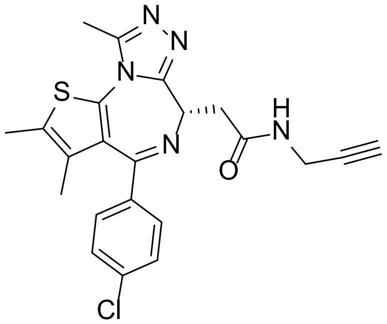

Chemical Formula: $C_{22}H_{20}ClN_5OS$
Exact Mass: 437.1077
Molecular Weight: 437.9451

Using the general method **1a** was isolated as a yellow solid (172.5mg, 0.37mmol, 50% yield). ^{1}H NMR (600 MHz, Chloroform-d) δ 7.40 (d, 8.0 Hz, 2H), 7.31 (d, 8.0 Hz, 2H), 7.28 (t, 5.5 Hz, 1H), 4.62 (t, 7.0 Hz, 1H), 4.18 (m, 1H), 3,98 (m, 1H), 3.53 (dd, 14.5, 7.0 Hz, 1H), 3.42 (dd, 14.5, 7.0 Hz, 1H), 2.66 (s, 3H), 2.39 (s, 3H), 2.22 (t, 2.6 Hz, 1H), 1.66 (s, 3H). ^{13}C NMR (151 MHz, Chloroform-d) δ 170.4, 164.1, 155.5, 149.9, 136.8, 136.5, 132.1, 131.0, 130.9, 130.5, 129.9 (2C), 128.7 (2C), 79.7, 71.3, 54.3, 38.9, 29.2, 14.4, 13.1, 11.8. LCMS RT 5.901 min, A% = 92%*, Mw [M+H] = 438.00.*Single peak LCMS with no other substantial peaks. HRMS - $C_{22}H_{20}ClN_5OS$ – Calculated [M+H] = 438.1155. Experimental [M+H] = 438.1150 (ppm = -1.1).

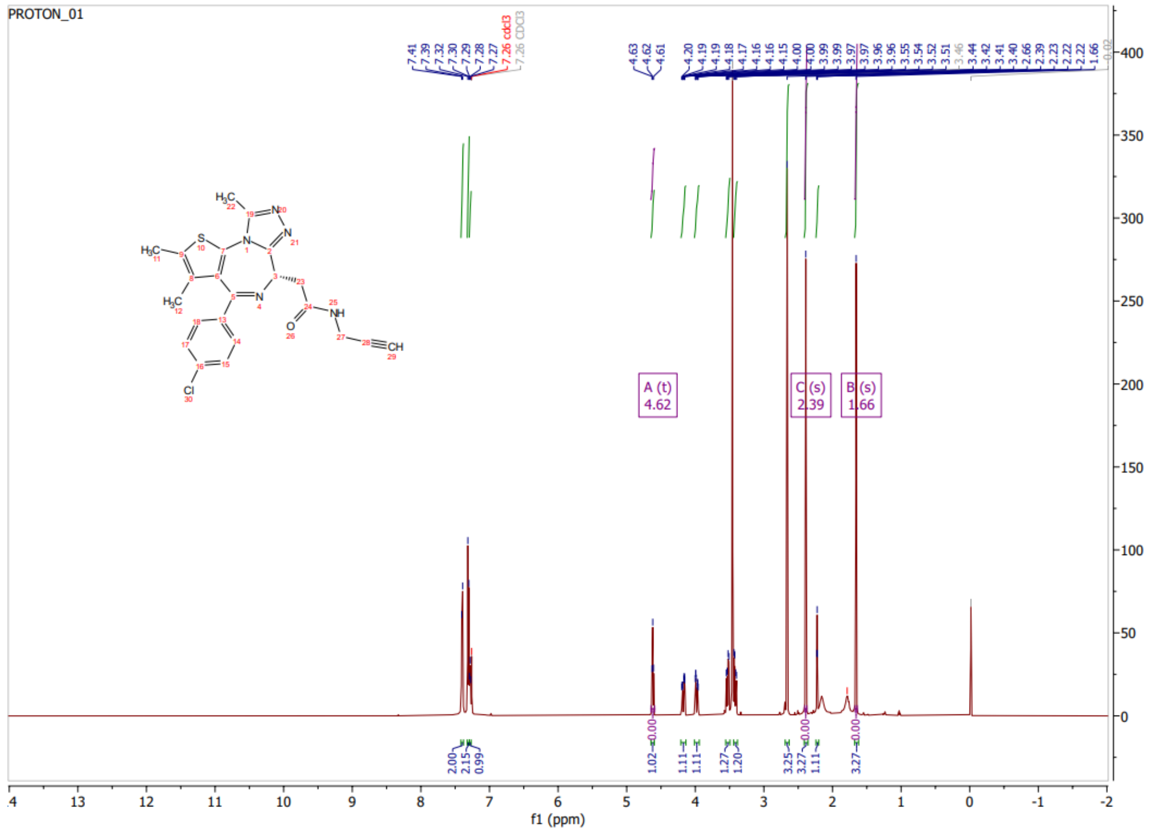

Figure S3 – ¹H Spectra for compound **1a**.

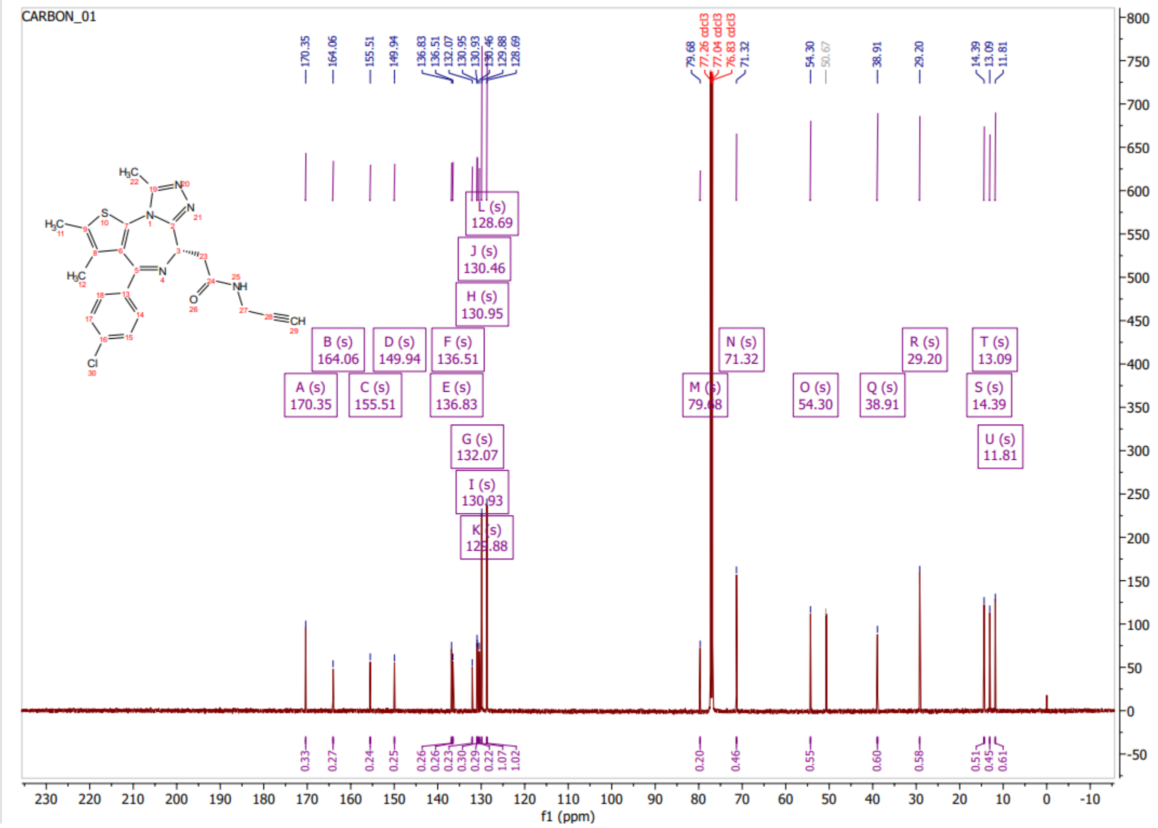

Figure S4 – ^{13}C Spectra for compound **1a**.

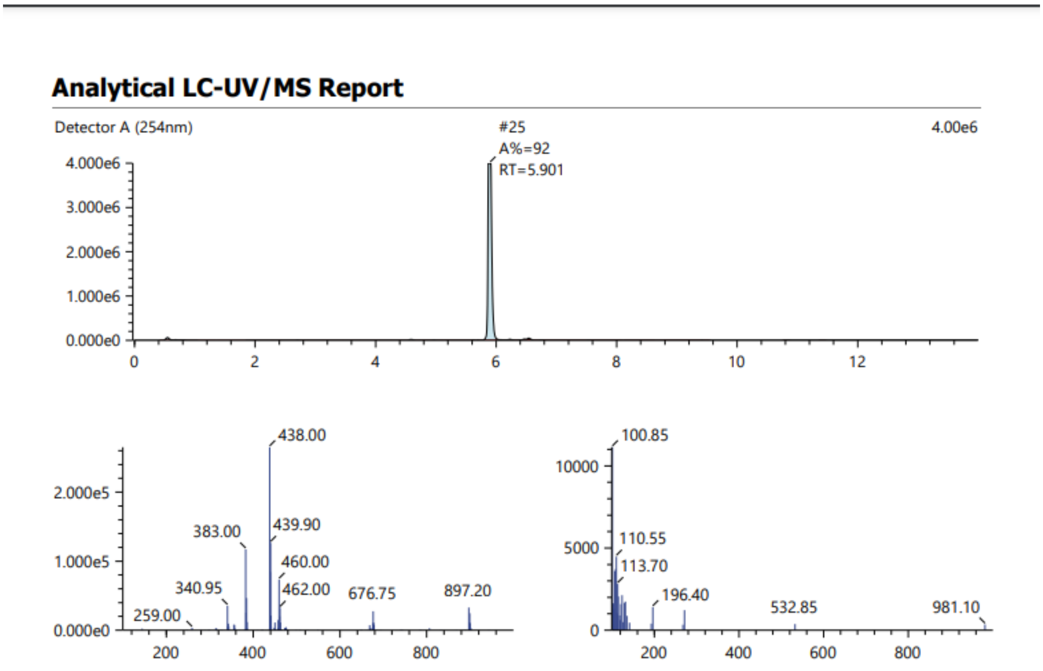

Figure S5 – LCMS report for compound **1a**.

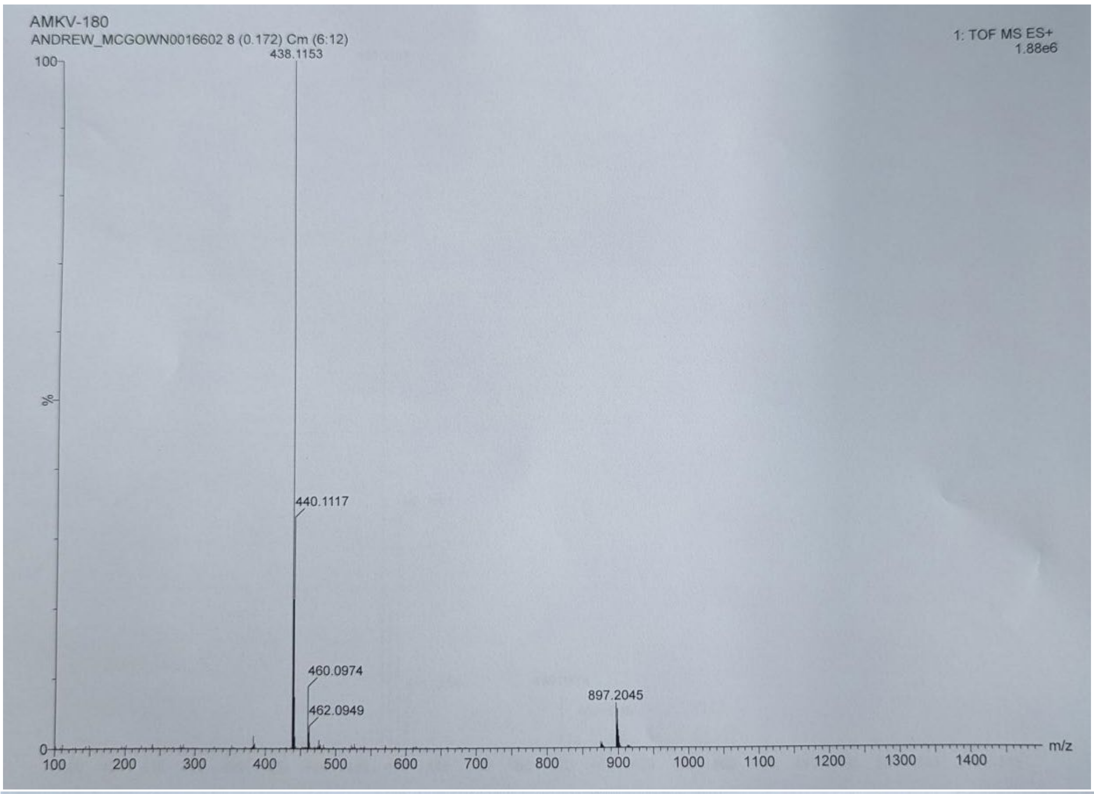

Elemental Composition Report Page 1

Single Mass Analysis
Tolerance = 50.0 PPM / DBE: min = -50.0, max = 75.0
Element prediction: Off
Number of isotope peaks used for i-FIT = 3

Monoisotopic Mass, Even Electron Ions
1 formula(e) evaluated with 1 results within limits (up to 50 best isotopic matches for each mass)
Elements Used:
C: 22-22 H: 0-1000 N: 5-5 O: 1-1 S: 1-1 Cl: 1-1
AMKV-180
ANDREW_MCGOWN0016602 8 (0.172) AM2 (Ar,20000.0,0.00,0.00); Cm (6:12) 1: TOF MS ES+
6.66e+006

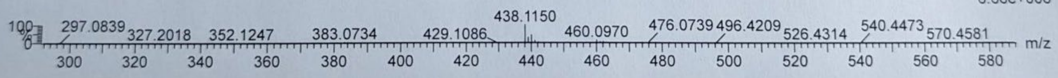

Minimum:				-50.0				
Maximum:		5.0	50.0	75.0				
Mass	Calc. Mass	mDa	PPM	DBE	i-FIT	Norm	Conf(%)	Formula
438.1150	438.1155	-0.5	-1.1	14.5	490.1	n/a	n/a	C22 H21 N5 O S Cl

Figure S6 – HRMS analysis for compound **1a**.

1b AMKV-048/AMPRO-032

(S)-2-(4-(4-chlorophenyl)-2,3,9-trimethyl-6H-thieno[3,2-f][1,2,4]triazolo[4,3-a][1,4]diazepin-6-yl)-N-(2-(2-(2-(prop-2-yn-1-yloxy)ethoxy)ethoxy)ethyl)acetamide.

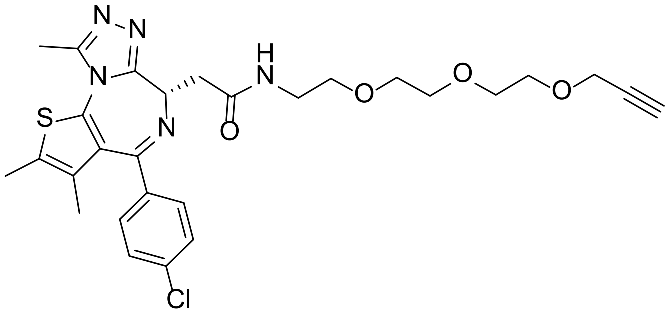

Chemical Formula: $C_{28}H_{32}ClN_5O_4S$
Exact Mass: 569.1864
Molecular Weight: 570.1028

Using the general method **1b** was obtained as a yellow, viscous oil (275.7mg, 0.48mmol, 78%).

^{1}H NMR (600 MHz, Chloroform-d) δ 7.38 (d, J = 8.0 Hz, 2H), 7.30 (d, J = 8.0 Hz, 2H), 6.91 (t, J = 5.5 Hz, 1H), 4.63 (t, J = 7.0 Hz, 1H), 4.19 (t, J = 2.0 Hz, 2H), 3.73 – 3.66 (m, 4H), 3.65 (s, 3H), 3.58 (m, 2H), 3.49 (m, 3H), 3.37 (dd, J = 14.5, 7.0 Hz, 1H), 2.77 (s, 1H), 2.64 (s, 3H), 2.44 (q, J = 2.0 Hz, 1H), 2.37 (s, 3H), 1.64 (s, 3H). ^{13}C NMR (151 MHz, Chloroform-d) δ 170.6, 163.8, 155.6, 149.8, 136.7, 136.6, 132.2, 130.9, 130.7, 130.4, 129.8 (2C), 128.7 (2C), 79.6, 74.7, 70.6, 70.4, 70.3, 69.8, 69.1, 58.4, 54.3, 39.4, 39.1, 14.4, 13.1, 11.8. LCMS RT = 6.518 min, A% = 98%, m/z [M+H] = 570.15. HRMS - $C_{28}H_{33}ClN_5O_4S$ – Calculated [M+H] = 570.1028. Experimental [M+H] = 570.1945 (ppm = +0.9).

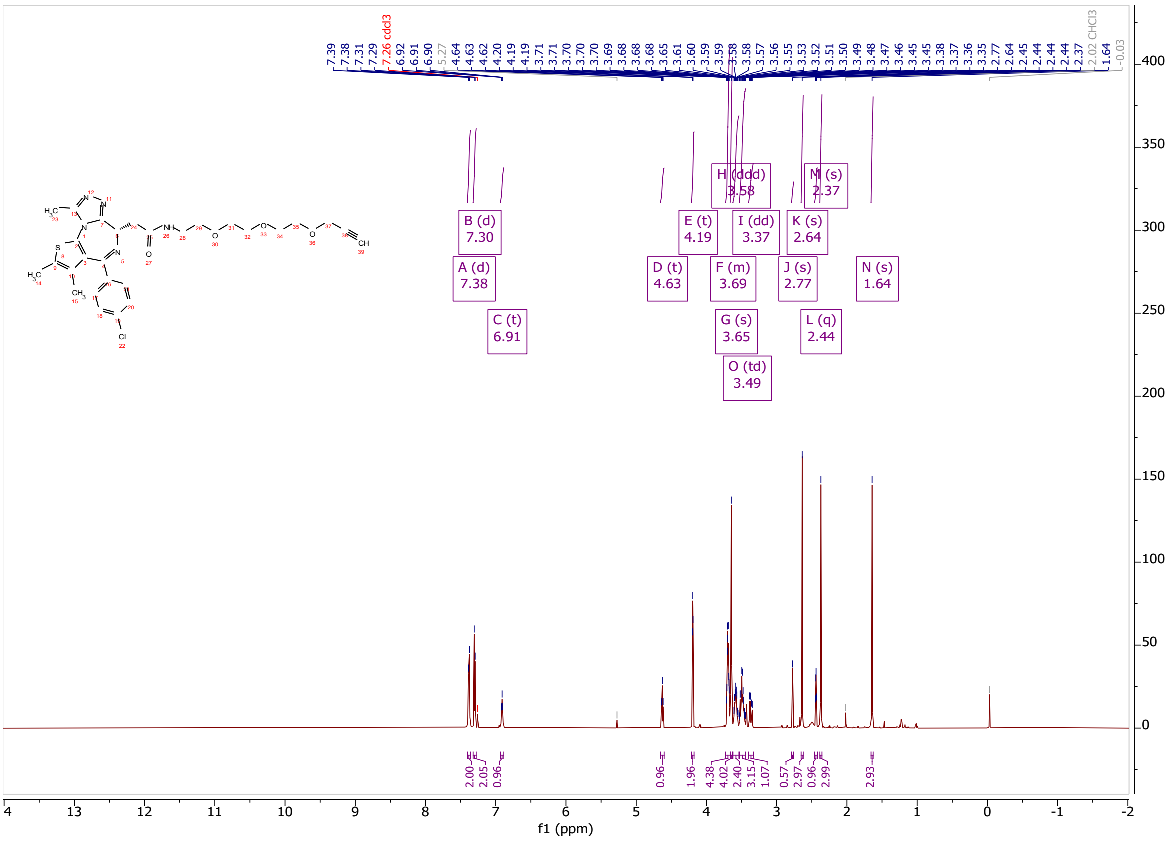

Figure S7 – ^{1}H spectra for compound **1b**.

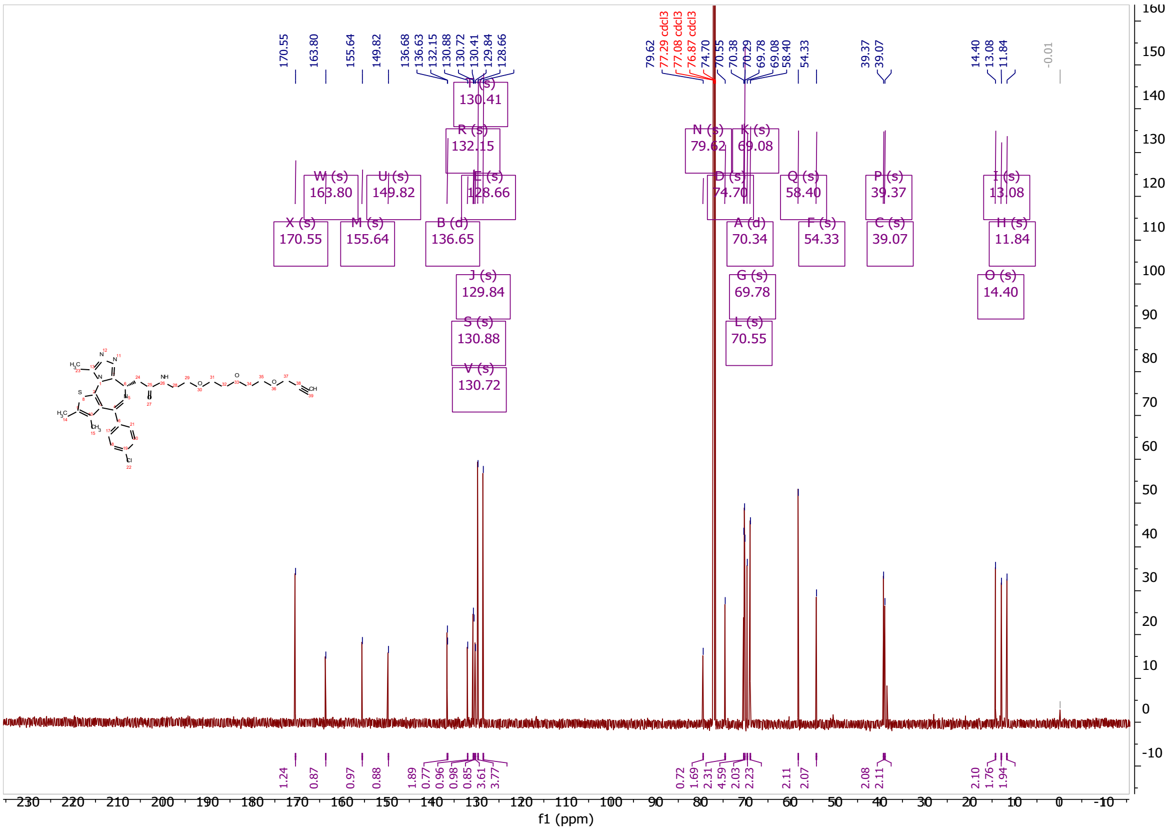

Figure S8– ^{13}C spectra for compound **1b**.

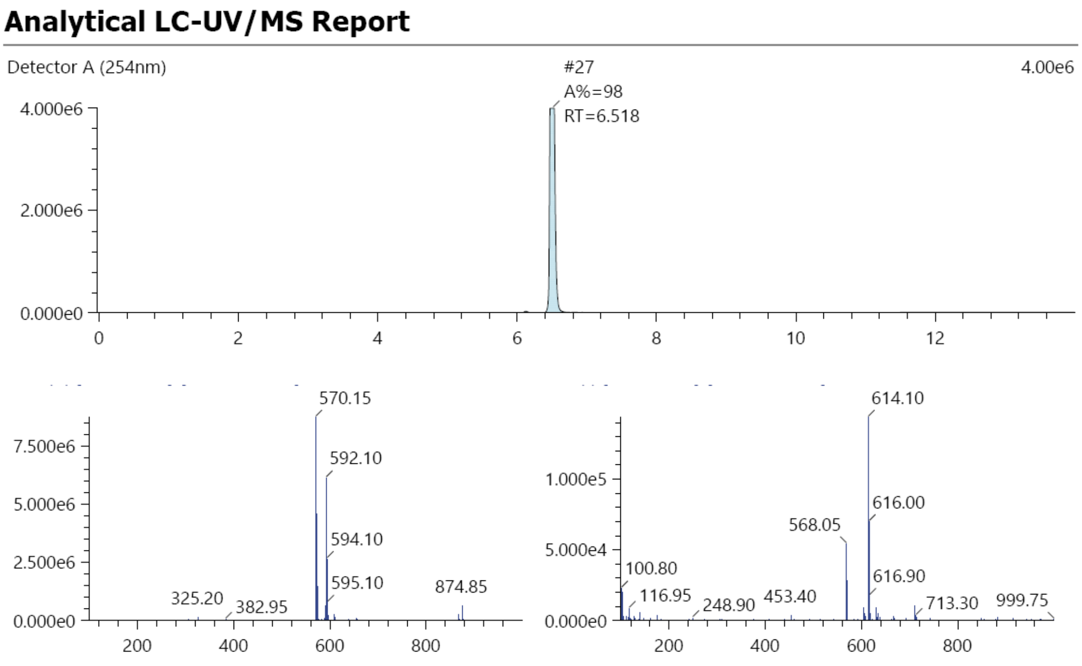

Figure S9 – LCMS analysis for compound **1b**.

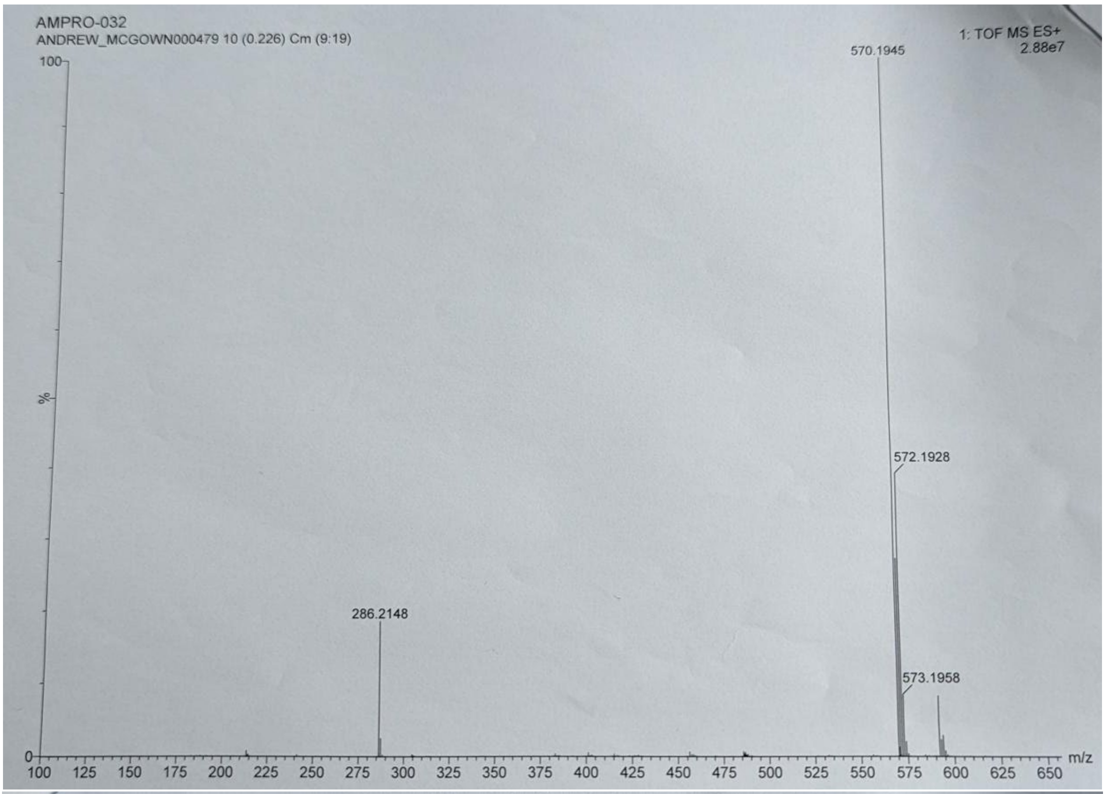

Single Mass Analysis
Tolerance = 100.0 PPM / DBE: min = -25.0, max = 75.0
Element prediction: Off
Number of isotope peaks used for i-FIT = 3

Monoisotopic Mass, Odd and Even Electron Ions
8 formula(e) evaluated with 1 results within limits (up to 50 closest results for each mass)
Elements Used:
C: 28-28 H: 0-100 N: 5-5 O: 4-4 S: 1-1 35Cl: 0-8
AMPRO-032
ANDREW_MCGOWN000479 10 (0.226) AM2 (Ar,20000.0,0.00,0.00); Cm (9:19)
1: TOF MS ES+
7.99e+007

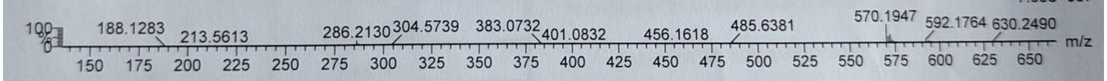

Minimum:				-25.0				
Maximum:		5.0	100.0	75.0				
Mass	Calc. Mass	mDa	PPM	DBE	i-FIT	Norm	Conf(%)	Formula
570.1947	570.1942	0.5	0.9	14.5	749.7	n/a	n/a	C28 H33 N5 O4 S 35Cl

Figure S10 – HRMS analysis for compound **1b**.

1c AMKV-049/AMPRO-033

(S)-2-(4-(4-chlorophenyl)-2,3,9-trimethyl-6H-thieno[3,2-f][1,2,4]triazolo[4,3-a][1,4]diazepin-6-yl)-N-(3,6,9,12-tetraoxapentadec-14-yn-1-yl)acetamide.

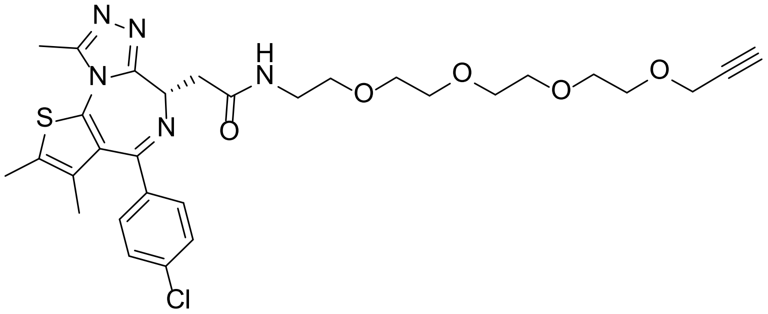

Chemical Formula: $C_{30}H_{36}ClN_5O_5S$
Exact Mass: 613.2126
Molecular Weight: 614.1553

Using the general method **1c** was isolated as yellow, viscous oil (247.5mg, 0.40mmol, 65%).

^{1}H NMR (600 MHz, Chloroform-d) δ 7.39 (d, J = 8.0 Hz, 2H), 7.31 (d, J = 8.0 Hz, 2H), 7.00 (t, J = 5.5 Hz, 1H), 4.63 (t, J = 7.0 Hz, 1H), 4.17 (d, J = 2.5 Hz, 2H), 3.69 – 3.66 (m, 8H), 3.64 (d, J = 5.5 Hz, 4H), 3.59 – 3.55 (m, 2H), 3.54 – 3.44 (m, 4H), 3.36 (dd, J = 14.5, 7.0 Hz, 1H), 2.65 (s, 3H), 2.38 (s, 3H), 1.65 (s, 3H). ^{13}C NMR (151 MHz, Chloroform-d) δ 170.7, 163.8, 155.7, 149.8, 136.7, 136.6, 132.1, 130.9, 130.8, 130.4, 129.9 (2C), 128.7 (2C), 79.6, 74.6, 70.5, 70.4, 70.3, 70.2, 70.0. 68.9. 60.4, 58.4, 54.3, 39.4, 39.1, 14.4, 13.1, 11.8. LCMS – RT 6.209 min, A% = 96%, Mw [M+H] = 614.15, [M+Na] = 636.10. Mw [M-H] = 612.10. HRMS – $C_{30}H_{36}ClN_5O_5S$ – Calculated [M+H] = 614.2204. Experimental [M+H] = 614.2215 (ppm = +2.0).

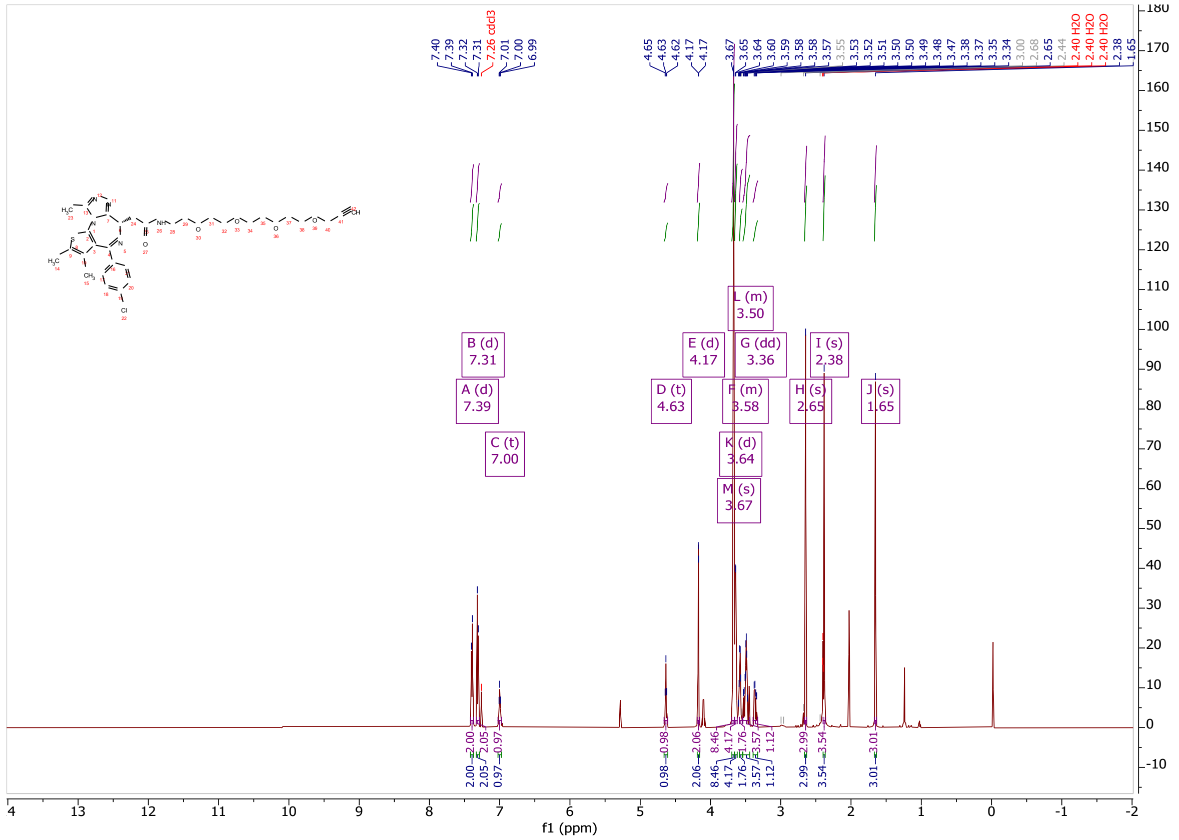

Figure S11 – ^{1}H spectra for compound **1c**.

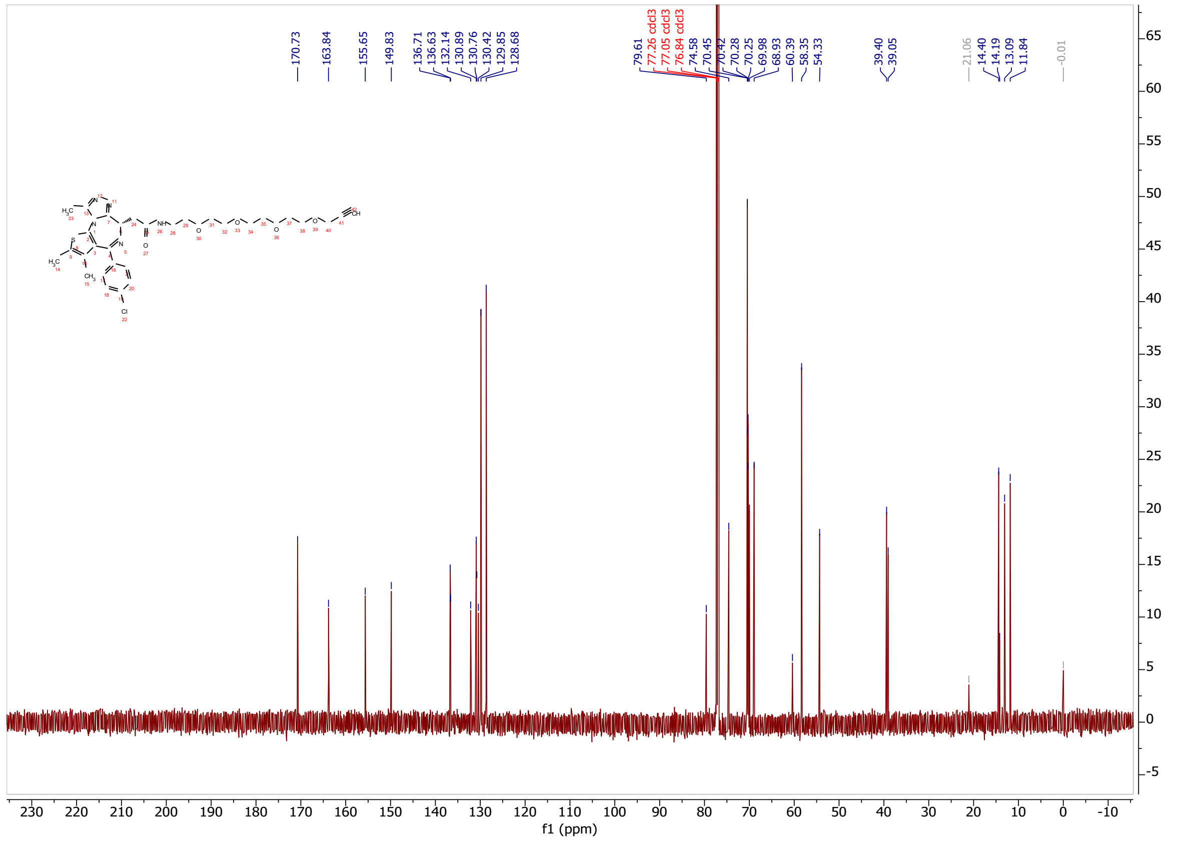

Figure S12 – ^{13}C spectra for compound **1c**.

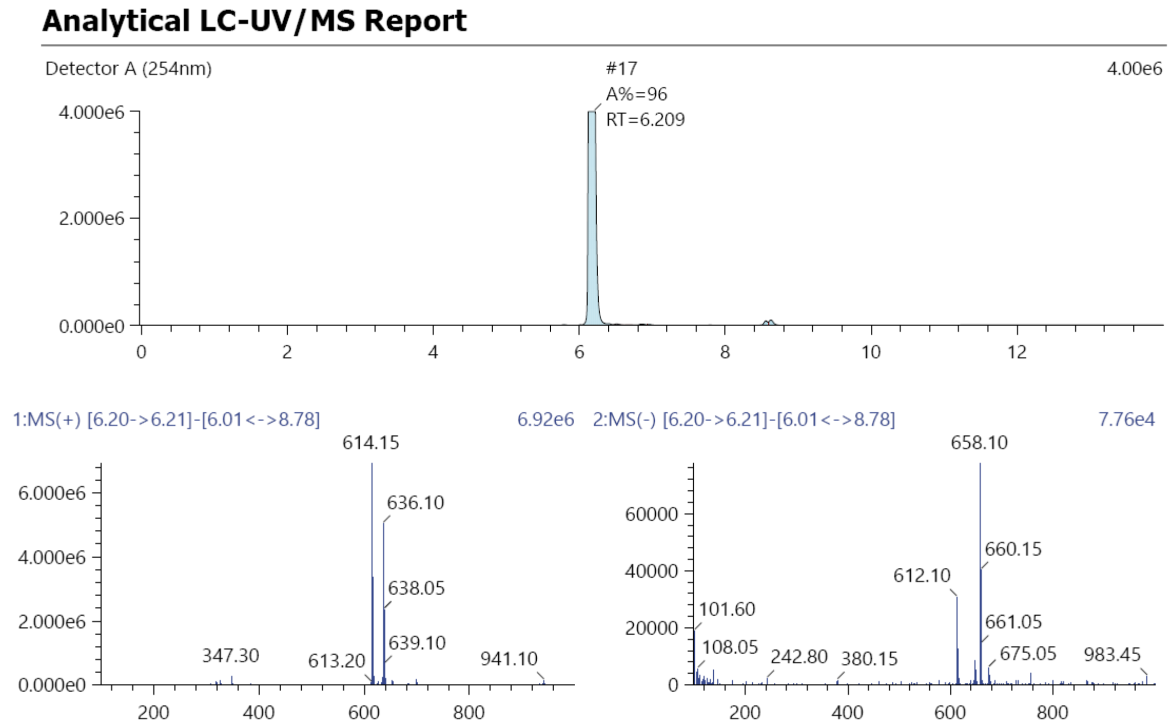

Figure S13 – LCMS analysis for compound **1c**.

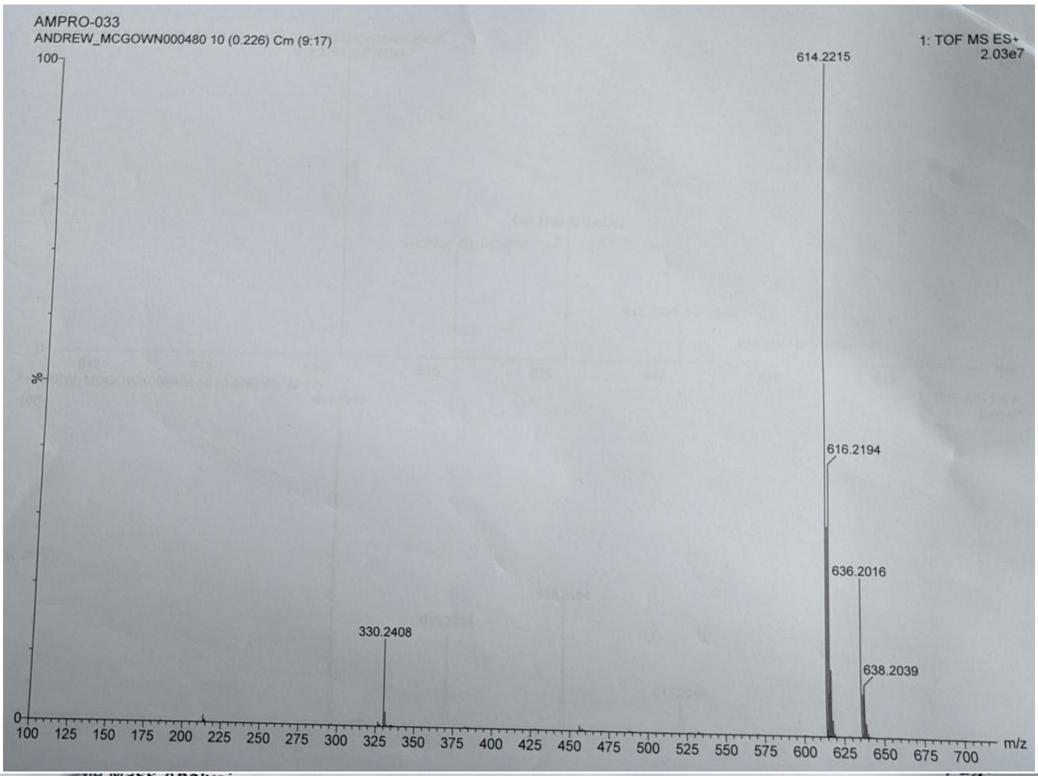

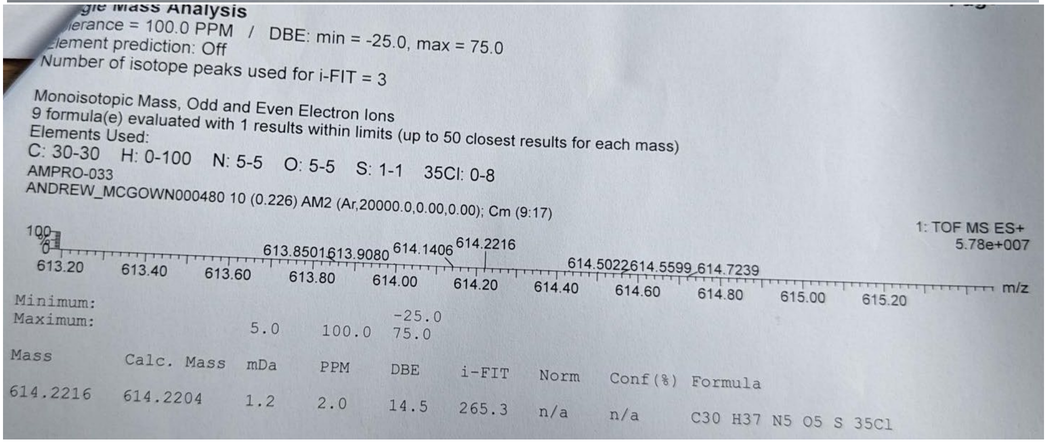

Mass	Calc. Mass	mDa	PPM	DBE	i-FIT	Norm	Conf(%)	Formula
Minimum:				-25.0				
Maximum:		5.0	100.0	75.0				
614.2216	614.2204	1.2	2.0	14.5	265.3	n/a	n/a	C30 H37 N5 O5 S 35Cl

Figure S14 – HRMS analysis for compound **1c**.

1d AMKV-044/AMPRO-030

(S)-2-(4-(4-chlorophenyl)-2,3,9-trimethyl-6H-thieno[3,2-f][1,2,4]triazolo[4,3-a][1,4]diazepin-6-yl)-N-(2-(2-(prop-2-yn-1-yloxy)ethoxy)ethyl)acetamide.

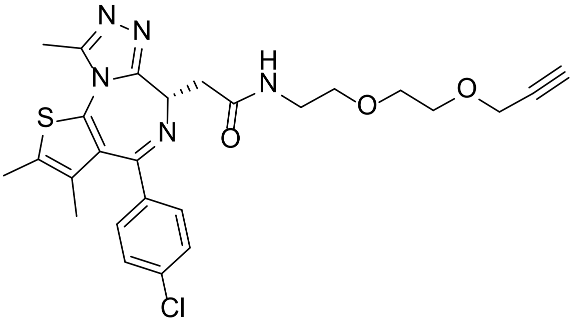

Chemical Formula: $C_{26}H_{28}ClN_5O_3S$
Exact Mass: 525.1601
Molecular Weight: 526.0502

Using the general method **1d** was isolated as a yellow viscous oil (283.8mg, 0.54mmol, 87%).

^{1}H NMR (600 MHz, Chloroform-d) δ 7.39 (d, J = 8.0 Hz, 2H), 7.32 (d, J = 8.0 Hz, 2H), 6.96 (t, J = 5.5 Hz, 1H), 4.64 (t, J = 7.0 Hz, 1H), 4.21 (d, J = 2.5 Hz, 2H), 3.73 – 3.68 (m, 2H), 3.68 – 3.64 (m, 2H), 3.59 (m, 2H), 3.54 – 3.48 (m, 3H), 3.37 (dd, J = 14.5, 7.0 Hz, 1H), 2.65 (s, 3H), 2.48 (t, 2.5 Hz, 1H), 2.38 (s, 3H), 1.65 (s, 3H). ^{1}H NMR spectrum contains some residual DCM and MeOH, used as such. ^{13}C NMR (151 MHz, Chloroform-d) δ 170.7, 164.1, 155.7, 150.0, 136.9, 136.6, 132.1, 131.0, 130.9, 130.6, 129.9 (2C), 128.8 (2C), 79.6, 75.0, 70.2, 69.9, 69.2, 58.5, 54.4, 39.5, 39.0, 14.5, 13.2, 11.9. LCMS RT – 6.126 min, A% = 95%, Mw [M+H] = 526.15. HRMS - $C_{26}H_{28}ClN_5O_3S$ – Calculated [M+H] = 526.1680. Experimental [M+H] = 526.1713 (ppm = +2.5).

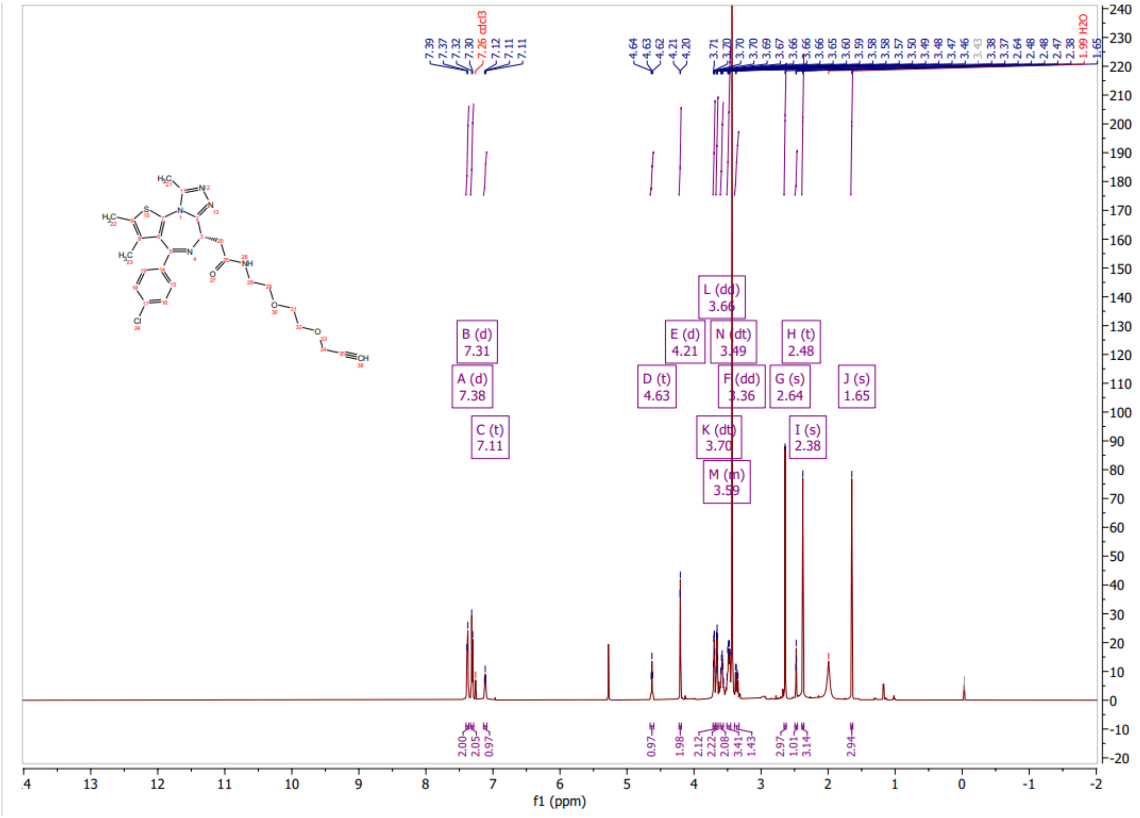

Figure S15 – ^{1}H spectra for compound **1d**.

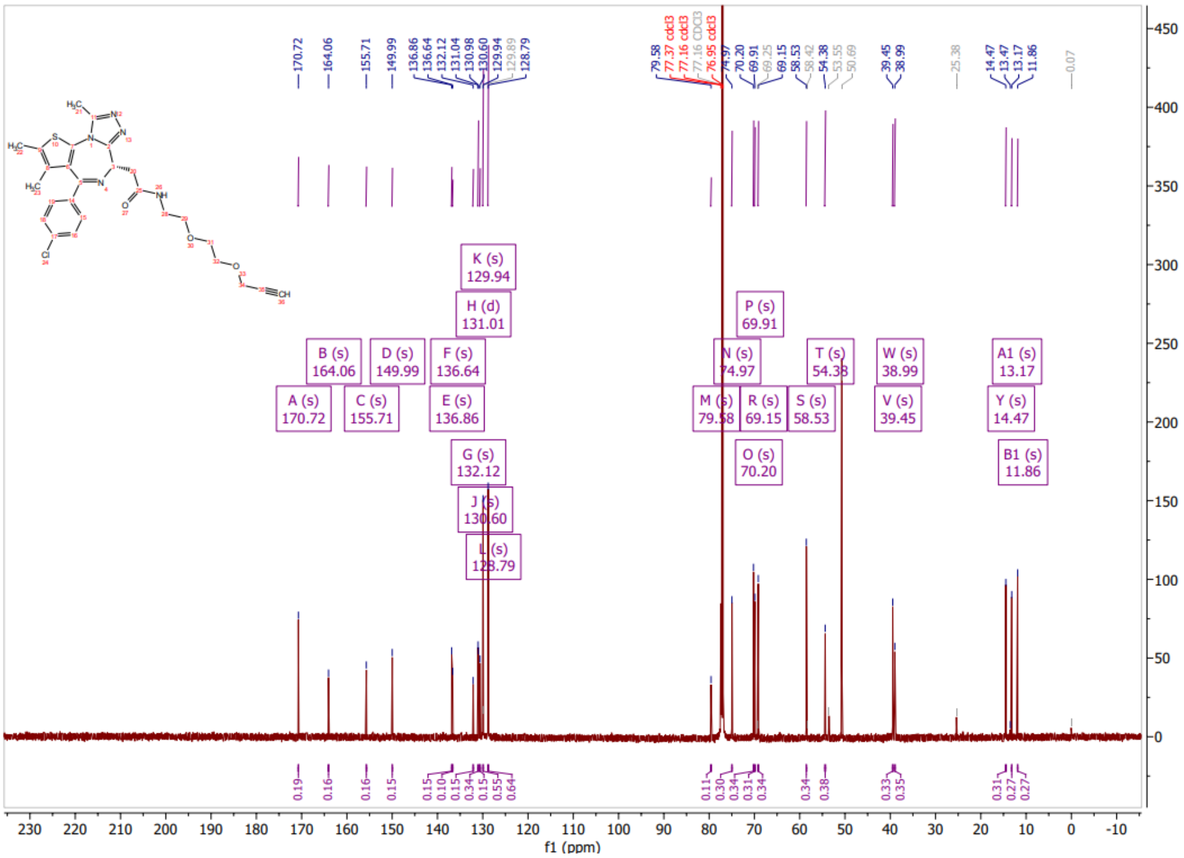

Figure S16 – ^{13}C spectra for compound **1d**.

Analytical LC-UV/MS Report

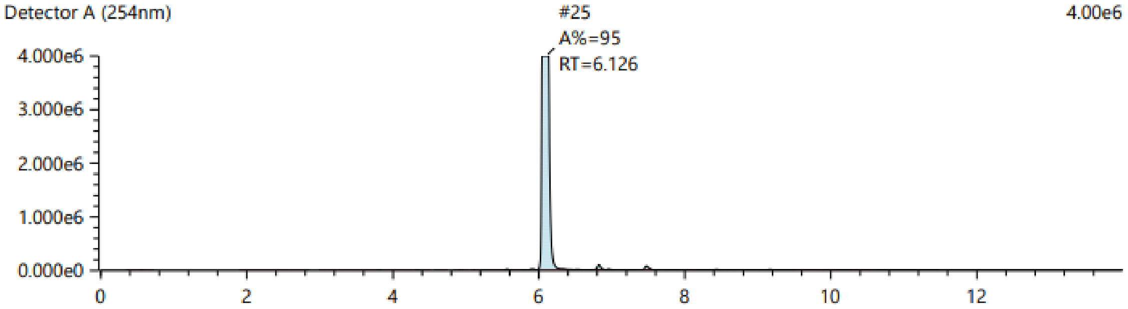

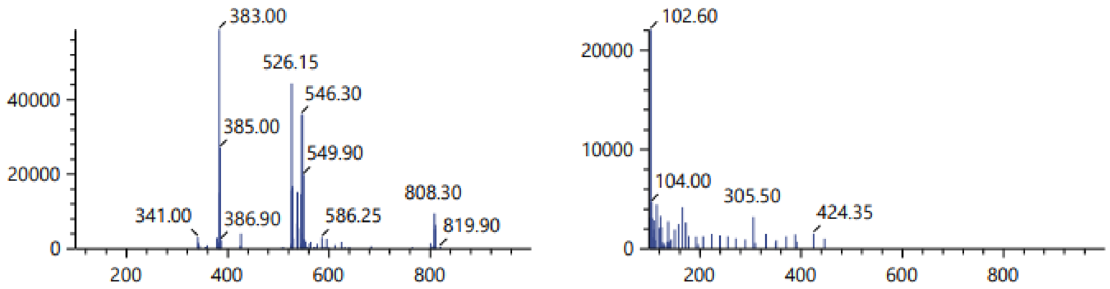

Figure S17 – LCMS analysis for compound **1d.**

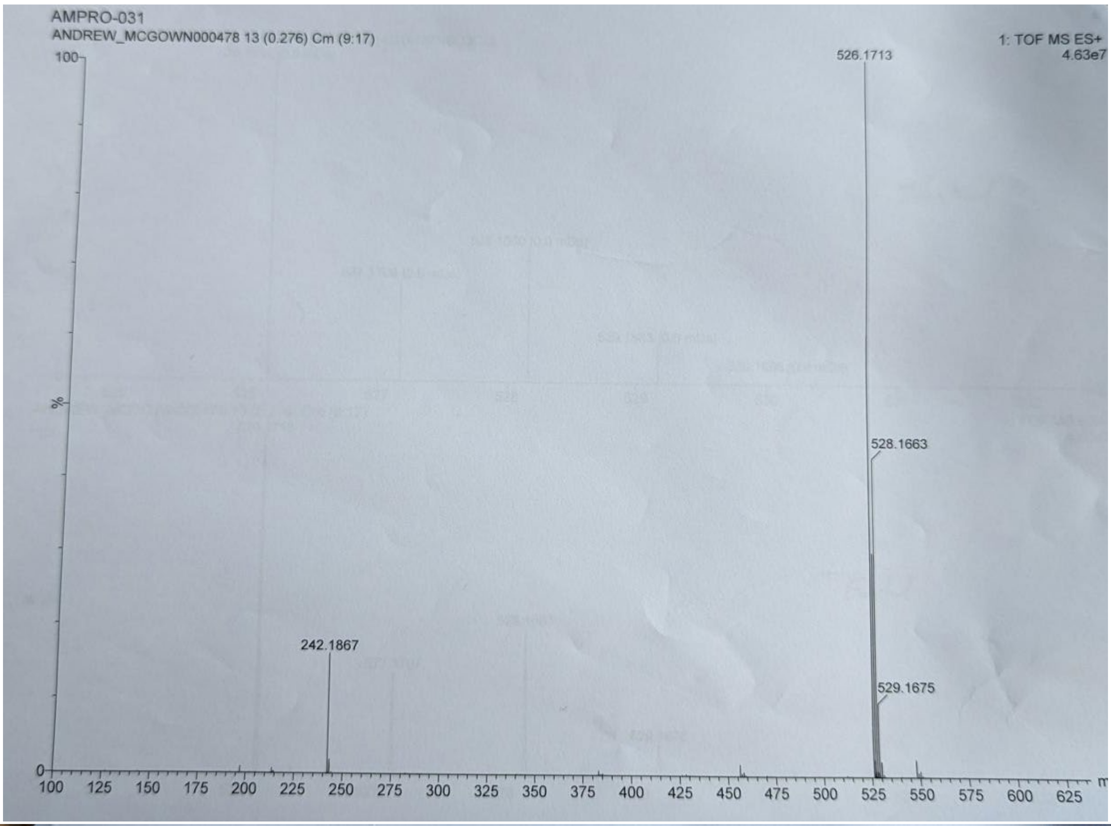

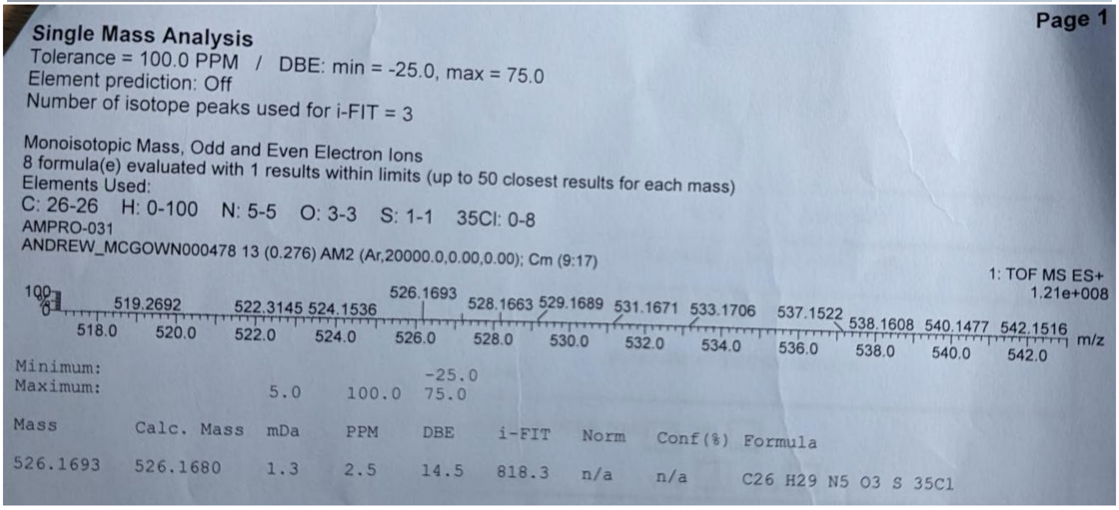

Single Mass Analysis Page 1

Tolerance = 100.0 PPM / DBE: min = -25.0, max = 75.0
Element prediction: Off
Number of isotope peaks used for i-FIT = 3

Monoisotopic Mass, Odd and Even Electron Ions
8 formula(e) evaluated with 1 results within limits (up to 50 closest results for each mass)
Elements Used:
C: 26-26 H: 0-100 N: 5-5 O: 3-3 S: 1-1 35Cl: 0-8
AMPRO-031
ANDREW_MCGOWN000478 13 (0.276) AM2 (Ar,20000.0,0.00,0.00); Cm (9:17)

1: TOF MS ES+
1.21e+008

519.2692 522.3145 524.1536 526.1693 528.1663 529.1689 531.1671 533.1706 537.1522 538.1608 540.1477 542.1516 m/z

Minimum:				-25.0					
Maximum:		5.0	100.0	75.0					
Mass	Calc. Mass	mDa	PPM	DBE	i-FIT	Norm	Conf(%)	Formula	
526.1693	526.1680	1.3	2.5	14.5	818.3	n/a	n/a	C26 H29 N5 O3 S 35Cl	

Figure S18 – HRMS analysis for compound **1d**.

1e - AMKV-181

(S)-tert-butyl (2-(2-(4-(4-chlorophenyl)-2,3,9-trimethyl-6H-thieno[3,2-f][1,2,4]triazolo[4,3-a][1,4]diazepin-6-yl)acetamido)ethyl)carbamate.

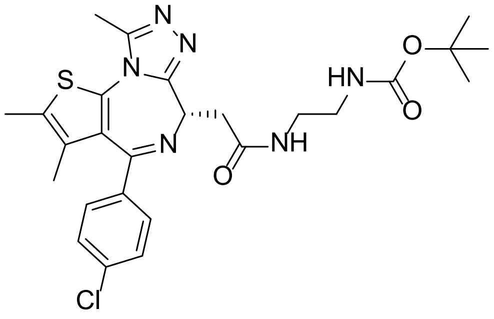

Chemical Formula: $C_{26}H_{31}ClN_6O_3S$
Exact Mass: 542.1867
Molecular Weight: 543.0807

Using the general method **1e** was isolated as a yellow solid (244mg, 0.45mmol, 73% yield).

^{1}H NMR (600 MHz, Chloroform-*d*) δ 7.65 (s, 1H), 7.37 (d, *J* = 8.0 Hz, 2H), 7.31 – 7.24 (m, 2H), 5.62 (s, 1H), 4.66 (t, *J* = 7.0 Hz, 1H), 3.53 (dd, *J* = 14.5, 7.5 Hz, 1H), 3.42 (m, 1H), 3.34 – 3.23 (m, 2H), 2.94 (d, *J* = 5.0 Hz, 1H), 2.66 (s, 3H), 2.38 (s, 3H), 1.65 (s, 3H), 1.40 (d, *J* = 7.0 Hz, 1H), 1.38 (s, 9H). Some residual EtOAc in spectra – used as such. ^{13}C NMR (151 MHz, Chloroform-d) δ 171.1, 164.0, 156.4, 155.7, 150.0, 136.8 (2C), 136.5, 132.0, 131.0, 130.5, 129.9 (2C), 128.7 (2C), 79.2, 54.3, 40.6, 40.0, 39.0, 28.4 (3C), 14.4, 13.1, 11.8. LCMS RT 6.501 min, A% = 96%, Mw [M+H] = 543.00 and [M+H] = 443.00 (deprotected). HRMS – $C_{26}H_{31}ClN_6O_3S$ – Calculated [M+H] = 543.1945. Experimental [M+H] = 543.1906 (ppm = - 3.7).

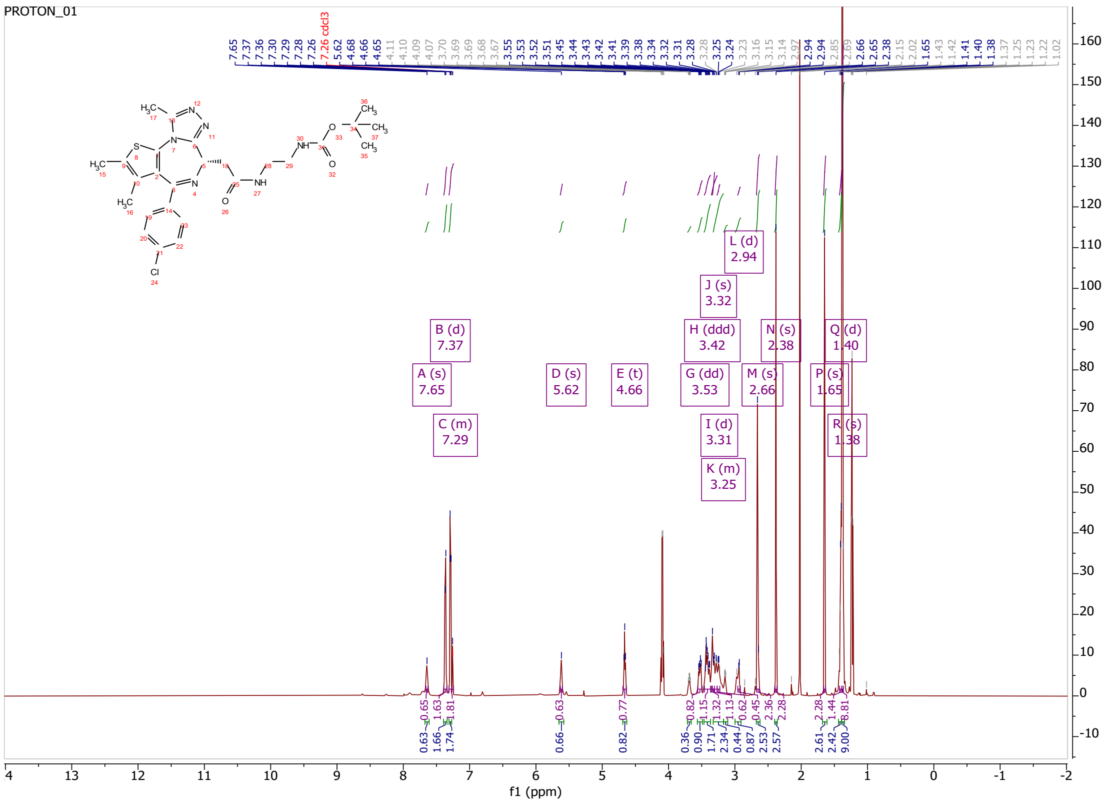

Figure S19 – ^{1}H spectra for compound **1e**.

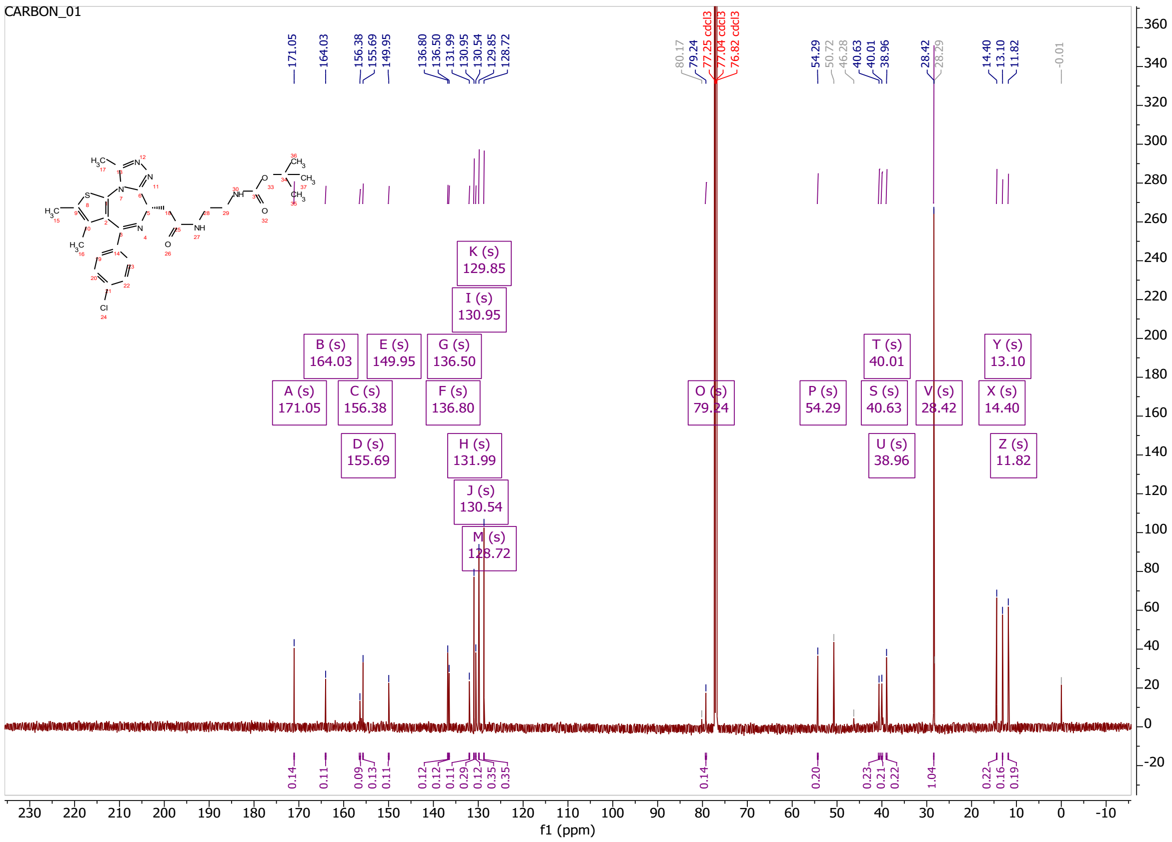

Figure S20 – ^{13}C spectra for compound **1e**.

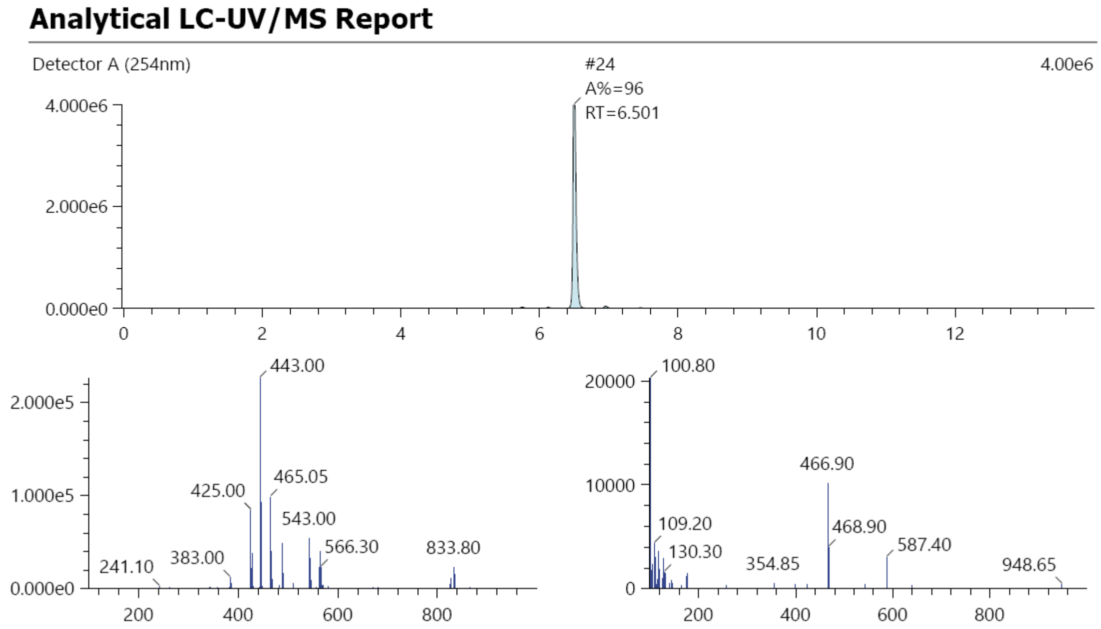

Figure S21 – LCMS analysis for compound **1e**.

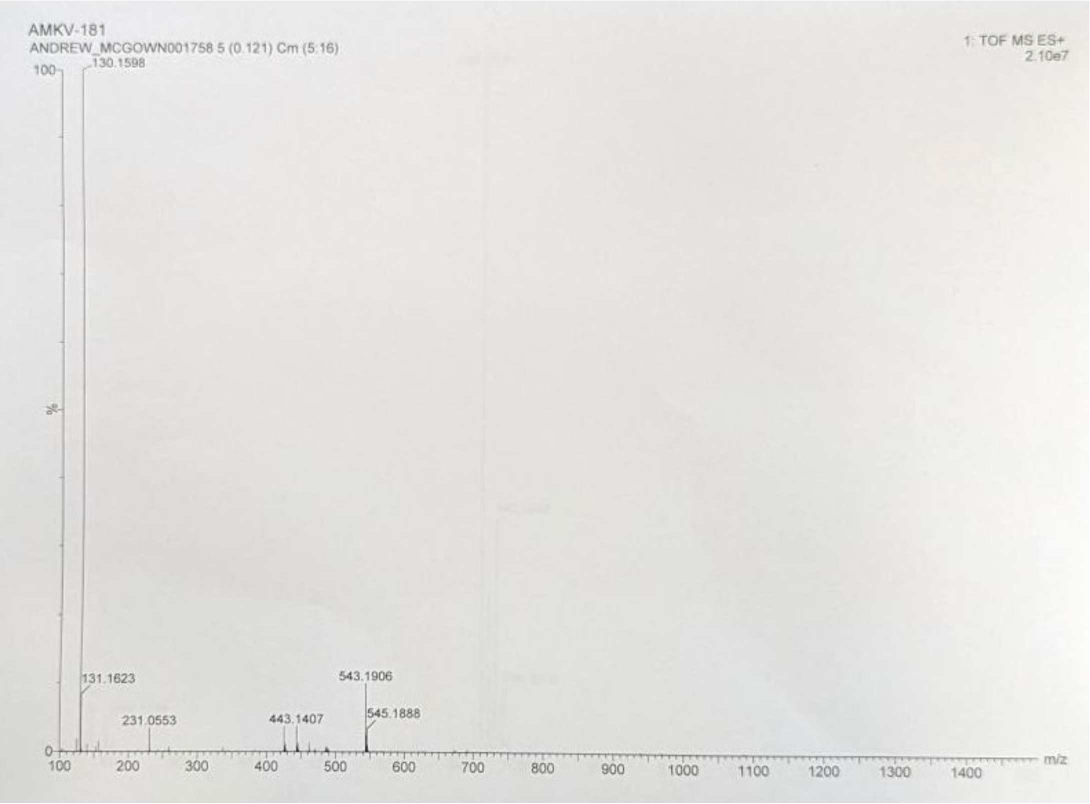

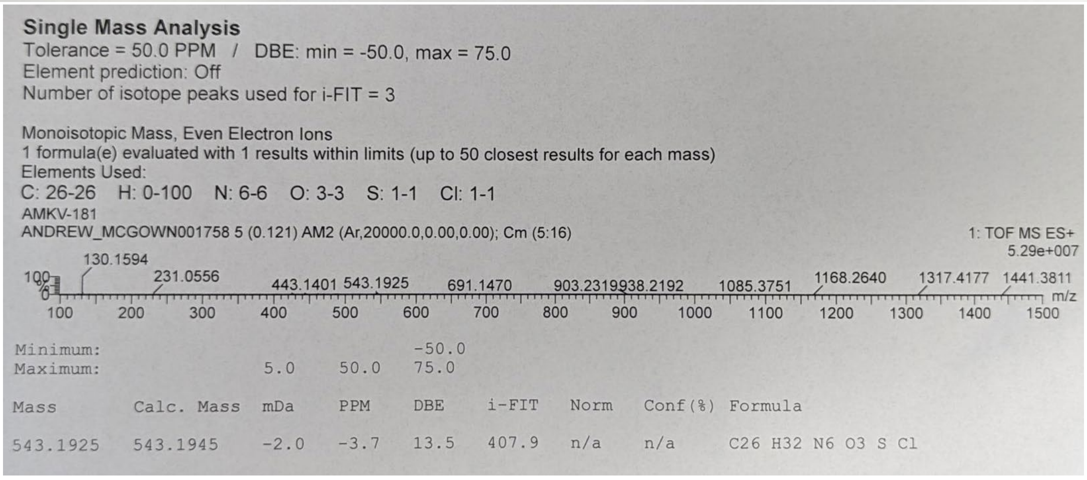

Single Mass Analysis
Tolerance = 50.0 PPM / DBE: min = -50.0, max = 75.0
Element prediction: Off
Number of isotope peaks used for i-FIT = 3

Monoisotopic Mass, Even Electron Ions
1 formula(e) evaluated with 1 results within limits (up to 50 closest results for each mass)
Elements Used:
C: 26-26 H: 0-100 N: 6-6 O: 3-3 S: 1-1 Cl: 1-1
AMKV-181
ANDREW_MCGOWN001758 5 (0.121) AM2 (Ar,20000.0,0.00,0.00); Cm (5:16)
1: TOF MS ES+
5.29e+007

130.1594 231.0556 443.1401 543.1925 691.1470 903.2319938.2192 1085.3751 1168.2640 1317.4177 1441.3811

Minimum:				-50.0				
Maximum:		5.0	50.0	75.0				
Mass	Calc. Mass	mDa	PPM	DBE	i-FIT	Norm	Conf(%)	Formula
543.1925	543.1945	-2.0	-3.7	13.5	407.9	n/a	n/a	C26 H32 N6 O3 S Cl

Figure S22 – HRMS analysis for compound **1e**.

1f- AMKV-187

(S)-N-(2-aminoethyl)-2-(4-(4-chlorophenyl)-2,3,9-trimethyl-6H-thieno[3,2-f][1,2,4]triazolo[4,3-a][1,4]diazepin-6-yl)acetamide.

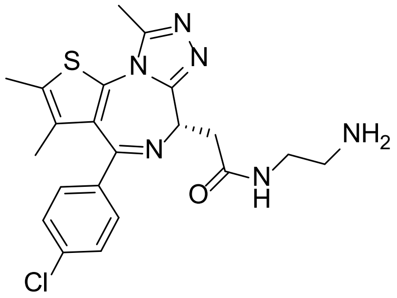

Chemical Formula: $C_{21}H_{23}ClN_6OS$
Exact Mass: 442.1343
Molecular Weight: 442.9649

1f (250 mg, 0.46 mmol, 1.0 eq) was dissolved in DCM (5 mL) and treated with 4N HCl in 1,4-dioxane (2 mL), the reaction mixture was stirred for 12 hours at room temperature until completion. The solution was concentrated to residue and suspended in methanol (3 mL) and passed through an Isolute Si-SCX2 column (catch and release). **2** was isolated as a pale-yellow solid in quantitative yields (200 mg, 0.46 mmol, 99 %). ^{1}H NMR (600 MHz, DMSO-*d*$_6$) δ 8.56 (t, *J* = 5.5 Hz, 1H), 8.12 (t, *J* = 6.0 Hz, 2H), 7.50 (d, *J* = 8.0 Hz, 2H), 7.42 (d, *J* = 8.0 Hz, 2H), 4.56 (t, *J* = 7.0 Hz, 1H), 3.35 (q, *J* = 6.0 Hz, 2H), 3.28 (d, *J* = 7.0 Hz, 2H), 2.86 (q, *J* = 6.5 Hz, 2H), 2.63 (s, 3H), 2.40 (s, 3H), 1.59 (s, 3H). ^{13}C NMR (151 MHz, DMSO-*d*$_6$) δ 170.5, 164.1, 155.2, 150.9, 136.6, 136.0, 132.5, 131.9, 130.9 (2C), 130.5, 130.1, 129.0 (2C), 53.8, 38.9, 37.6, 36.8, 14.5, 13.2, 11.7. LCMS RT 6.501 min, A% = 93% single major peak, Mw [M+H] = 443.00, [M+Na] = 465.00. Mw [M-H] = 441.05. HRMS – $C_{21}H_{23}ClN_6OS$ – Calculated [M+H] = 443.1421. Experimental [M+H] = 443.1407 (ppm = - 0.9).

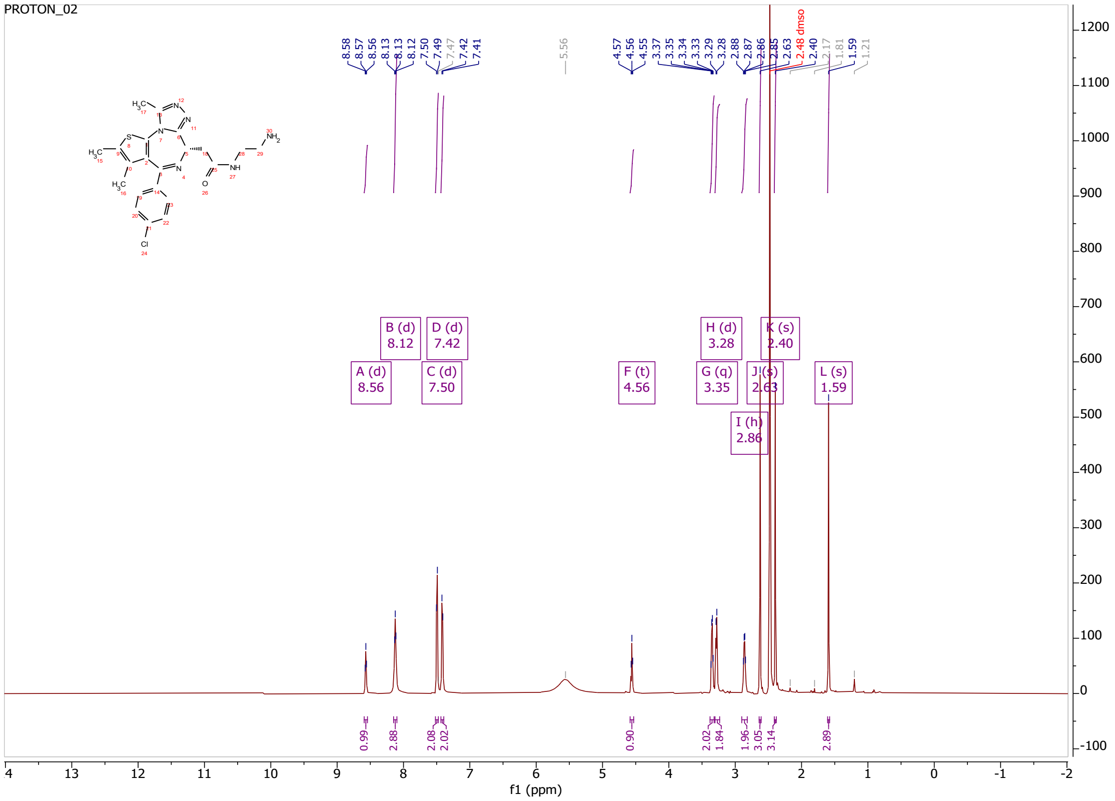

Figure S23 – ^{1}H spectra for compound **1f.**

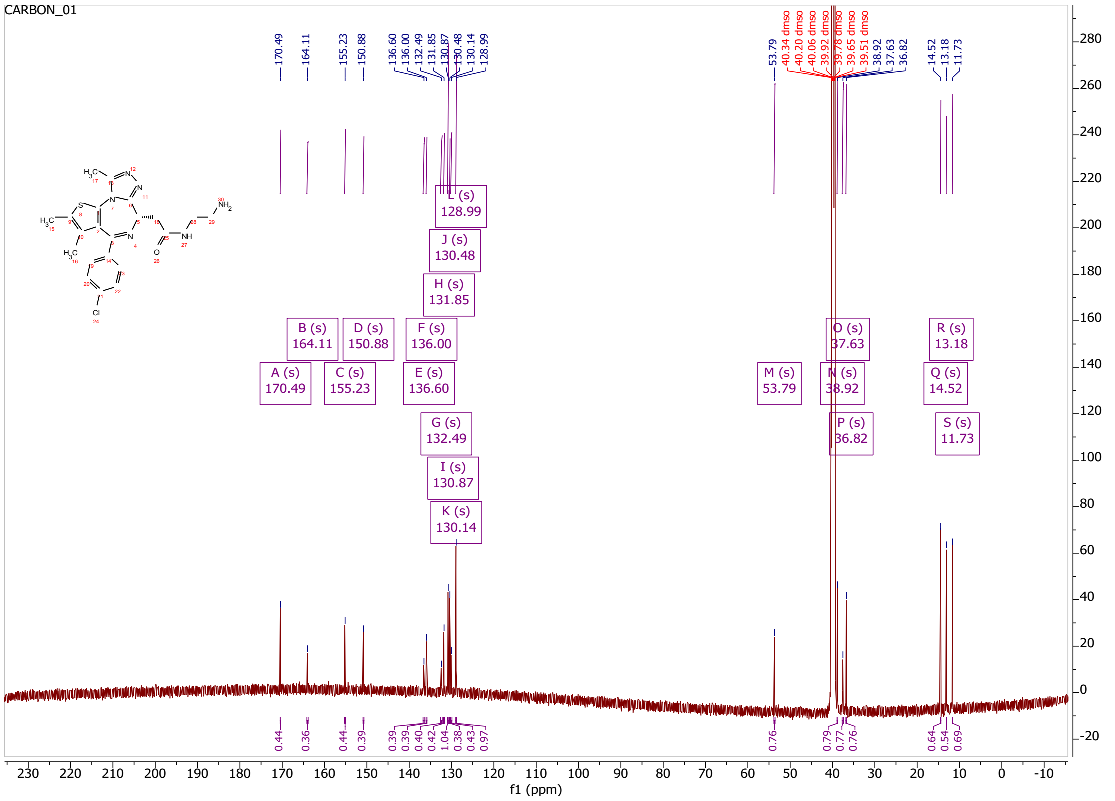

Figure S24 – ^{13}C spectra for compound **1f**.

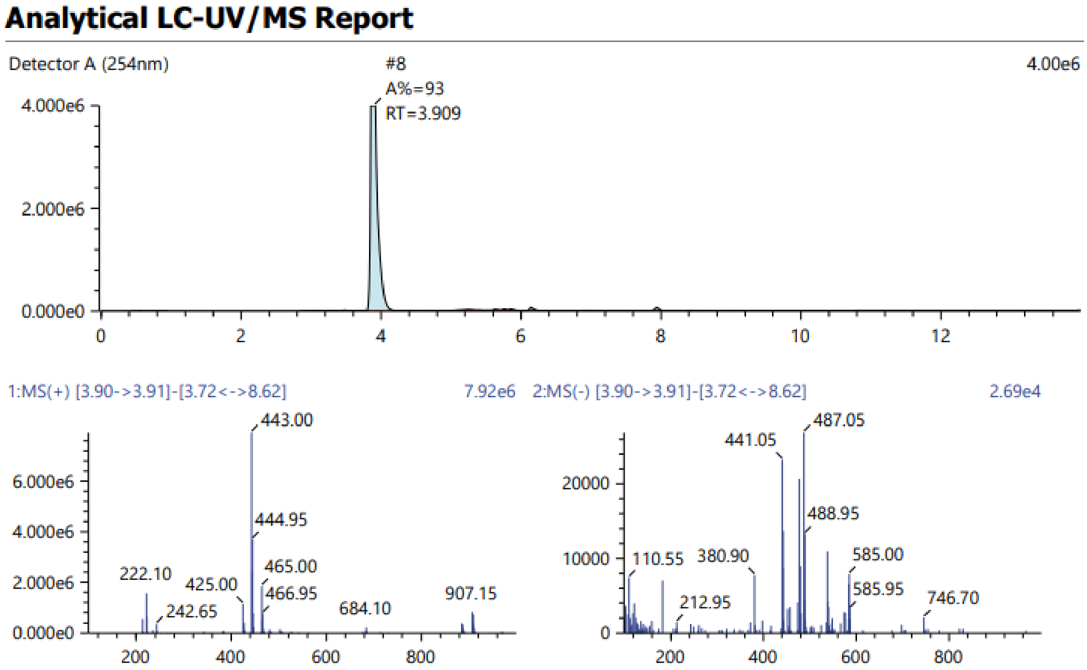

Figure S25 – LCMS analysis for compound **1f.**

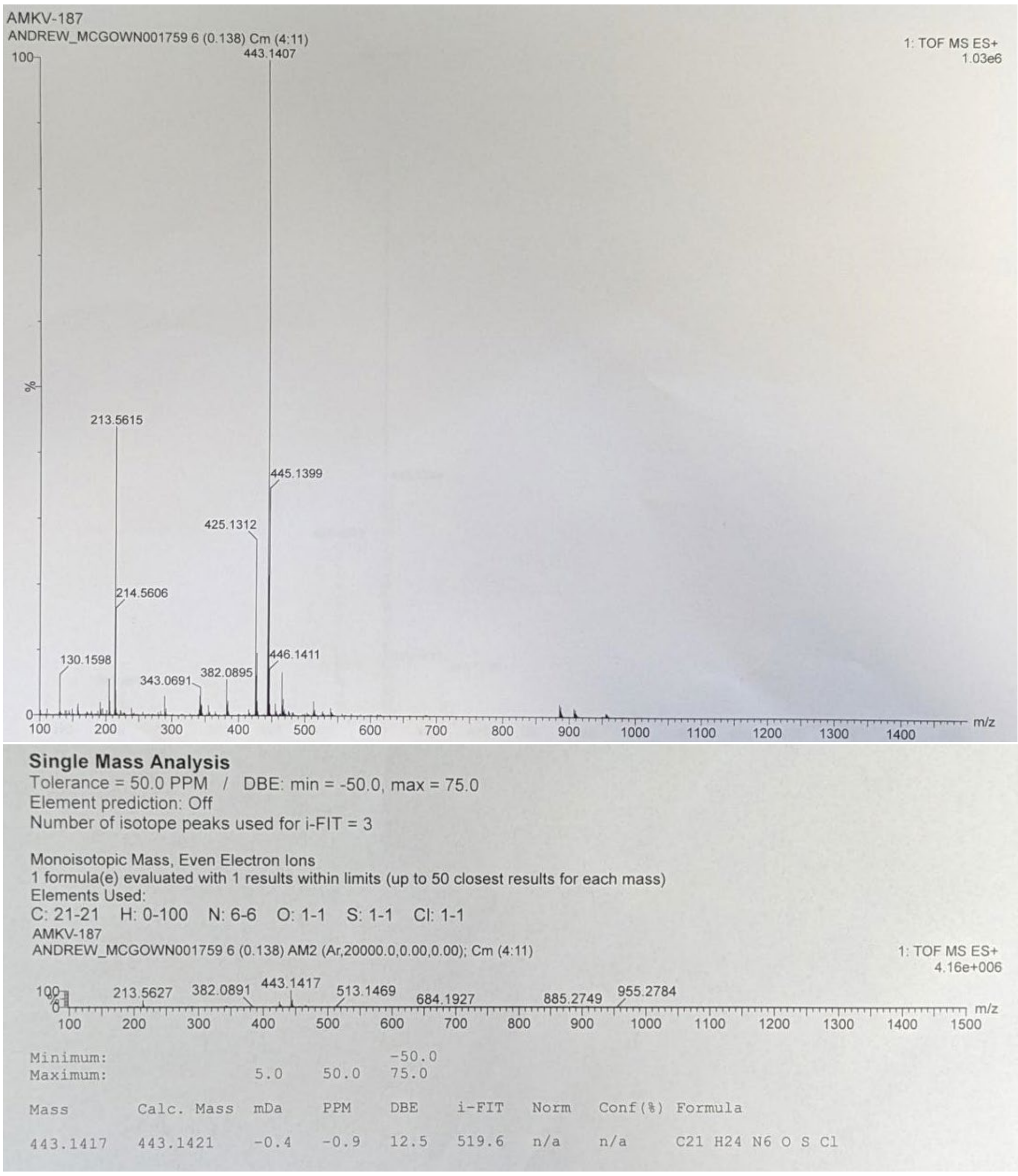

Figure S26 – HRMS analysis for compound **1f**.

A3 Protocol

In a microwave vial, the aldehyde (1.0mmol, 1.0eq), amine (1.0mmol, 1.0eq) and copper (II) bromide (0.1mmol, 0.1eq) were suspended in acetonitrile (3.0mL). The reaction vial was sealed, purged with nitrogen, and sonicated for 5 minutes leading to an immediate colour change, for some examples, a precipitate may form. Alkyne (1.0 mmol, 1.0 eq) was added and the reaction was heated to 60°C for 2 hours in a microwave reactor (300W). Upon completion, after cooling, the reaction mixture was concentrated to a residue before resuspending in ethyl acetate (20mL). The organic extracts were washed with aq. sat. $NaHCO_3$ (3 x 20mL), sat. brine (30mL) and dried over $MgSO_4$. The organics were concentrated to yield a viscous semi-solid, which was purified by column chromatography (SiO_2, 12g, petroleum ether: ethyl acetate).

2a AMKV-092/AMPRO-236

tert-butyl (1-(1-cyclohexyl-5-hydroxypent-2-yn-1-yl)piperidin-4-yl)carbamate.

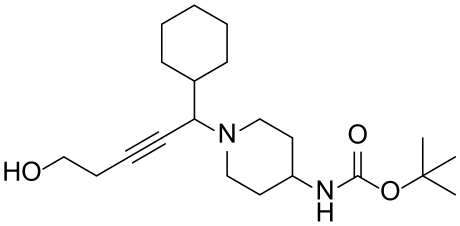

Chemical Formula: $C_{21}H_{36}N_2O_3$
Exact Mass: 364.2726
Molecular Weight: 364.5221

Using the general method **2a** was synthesised from cyclohexanecarboxaldehyde (2976mg, 3214μL, 26.5mmol, 1.0 eq), 4-N-BOC-aminopiperidine (5315mg, 26.5mmol, 1.0 eq) and 3-butyn-1-ol (1860mg, 2000μL, 26.5mmol, 1.0 eq). The solid product was recrystalised in hot petroleum ether and, upon cooling, pure **2a** was collected as a colourless, crystalline solid (4104.6mg, 10.7mmol, 43%,). ^{1}H NMR (600 MHz, Chloroform-d) δ 4.48 (s, 1H), 3.70 (t, J = 7.0 Hz, 2H), 3.44 (s, 1H), 2.92 – 2.86 dt, J = 10.0, 2.5 Hz, 1H), 2.71 – 2.64 (dd, J = 15.0, 5.0 Hz, 1H), 2.56 – 2.51 (m, 1H), 2.51 – 2.47 (m, 1H), 2.48 (d, J = 4.0 Hz, 1H), 2.49 – 2.43 (m, 1H), 2.19 – 2.12 (m, 1H), 1.97 (d, J = 12.0 Hz, 1H), 1.91 (d, J = 15.0 Hz, 4H), 1.74 - 1.71 (m, 2H), 1.67 – 1.62 (m, 1H), 1.43 (s, 9H), 1.34 (d, J = 10.0 Hz, 1H), 1.27 – 1.17 (m, 1H), 1.14 (m, 2H), 0.88 (m, 2H).^{13}C NMR (151 MHz, Chloroform-d) δ 155.2, 82.4, 79.2, 79.1, 63.3, 61.4, 45.6, 39.6, 32.9, 32.6, 31.1, 30.3, 28.4 (3C), 26.7 (2C), 26.1, 26.0, 23.1 (2C). HRMS $C_{21}H_{36}N_2O_3$ Calculated [M+H] = 365.2804. Experimental [M+H] 365.2784 (ppm = -0.3)

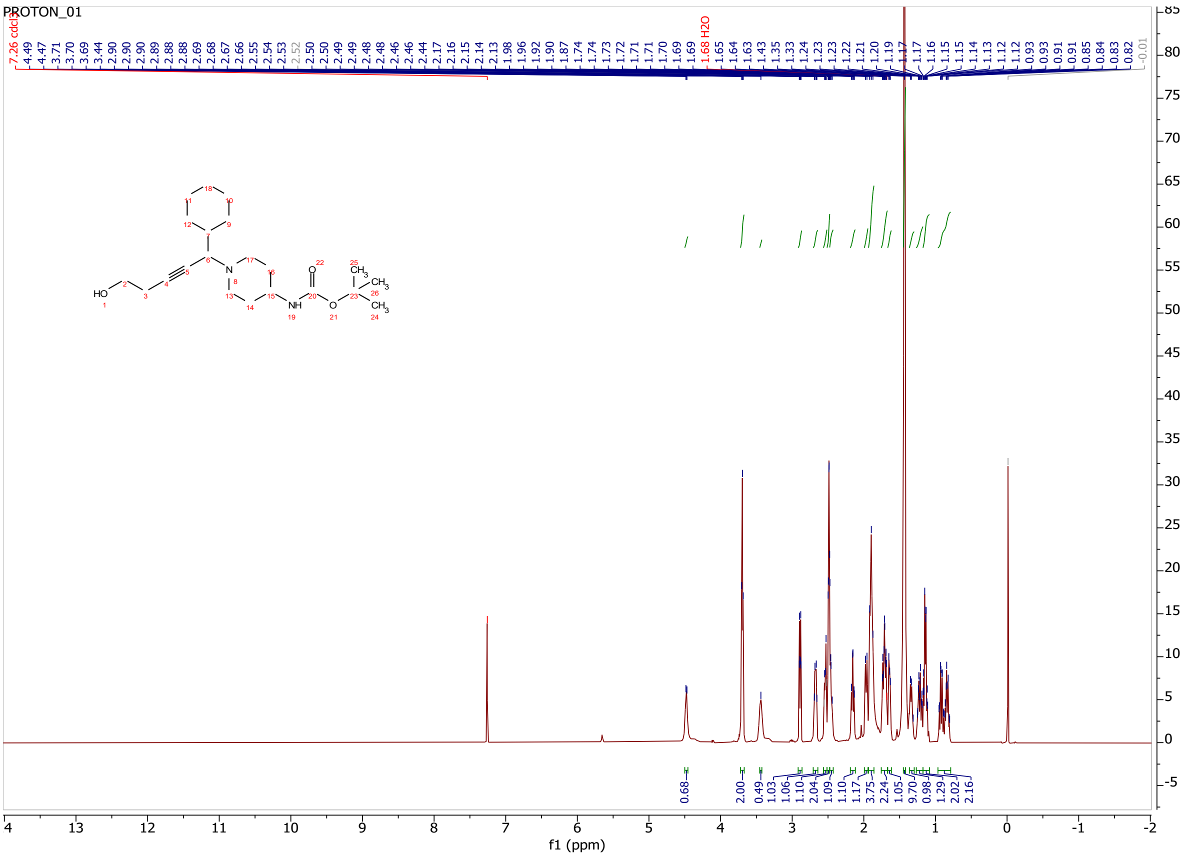

Figure S27 – ^{1}H spectra of compound **2a**.

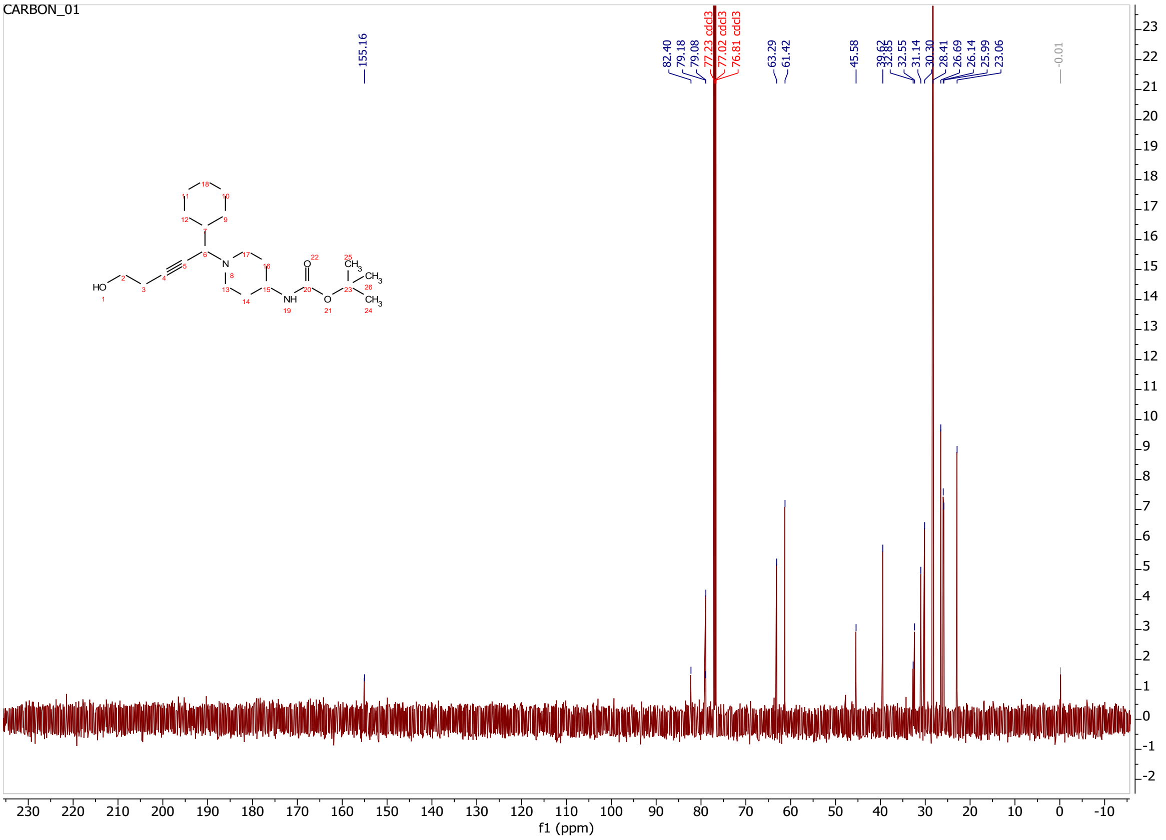

Figure S28 – ^{13}C spectra of compound **2a**.

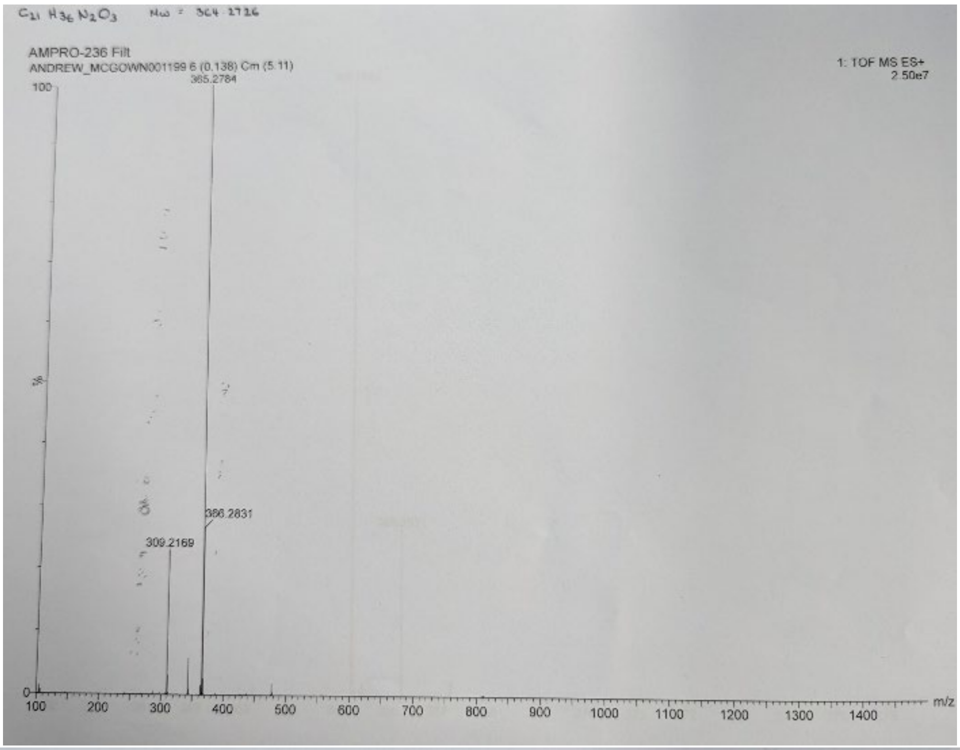

Elemental Composition Report **Page 1**

Single Mass Analysis
Tolerance = 100.0 PPM / DBE: min = -50.0, max = 100.0
Element prediction: Off
Number of isotope peaks used for i-FIT = 3

Monoisotopic Mass, Even Electron Ions
1 formula(e) evaluated with 1 results within limits (up to 50 closest results for each mass)
Elements Used:
C: 21-21 H: 0-100 N: 2-2 O: 3-3
AMPRO-236 Filt
ANDREW_MCGOWN001199 6 (0.138) AM2 (Ar,20000.0,0.00,0.00); Cm (5:11)
1: TOF MS ES+
7.34e+007

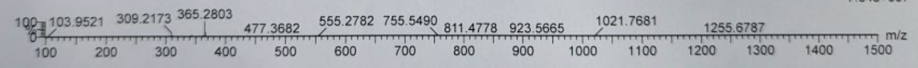

Minimum:				-50.0				
Maximum:		5.0	100.0	100.0				
Mass	Calc. Mass	mDa	PPM	DBE	i-FIT	Norm	Conf(%)	Formula
365.2803	365.2804	-0.1	-0.3	4.5	767.3	n/a	n/a	C21 H37 N2 O3

Figure S29 – HRMS analysis of compound **2a.**

**GENERAL INFORMATION**	
**Customer**	Sussex
**Sales Order Number**	2023-04 LSV-BTE
**Sample code (BT ref.)**	**US-16 1 / US-16 2**
**Sample description (Customer ref.)**	**US-16 1 / US-16 2**
**VCD-spectrometer**	ChiralIR w/ Dual*PEM*
**Report prepared by: (name / signature as needed)**	Jordan Nafie
**Report validated and signed by**	Rina K Dukor
**Date**	May 3, 2023
**RESULTS**	
Absolute Configuration of US-16 2 is **(R)**<br>Absolute Configuration of US-16 1 is **(S)**	Confidence Level: **99%**
**MEASUREMENT PARAMETERS**	
**Concentration**	10mg / 150uL
**Solvent**	$CDCl_3$
**Instrument Resolution**	4 cm^{-1}
**PEM setting**	1400 cm^{-1}
**Number of scans/Measurement time**	12 hours per enantiomer
**Sample cell**	BaF_2
**Path length**	100 µm
**CALCULATION DETAILS**	
**Molecular Mechanics Force Field**	**MMFF94 (Compute VOA)**
**DFT Software version**	**Gaussian '09**
**Number of conformers used for Boltzmann sum**	**27 (cc-pVTZ / B3PW91)**
**Methodology and basis sets for DFT calculations**	**6-31G(d), cc-pVTZ/ B3LYP, B3PW91 / CPCM ($CDCl_3$)**
**Enantiomer used for calculation**	**R**
**Total calculated conformers**	**1340**
**Number of low-energy conformations shown in report**	**4**

Structure of US-16 2:

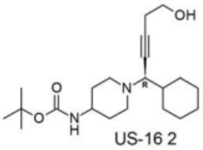

US-16 2

Structure of US-16 1:

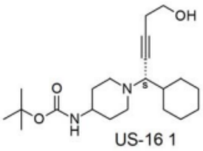

US-16 1

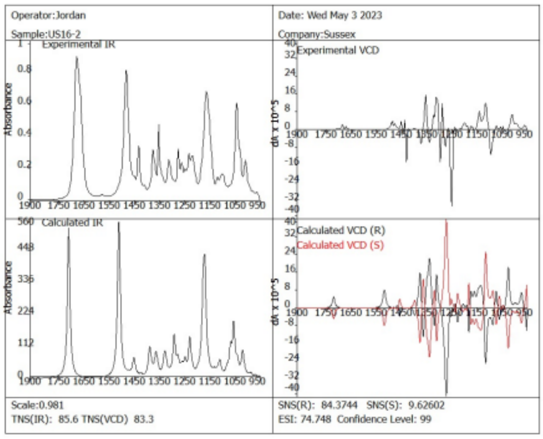

CompareVOA Results.
Please note that the calculated spectra are not scaled in this view.

Figure S30 – VCD analysis of compound **2a**.

Results Summary:
AMPRO-236 (assigned internally as lot number US16);
Incoming purity by achiral SFC: 98.9%
Incoming purity by chiral SFC: 95.5%
Incoming quantity : 2079.5mg

US16_1;
Chemical purity by achiral SFC: 99.8% , m/z = 365.2
Chemical purity by chiral SFC: 99.0% , m/z = 365.2
Enantiomeric excess : 98
Gross vial : 56648.3 mg
Tare vial : 55938.5 mg
Quantity : 709.8 mg

US16_2;
Chemical purity by achiral SFC: 99.9% , m/z = 365.2
Chemical purity by chiral SFC: 99.7% , m/z = 365.2
Enantiomeric excess : 99.4
Gross vial : 56551.8 mg
Tare vial : 55977.6 mg
Quantity : 574.2 mg

Sample Name US16_1 Final

Method QDa_Ethanol NH3 Gradient   Column Name Torus 2-PIC

Date Acquired 12/04/2023 14:52:09 BST

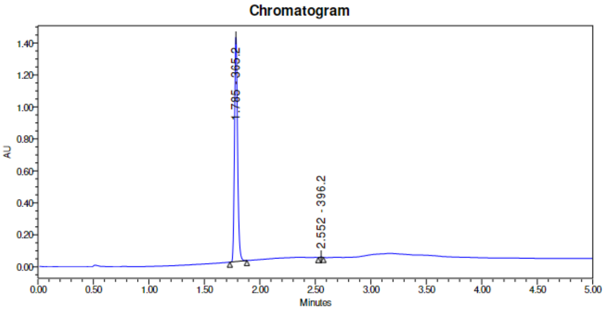

Peak Results

	Retention Time (min)	Area (µV*sec)	% Area	Width @ 50%
1	1.78	2712140	99.8	0.02943
2	2.55	5904	0.2	0.02436

Sample Name US16_2 Final

Method QDa_Ethanol NH3 Gradient   Column Name Torus 2-PIC

Date Acquired 12/04/2023 15:04:11 BST

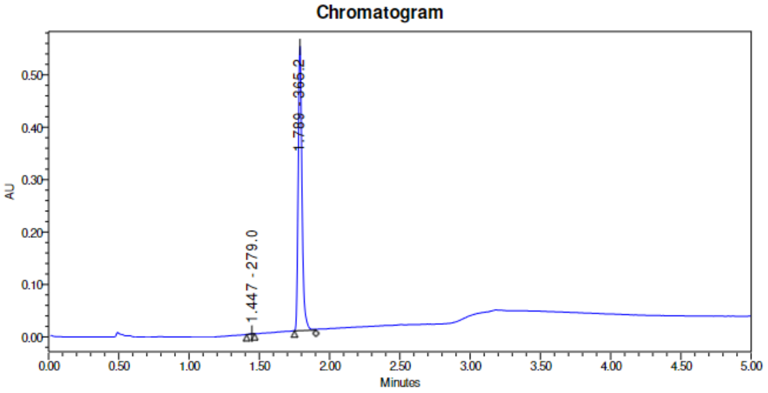

Peak Results

	Retention Time (min)	Area (µV*sec)	% Area	Width @ 50%
1	1.45	1264	0.1	0.03524
2	1.79	1028022	99.9	0.02816

Figure S31 – Chiral separation report of compound **2a**.

2b AMKV-094/AMPRO-238

tert-Butyl (1-(6-chloro-1-cyclohexylhex-2-yn-1-yl)piperidin-4-yl)carbamate.

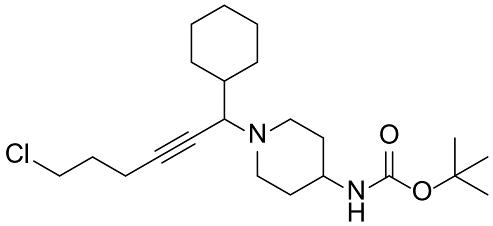

Chemical Formula: $C_{22}H_{37}ClN_2O_2$
Exact Mass: 396.2544
Molecular Weight: 396.9944

Using the general method **2b** synthesised from cyclohexanecarboxaldehyde (2118mg, 2287µL, 18.9mmol, 1.0 eq), 4-N-BOC-aminopiperidine (3781mg, 18.9mmol, 1.0 eq) and 5-chloro-1-pentyne (1936mg, 2000µL, 18.9mmol, 1.0 eq). The resulting crude oil was triturated in hot hexanes to yield pure **2b** as a colourless solid (5015.0mg, 12.0mmol, 67%). ^{1}H NMR (600 MHz, Chloroform-d) δ 4.43 (d, J = 8.0 Hz, 1H), 3.66 (t, J = 6.5 Hz, 2H), 3.44 (s, 1H), 2.87 (dt, J = 11.0, 2.5 Hz, 1H), 2.69 – 2.63 (m, 1H), 2.53 (m, 1H), 2.47 (m, 1H), 2.41 (m, 2H), 2.15 (t, J = 11.0 Hz, 1H), 2.00 – 1.88 (m, 7H), 1.76 – 1.67 (m, 2H), 1.67 – 1.62 (m, 1H), 1.44 (s, 9H), 1.43 – 1.29 (m, 2H), 1.24 -1.22 (m, 1H), 1.19 – 1.08 (m, 2H), 0.88 (m, 2H). ^{13}C NMR (151 MHz, Chloroform-d) δ 155.2, 84.0, 79.2, 78.0, 63.2 (2C), 45.5, 43.8 (2C), 39.7, 33.0, 32.7, 31.8, 31.1, 30.3, 28.4 (3C), 26.7, 26.2, 26.0, 16.1. HRMS $C_{22}H_{37}ClN_2O_2$ Calculated [M+H] = 397.2622 and 399.2592. Experimental [M+H] = 397.2627 and 399.2616 (ppm = +5.8).

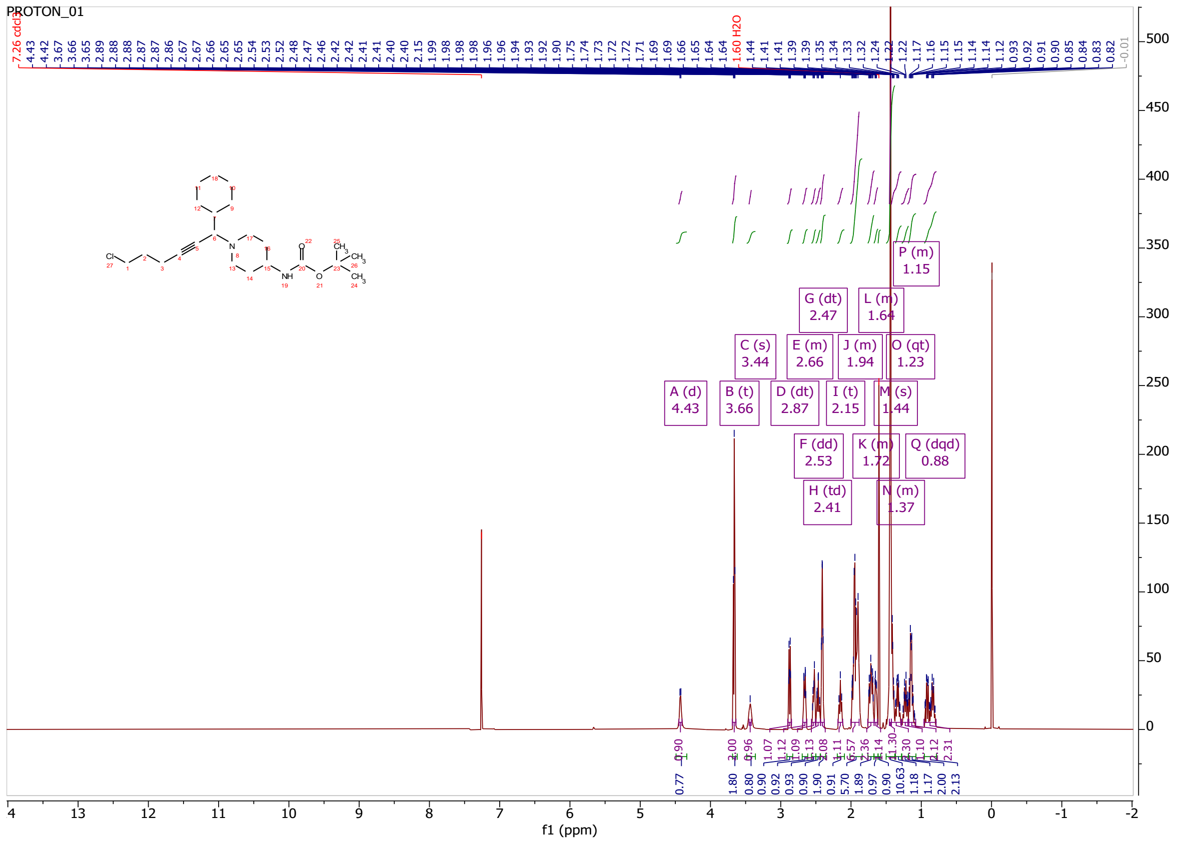

Figure S32 – ^{1}H spectra of compound **2b.**

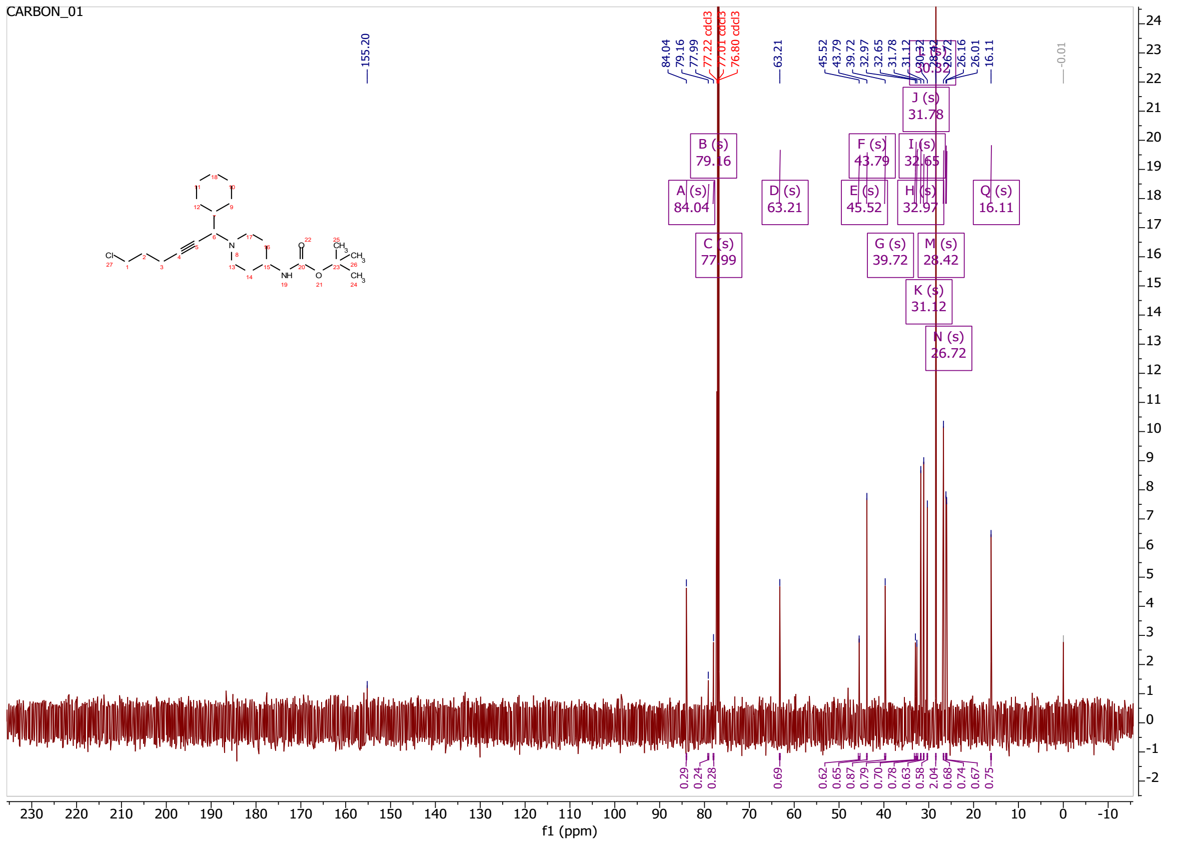

Figure S33 – ^{13}C spectra of compound **2b.**

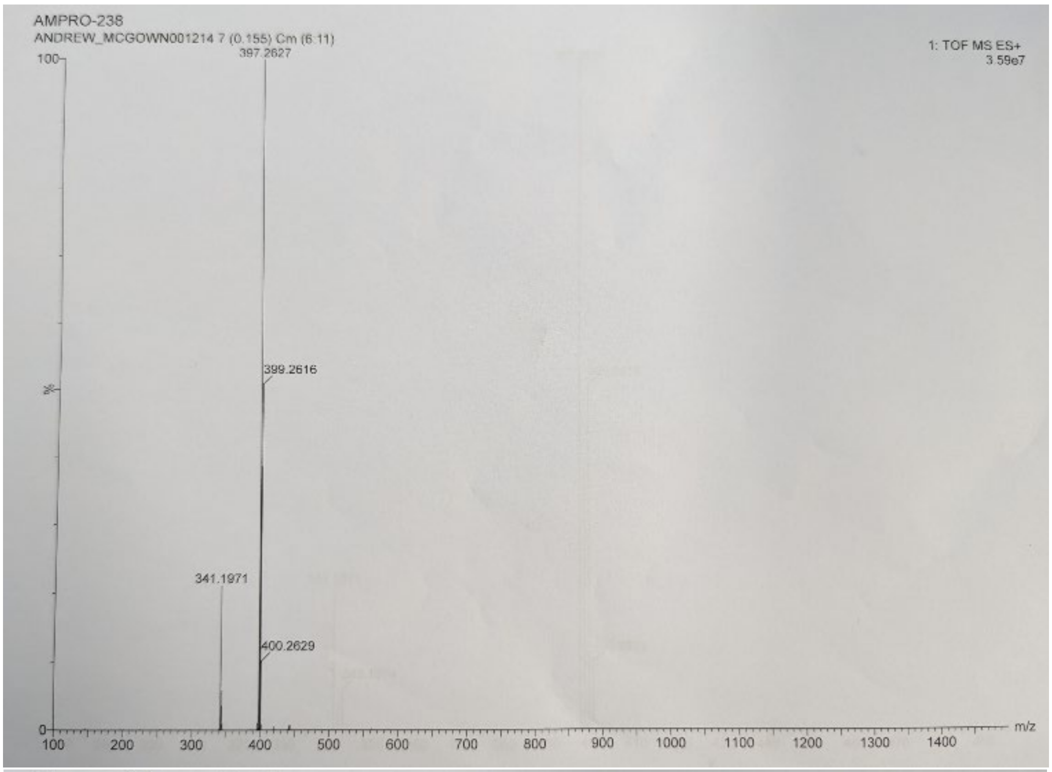

Elemental Composition Report **Page 1**

Single Mass Analysis
Tolerance = 100.0 PPM / DBE: min = -50.0, max = 100.0
Element prediction: Off
Number of isotope peaks used for i-FIT = 3

Monoisotopic Mass, Even Electron Ions
1 formula(e) evaluated with 1 results within limits (up to 50 closest results for each mass)
Elements Used:
C: 22-22 H: 0-100 N: 2-2 O: 2-2 Cl: 1-1
AMPRO-238
ANDREW_MCGOWN001214 7 (0.155) AM2 (Ar,20000.0,0.00,0.00); Cm (6:11)

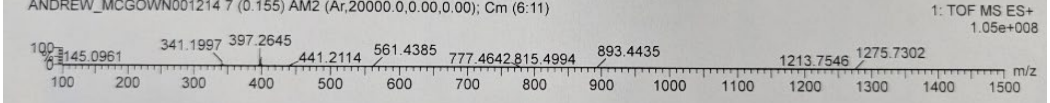

Minimum:				-50.0				
Maximum:		5.0	100.0	100.0				
Mass	Calc. Mass	mDa	PPM	DBE	i-FIT	Norm	Conf(%)	Formula
397.2645	397.2622	2.3	5.8	4.5	935.7	n/a	n/a	C22 H38 N2 O2 Cl

Figure S34 – HRMS analysis of compound **2b.**

VCD Absolute Configuration Determination Report

**GENERAL INFORMATION**	
**Customer**	Sussex
**Sales Order Number**	2023-04 LSV-BTE
**Sample code (BT ref.)**	**US-17 1 / US-17 2**
**Sample description (Customer ref.)**	**US-17 1 / US-17 2**
**VCD-spectrometer**	ChiralIR w/ Dual*PEM*
**Report prepared by: (name / signature as needed)**	Jordan Nafie
**Report validated and signed by**	Rina K Dukor
**Date**	July 13, 2023
**RESULTS**	
Absolute Configuration of US-17 1 is **(R)**<br>Absolute Configuration of US-17 2 is **(S)**	Confidence Level: **100%**
**MEASUREMENT PARAMETERS**	
**Concentration**	11mg / 125uL
**Solvent**	$CDCl_3$
**Instrument Resolution**	4 cm^{-1}
**PEM setting**	1400 cm^{-1}
**Number of scans/Measurement time**	12 hours per enantiomer
**Sample cell**	BaF_2
**Path length**	100 μm
**CALCULATION DETAILS**	
**Molecular Mechanics Force Field**	**MMFF94 (Compute VOA)**
**DFT Software version**	**Gaussian '09**
**Number of conformers used for Boltzmann sum**	**36 (cc-pVTZ / B3PW91)**
**Methodology and basis sets for DFT calculations**	**6-31G(d), cc-pVTZ/ B3LYP, B3PW91 / CPCM ($CDCl_3$)**
**Enantiomer used for calculation**	**R**
**Total calculated conformers**	**2545**
**Number of low-energy conformations shown in report**	**4**

Structure of US-17 1:

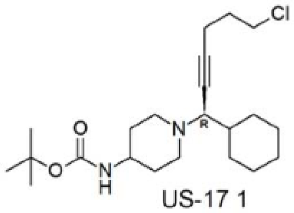

US-17 1

Structure of US-17 2:

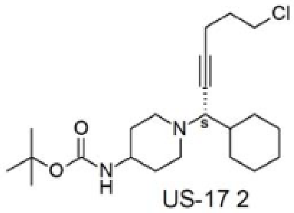

US-17 2

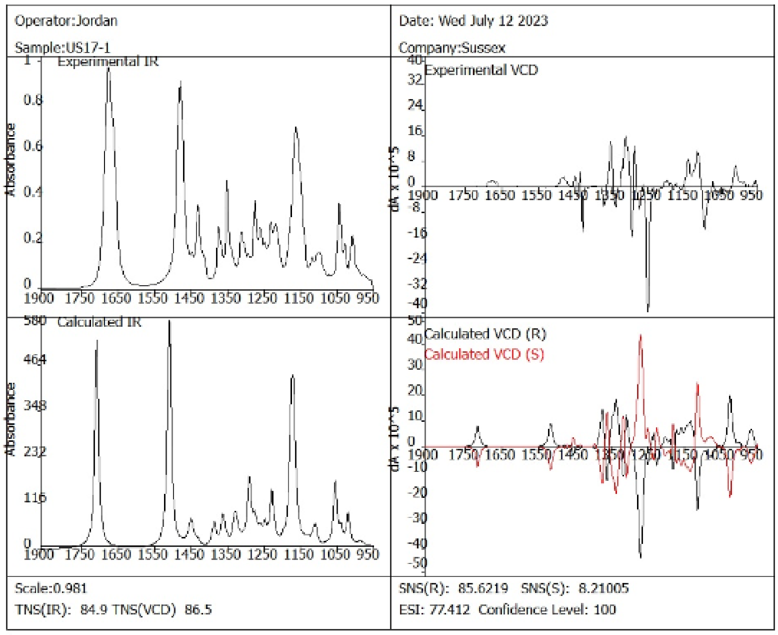

CompareVOA Results.
Please note that the calculated spectra are not scaled in this view.

Figure S35 – VCD analysis of compound **2b.**

Results Summary:
AMPRO-238 (assigned internally as lot number US17);
Incoming purity by achiral SFC : 96.7%
Incoming purity by chiral SFC : 96.0%
Incoming quantity : 1974.5mg

US17_1;
Chemical purity by achiral SFC : 97.7% , m/z = 397.3
Chemical purity by chiral SFC : 96.2%
Enantiomeric excess : 98.8
Gross vial : 57248.1 mg
Tare vial : 56414.7 mg
Quantity : 833.4 mg

US17_2;
Chemical purity by achiral SFC : 99.2% , m/z = 397.3
Chemical purity by chiral SFC: 99.2%
Enantiomeric excess : 97.0
Gross vial : 57260.5 mg
Tare vial : 56408.0 mg
Quantity : 852.5 mg

Sample Name US17_1 Final

Method QDa_Ethanol NH3 Gradient   Column Name Torus Diol

Date Acquired 12/04/2023 14:10:56 BST

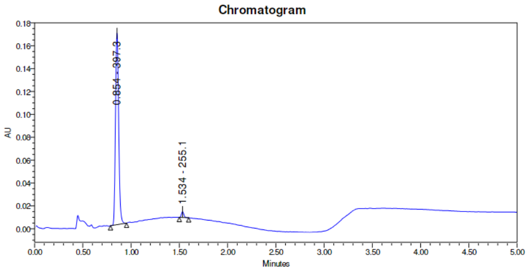

Peak Results

	Retention Time (min)	Area (µV*sec)	% Area	Width @ 50%
1	0.85	383048	97.7	0.03542
2	1.53	9001	2.3	0.02796

Sample Name US17_2 Final

Method QDa_Ethanol NH3 Gradient   Column Name Torus Diol

Date Acquired 12/04/2023 13:49:54 BST

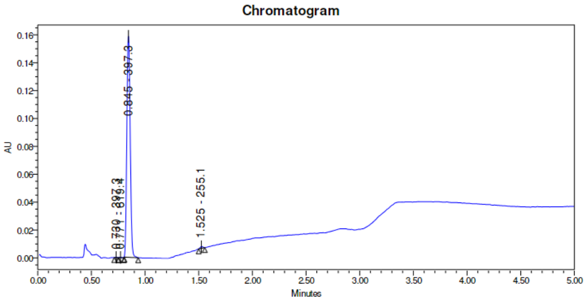

Peak Results

	Retention Time (min)	Area (µV*sec)	% Area	Width @ 50%
1	0.73	260	0.1	0.02200
2	0.77	337	0.1	0.02528
3	0.85	346008	99.2	0.03458
4	1.53	2291	0.7	0.02963

Figure S36 – Chiral separation report of compound **2b.**

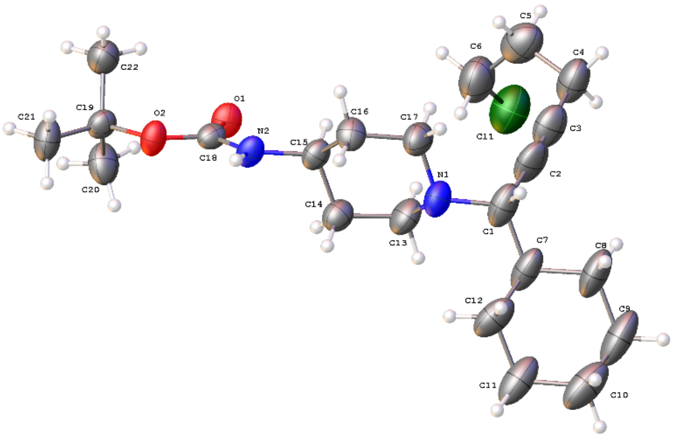

**Reflections:**	d min (CuKα) 2Θ=100.9° 1.00	I/σ(I) 10.1	Rint m=4.50 13.23%	Full 100.9° 99.5
**Refinement:**	Shift 0.000	Max Peak 0.5	Min Peak -0.5	GooF 1.213

Figure S37 – X-ray structural analysis of compound **2b.**

2c AMKV-095

tert-butyl (1-(5-hydroxy-1-(tetrahydro-2H-pyran-4-yl)pent-2-yn-1-yl)piperidin-4-yl)carbamate.

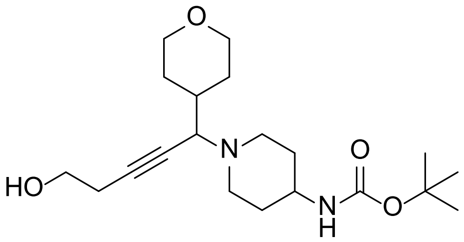

Chemical Formula: $C_{20}H_{34}N_2O_4$
Exact Mass: 366.2519
Molecular Weight: 366.4950

Using the general method **2c** was synthesised from tetrahydro-2H-pyran-4-carboxaldehyde (1508mg, 1376µL, 13.2mmol, 1.0 eq), 4-(N-Boc-amino)piperidine (2646mg, 13.2mmol, 1.0 eq) and 3-butyn-1-ol (926mg, 1000µL, 13.2mmol, 1.0 eq). The resulting crude oil was triturated with petroleum ether leading to the isolation of **2c** (3194.8mg, 8.3mmol, 63%) as a colourless solid. ^{1}H NMR (600 MHz, Chloroform-d) δ 4.59 (d, J = 8.0 Hz, 1H), 3.97 – 3.83 (m, 2H), 3.64 (t, J = 7.0 Hz, 2H), 3.37 (s, 1H), 3.29 (dt, J = 12.0 Hz, 2H), 2.89 (d, J = 10.0 Hz, 1H), 2.61 (d, J = 12.0 Hz, 2H), 2.50 (d, J = 11.0 Hz, 1H), 2.45 - 2.41 (m, 4H), 2.15 (t, J = 11.0 Hz, 1H), 1.87 - 1.81 (m, 3H), 1.75 (d, J = 14.0 Hz, 1H), 1.66 - 1.62 (m, 1H), 1.38 (s, 9H), 1.31 – 1.10 (m, 3H). ^{13}C NMR (151 MHz, Chloroform-d) δ 155.2, 83.2, 79.2, 77.8, 67.9, 67.8, 62.9, 61.4, 47.8, 45.4 (2C), 37.2, 32.8, 32.5, 31.1, 30.6, 28.4 (3C), 23.0. HRMS $C_{20}H_{34}N_2O_4$ Calculated [M+H] = 367.2597. Experimental [M+H] = 367.2540 (PPM = +11.7).

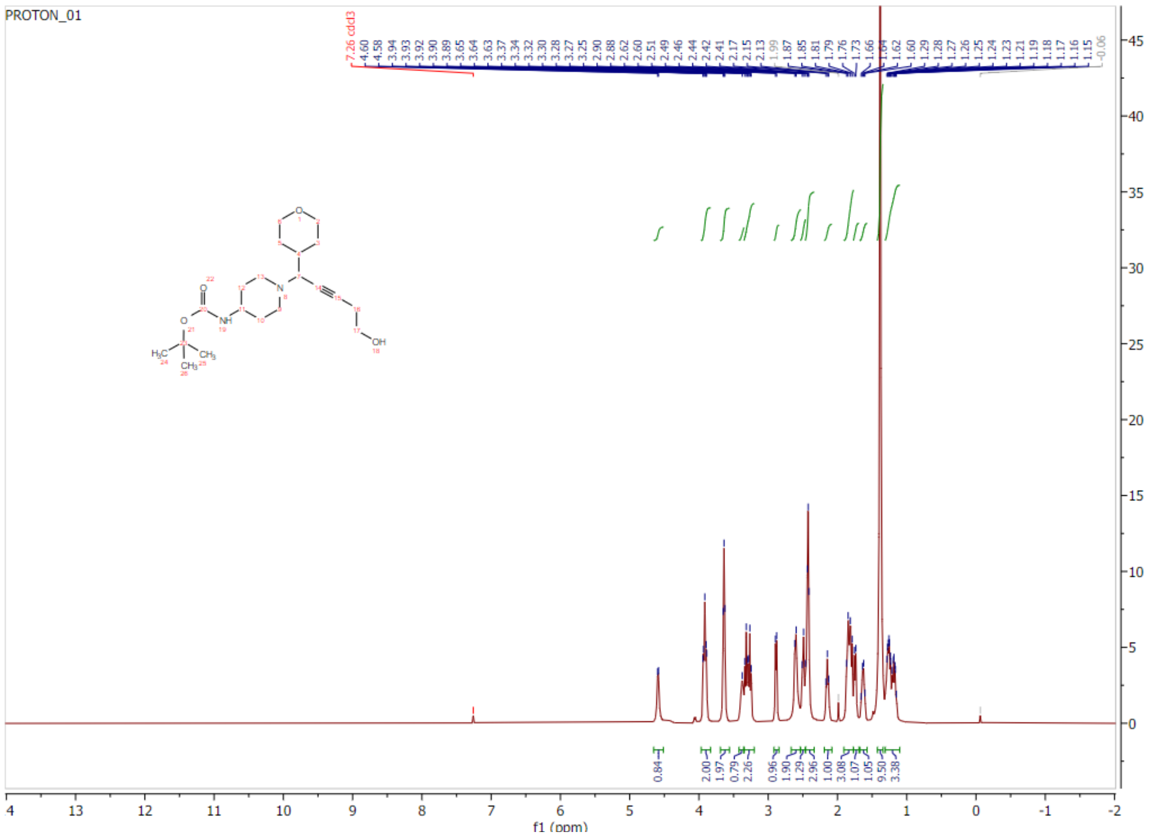

Figure S38 – ^{1}H spectra of compound **2c.**

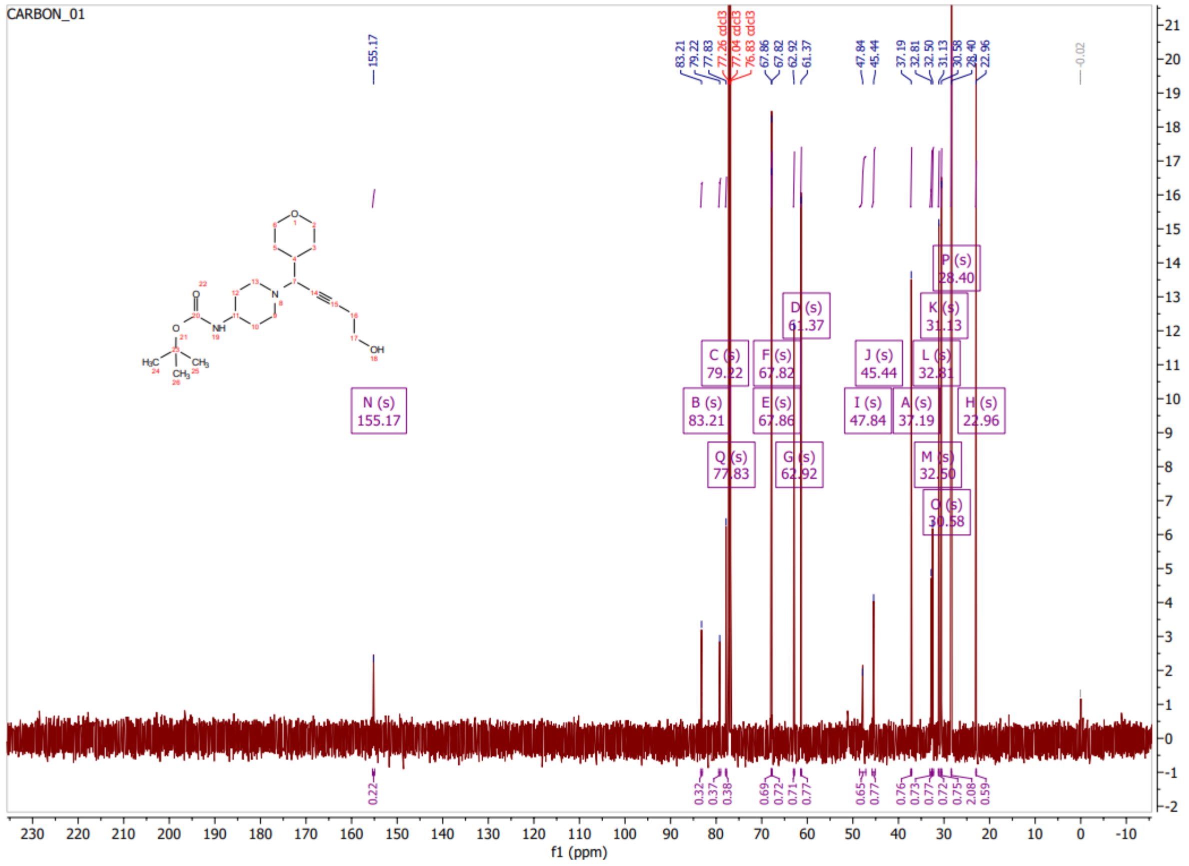

Figure S39 – ^{13}C spectra of compound **2c.**

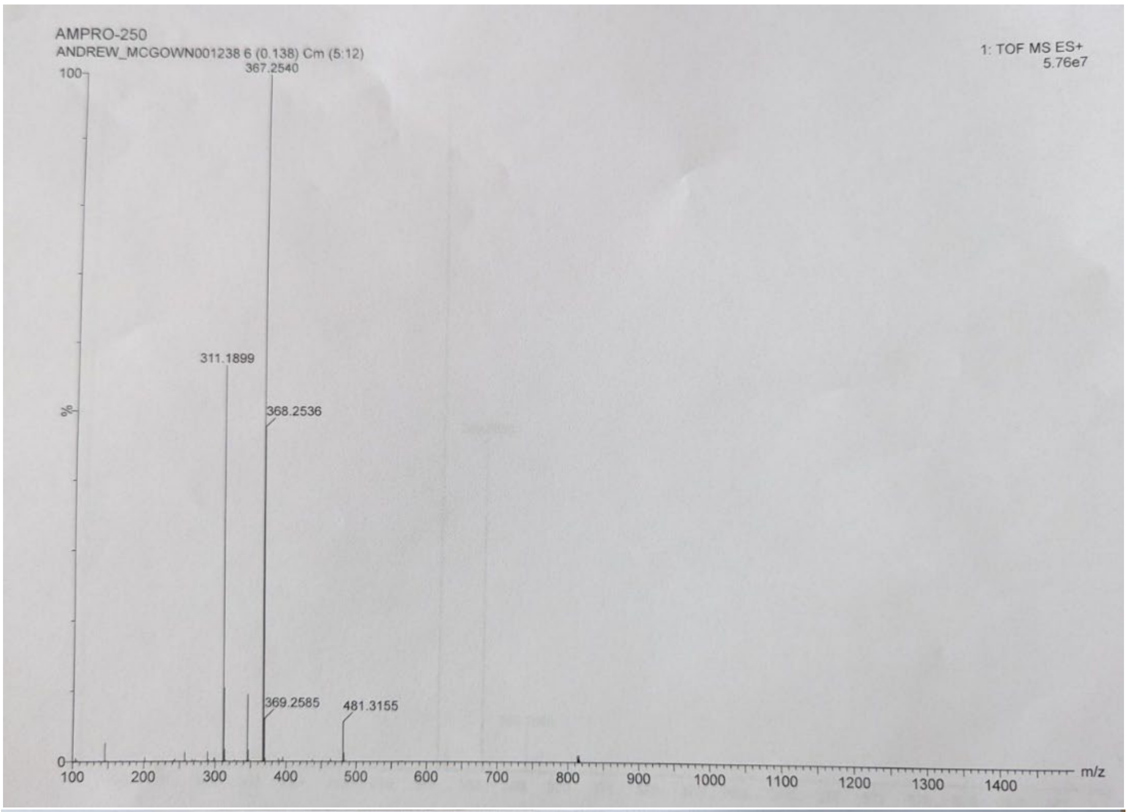

Elemental Composition Report **Page 1**

Single Mass Analysis
Tolerance = 100.0 PPM / DBE: min = -150.0, max = 250.0
Element prediction: Off
Number of isotope peaks used for i-FIT = 3

Monoisotopic Mass, Even Electron Ions
1 formula(e) evaluated with 1 results within limits (up to 50 closest results for each mass)
Elements Used:
C: 20-20 H: 0-100 N: 2-2 O: 4-4
AMPRO-250
ANDREW_MCGOWN001238 6 (0.138) AM2 (Ar,20000.0,0.00,0.00); Cm (6:12) 1: TOF MS ES+ 1.54e+008

345.2309 347.2359 364.2693 367.2554 369.2573 381.2656 389.2322 395.2810 397.2855 407.2446 411.2858 m/z
340.0 350.0 360.0 370.0 380.0 390.0 400.0 410.0

Minimum:				-150.0				
Maximum:		5.0	100.0	250.0				
Mass	Calc. Mass	mDa	PPM	DBE	i-FIT	Norm	Conf(%)	Formula
367.2554	367.2597	-4.3	-11.7	4.5	929.7	n/a	n/a	C20 H35 N2 O4

Figure S40 – HRMS analysis of compound **2c**.

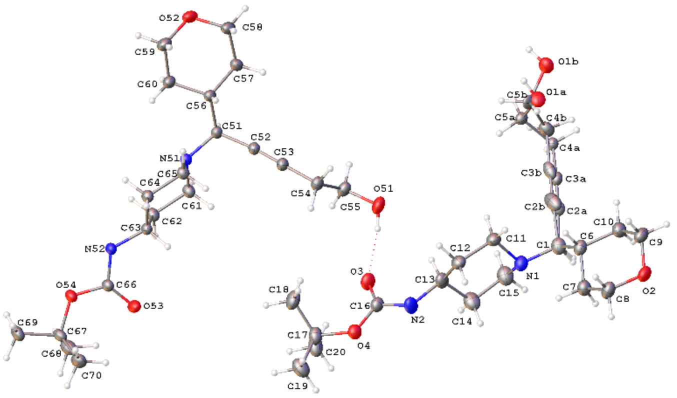

**Reflections:**	d min (MoKα) 2Θ=76.1° 0.58	I/σ(I) 43.1	Rint m=4.73 2.37%	Full 50.5° 94% to 76.1° 100
**Refinement:**	Shift -0.001	Max Peak 1.4	Min Peak -0.7	GooF 1.038

Figure S41 – X-ray structural analysis of compound **2c**.

2d AMKV-097/AMPRO-251

tert-butyl (1-(5-bromo-1-(tetrahydro-2H-pyran-4-yl)pent-2-yn-1-yl)piperidin-4-yl)carbamate.

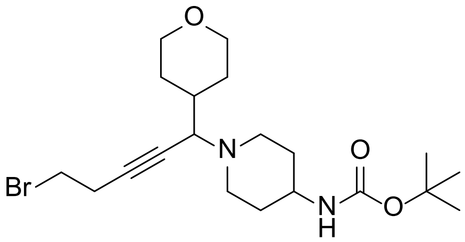

Chemical Formula: $C_{20}H_{33}BrN_2O_3$
Exact Mass: 428.1675
Molecular Weight: 429.3916

Using the general method **2d** was synthesised from tetrahydro-2H-pyran-4-carboxaldehyde (1216mg, 1110µL, 10.7mmol, 1.0 eq), 4-(N-Boc-amino)piperidine (2134mg, 10.7mmol, 1.0 eq) and 4-bromo-1-butyne (1417mg, 1000µL, 10.7mmol, 1.0 eq). The crude oil was triturated in petroleum spirit and **2d** was isolated as a colourless solid (1121.6mg, 2.5mmol, 23%). ^{1}H NMR (600 MHz, Chloroform-d) δ 4.47 (s, 1H), 4.02 – 3.93 (m, 2H), 3.49 (s, 1H), 3.43 (t, J = 7.0 Hz, 2H), 3.40 - 3.35 (m, 1H), 3.34 - 3.30 (m, 1H), 2.94 (d, J = 10.0 Hz, 1H), 2.78 (m, 2H), 2.67 - 2.64 (m, 1H), 2.56 – 2.49 (m, 2H), 2.24 (t, J = 10.0 Hz, 1H), 1.95 – 1.84 (m, 3H), 1.81 (dd, J = 13.5, 3.5 Hz, 1H), 1.70 -1.68 (m, 1H), 1.61 (s, 1H), 1.44 (s, 9H), 1.35 – 1.17 (m, 3H). ^{13}C NMR (151 MHz, Chloroform-d) δ 155.2, 83.4, 79.2, 78.3, 67.9, 67.8, 62.9, 47.7, 45.5 (2C), 37.1, 32.8, 32.5, 31.2, 30.6, 30.5, 28.4 (3C), 23.2. HRMS $C_{20}H_{33}BrN_2O_3$ Calculated [M+H] = 429.1753 and 431.1732. Experimental [M+H] = 429.1735 and 431.1724 (ppm = -2.3) Br isotopes observed.

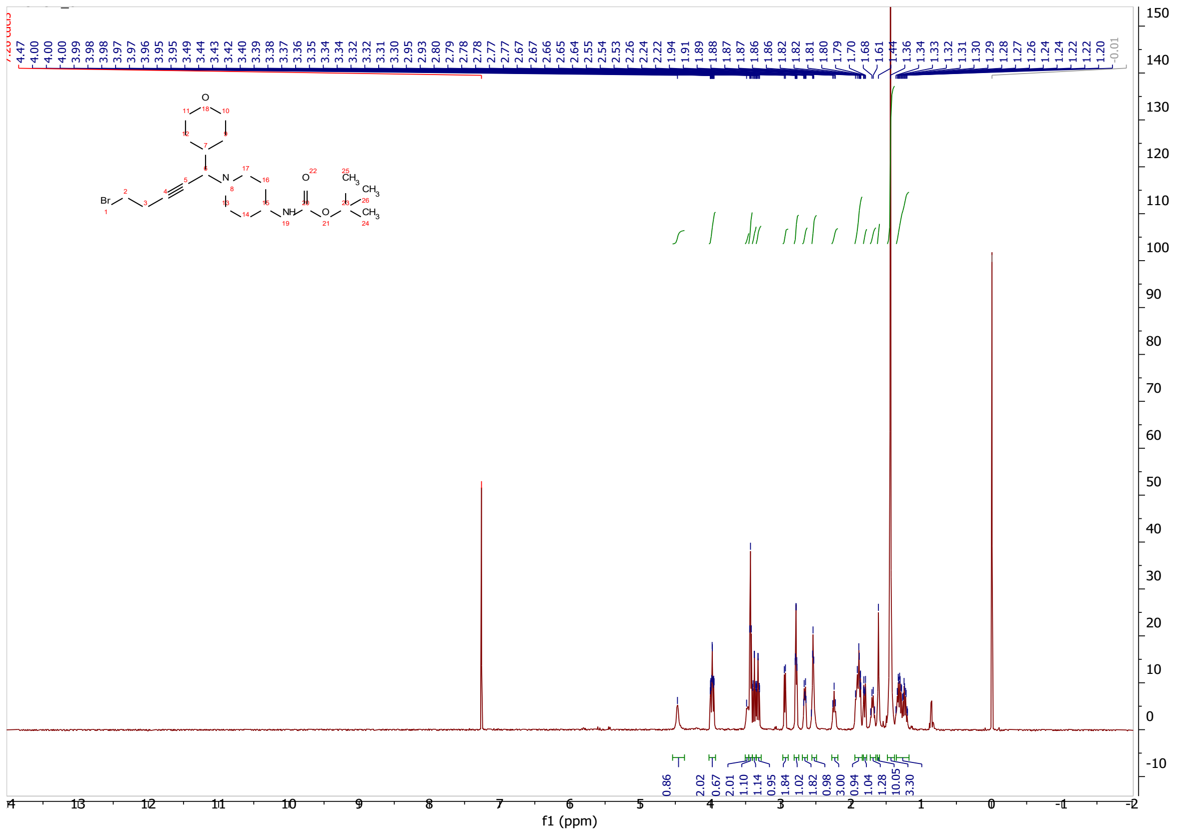

Figure S42 – ^{1}H spectra of compound **2d.**

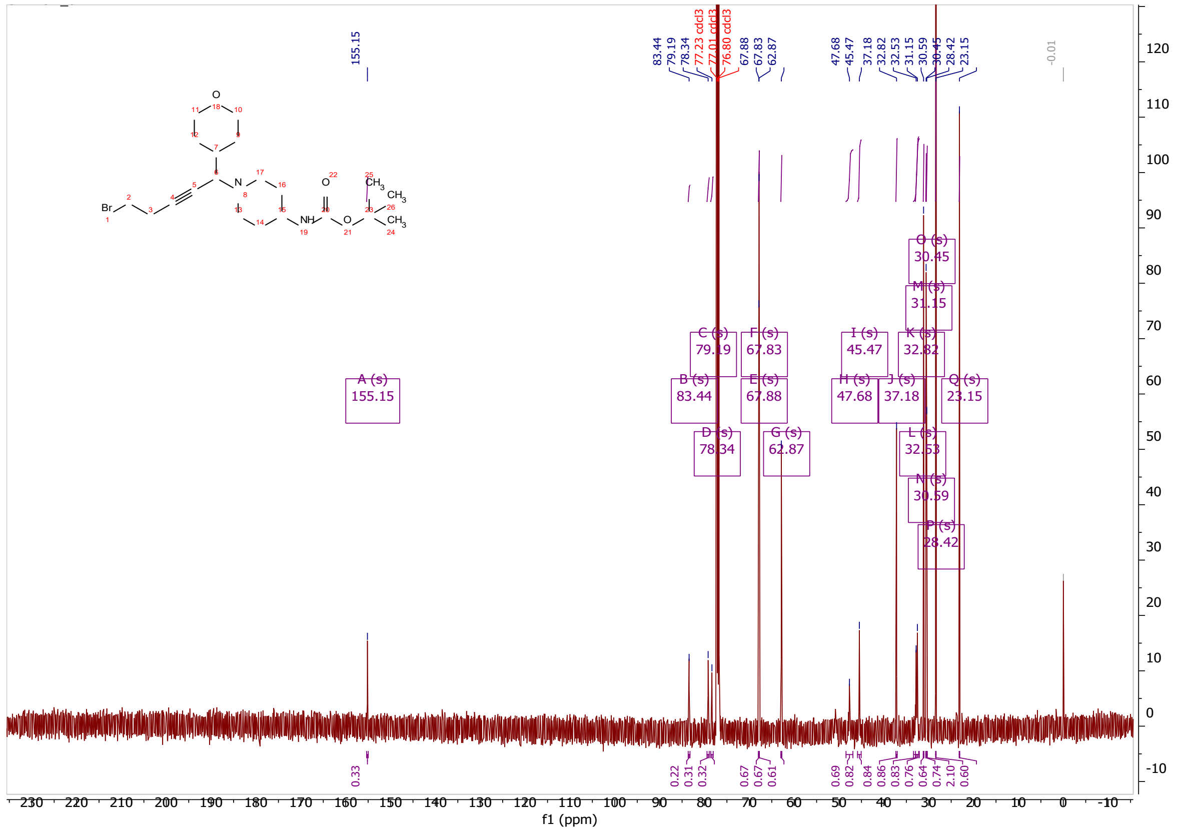

Figure S43 – ^{13}C spectra of compound **2d.**

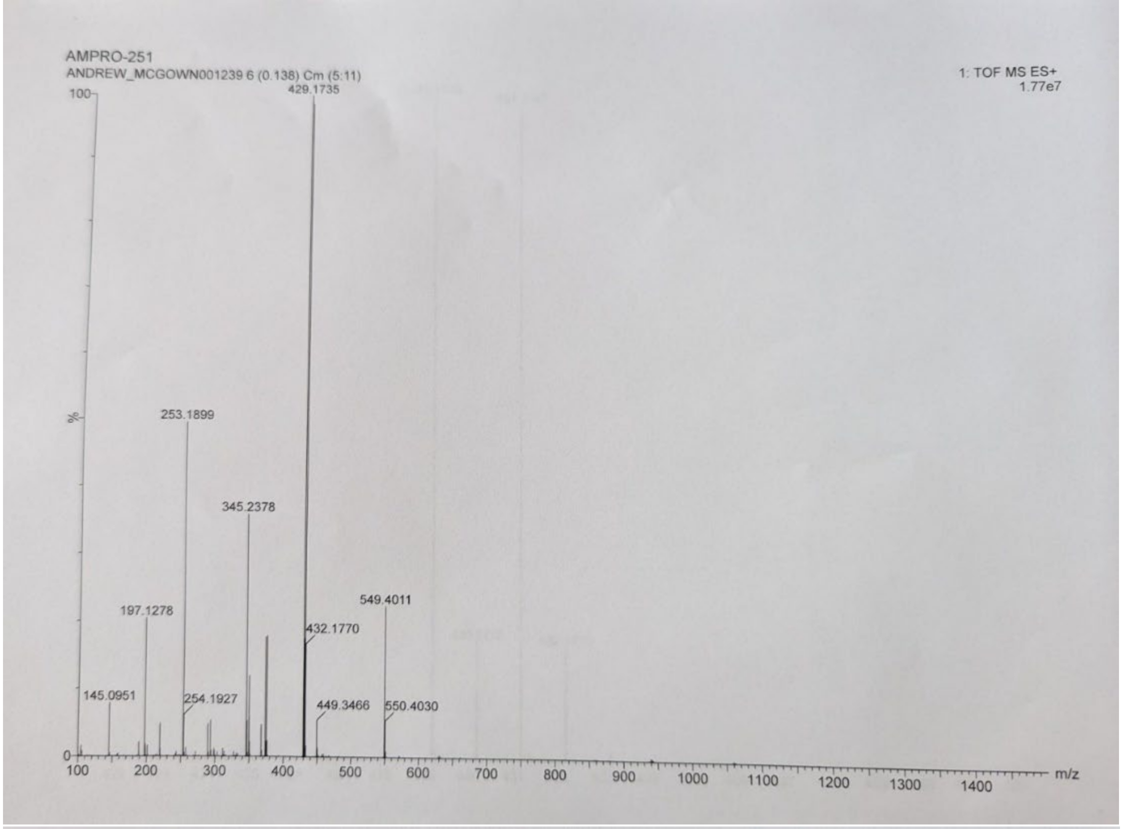

Elemental Composition Report

Page 1

Single Mass Analysis

Tolerance = 10.0 PPM / DBE: min = -1.5, max = 50.0
Element prediction: Off
Number of isotope peaks used for i-FIT = 3

Monoisotopic Mass, Even Electron Ions
5 formula(e) evaluated with 1 results within limits (up to 50 closest results for each mass)
Elements Used:
C: 20-20 H: 0-1000 N: 2-2 O: 3-3 Br: 0-8

AMPRO-251
ANDREW_MCGOWN001239 6 (0.138) AM2 (Ar,20000.0,0.00,0.00); Cm (5:11)

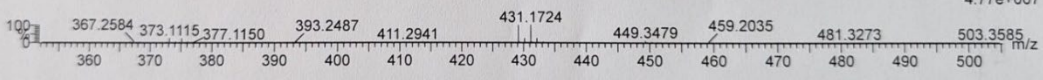

Minimum:				-1.5				
Maximum:		5.0	10.0	50.0				
Mass	Calc. Mass	mDa	PPM	DBE	i-FIT	Norm	Conf(%)	Formula
429.1743	429.1753	-1.0	-2.3	4.5	839.1	n/a	n/a	C20 H34 N2 O3 Br

Figure S44 – HRMS analysis of compound **2d.**

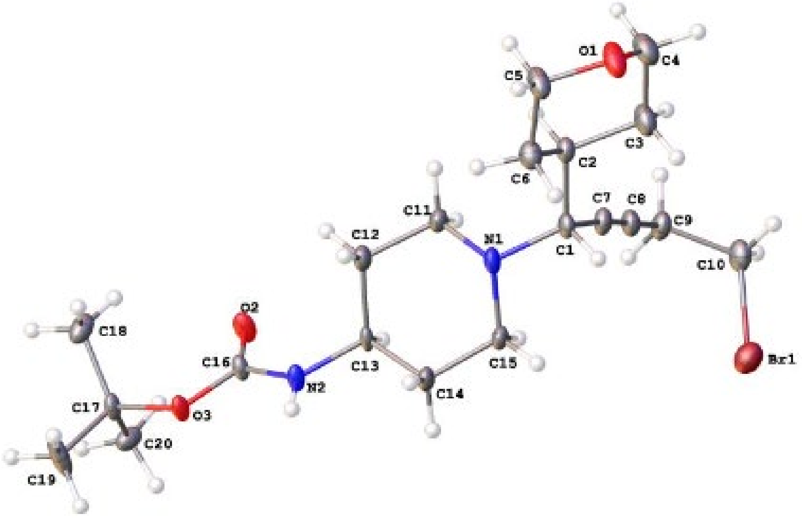

Structure Quality Indicators

**Reflections:**	d min (Cu\a) 2Θ=154.9° 0.79	I/σ(I) 15.6	Rint m=5.04 10.56%	Full 135.4° 93% to 154.9° 99.9
**Refinement:**	Shift -0.000	Max Peak 1.1	Min Peak -1.5	GooF 1.023

Figure S45 – X-ray structural analysis of compound **2d**.

2e AMKV-098/AMPRO-252

tert-butyl (1-(6-chloro-1-(tetrahydro-2H-pyran-4-yl)hex-2-yn-1-yl)piperidin-4-yl)carbamate.

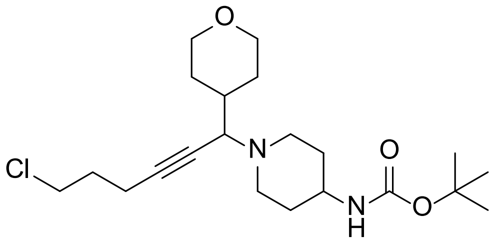

Chemical Formula: $C_{21}H_{35}ClN_2O_3$
Exact Mass: 398.2336
Molecular Weight: 398.9672

Using the general method **2e** was synthesised from tetrahydro-2H-pyran-4-carboxaldehyde (1078mg, 983µL, 9.4mmol, 1.0 eq), 4-(N-Boc-amino)piperidine (1891mg, 9.4mmol, 1.0 eq) and 5-chloro-1-pentyne (968mg, 1000µL, 9.4mmol, 1.0 eq). The crude oil was triturated in petroleum spirit to yield **2e** (1243mg, 3.0mmol, 33%) as a colourless solid. ^{1}H NMR (600 MHz, Chloroform-d) δ 4.44 (d, J = 8. Hz, 1H), 4.02 – 3.93 (m, 2H), 3.65 (t, J = 6.0 Hz, 2H), 3.44 (s, 1H), 3.37 (m, 1H), 3.32 (m, 1H), 2.93 (m, 1H), 2.64 (m, 1H), 2.57 – 2.45 (m, 2H), 2.42 -2.42 (m, 2H), 2.19 (t, J = 11.0 Hz, 1H), 1.98 – 1.87 (m, 4H), 1.86 -1.78 (m, 2H), 1.70 – 1.64 (m, 1H), 1.44 (s, 9H) 1.35 – 1.17 (m, 3H). ^{13}C NMR (151 MHz, Chloroform-d) δ 155.2, 84.7, 79.2, 67.9, 67.9, 62.9, 47.9, 45.4, 43.7 (2C), 37.3, 32.9, 32.6, 31.7, 31.2, 30.6, 28.4 (3C), 16.1. HRMS $C_{21}H_{35}ClN_2O_3$ Calculated [M+H] = 399.2414. Experimental m/z = 399.2452 (PPM = -86.7).

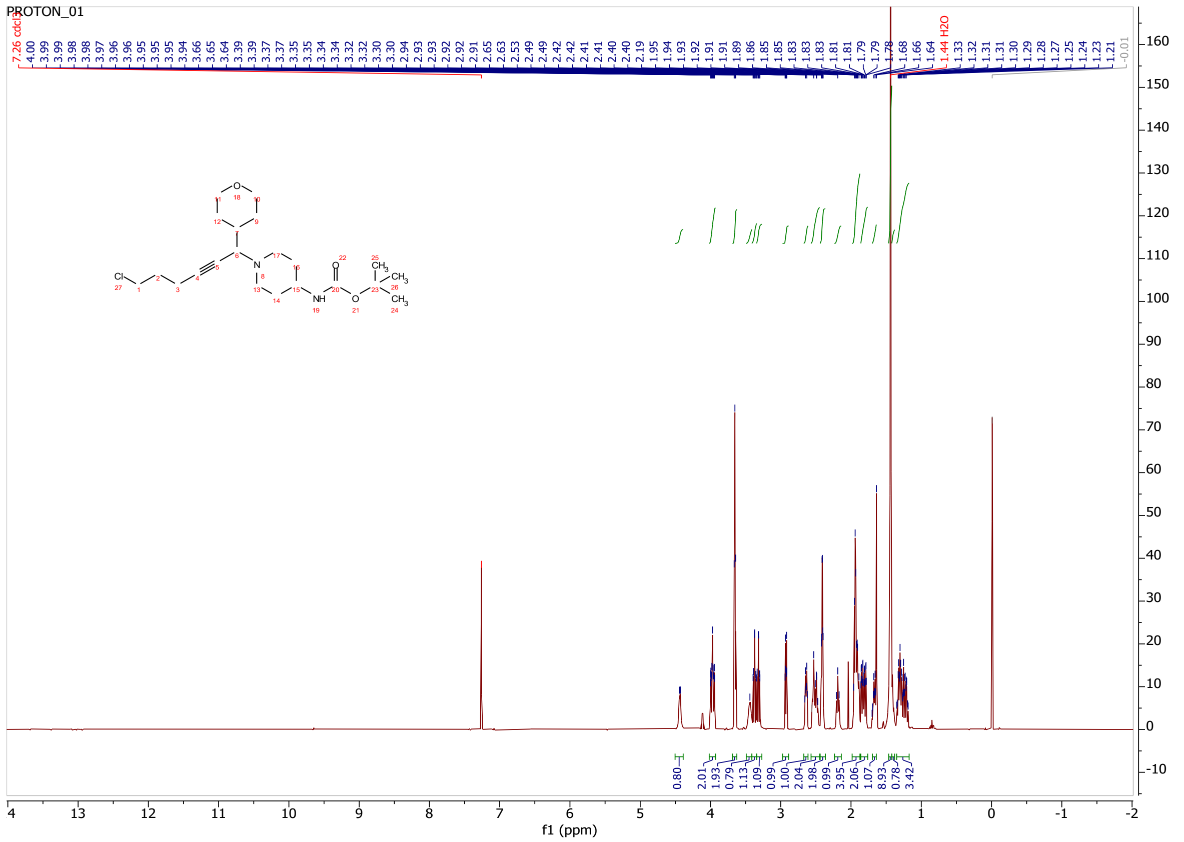

Figure S46 – ^{1}H spectra of compound **2e**.

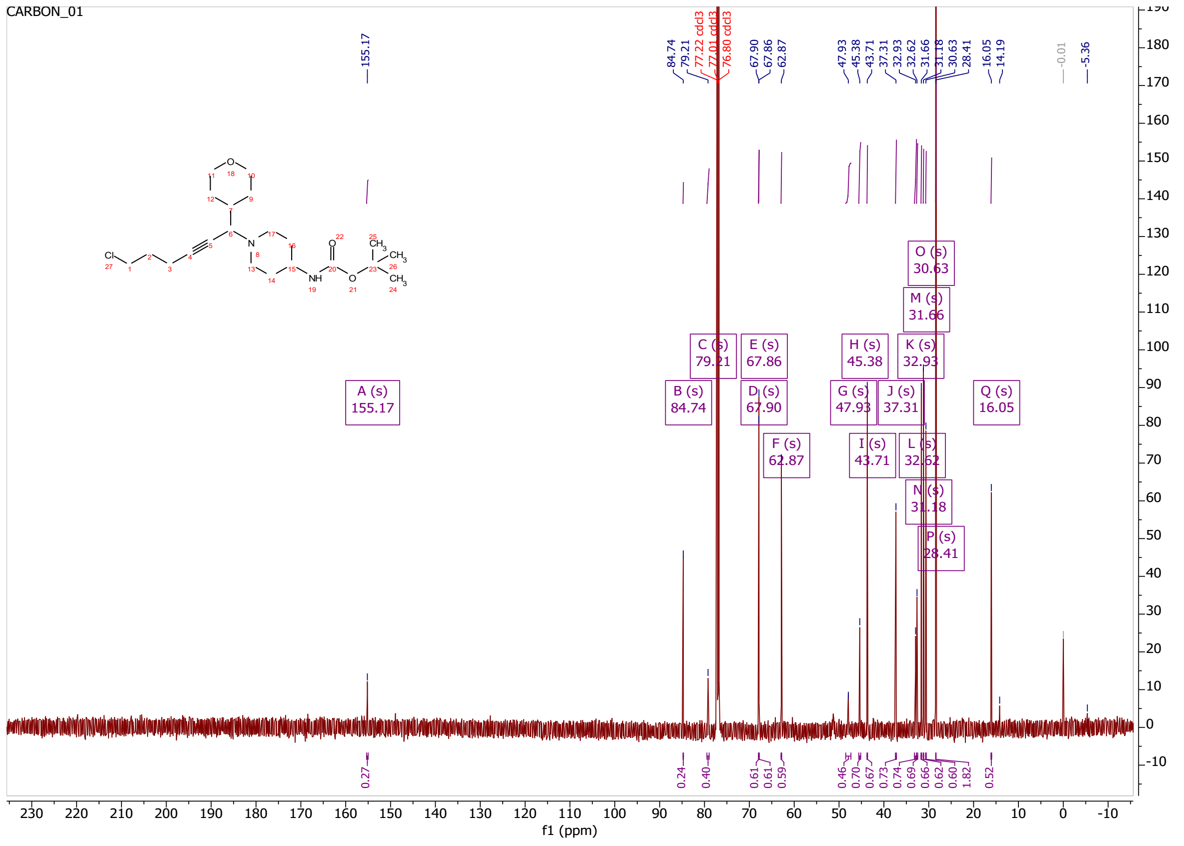

Figure S47 – ^{13}C spectra of compound **2e**.

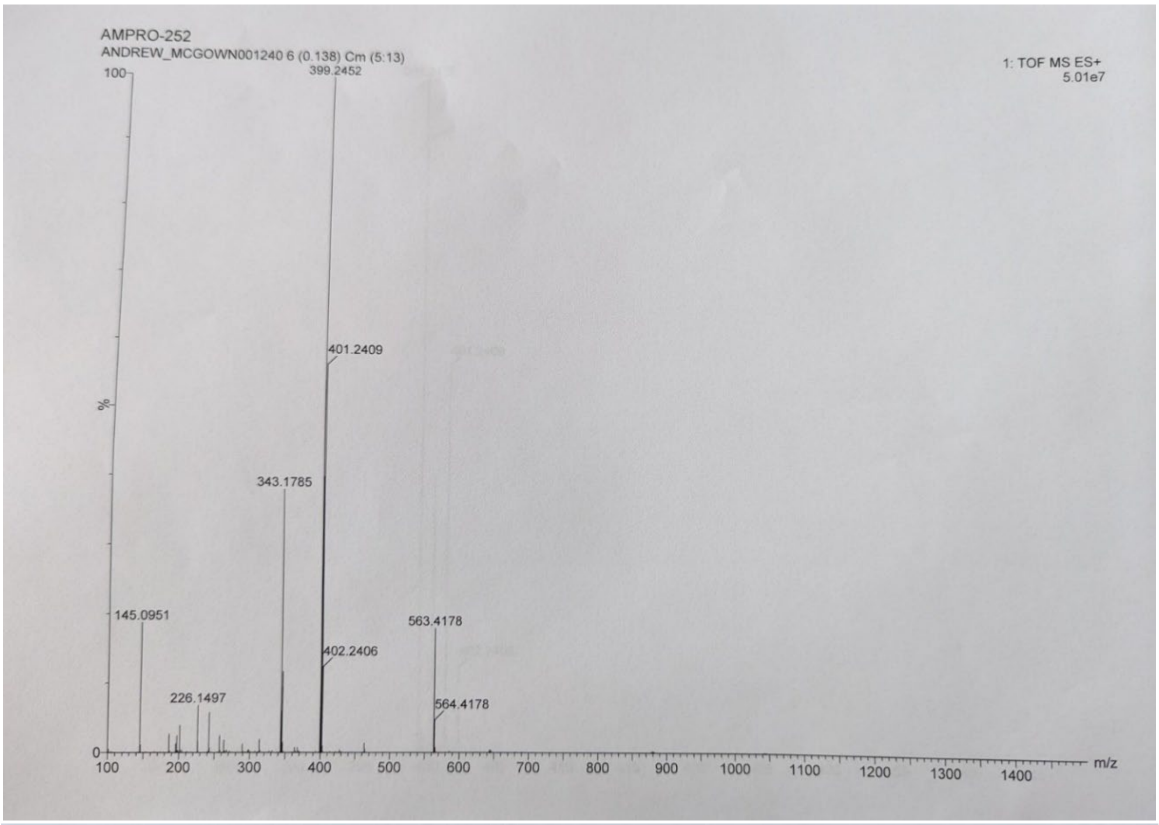

Elemental Composition Report | **Page 1**

Single Mass Analysis
Tolerance = 100.0 PPM / DBE: min = -150.0, max = 250.0
Element prediction: Off
Number of isotope peaks used for i-FIT = 3

Monoisotopic Mass, Even Electron Ions
1 formula(e) evaluated with 1 results within limits (up to 50 closest results for each mass)
Elements Used:
C: 22-22 H: 0-100 N: 2-2 O: 2-2 Cl: 1-1
AMPRO-252
ANDREW_MCGOWN001240 6 (0.138) AM2 (Ar,20000.0,0.00,0.00); Cm (6:12)
1: TOF MS ES+
1.28e+008

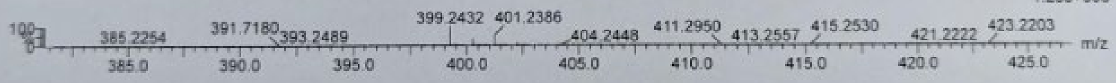

Minimum:				-150.0				
Maximum:		5.0	100.0	250.0				
Mass	Calc. Mass	mDa	PPM	DBE	i-FIT	Norm	Conf(%)	Formula
399.2432	399.2778	-34.6	-86.7	3.5	982.3	n/a	n/a	C22 H40 N2 O2 Cl

Figure S48 – HRMS analysis of compound **2e**.

2f AMKV-099/AMPRO-291

tert-butyl (1-(6-chloro-1-(tetrahydro-2H-pyran-4-yl)hex-2-yn-1-yl)piperidin-4-yl)carbamate.

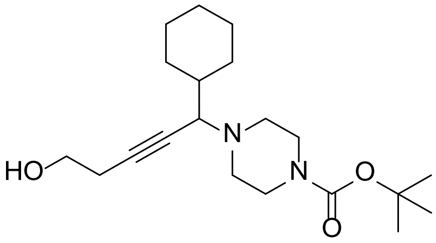

Chemical Formula: $C_{20}H_{34}N_2O_3$
Exact Mass: 350.2569
Molecular Weight: 350.4956

Using the general method **2f** was synthesised from cyclohexanecarboxaldehyde (744mg, 803μL, 6.6mmol, 1.0 eq), 1-BOC-piperazine (1236mg, 6.6mmol, 1.0 eq), and 3-butyn-1-ol (465mg, 500μL, 6.6mmol, 1.0 eq). **2f** was isolated as a pale yellow, viscous oil (1432mg, 4.0mmol, 58%). ^{1}H NMR (600 MHz, Chloroform-d) δ 3.69 (t, J = 6.0 Hz, 2H), 3.42 (s, 1H), 3.39 (s, 2H), 2.92 (dt, J = 10.0, 2.0 Hz, 1H), 2.54 – 2.50 (m, 2H), 2.49 (td, J = 6.0, 2.0 Hz, 2H), 2.31 (s, 1H), 2.00 – 1.93 (m, 3H), 1.77 – 1.68 (m, 2H), 1.65 (d, J = 9.0 Hz, 1H), 1.45 (s, 9H), 1.23 – 1.19 (m, 2H), 1.18 - 1.12 (m, 2H), 0.99 – 0.89 (m, 2H), 0.89 – 0.82 (m, 2H). *Some residual EtOAc*. ^{13}C NMR (151 MHz, Chloroform-d) δ 154.9, 82.8, 79.5, 78.6, 63.3 (2C), 61.4 (2C), 39.4, 31.0, 30.3, 28.4 (3C), 26.7 (2C), 26.1, 25.9, 23.1 (2C). HRMS $C_{20}H_{34}N_2O_3$ Calculated [M+H] = 351.2648. Experimental [M+H] = 351.2687 (+0.3ppm).

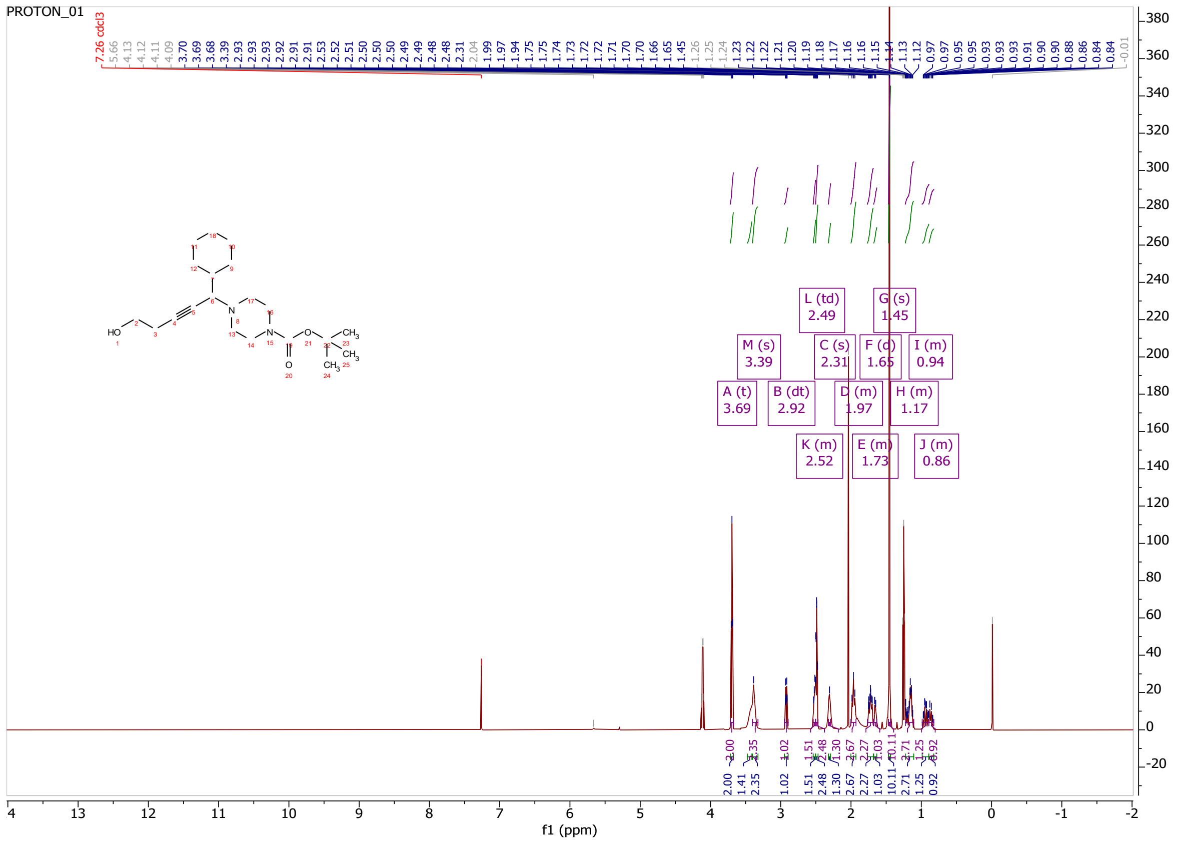

Figure S49 – ^{1}H spectra of compound **2f**.

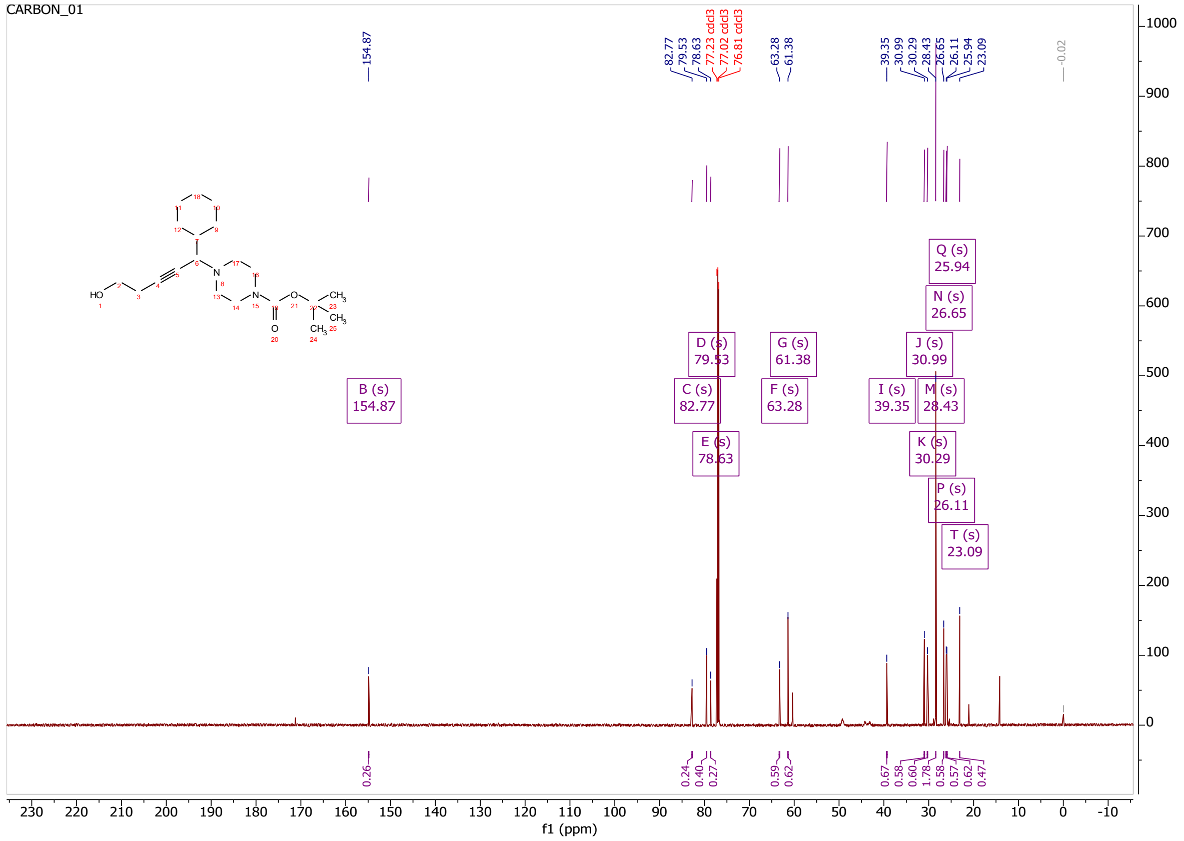

Figure S50 – ^{13}C spectra of compound **2f**.

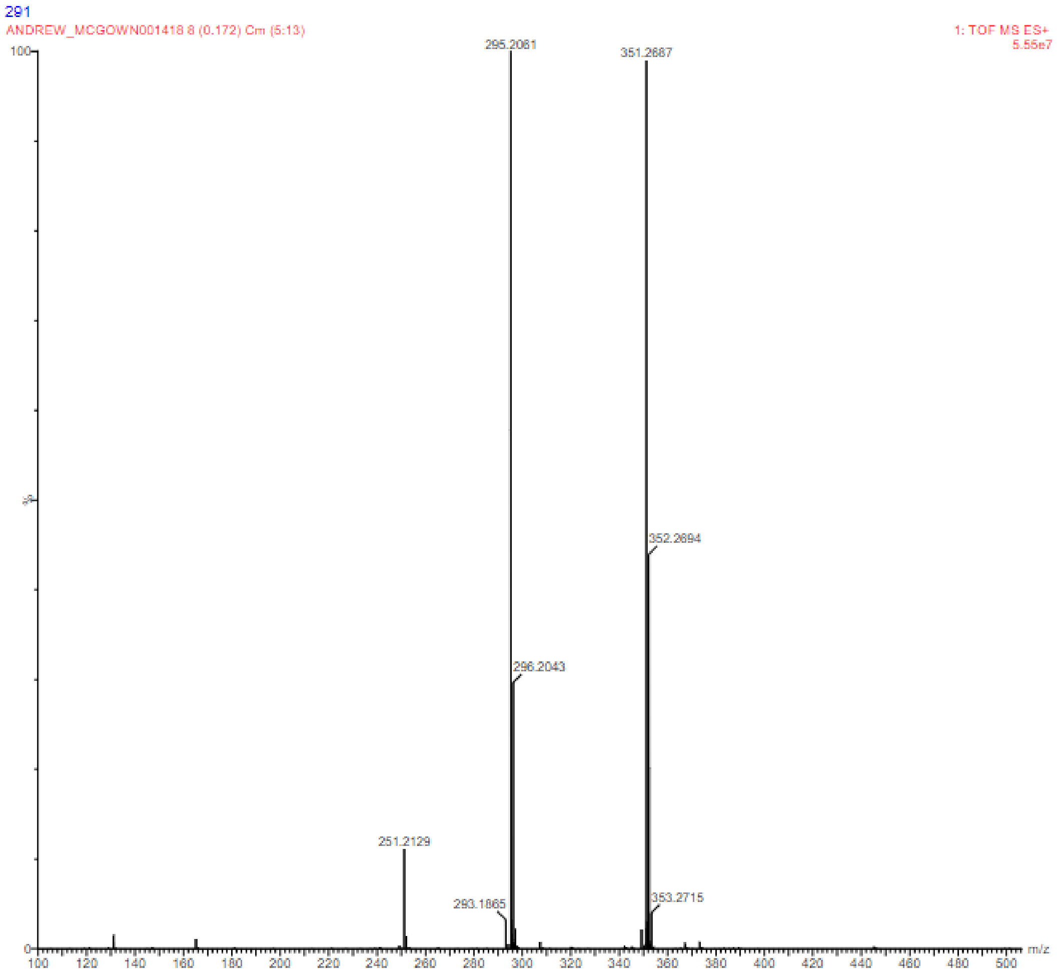

C: 20-20 H: 0-100 N: 2-2 O: 3-3

Minimum:			-1.5		
Maximum:	5.0	100.0	50.0		
Mass	Calc. Mass	mDa	PPM	DBE	Formula
351.2649	351.2648	0.1	0.3	4.5	C20 H35 N2 O3

Figure S51 – HRMS analysis of compound **2f**.

2g AMKV-101/AMPRO-293

tert-butyl 4-(5-bromo-1-cyclohexylpent-2-yn-1-yl)piperazine-1-carboxylate.

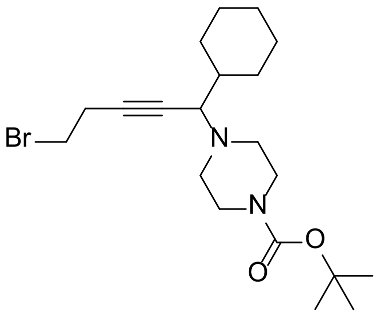

Chemical Formula: $C_{20}H_{33}BrN_2O_2$
Exact Mass: 412.1725
Molecular Weight: 413.3922

Using the general method **2g** was synthesised from cyclohexanecarboxaldehyde (298mg, 322µL, 2.7mmol, 1.0 eq), 1-BOC-piperazine (496mg, 2.7mmol, 1.0 eq), 4-bromo-1-butyne (354mg, 250µL, 2.7mmol, 1.0 eq). **2g** was isolated as a pale yellow, viscous oil (898.7mg, 2.17mmol, 78%). ^{1}H NMR (600 MHz, Chloroform-d) δ 3.43 (t, J = 7.0 Hz, 2H), 3.40 (s, 3H), 2.92 (d, J = 10.0 Hz, 1H), 2.77 (td, J = 7.0, 2.0 Hz, 2H), 2.54 - 2.52 (m, 2H), 2.34 - 2.32 (m, 2H), 1.99 – 1.95 (m, 2H), 1.75 – 1.70 (m, 3H), 1.66 - 1.64 (m, 1H), 1.46 (s, 9H), 1.25 - 1.19 (m, 2H), 1.18 - 1.12 (m, 2H), 0.99 – 0.88 (m, 2H). ^{13}C NMR (151 MHz, Chloroform-d) δ 154.8, 83.2, 79.5, 78.9, 63.3 (2C), 44.2, 39.3 (2C), 30.9, 30.2, 28.4 (3C), 26.7 (2C), 26.1, 26.0, 23.2 (2C). HRMS $C_{20}H_{33}BrN_2O_2$ Calculated [M+H] = 413.1804 and 415.1783. Experimental [M+H] = 413.1816 and 415.1798 (ppm = +2.9) Br isotopes observed.

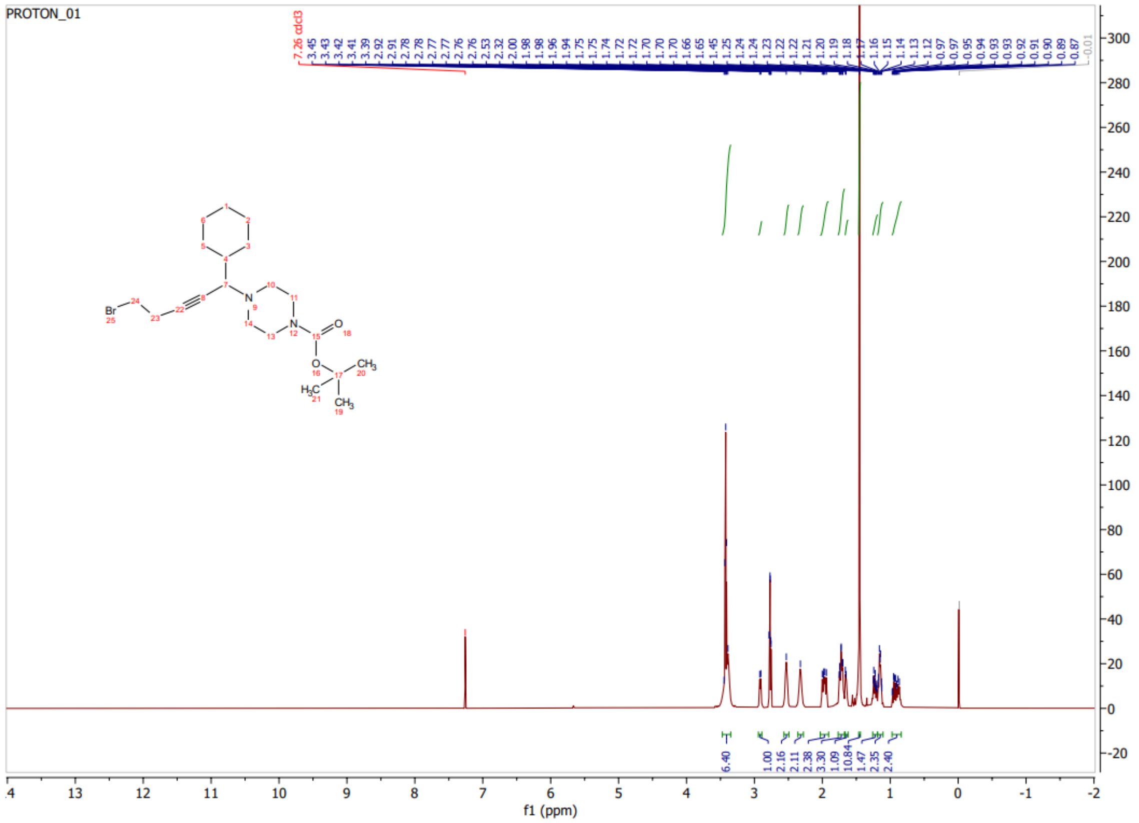

Figure S52 – ^{1}H spectra of compound **2g**.

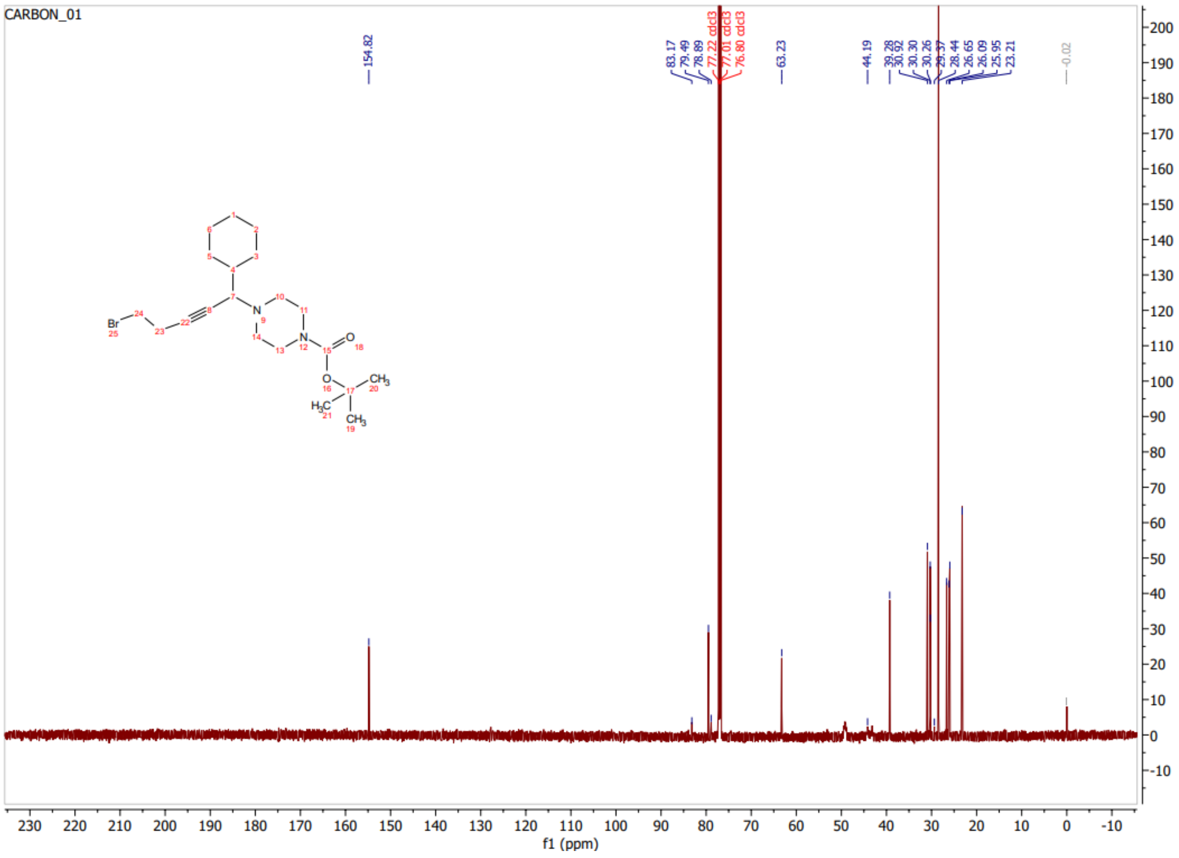

Figure S53 – ^{13}C spectra of compound **2g**.

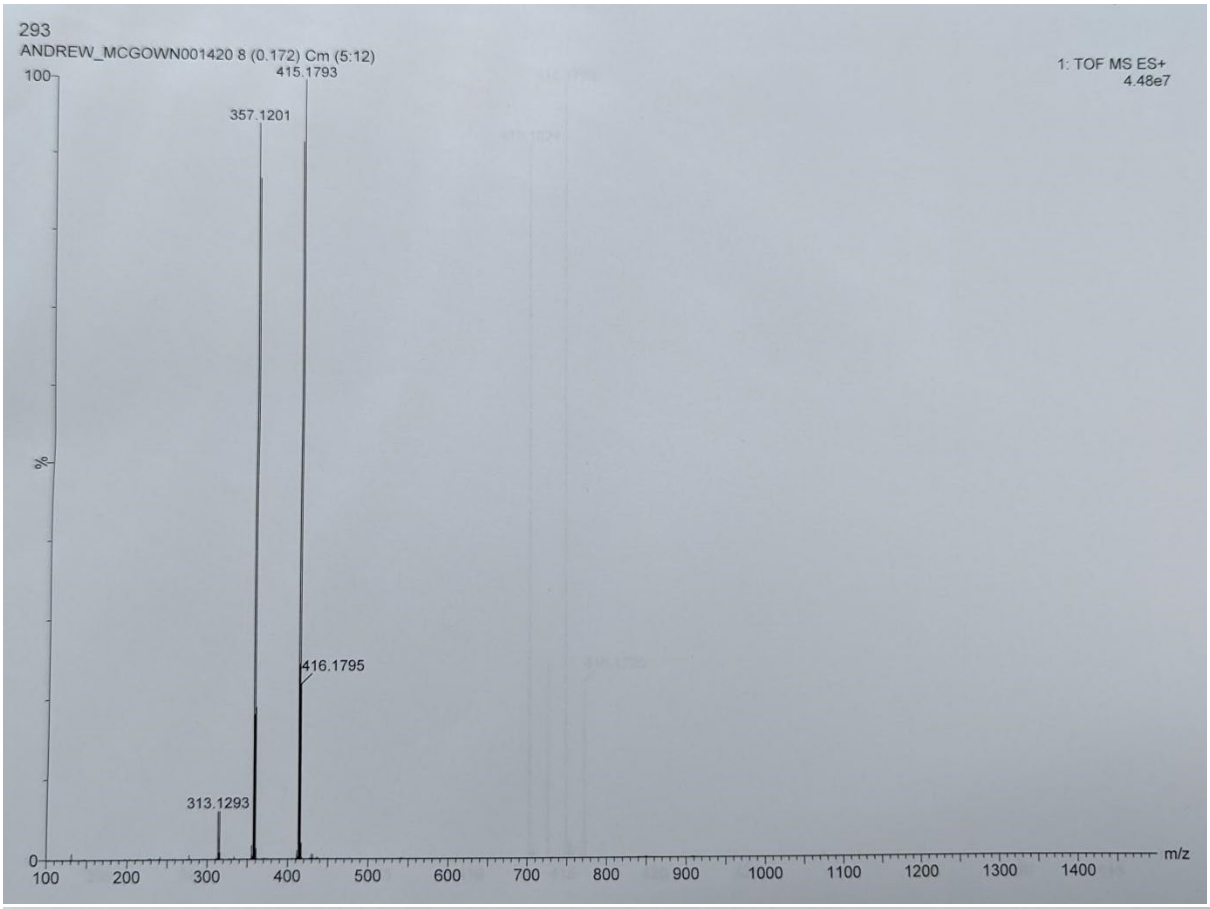

Elemental Composition Report **Page 1**

Single Mass Analysis
Tolerance = 100.0 PPM / DBE: min = -150.0, max = 250.0
Element prediction: Off
Number of isotope peaks used for i-FIT = 3

Monoisotopic Mass, Even Electron Ions
1 formula(e) evaluated with 1 results within limits (up to 50 closest results for each mass)
Elements Used:
C: 20-20 H: 0-100 N: 2-2 O: 2-2 Br: 1-1

293
ANDREW_MCGOWN001420 8 (0.172) AM2 (Ar,20000.0,0.00,0.00); Cm (5:12)

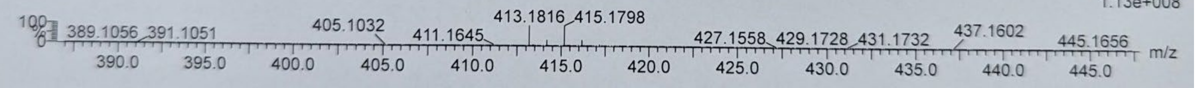

Minimum:				-150.0				
Maximum:		5.0	100.0	250.0				
Mass	Calc. Mass	mDa	PPM	DBE	i-FIT	Norm	Conf(%)	Formula
413.1816	413.1804	1.2	2.9	4.5	848.8	n/a	n/a	C20 H34 N2 O2 Br

Figure S54 – HRMS analysis of compound **2g**.

2h AMKV-102/AMPRO-274

tert-butyl (1-(7-bromo-2-methylhept-4-yn-3-yl)piperidin-4-yl)carbamate.

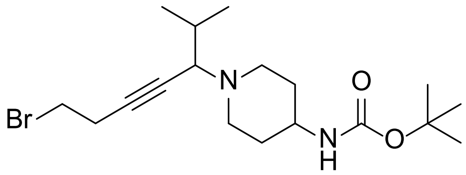

Chemical Formula: $C_{18}H_{31}BrN_2O_2$
Exact Mass: 386.1569
Molecular Weight: 387.3549

Using the general method **2h** was synthesised from isobutyraldehyde (234 mg, 299 µL, 3.3 mmol, 1.0 eq), 4-(N-BOC-amino)piperidine (655 mg, 3.3 mmol, 1.0 eq), and 4-bromo-1-butyne (435 mg, 300 µL, 3.3 mmol, 1.0 eq). **2h** was isolated as a colourless solid (540.5 mg, 1.4 mmol, 41%). ^{1}H NMR (600 MHz, Chloroform-d) δ 4.47 (s, 1H), 3.46 (s, 1H), 3.43 (t, J = 7.0 Hz, 2H), 2.80 – 2.76 (m, 3H), 2.72 -2.69 (m, 1H), 2.56 (m, 1H), 2.50 – 2.43 (m, 1H), 2.19 (t, J = 10.0 Hz, 1H), 1.90 (t, 10.5 Hz, 2H), 1.78 -1.74 (m, 1H), 1.44 (s, 9H), 1.43 (m, 1H), 1.37 - 1.35 (m, 1H), , 1.00 (d, J = 7.0 Hz, 3H), 0.92 (d, J = 7.0 Hz, 3H). ^{13}C NMR (151 MHz, Chloroform-d) δ 155.2, 82.6, 79.5, 79.1, 64.5, 47.8, 45.8, 32.9, 32.6, 30.5, 30.4, 28.4 (3C), 23.2, 20.4, 19.7 (2C). HRMS $C_{18}H_{31}BrN_2O_2$ Calculated [M+H] = 387.1647 and 389.1627. Experimental [M+H] = 387.1664 and 389.1640 (ppm = +0.5) - Br isotopes observed.

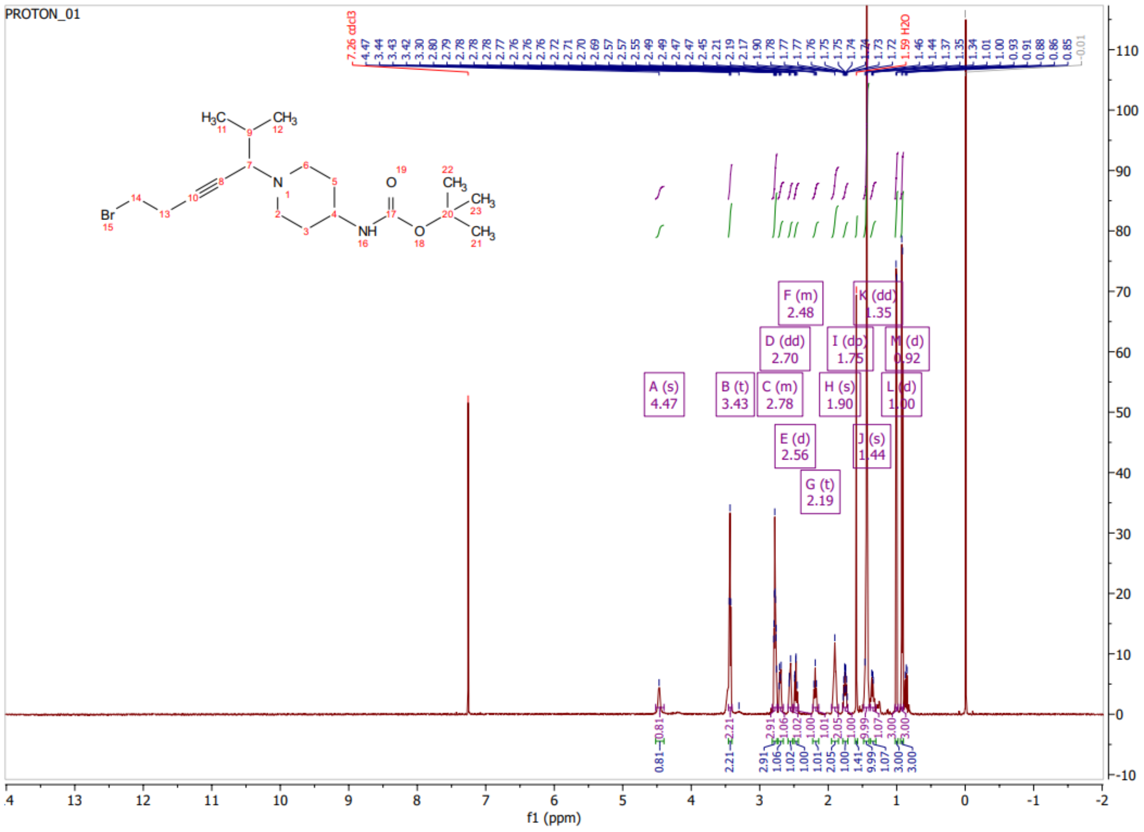

Figure S55 – ^{1}H spectra of compound **2h**.

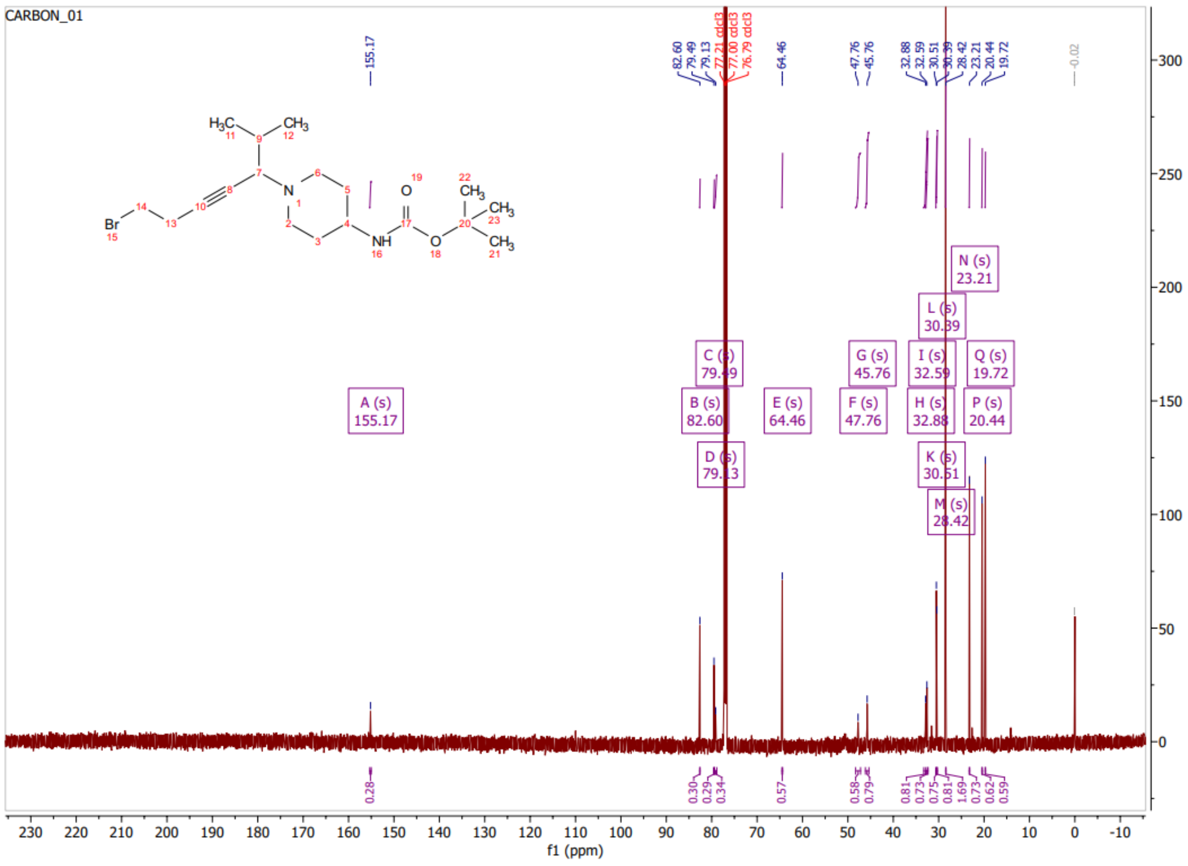

Figure S56 – ^{13}C spectra of compound **2h**.

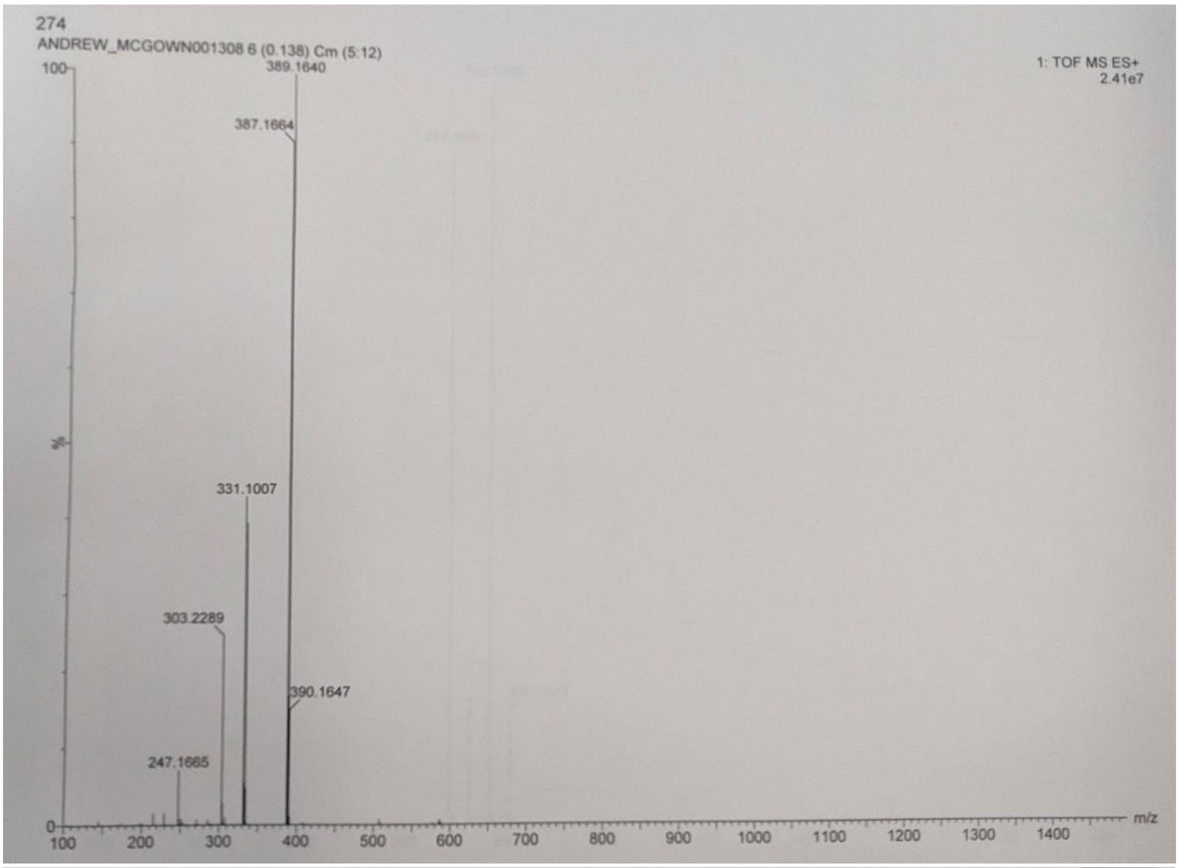

Elemental Composition Report

Single Mass Analysis

Tolerance = 100.0 PPM / DBE: min = -150.0, max = 250.0
Element prediction: Off
Number of isotope peaks used for i-FIT = 3

Monoisotopic Mass, Even Electron Ions
1 formula(e) evaluated with 1 results within limits (up to 50 closest results for each mass)
Elements Used:
C: 18-18 H: 0-100 N: 2-2 O: 2-2 Br: 1-1

274
ANDREW_MCGOWN001308 6 (0.138) AM2 (Ar,20000.0,0.00,0.00); Cm (5:12)

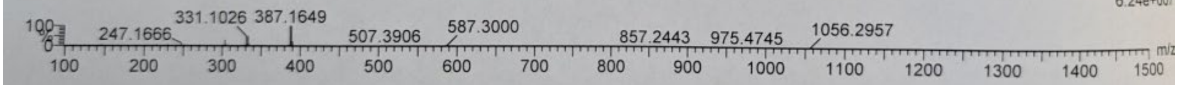

Minimum:				-150.0				
Maximum:		5.0	100.0	250.0				
Mass	Calc. Mass	mDa	PPM	DBE	i-FIT	Norm	Conf(%)	Formula
387.1649	387.1647	0.2	0.5	3.5	907.0	n/a	n/a	C18 H32 N2 O2 Br

Figure S57 – HRMS analysis of compound **2h**.

2i AMPRO-292

tert-butyl 4-(5-hydroxy-1-(tetrahydro-2H-pyran-4-yl)pent-2-yn-1-yl)piperazine-1-carboxylate.

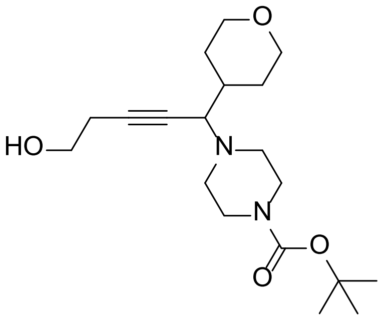

Chemical Formula: $C_{19}H_{32}N_2O_4$
Exact Mass: 352.2362
Molecular Weight: 352.4684

Using the general method **2i** was synthesised from tetrahydro-2H-pyran-4-carboxaldehyde (757mg, 691µL, 6.6mmol, 1.0 eq), 1-BOC-piperazine (1236mg, 6.6mmol, 1.0 eq) and 3-butyn-1-ol (465mg, 500µL, 6.6mmol, 1.0 eq). **2i** was isolated as a viscous, pale-yellow oil (2174mg, 6.17mmol, 93%). ^{1}H NMR (600 MHz, Chloroform-*d*) δ 4.03 – 3.93 (m, 2H), 3.70 (t, *J* = 6.0 Hz, 2H), 3.40 – 3.36 (m, 3H), 3.35 - 3.30 (m, 1H), 2.98 (dt, *J* = 10.0, 2.0 Hz, 1H), 2.57 – 2.47 (m, 4H), 2.34 (s, 2H), 1.87 - 1.84 (m, 2H), 1.74 – 1.68 (m, 4H), 1.46 (s, 9H), 1.35 – 1.23 (m, 2H). ^{13}C NMR (151 MHz, Chloroform-*d*) δ 154.8, 83.6, 79.7, 77.4, 67.8, 67.7, 62.9 (2C), 61.3 (2C), 53.4, 36.9, 31.0, 30.6, 28.4 (3C), 23.0 (2C). HRMS $C_{19}H_{32}N_2O_4$ calculated [M+H] = 353.2440. Experimental [M+H] = 353.2454 (ppm = +4.0).

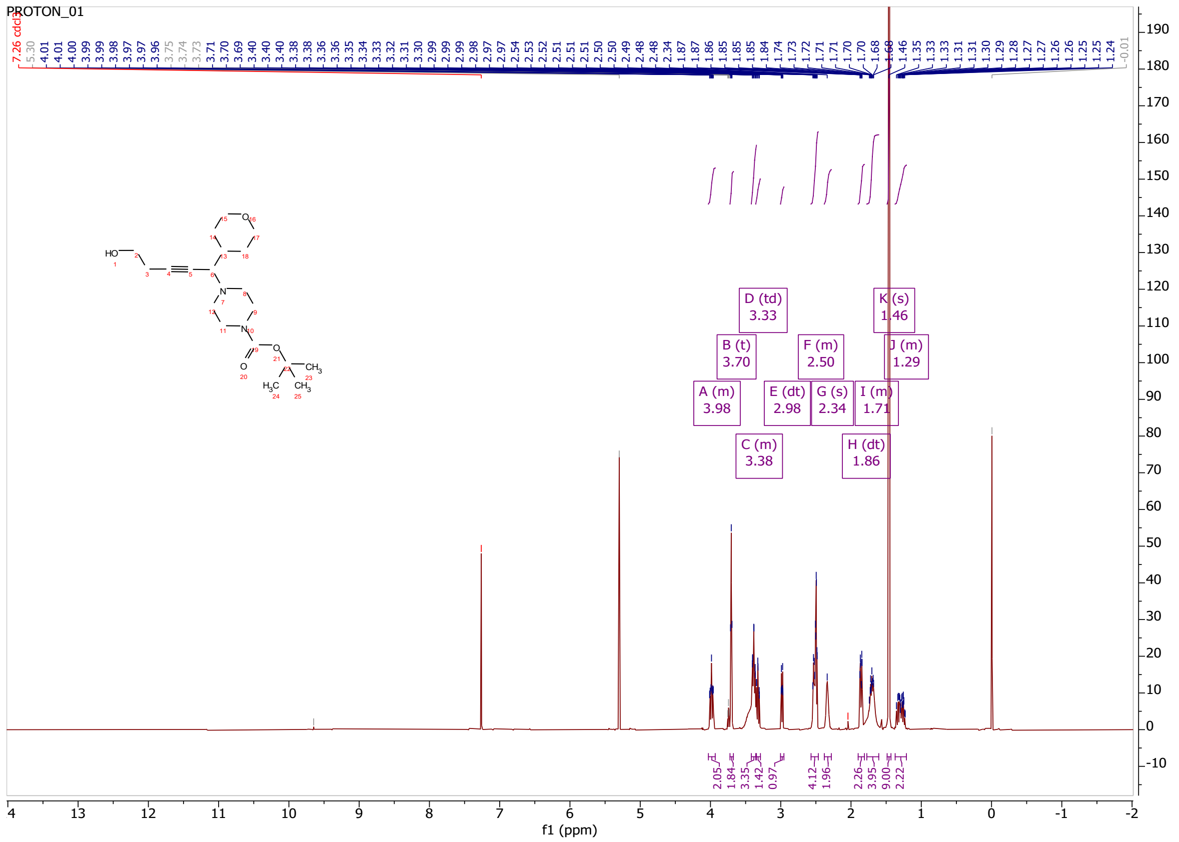

Figure S58 – ^{1}H spectra of compound **2i**.

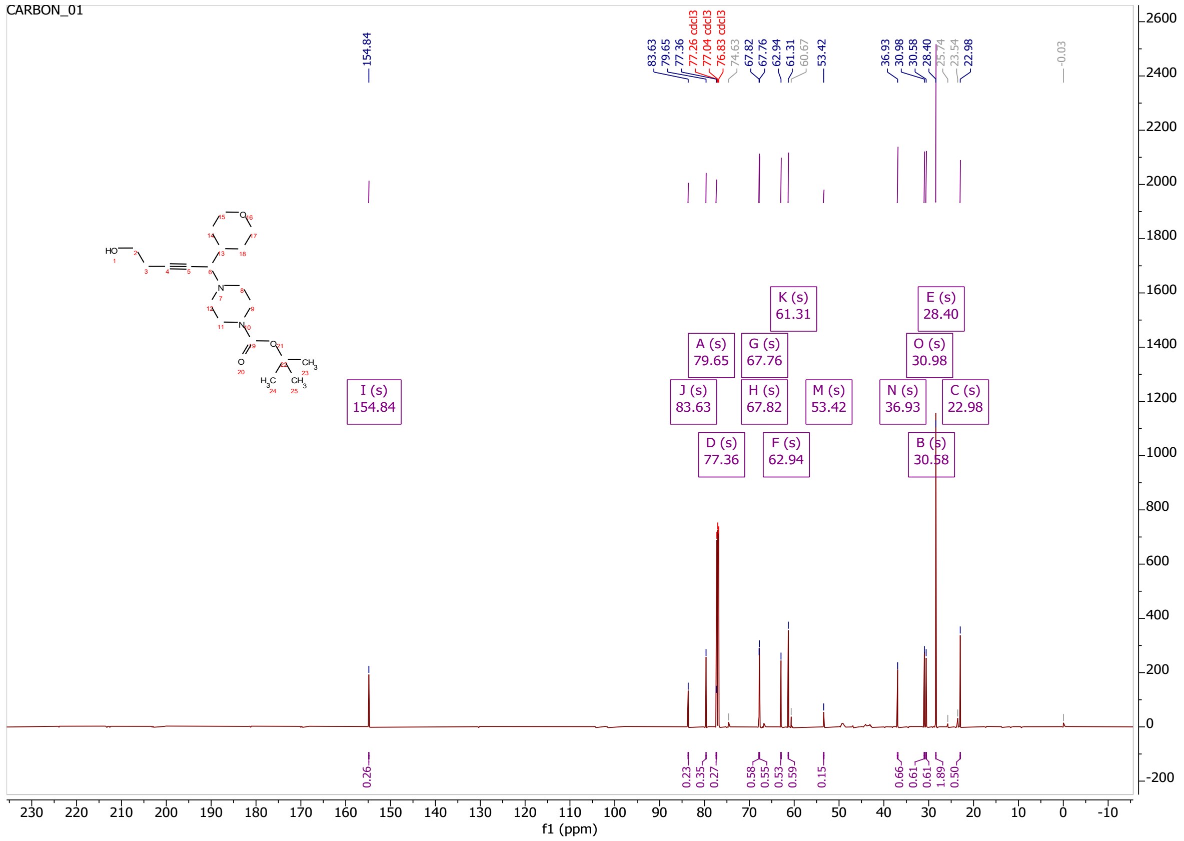

Figure S59 – ^{13}C spectra of compound **2i**.

ANDREW_MCGOWN001419 (292)

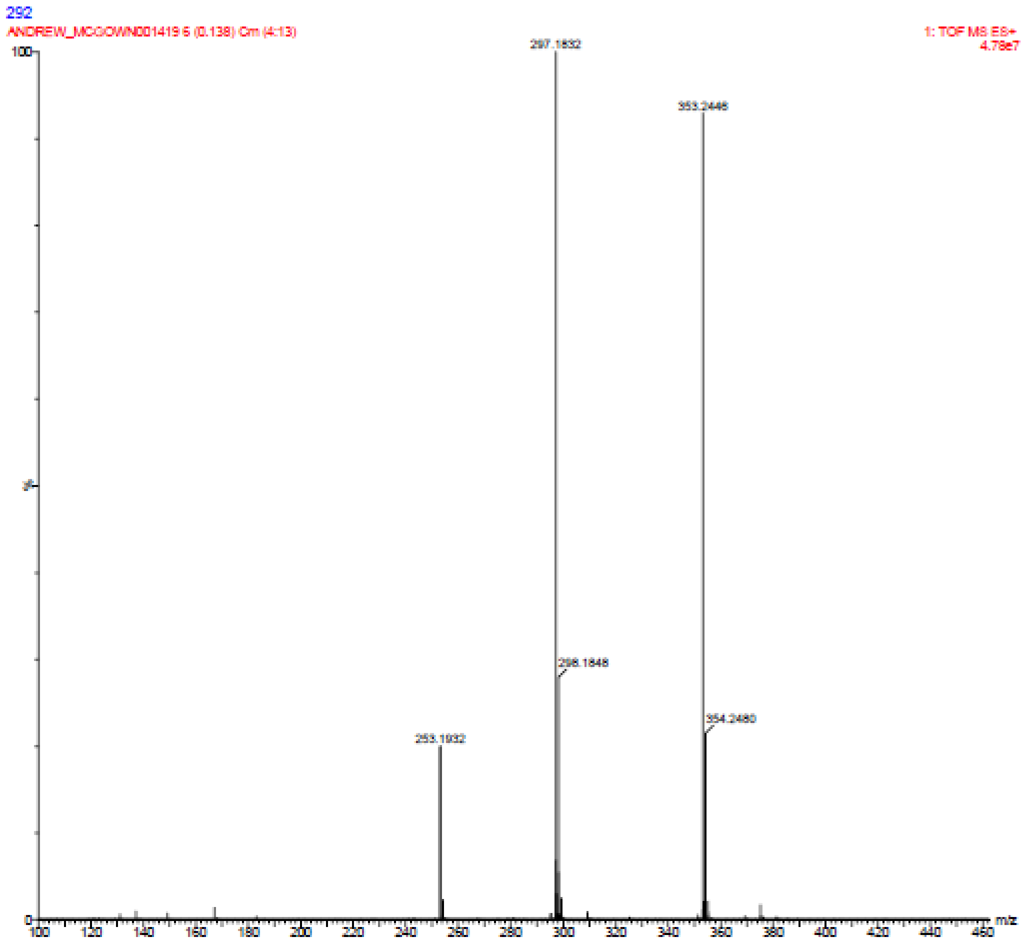

Elemental Composition Report

C: 19-19   H: 0-100   N: 2-2   O: 4-4

Minimum:			-1.5					
Maximum:		5.0	100.0	50.0				
Mass	Calc. Mass	mDa	PPM	DBE	i-FIT	Norm	Conf(%)	Formula
353.2454	353.2440	1.4	4.0	4.5	849.7	n/a	n/a	C19 H33 N2 O4

Figure S60 – HRMS analysis of compound **2i**.

2j AMPRO-268

tert-butyl 4-(5-bromo-1-(4-((tert-butoxycarbonyl)amino)piperidin-1-yl)pent-2-yn-1-yl)piperidine-1-carboxylate

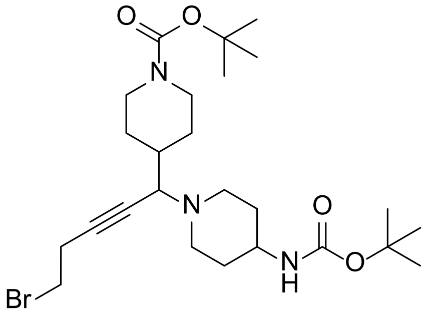

Chemical Formula: $C_{25}H_{42}BrN_3O_4$
Exact Mass: 527.2359
Molecular Weight: 528.5227

Using the general method **2j** was synthesised from N-BOC-4-piperidinecarboxaldehyde (1604mg, 1440µL, 7.5mmol, 1.0 eq) 4-(N-BOC-amino)piperidine (1506mg, 7.5mmol, 1.0eq) and 4-bromo-1-butyne (1000mg, 690µL, 7.5mmol, 1.0 eq). **2j** was isolated as a colourless solid (1082mg, 2.0mmol, 27 %). ^{1}H NMR (600 MHz, Chloroform-*d*) δ 4.47 (d, *J* = 8.0 Hz, 1H), 4.09 (s, 2H), 3.48– 3.46 (m, 1H), 3.43 (t, *J* = 7.0 Hz, 2H), 2.92 (dt, *J* = 10.5, 2.0 Hz, 1H), 2.79 - 2.76 (m, 3H), 2.66 – 2.63 (m, 2H), 2.53 – 2.51 (m, 2H), 2.23 (t, *J* = 10.5 Hz, 1H), 1.93 (t, 12.0 Hz, 3H), 1.64 (s, 1H), 1.60 – 1.56 (m, 1H), 1.44 (s, 9H), 1.43 (s, 9H), 1.35 - 1.31 (m, 1H), 1.14 - 1.00 (m, 2H).^{13}C NMR (151 MHz, Chloroform-*d*) δ 155.2, 154.9, 83.4, 79.3, 79.2, 78.4, 62.5, 47.7, 45.5, 44.2, 38.2, 32.8, 32.5, 30.5 (2C), 30.2, 29.5, 28.5 (3C), 28.4 (3C), 23.2 (2C). HRMS $C_{25}H_{42}BrN_3O_4$ Calculated [M+H] = 528.2437 and 530.2416. Experimental [M+H] = 528.2442 and 530.2429 (ppm = + 0.9) (Br isotopes observed).

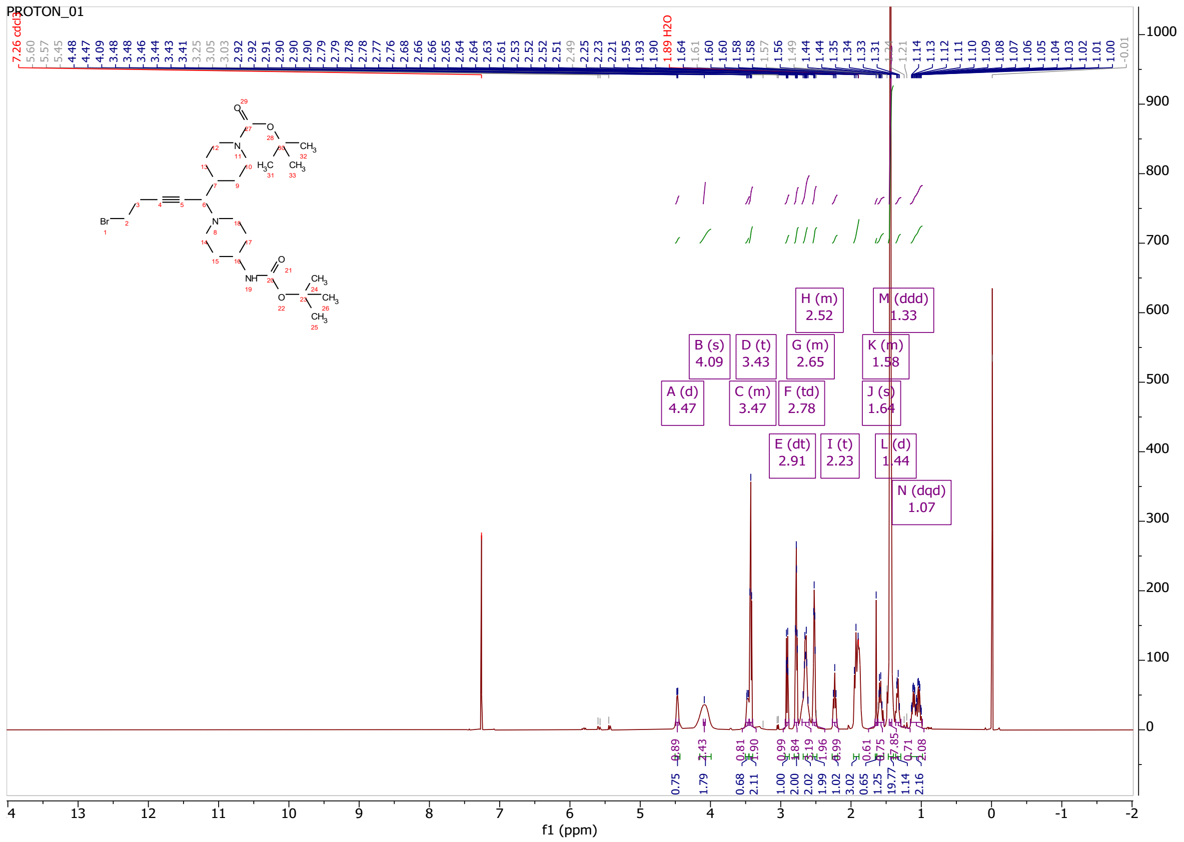

Figure S61 – ^{1}H spectra of compound **2j.**

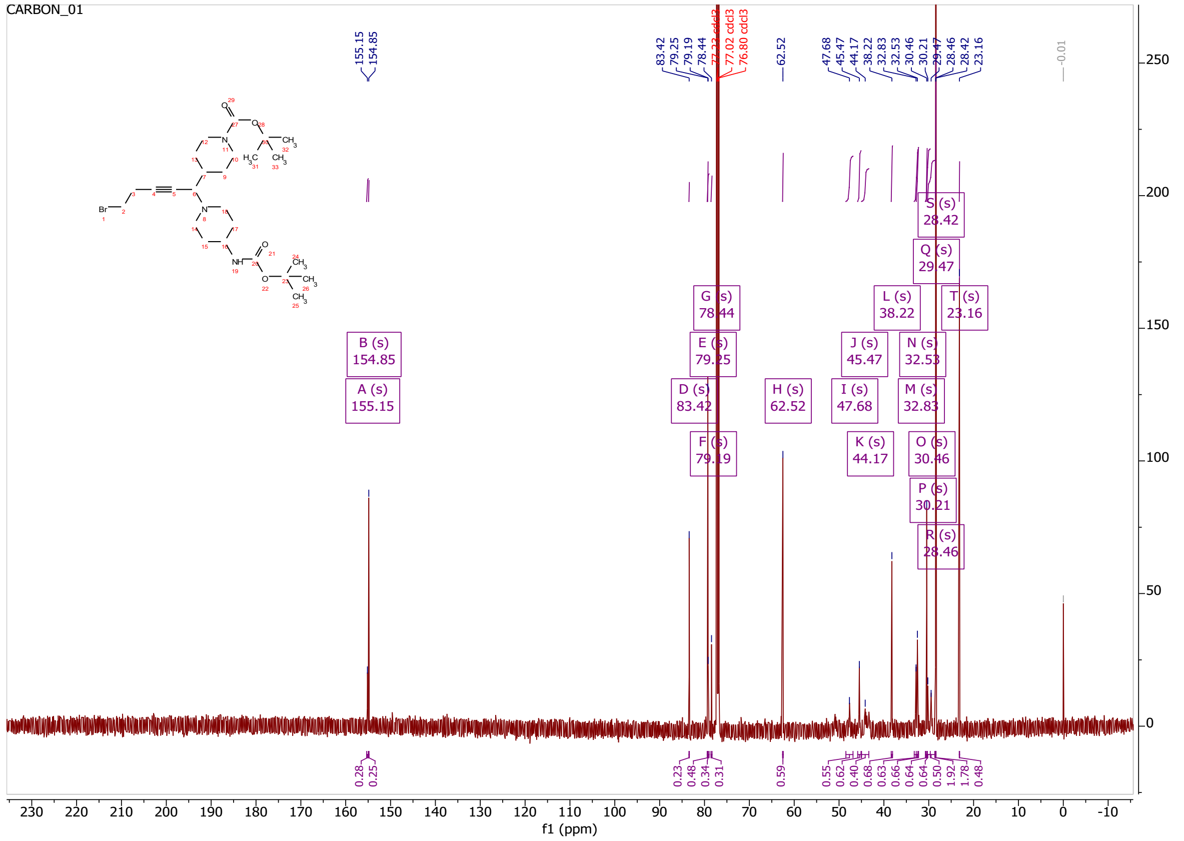

Figure S62 – ^{13}C spectra of compound **2j.**

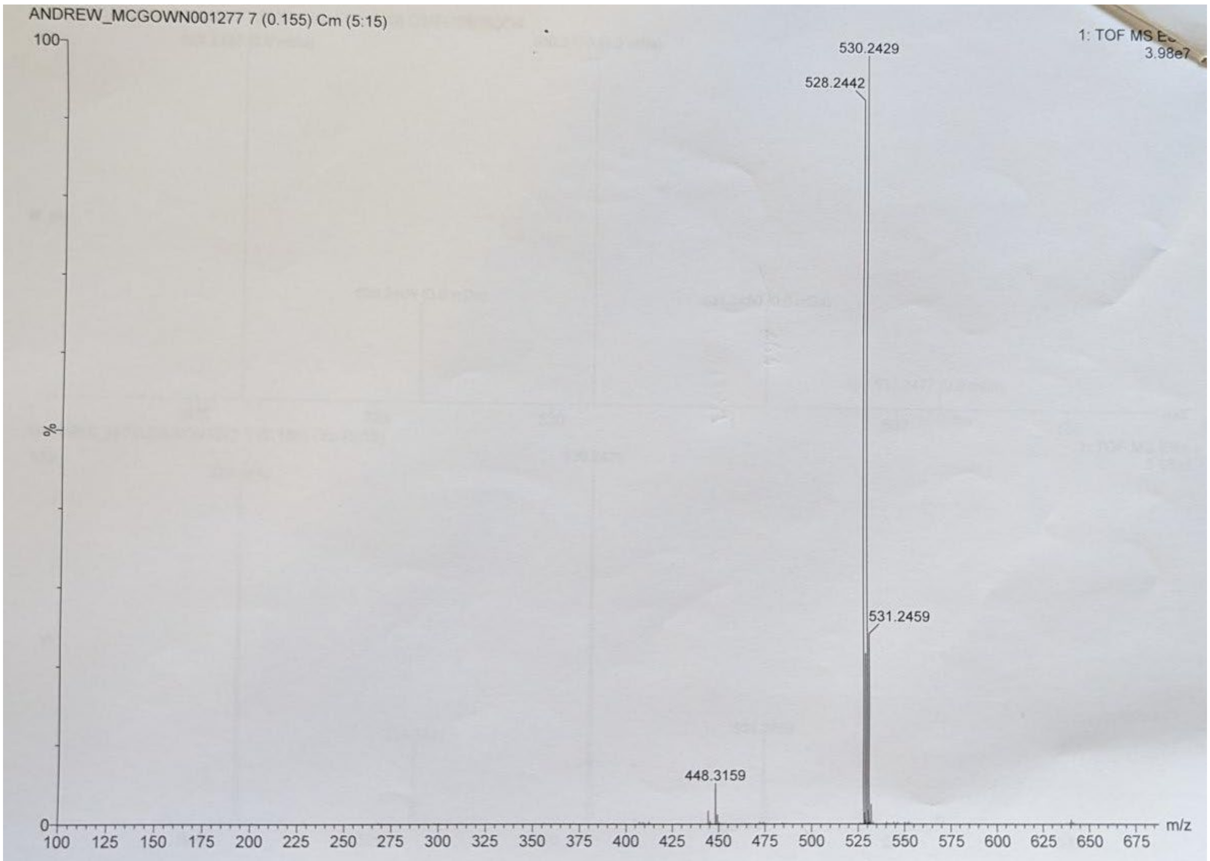

al Composition Report — Page 1

e Mass Analysis

rance = 100.0 PPM / DBE: min = -50.0, max = 75.0
ment prediction: Off

Monoisotopic Mass, Odd and Even Electron Ions
1 formula(e) evaluated with 1 results within limits (up to 50 closest results for each mass)
Elements Used:
C: 25-25 H: 0-1000 N: 3-3 O: 4-4 Br: 1-1

AMPRO-264
ANDREW_MCGOWN001277 33 (0.674)

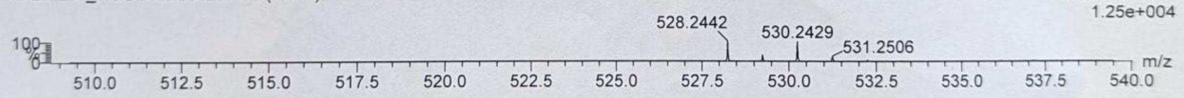

Minimum:				-50.0	
Maximum:		5.0	100.0	75.0	
Mass	Calc. Mass	mDa	PPM	DBE	Formula
528.2442	528.2437	0.5	0.9	5.5	C25 H43 N3 O4 Br

Figure S63 – HRMS analysis for compound **2j**.

2k N2261-045

tert-butyl 4-(1-(1-(tert-butoxycarbonyl)piperidin-4-yl)-4-(2-((S)-4-(4-chlorophenyl)-2,3,9-trimethyl-6H-thieno[3,2-f][1,2,4]triazolo[4,3-a][1,4]diazepin-6-yl)acetamido)but-2-yn-1-yl)piperazine-1-carboxylate.

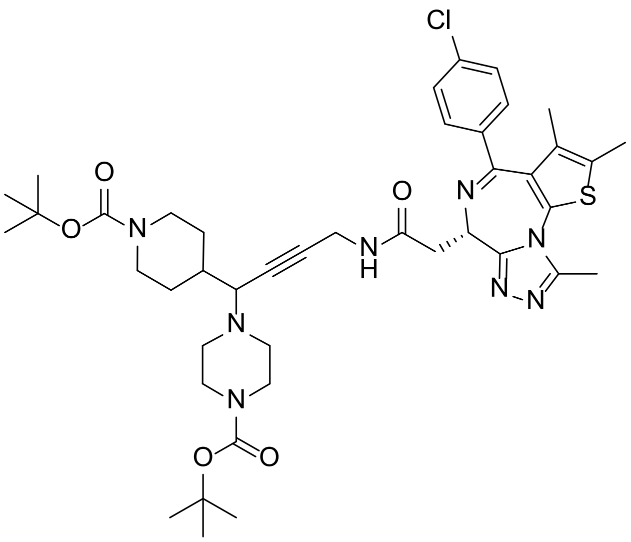

Chemical Formula: $C_{42}H_{55}ClN_8O_5S$
Exact Mass: 818.3705
Molecular Weight: 819.4547

Using the general method **2k** was synthesised from tert-Butyl 4-formylpiperidine-1-carboxylate (74.8mg, 0.35mmol, 1.2 eq), 1-BOC-Piperazine (65.3mg, 0.35mmol, 1.2 eq) and **1a** (128mg, 0.29mmol, 1.0 eq) using the general method. **2k** was isolated as an off white solid (165mg, 0.19mmol, 65% yield).

^{1}H NMR (600 MHz, DMSO-d_6) δ 8.64 – 8.61 (m, 1H), 7.48 (d, *J*= 8.5 Hz, 2H), 7.44 – 7.39 (m, 2H), 4.51 (t, *J*= 7.0 Hz, 1H), 3.98 (d, *J*= 5.0 Hz, 2H), 3.96 – 3.85 (m, 2H), 3.33 – 3.20 (m, 6H), 3.10 (d, *J*= 10.0 Hz, 1H), 2.59 (s, 3H), 2.45 (d, *J*= 8.0 Hz, 2H), 2.41 (s, 3H), 2.32 – 2.25 (m, 2H), 1.86 (d, *J*= 13.0 Hz, 2H), 1.69 – 1.64 (m, 1H), 1.62 (s, 3H), 1.38 (s, 18H), 1.05 – 0.90 (m, 2H). Remaining 2H likely under DMSO peak. ^{13}C NMR (151 MHz, DMSO-d_6) δ 169.2, 163.1, 163.0, 155.1, 153.9, 153.8, 149.9, 136.8, 136.7, 135.3, 132.3, 130.7, 130.1, 129.8, 129.6, 128.5, 83.6, 78.8, 78.4, 77.8, 77.7, 64.9, 61.3, 53.8, 48.6, 43.6, 42.8, 37.4, 37.0, 36.9, 29.6, 29.0, 28.1 (3C), 28.0 (3C), 15.2, 14.1, 12.7, 11.3. HRMS $C_{42}H_{55}ClN_8O_5S$ Calculated [M+H] = 819.3787. Experimental [M+H] = 819.3783 (ppm = + 0.5). LCMS RT 7.094 min, A% = 97 %, Mw [M+H] = 819.75, [M-H] = 817.35.

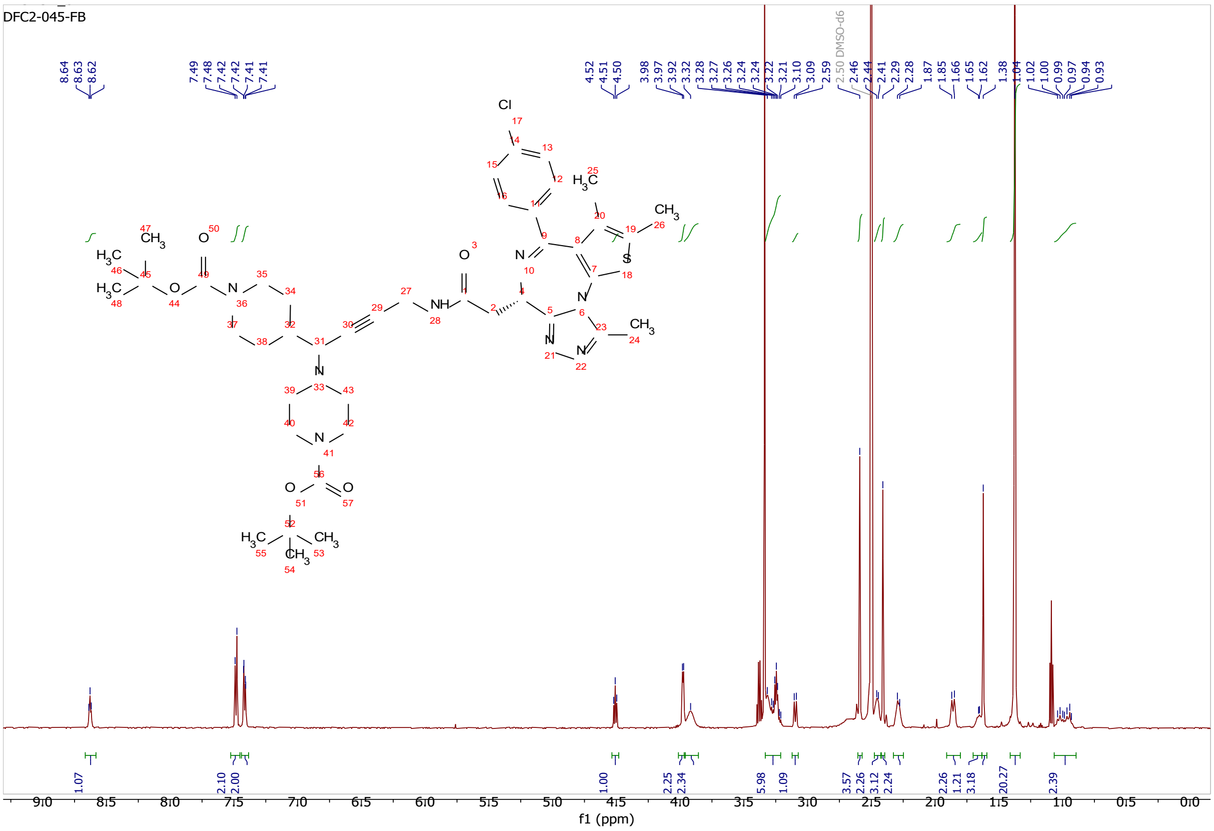

Figure S64 – ^{1}H spectra of compound **2k**.

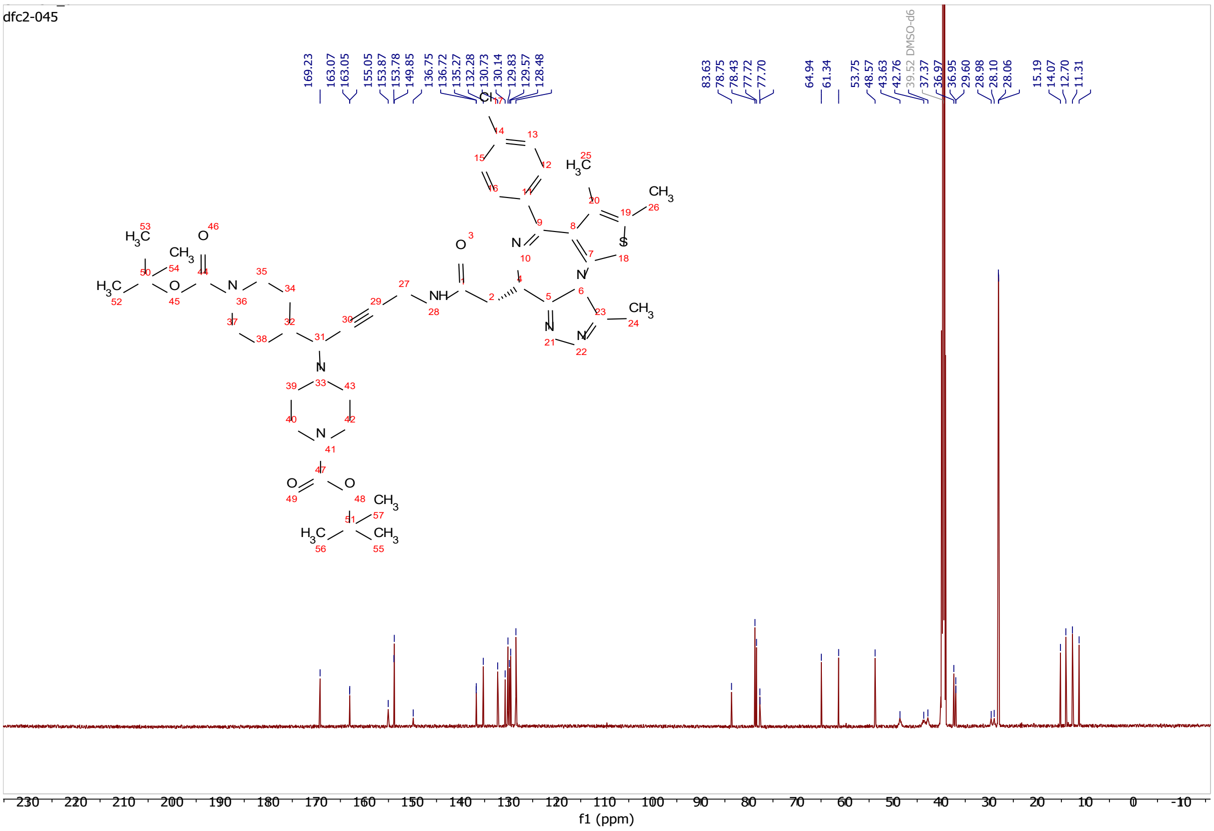

Figure S65 – ^{13}C spectra of compound **2k**.

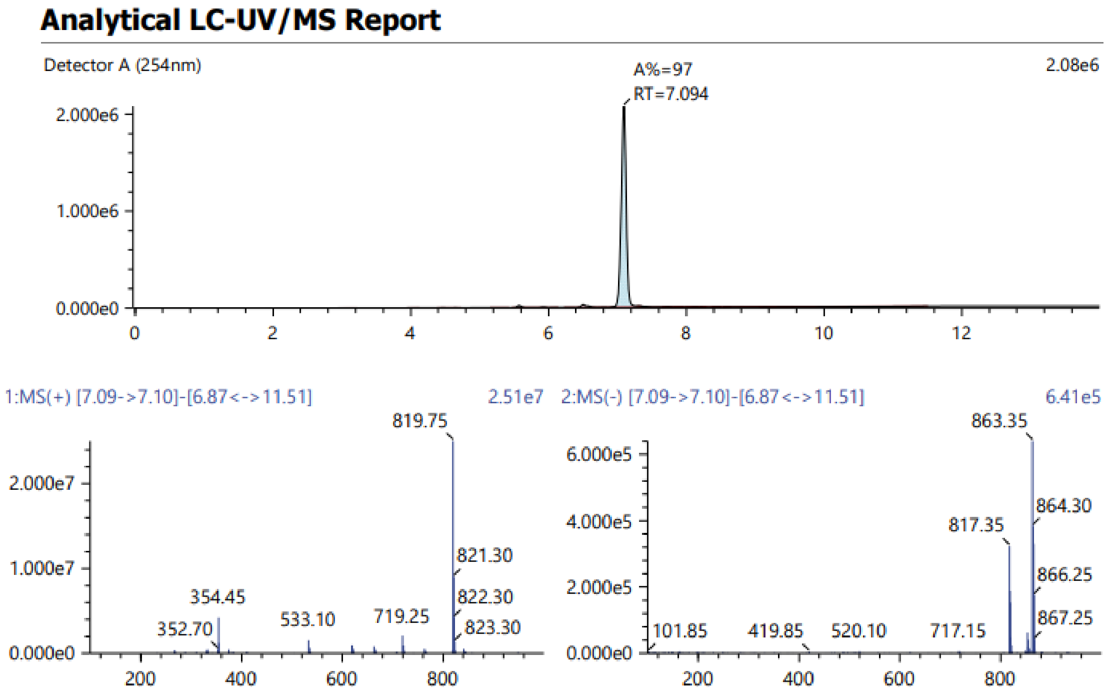

Figure S66 – LCMS analysis of compound **2k**.

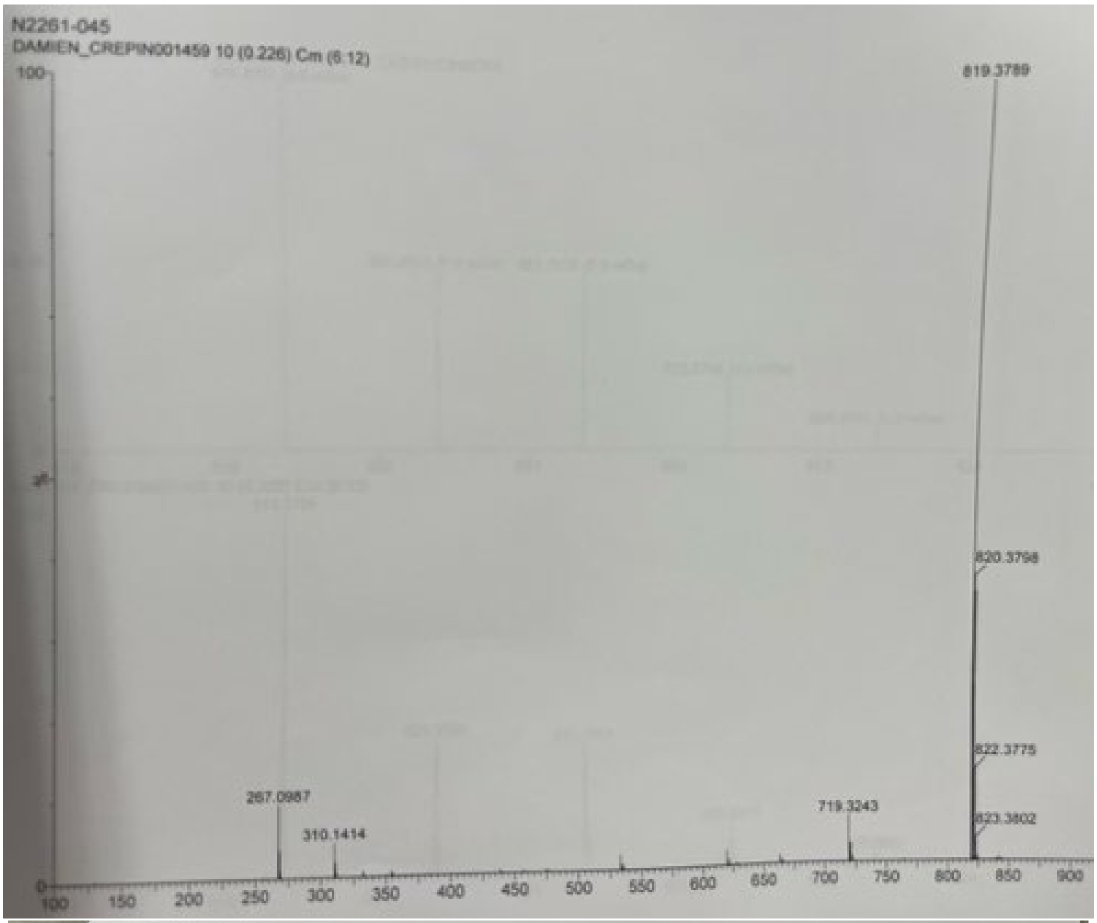

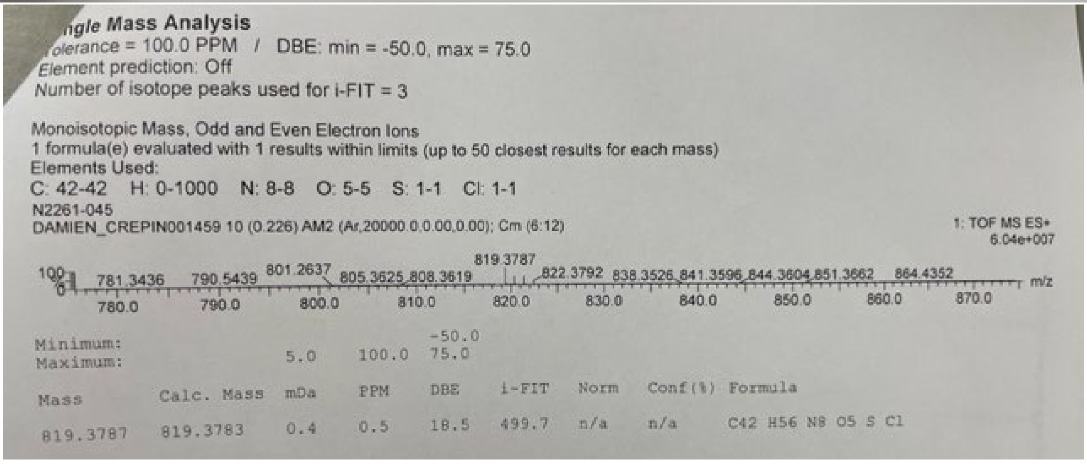

ngle **Mass Analysis**
olerance = 100.0 PPM / DBE: min = -50.0, max = 75.0
Element prediction: Off
Number of isotope peaks used for i-FIT = 3

Monoisotopic Mass, Odd and Even Electron Ions
1 formula(e) evaluated with 1 results within limits (up to 50 closest results for each mass)
Elements Used:
C: 42-42 H: 0-1000 N: 8-8 O: 5-5 S: 1-1 Cl: 1-1
N2261-045
DAMIEN_CREPIN001459 10 (0.226) AM2 (Ar,20000.0,0.00,0.00); Cm (6:12)
1: TOF MS ES+
6.04e+007

Mass	Calc. Mass	mDa	PPM	DBE	i-FIT	Norm	Conf(%)	Formula
Minimum:				-50.0				
Maximum:		5.0	100.0	75.0				
819.3787	819.3783	0.4	0.5	18.5	499.7	n/a	n/a	C42 H56 N8 O5 S Cl

Figure 67 – HRMS analysis of compound **2k**.

2l – N2269-14

tert-butyl (4-(1-(4-allylpiperazin-1-yl)-5-hydroxypent-2-yn-1-yl)cyclohexyl)carbamate.

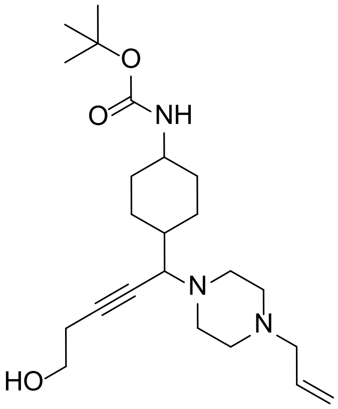

Chemical Formula: $C_{23}H_{39}N_3O_3$
Exact Mass: 405.2991
Molecular Weight: 405.5741

Using the general method **2l** was synthesised from tert-butyl N-(4-formylcyclohexyl)carbamate (4000 mg, 17.6mmol, 1.0 eq), 1-allylpiperazine (2437mg, 2708µL, 19.3mmol, 1.1 eq) and 3-butyn-1-ol (1477mg, 893µL, 21.1mmol, 1.2 eq). **2l** was isolated as a yellow solid (6029.7 mg, 16.2mmol, 92%). ^{1}H NMR (600 MHz, DMSO-d_6) δ 6.66 (d, J = 8.0 Hz, 1H), 5.83 – 5.76 (m, 1H), 5.15 (d, J = 15.0 Hz, 1H), 5.10 (d, J = 10.0 Hz, 1H), 4.75 (t, J = 5.5 Hz, 1H), 3.47 (td, J = 7.0, 5.5 Hz, 2H), 3.15 – 3.08 (m, 1H), 2.93 – 2.82 (m, 3H), 2.49 – 2.14 (m, 10H), 1.98 – 1.90 (m, 2H), 1.79 – 1.72 (m, 2H), 1.36 (s, 9H), 1.31 – 1.26 (m, 1H), 1.15 – 1.00 (m, 2H), 0.95 – 0.88 (m, 1H), 0.85 – 0.78 (m, 1H). ^{13}C NMR (151 MHz, DMSO-d_6) δ 155.2, 136.1, 117.8, 84.1, 77.7, 77.6, 62.4, 61.4, 60.8 (2C), 53.2 (2C), 49.9, 38.7, 32.8, 32.6, 29.9, 29.1, 28.7 (3C), 23.0 (2C). HRMS $C_{23}H_{40}N_3O_3$ Calculated [M+H] 406.3070, Experimental [M+H] 406.3079 (ppm = +2.2).

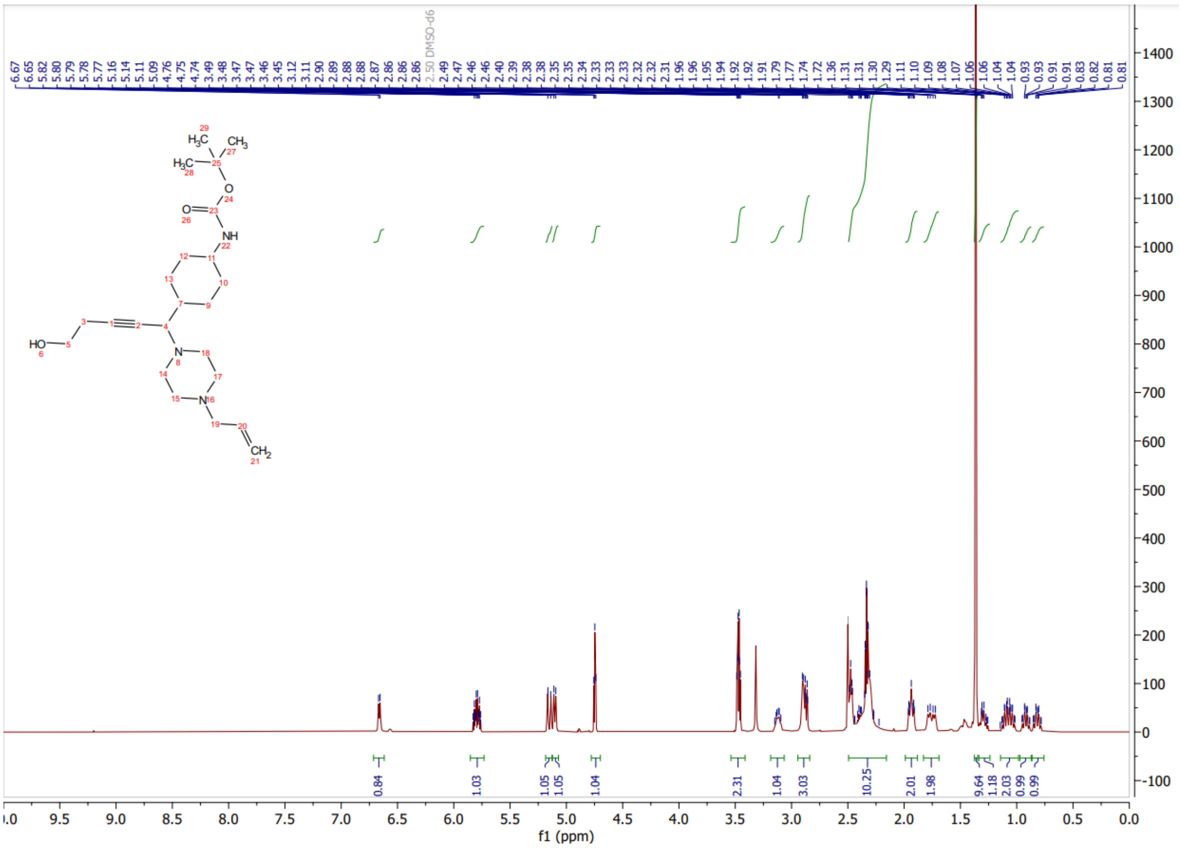

Figure S68 – ^{1}H spectra of compound **2l**.

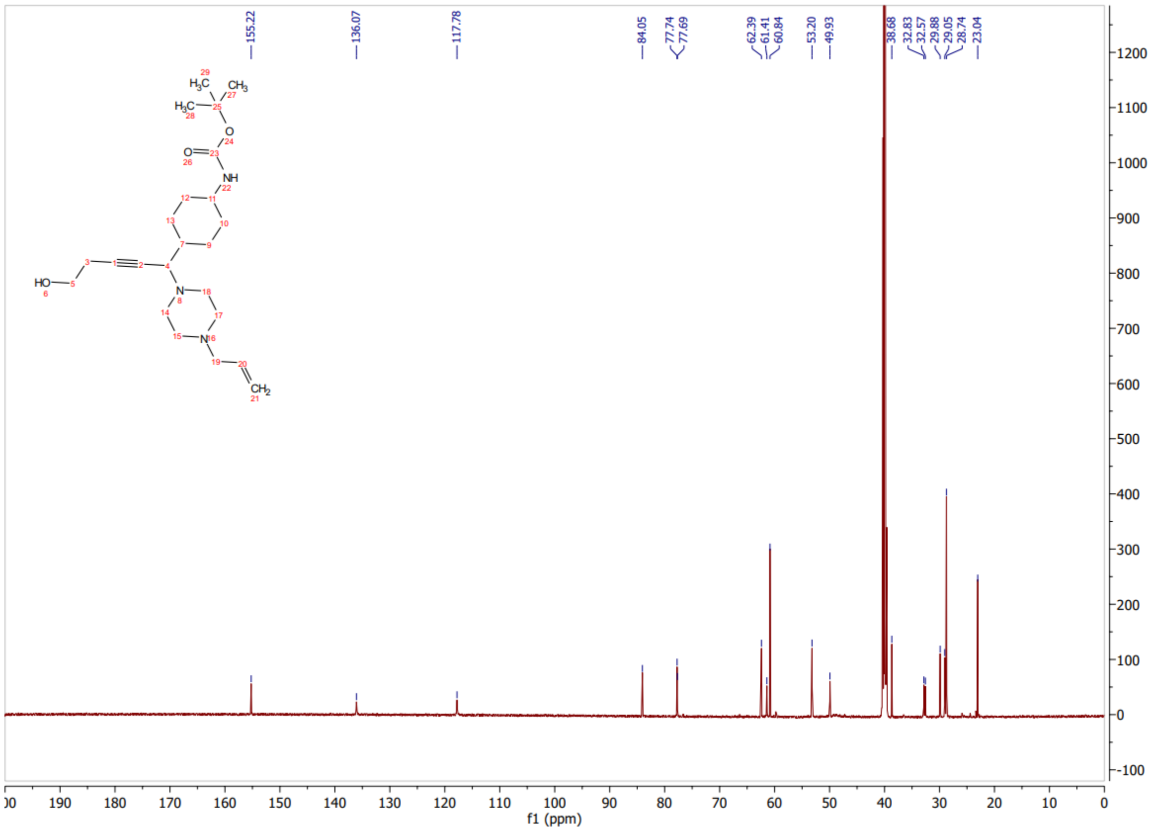

Figure S69 – ^{13}C spectra of compound **2l**.

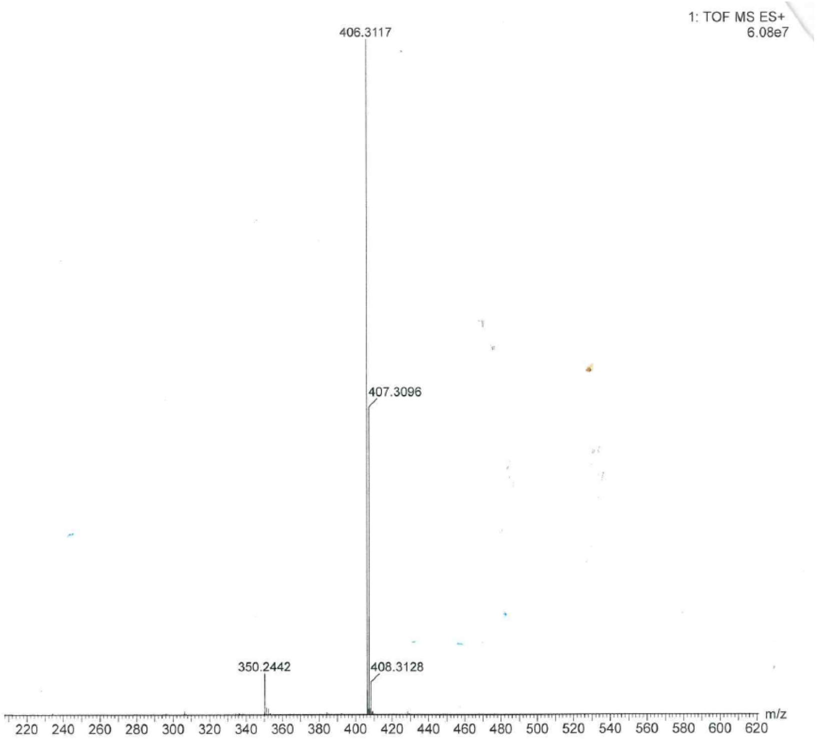

..mental Composition Report

Page 1

Single Mass Analysis
Tolerance = 100.0 PPM / DBE: min = -50.0, max = 80.0
Element prediction: Off
Number of isotope peaks used for i-FIT = 3

Monoisotopic Mass, Even Electron Ions
1 formula(e) evaluated with 1 results within limits (up to 50 closest results for each mass)
Elements Used:
C: 23-23 H: 0-100 N: 3-3 O: 3-3
238540
YAN_LIN001998 4 (0.104) AM2 (Ar,20000.0,0.00,0.00)

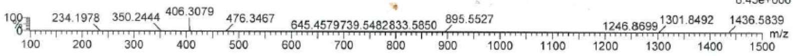

Minimum:				-50.0				
Maximum:		5.0	100.0	80.0				
Mass	Calc. Mass	mDa	PPM	DBE	i-FIT	Norm	Conf(%)	Formula
406.3079	406.3070	0.9	2.2	5.5	665.3	n/a	n/a	C23 H40 N3 O3

Figure S70 – HRMS analysis of compound **2l.**

2m – N2269-13

tert-butyl (4-(5-hydroxy-1-(piperazin-1-yl)pent-2-yn-1-yl)cyclohexyl)carbamate.

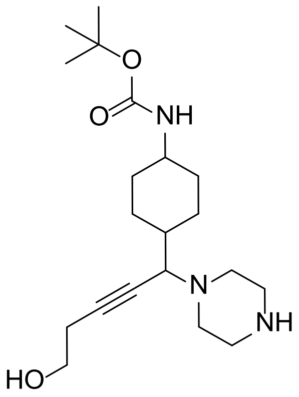

Chemical Formula: $C_{20}H_{35}N_3O_3$
Exact Mass: 365.2678
Molecular Weight: 365.5102

To a solution of tert-butyl **2l** (1440mg, 3.6mmol, 1.0 eq) in dry tetrahydrofuran (15mL) was added Pd(dba)$_2$ (204.2mg,0.4mmol, 0.1 eq), dppb (151.4mg, 0.4mmol, 0.1 wq), and thiosalicylic acid (821.2mg, 5.3mmol, 1.5 eq) . The reaction mixture was stirred under an inert atmosphere at 60ºC for 12 hours. Upon completion the resulting crude mixture was filtered through celite and concentrated under reduced pressure. The residue was dissolved in methanol (10mL) and Isolute MP-TMT metal scavenger (1.5 g) was added before stirring overnight. The resin was removed by filtration and the mixture was concentrated to residue before purification by preparative HPLC (C18, 20 x 150 mm, 5 µM) to yield **2m** as a colourless solid (600mg, 1.39mmol, 39%). ^{1}H NMR (600 MHz, DMSO-d_6) δ 8.37 (s, 1H), 6.69 (d, J = 8.0 Hz, 1H), 3.50 – 3.46 (m, 2H), 3.17 – 3.05 (m, 1H), 2.96 – 2.87 (m, 5H), 2.59 – 2.56 (m, 2H), 2.43 – 2.39 (m, 2H), 2.35 – 2.32 (m, 2H), 1.97 – 1.92 (m, 2H), 1.79 – 1.72 (m, 2H), 1.36 (s, 9H), 1.33 – 1.30 (m, 1H), 1.14 – 1.03 (m, 2H), 0.96 – 0.89 (m, 1H), 0.87 – 0.80 (m, 1H). Alcohol OH proton not observed. ^{13}C NMR (151 MHz, DMSO-d_6) δ 165.9, 155.2, 84.5, 77.7, 77.3, 62.7, 60.7 (2C), 49.9, 44.1 (2C), 38.5, 32.8, 32.5, 29.8, 29.0, 28.7 (3C), 23.1 (2C). HRMS $C_{20}H_{36}N_3O_3$ calculated [M+H] = 366.2757, experimental [M+H]=: 366.2774 (ppm = 4.6 ppm).

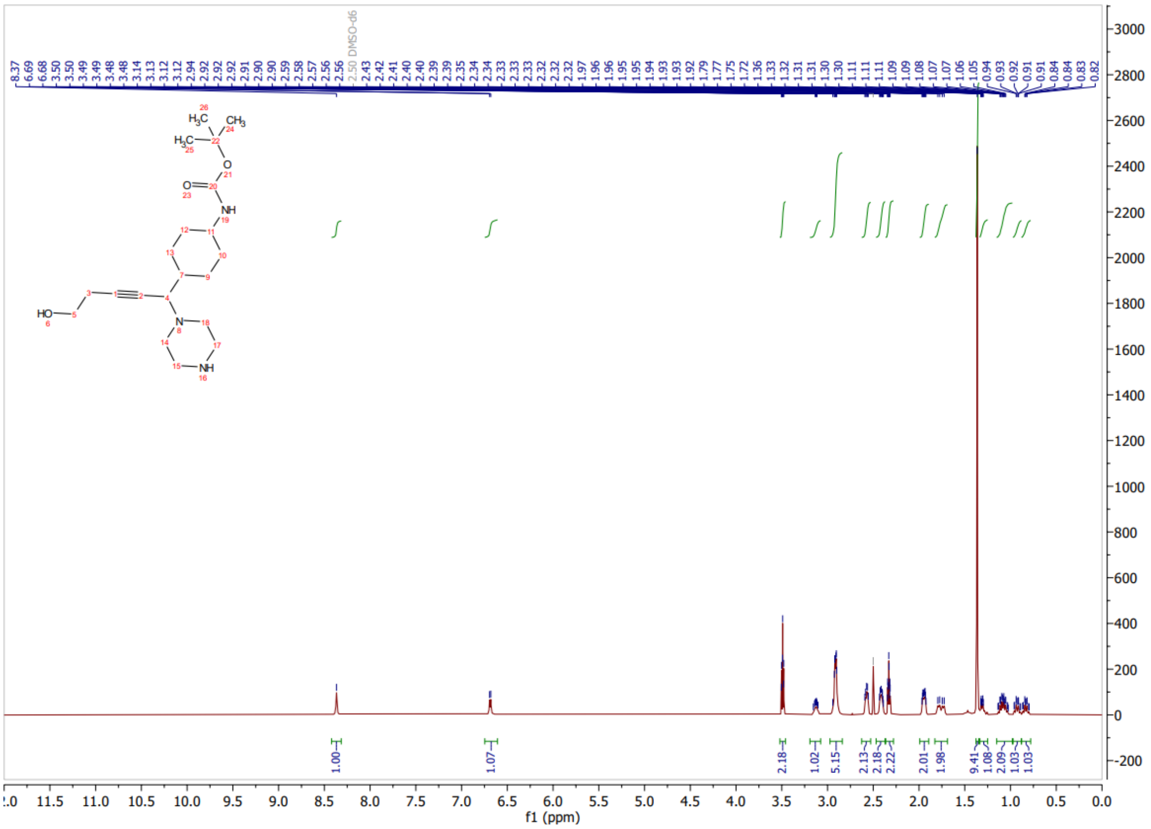

Figure S71 – ^{1}H spectra of compound **2m**.

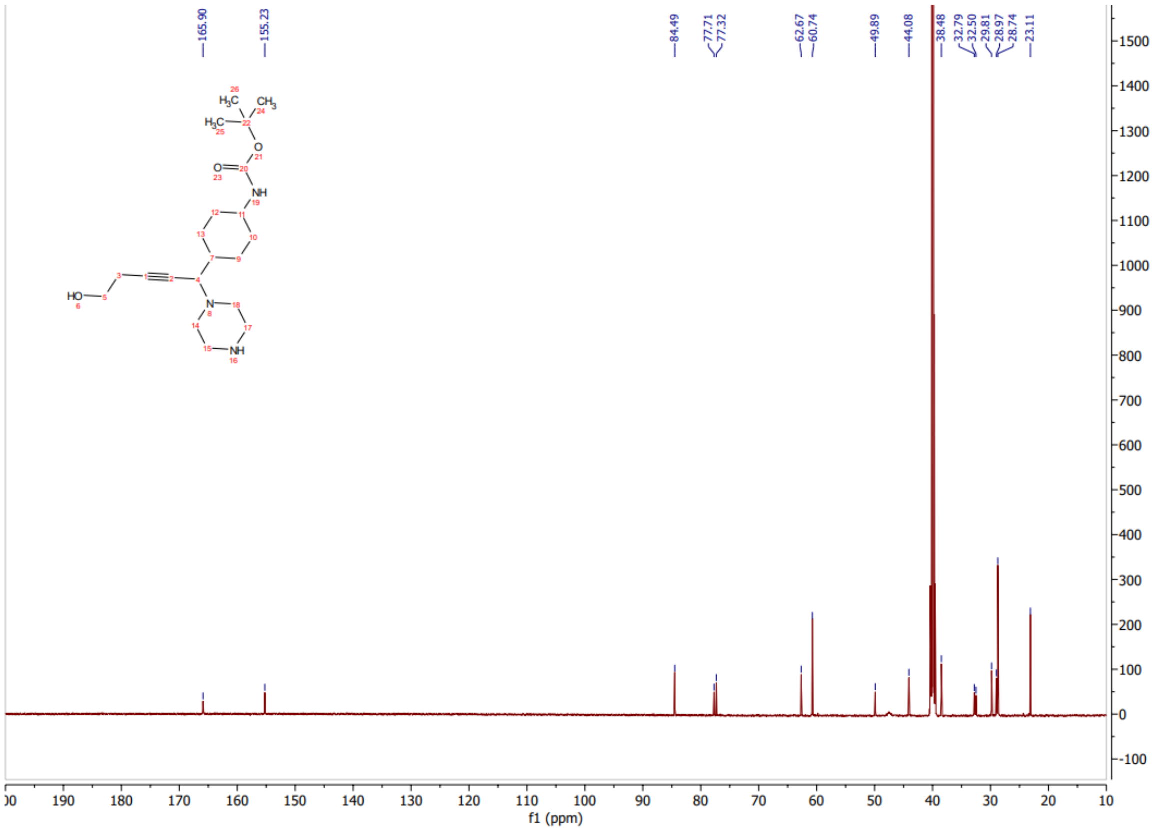

Figure S72 – ^{13}C spectra of compound **2m.**

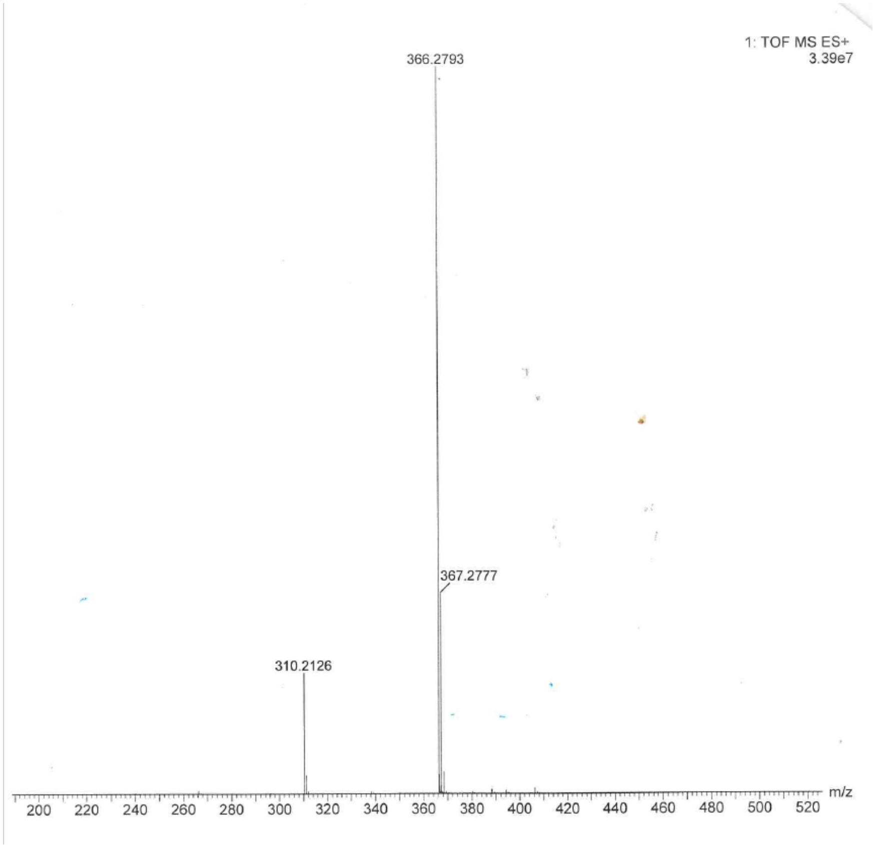

mental Composition Report

Page 1

Single Mass Analysis
Tolerance = 100.0 PPM / DBE: min = -50.0, max = 80.0
Element prediction: Off
Number of isotope peaks used for i-FIT = 3

Monoisotopic Mass, Odd and Even Electron Ions
1 formula(e) evaluated with 1 results within limits (up to 50 closest results for each mass)
Elements Used:
C: 20-20 H: 0-100 N: 3-3 O: 3-3
YI-238460
YAN_LIN002015 6 (0.138) AM2 (Ar,20000.0,0.00,0.00); Cm (3:13)

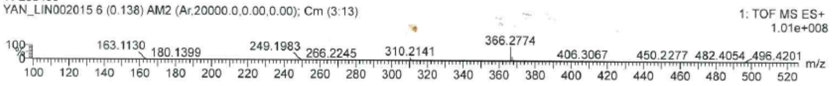

Minimum:				-50.0				
Maximum:		5.0	100.0	80.0				
Mass	Calc. Mass	mDa	PPM	DBE	i-FIT	Norm	Conf(%)	Formula
366.2774	366.2757	1.7	4.6	4.5	822.5	n/a	n/a	C20 H36 N3 O3

Figure S73 – HRMS spectra of compound **2m.**

3 – N2268-20

10-(2-carboxyethyl)-5,5-difluoro-1,3,7,9-tetramethyl-5H-dipyrrolo[1,2-c:2',1'-f][1,3,2]diazaborinin-4-ium-5-uide.

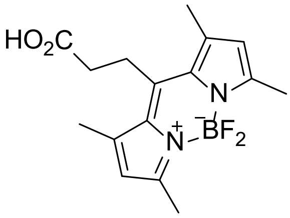

Chemical Formula: $C_{16}H_{19}BF_2N_2O_2$
Exact Mass: 320.1508
Molecular Weight: 320.1421

Compound **3** was synthesised using the method outlined in previously reported literature [1]. **3** was isolated as a red solid (420mg, 1.18mmol, 24%). Data reported was in concordance with previously reported. ^{1}H NMR (600 MHz, Chloroform-*d*) δ 6.08 (s, 2H), 3.36 – 3.30 (m, 2H), 2.69 – 2.63 (m, 2H), 2.52 (s, 6H), 2.45 (s, 6H). Carboxylic acid OH was not observed.

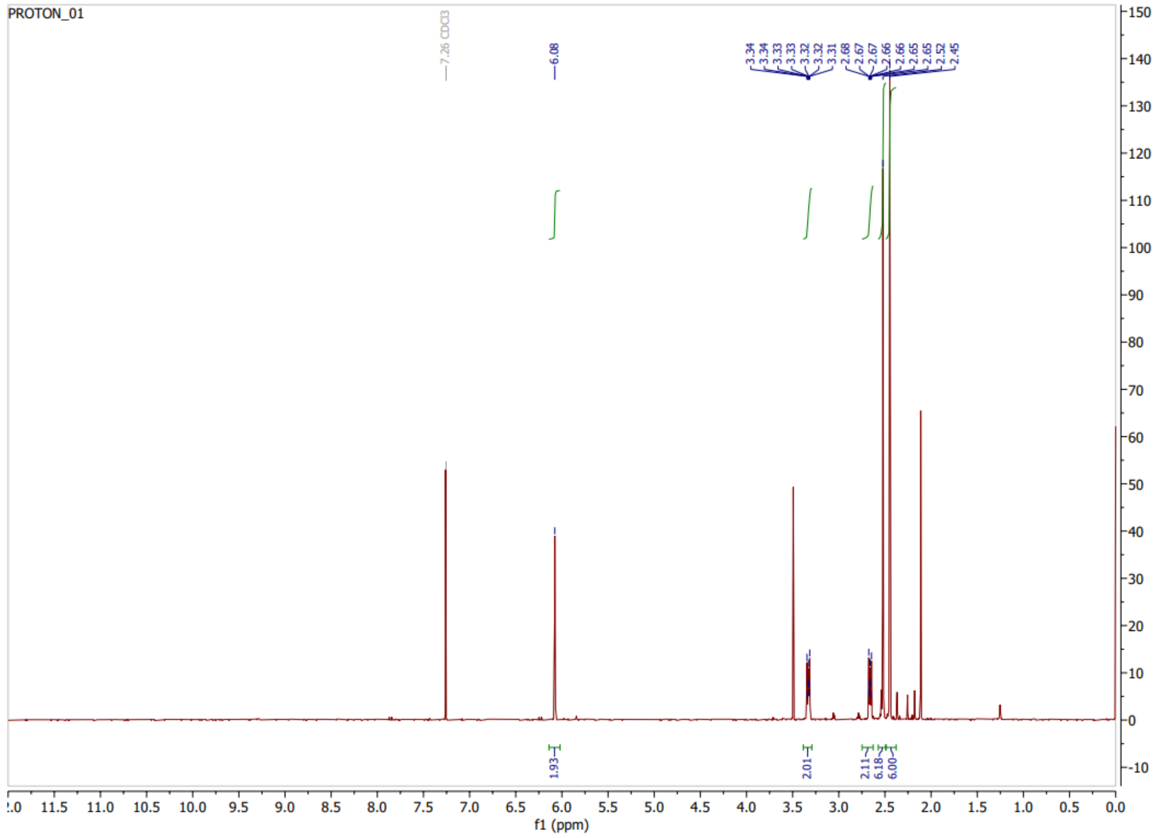

Figure S74 – ^{1}H Spectra of compound **3**.

4a – N2268-49

10-(3-(4-(1-(4-((tert-butoxycarbonyl)amino)cyclohexyl)-5-hydroxypent-2-yn-1-yl)piperazin-1-yl)-3-oxopropyl)-5,5-difluoro-1,3,7,9-tetramethyl-5H-dipyrrolo[1,2-c:2',1'-f][1,3,2]diazaborinin-4-ium-5-uide.

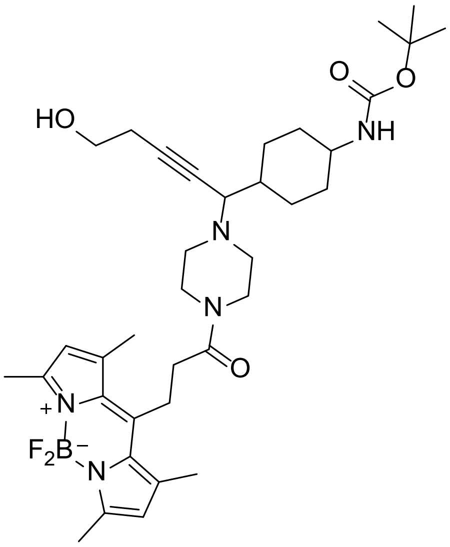

Chemical Formula: $C_{36}H_{52}BF_2N_5O_4$
Exact Mass: 667.4080
Molecular Weight: 667.6370

A mixture of **3** (401.8mg, 0.96mmol, 1.0eq), N,N-diisopropylethylamine (670µL, 3.85mmol, 4.0eq) in DMF (8mL) was stirred for 5 minutes to ensure full dissolution before **2m** (352mg, 0.96mmol, 1.0eq) was added and the reaction mixture was stirred at room temperature for 12 hours. Upon completion the reaction was diluted with ethyl acetate (10mL) and the resulting organics were washed with aqueous NH_4Cl (2 x 10mL) and brine (10mL). The organics were dried over $MgSO_4$ and concentrated to residue. The residue was purified by column chromatography (SiO_2, 12g, DCM: MeOH) to yield **4a** as a red solid (530mg, 0.79mmol, 82%). ^{1}H NMR (600 MHz, Chloroform-*d*) δ 6.06 (s, 2H), 4.39 (d, *J* = 8.0 Hz, 1H), 3.69 (t, *J* = 6.5 Hz, 3H), 3.57 (s, 1H), 3.40 – 3.37 (m, 2H), 3.37 – 3.32 (m, 2H), 2.90 (dt, *J* = 10.0, 2.0 Hz, 1H), 2.61 – 2.57 (m, 2H), 2.56 – 2.52 (m, 2H), 2.51 (s, 6H), 2.48 (td, *J* = 6.5, 2.0 Hz, 2H), 2.40 (s, 6H), 2.33 (m, 2H), 2.16 (s, 1H), 2.02 (m, 4H), 1.43 (s, 9H), 1.12 – 0.93 (m, 4H). Amide NH not observed. ^{13}C NMR (151 MHz, Chloroform-*d*) δ 169.0, 154.4 (2C), 145.1 (2C), 140.4, 131.3 (2C), 121.8 (2C), 83.4, 77.7, 62.9, 61.3 (2C), 45.4 (2C), 42.0, 38.6, 34.3 (2C), 33.2, 33.0, 30.9, 29.6, 28.9, 28.4 (3C), 23.6, 23.0 (2C), 16.5 (2C), 14.4 (CF_4 = 2.5 Hz, 2C). HRMS $C_{36}H_{52}BF_2N_5O_4$ Calculated [M+H] = 668.4159. Experimental [M+H] = 668.4155 (ppm = -3.7).

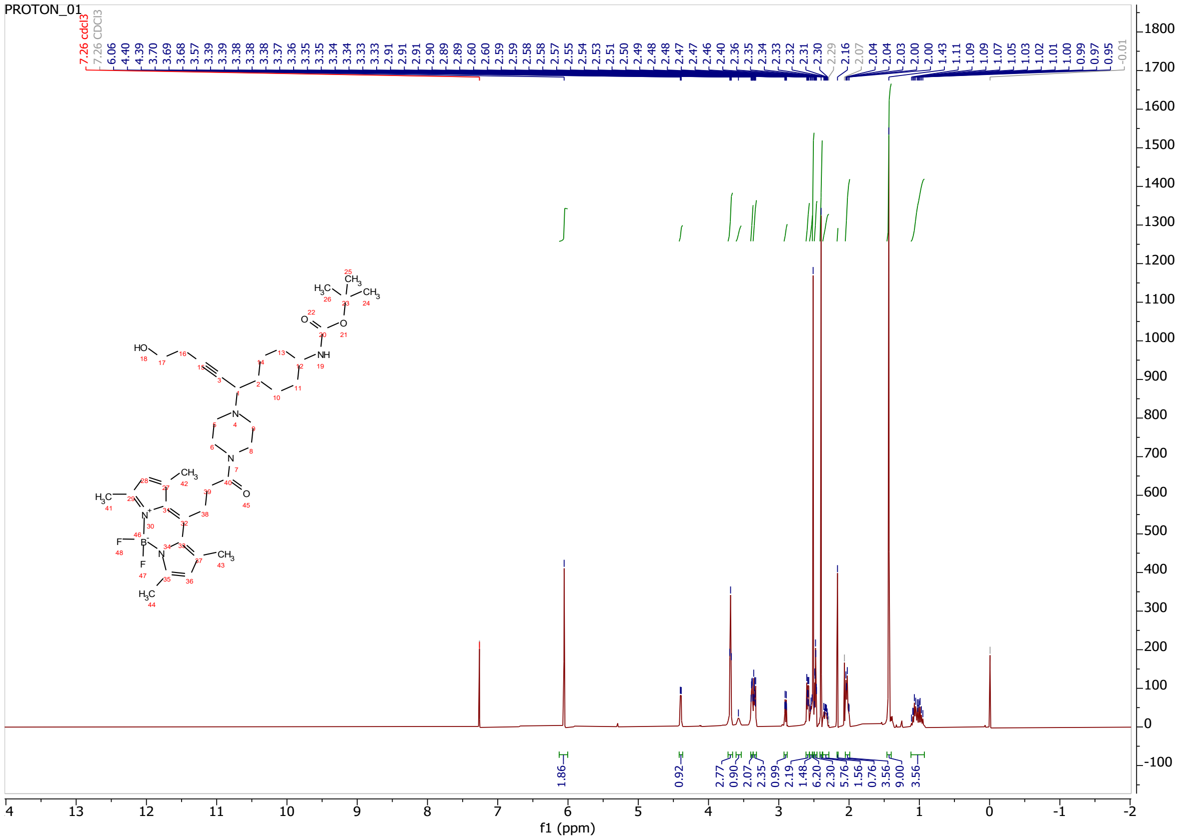

Figure S75 – ^{1}H Spectra of compound **4a**.

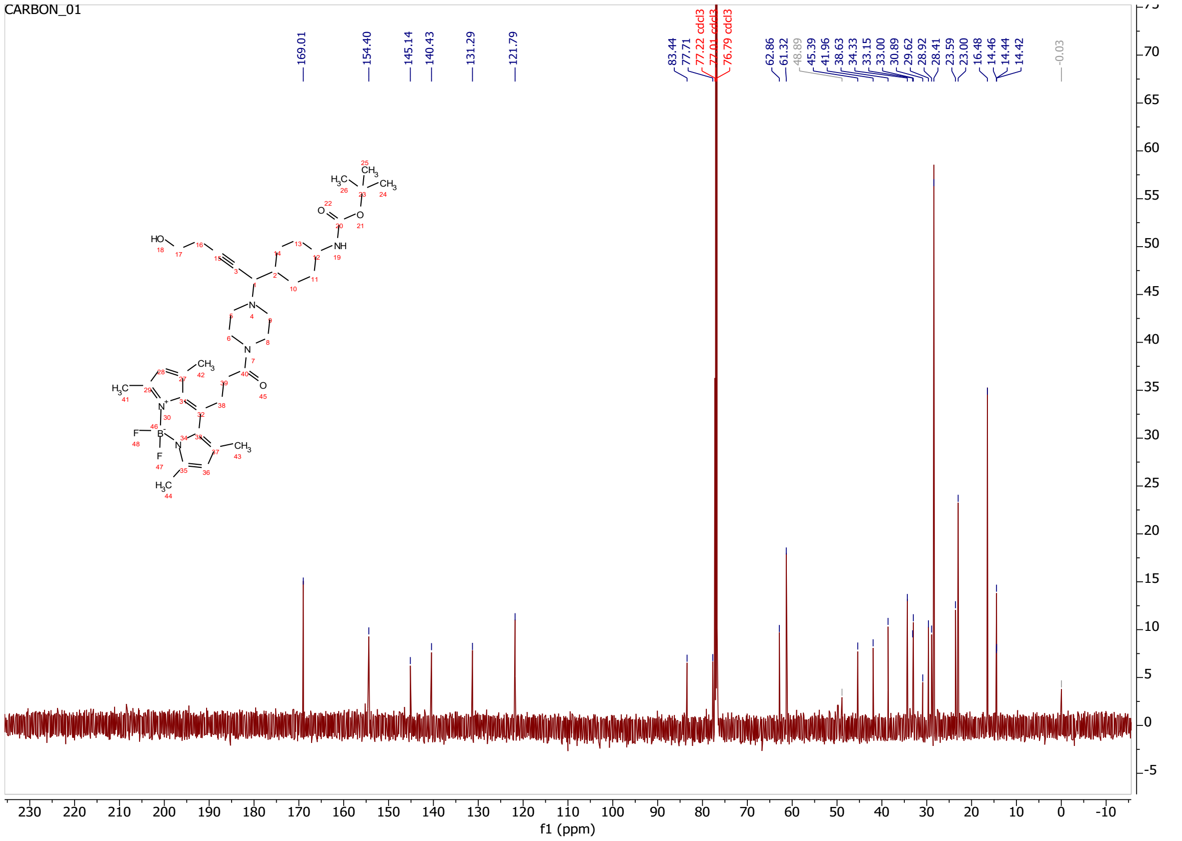

Figure S76 – ^{13}C spectra of compound **4a**.

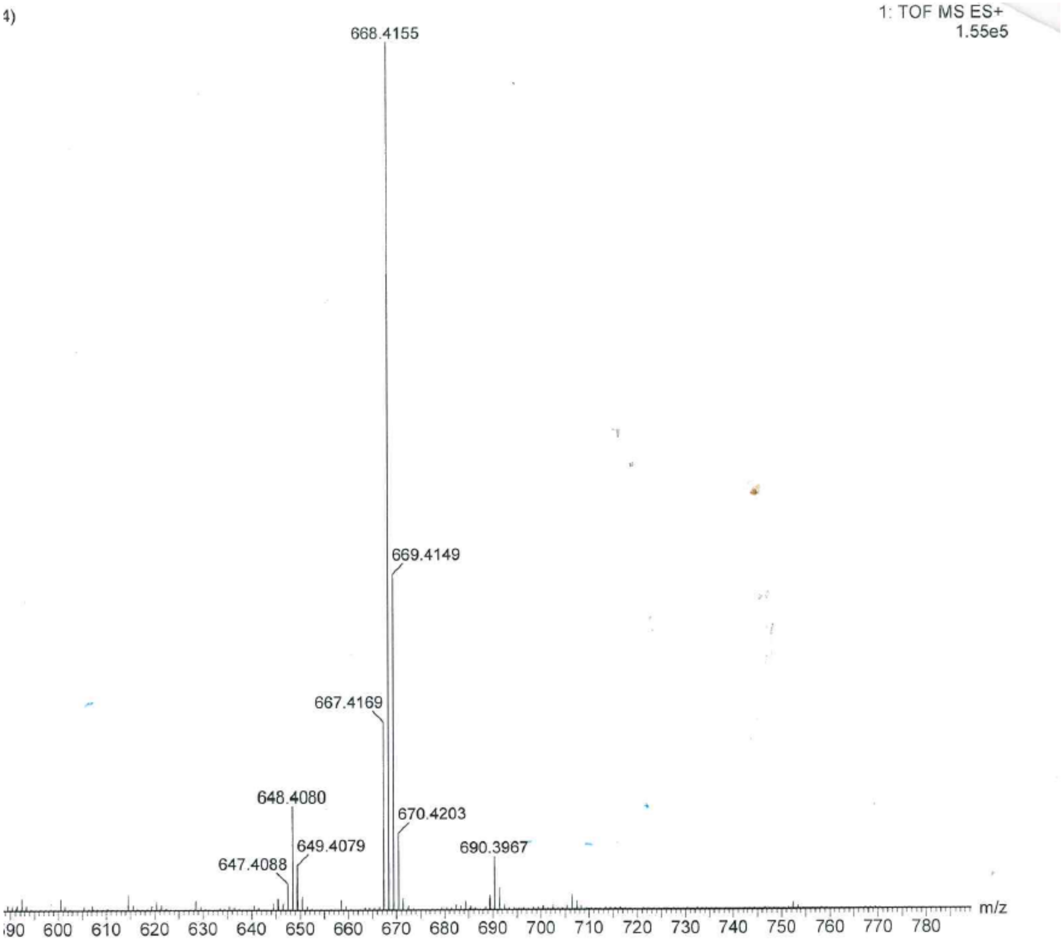

...ental Composition Report

Page 1

...ngle Mass Analysis

...olerance = 1000.0 PPM / DBE: min = -50.0, max = 80.0
Element prediction: Off
Number of isotope peaks used for i-FIT = 3

Monoisotopic Mass, Odd and Even Electron Ions
1 formula(e) evaluated with 1 results within limits (up to 50 closest results for each mass)
Elements Used:
C: 36-36 H: 0-100 B: 1-1 N: 5-5 O: 4-4 F: 2-2
239441
YAN_LIN002083B 13 (0.276) AM2 (Ar,20000.0,0.00,0.00); Cm (9:34)

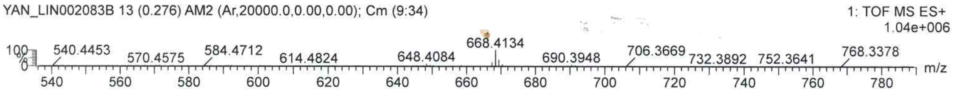

Minimum:				-50.0				
Maximum:		5.0	1000.0	80.0				
Mass	Calc. Mass	mDa	PPM	DBE	i-FIT	Norm	Conf(%)	Formula
668.4134	668.4159	-2.5	-3.7	12.5	370.4	n/a	n/a	C36 H53 B N5 O4 F2

Figure S77 – HRMS analysis of compound **4a**.

4b- N2268-48

10-(3-(4-(1-(4-aminocyclohexyl)-5-hydroxypent-2-yn-1-yl)piperazin-1-yl)-3-oxopropyl)-5,5-difluoro-1,3,7,9-tetramethyl-5H-dipyrrolo[1,2-c:2',1'-f][1,3,2]diazaborinin-4-ium-5-uide.

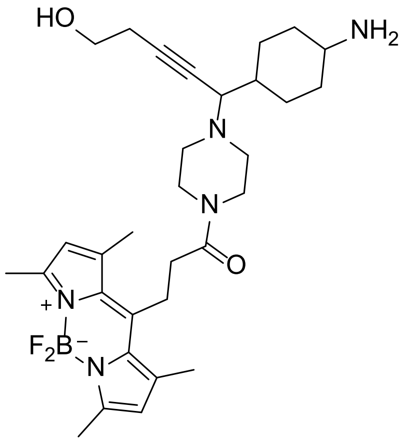

Chemical Formula: $C_{31}H_{44}BF_2N_5O_2$
Exact Mass: 567.3556
Molecular Weight: 567.5212

At 0 °C, a **4a** (40mg, 0.06mmol, 1.0eq) , 4M HCL in 1,4-dioxane (450µL, 1.8mmol, 30eq), and triethylsilane (0.02mL, 0.1mmol, 2.0eq) was stirred vigorously. After 1 hour the solution was treated with saturated $NaHCO_3$ (10mL) and extracted into DCM (2 x 10mL). The combined organics were dried over $MgSO_4$ and concentrated to residue which was purified by preparative HPLC (C_{18}, 20 x 150 mm, 5 µM) to yield **4b** as an red solid (73.9mg, 0.09 mmol, 70%) which was used without further purification or characterization in the synthesis of **4c**. LCMS RT 2.775 min, A% = 43%, [M+H] = 568.25. [M-H] = 566.20. Very low concentration sample leading to solvent fronting and an unsteady baseline accounting for reduced A% with regards to the major single peak observed.

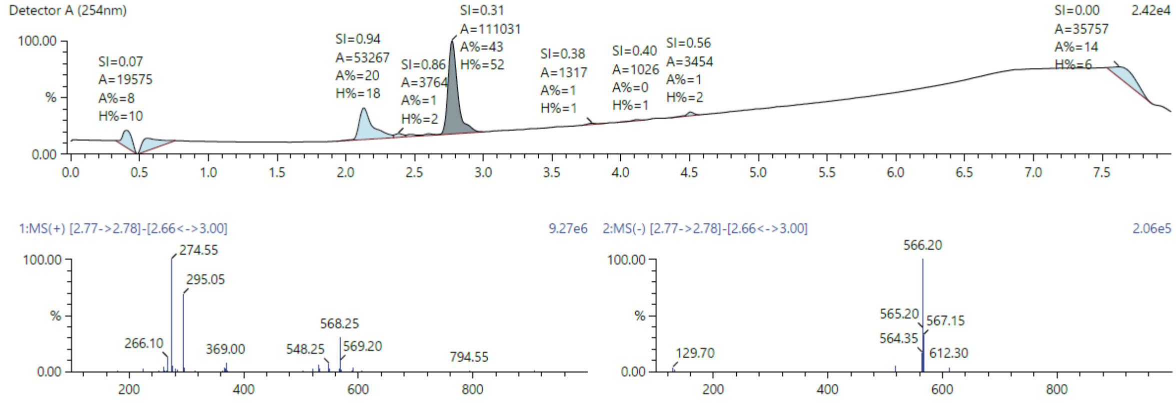

Figure S78 – LCMS analysis of compound **4b**.

4c – N2286-54

10-(3-(4-(1-(4-(3-(2-(2-(2-(2,6-dioxopiperidin-3-yl)-1,3-dioxoisoindoline-5-carboxamido)ethoxy)ethoxy)propanamido)cyclohexyl)-5-hydroxypent-2-yn-1-yl)piperazin-1-yl)-3-oxopropyl)-5,5-difluoro-1,3,7,9-tetramethyl-5H-dipyrrolo[1,2-c:2',1'-f][1,3,2]diazaborinin-4-ium-5-uide.

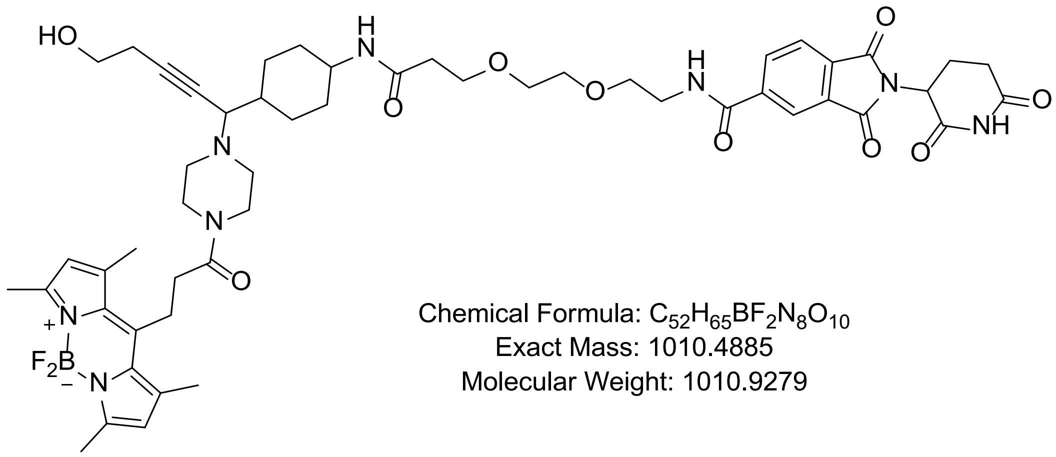

Chemical Formula: $C_{52}H_{65}BF_2N_8O_{10}$
Exact Mass: 1010.4885
Molecular Weight: 1010.9279

To a solution of 3-[2-[2-[[2-(2,6-dioxo-3-piperidyl)-1,3-dioxo-isoindoline-5-carbonyl]amino]ethoxy] ethoxy]propanoic acid (*Broadpharm, Thalidomide-5-(PEG2-acid)*) (60.0 mg, 0.13mmol, 1.0eq), HATU (99.0mg, 0.26mmol, 2.0eq), and N,N-diisopropylethylamine (91µL, 0.52mmol, 4.0eq) in N,N-dimethylformamide (1mL), **4b** (73.8mg, 0.13mmol, 1.0eq) was added. The resulting mixture was stirred for 12 hours at room temperature. Upon completion the reaction mixture was diluted with DCM (5mL) and the resulting organics were washed with water (3 x 5mL) and brine (5mL), dried over $MgSO_4$, and concentrated to residue. The impure residue was purified by column chromatography (SiO_2, 12g, DCM/MeOH) allowing the isolation of **4c** as an orange solid (67mg, 0.06 mmol, 48%). ^{1}H NMR (600 MHz, Chloroform-*d*) δ 9.01 (d, *J* = 8.0 Hz, 1H), 8.32 – 8.30 (m, 2H), 7.91 (d, *J* = 8.0 Hz, 1H), 7.76 (m, 1H), 6.04 (s, 2H), 4.98 (dd, *J* = 12.5, 5.5 Hz, 1H), 3.79 – 3.74 (m, 2H), 3.69 – 3.66 (m, 6H), 3.64 – 3.62 (m, 5H), 3.54 (s, 1H), 3.37 (t, *J* = 5.0 Hz, 2H), 3.35 – 3.30 (m, 2H), 2.90 – 2.83 (m, 2H), 2.84 – 2.70 (m, 2H), 2.61 – 2.55 (m, 2H), 2.52 (d, *J* = 8.0 Hz, 1H), 2.50 (s, 6H), 2.45 (m, 2H), 2.40 (t, *J* = 6.0 Hz, 2H), 2.38 (s, 6H), 2.37 – 2.28 (m, 6H), 2.17 – 2.13 (m, 1H), 2.01 – 1.95 (m, 2H), 1.95 – 1.90 (m, 2H), 1.38 – 1.33 (m, 1H), 1.09 – 1.02 (m, 1H), 1.01 – 0.97 (m, 2H), 0.92 – 0.89 (m, 1H). ^{13}C NMR (151 MHz, Chloroform-*d*) δ 171.2, 170.5, 169.1, 168.3, 166.8 (2C), 166.5, 165.4, 165.3, 154.4, 145.1, 140.6 (J_{CF4} = 3.5 Hz), 140.5, 134.3, 133.5, 131.8, 131.3, 124.0, 122.3, 121.8 (2C), 83.7, 77.5, 70.3, 70.1 (2C), 69.5, 67.2, 62.7, 61.3, 49.5 (2C), 48.6 (2C), 45.4, 41.9, 40.2, 38.6, 37.2, 34.3, 32.6, 32.5, 31.4, 29.5, 23.6, 23.0 (2C), 22.5, 16.5 (2C), 14.4 (2C, J_{CF4} = 3.5 Hz). ^{19}F NMR (376 MHz, Chloroform-*d*) δ -146.17 – -146.94. HRMS $C_{52}H_{66}BN_8O_{10}F_2$, Calculated [M+H] = 1011.4963. Experimental [M+H] = 1011.4984 (ppm = +2.1). LCMS RT 3.425 min, A% = 81%*, Mw [(M+H)/2] = 506.30. Very low concentration sample leading to solvent fronting and an unsteady baseline accounting for reduced A% with regards to the major single peak observed.

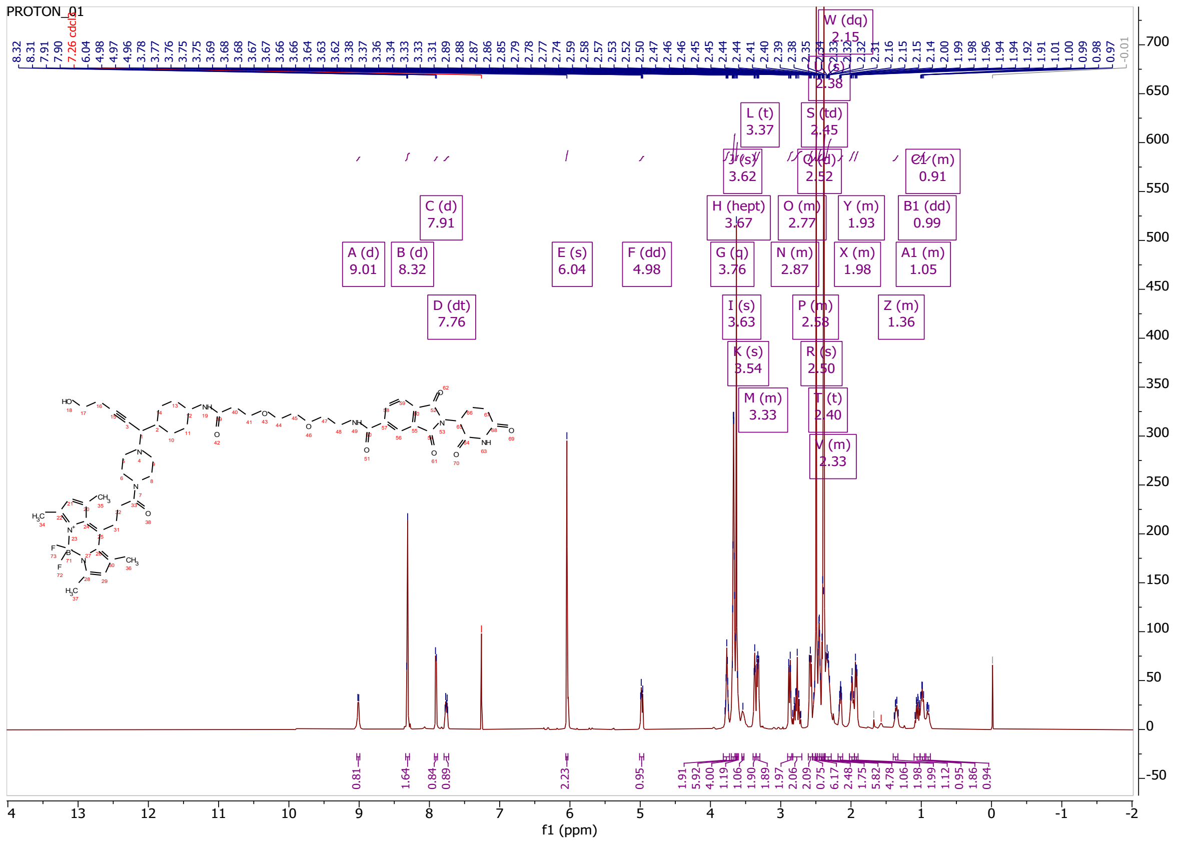

Figure S79 – ^{1}H NMR spectra of compound **4c.**

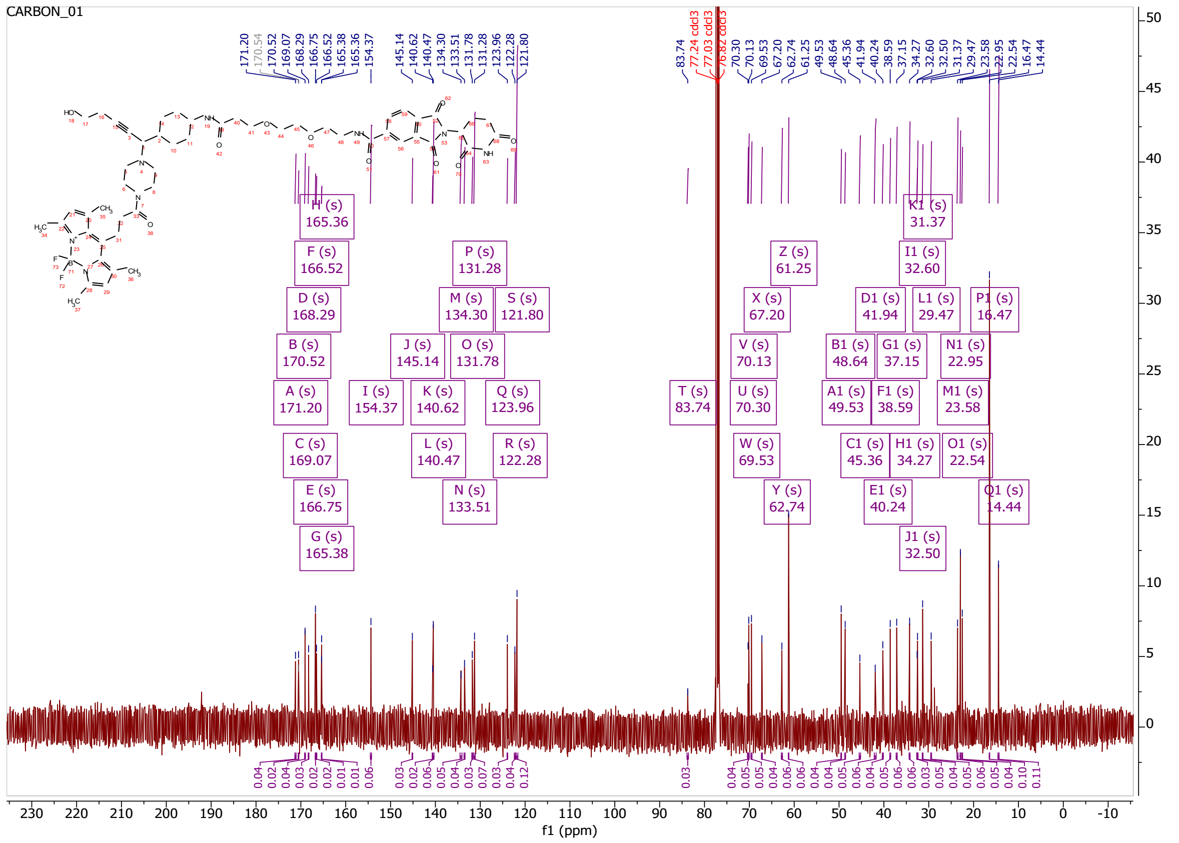

Figure S80 – ^{13}C NMR spectra of compound **4c**.

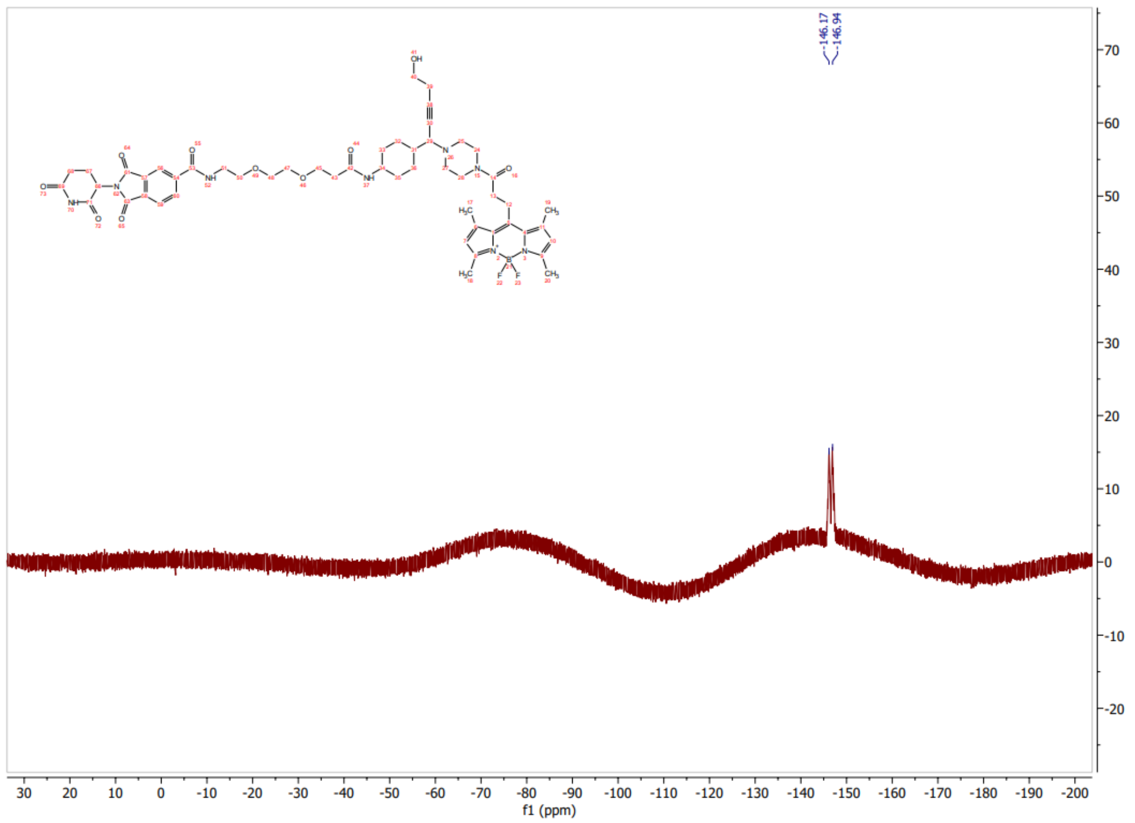

Figure S81 – ^{19}F NMR spectra of compound **4c**.

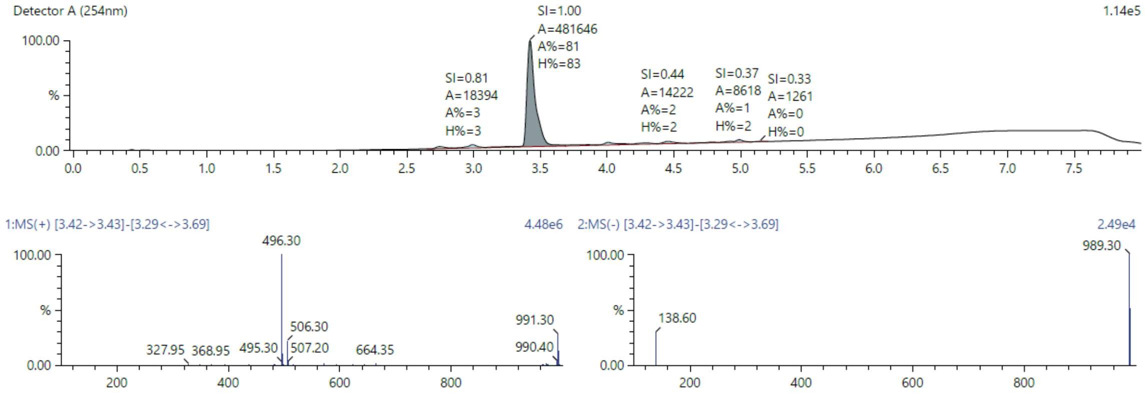

Figure S82 – LCMS analysis of compound **4c**.

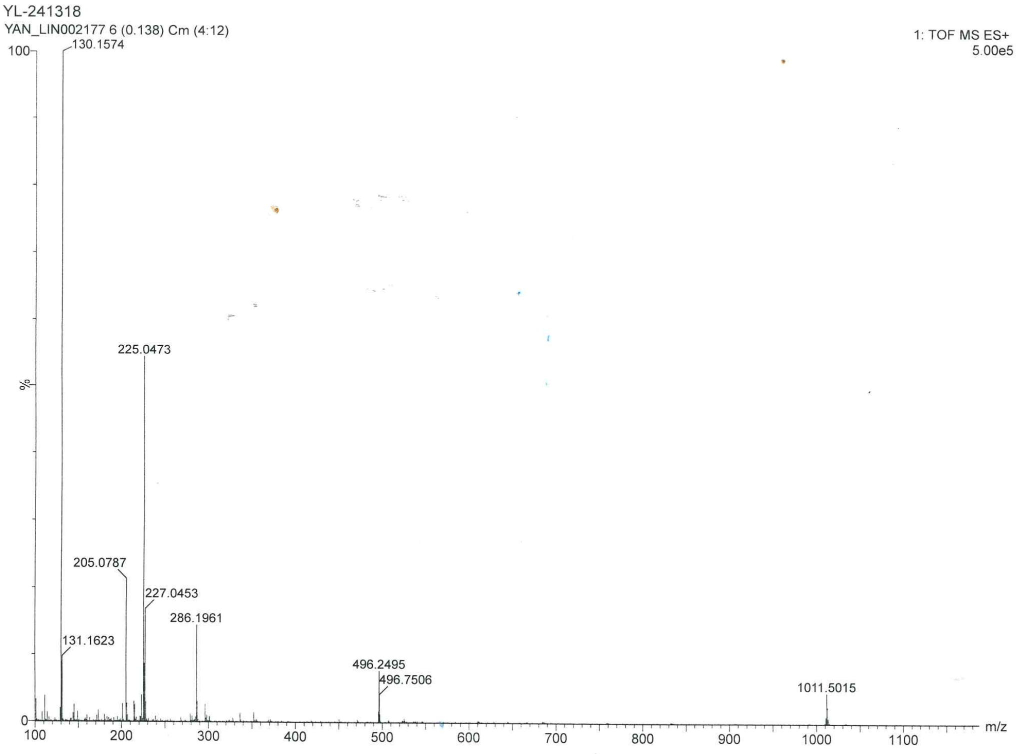

Elemental Composition Report

Page 1

Single Mass Analysis

Tolerance = 1000.0 PPM / DBE: min = -50.0, max = 80.0
Element prediction: Off
Number of isotope peaks used for i-FIT = 3

Monoisotopic Mass, Odd and Even Electron Ions
2 formula(e) evaluated with 1 results within limits (up to 50 closest results for each mass)
Elements Used:
C: 52-52 H: 0-100 B: 1-1 N: 8-8 O: 10-10 F: 2-2 151Eu: 0-1

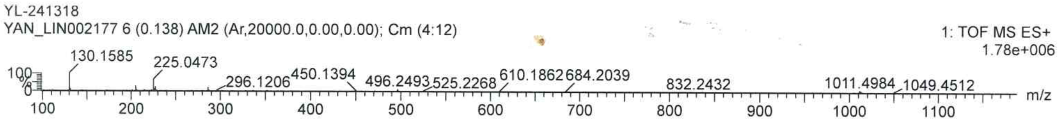

Minimum:				-50.0				
Maximum:		5.0	1000.0	80.0				
Mass	Calc. Mass	mDa	PPM	DBE	i-FIT	Norm	Conf(%)	Formula
1011.4984	1011.4963	2.1	2.1	23.5	265.5	n/a	n/a	C52 H66 B N8 O10 F2

Figure S83 – HRMS analysis of compound **4c**.

Alkylation of bromo-A3 core protocol.

Bromo-A3 core (1.2 mmol, 1.0 eq), thiol (1.4mmol, 1.2eq) and K_2CO_3 (1.76mmol, 1.5eq) were suspended in acetonitrile (10mL) and heated to reflux for 16 hours. The reaction was monitored by TLC and, upon completion, the mixture was cooled to room temperature and diluted with ethyl acetate (20mL). The organics were washed with aq. NaOH (1N, 2 x 15mL), brine (15mL), dried over $MgSO_4$ and concentrated to residue. This residue was purified by column chromatography (SiO_2, 12 g, DCM: MeOH).

5a AMPRO-186

tert-butyl (1-(5-((2-aminoethyl)thio)-1-(tetrahydro-2H-pyran-4-yl)pent-2-yn-1-yl)piperidin-4-yl)carbamate.

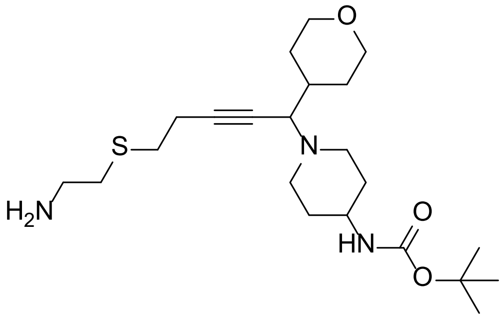

Chemical Formula: $C_{22}H_{39}N_3O_3S$
Exact Mass: 425.2712
Molecular Weight: 425.6284

2d (250mg, 0.59mmol, 1.0 eq), cysteamine (54mg, 0.70mmol, 1.2 eq) and potassium carbonate (123mg, 0.88mmol, 1.50 eq) were suspended in MeCN (10 mL) and heated to 80°C for 12 hours. Upon completion the reaction mixture was cooled to room temperature, diluted with EtOAc (15mL), and washed with aq. NaOH (1N, 20mL). The aqueous was re-extracted with EtOAc (10mL) and the combined organics were washed with water (10mL) and brine (10mL) before being dried over $MgSO_4$ and concentrated to residue.

The material was columned to purify (SiO_2, 12g, DCM: MeOH (modified with 0.1% NEt_3)) with **5a** being isolated as a white solid (183mg, 0.43mmol, 77%).

^{1}H NMR (600 MHz, Chloroform-*d*) δ 4.49 (s, 1H), 3.97 (m, 2H), 3.45 (s, 1H), 3.38 - 3.29 (m, 2H), 2.92 (t, *J* = 5 Hz, 3H), 2.68 -2.65 (m, 4H), 2.55 – 2.47 (m, 4H), 2.23 (t, *J* = 10.5 Hz, 1H), 2.02 – 1.99 (m, 4H), 1.92 - 1.85 (m, 3H), 1.79 (d, *J* = 13.5 Hz, 1H), 1.68 (d, *J* = 11.5 Hz, 1H), 1.43 (s, 9H), 1.34 - 1.20 (m, 3H). ^{13}C NMR (151 MHz, Chloroform-*d*) δ 155.2, 84.7, 79.2, 77.3, 67.9, 67.8, 62.9, 47.9, 45.4, 37.2 (2C), 32.9, 32.6, 31.4, 31.1 (2C), 30.6 (2C), 28.4 (3C), 20.2. HRMS $C_{22}H_{39}N_3O_3S$ Calculated [M+H] = 426.2790. Experimental [M+H] = 426.2818 (ppm = + 0.2).

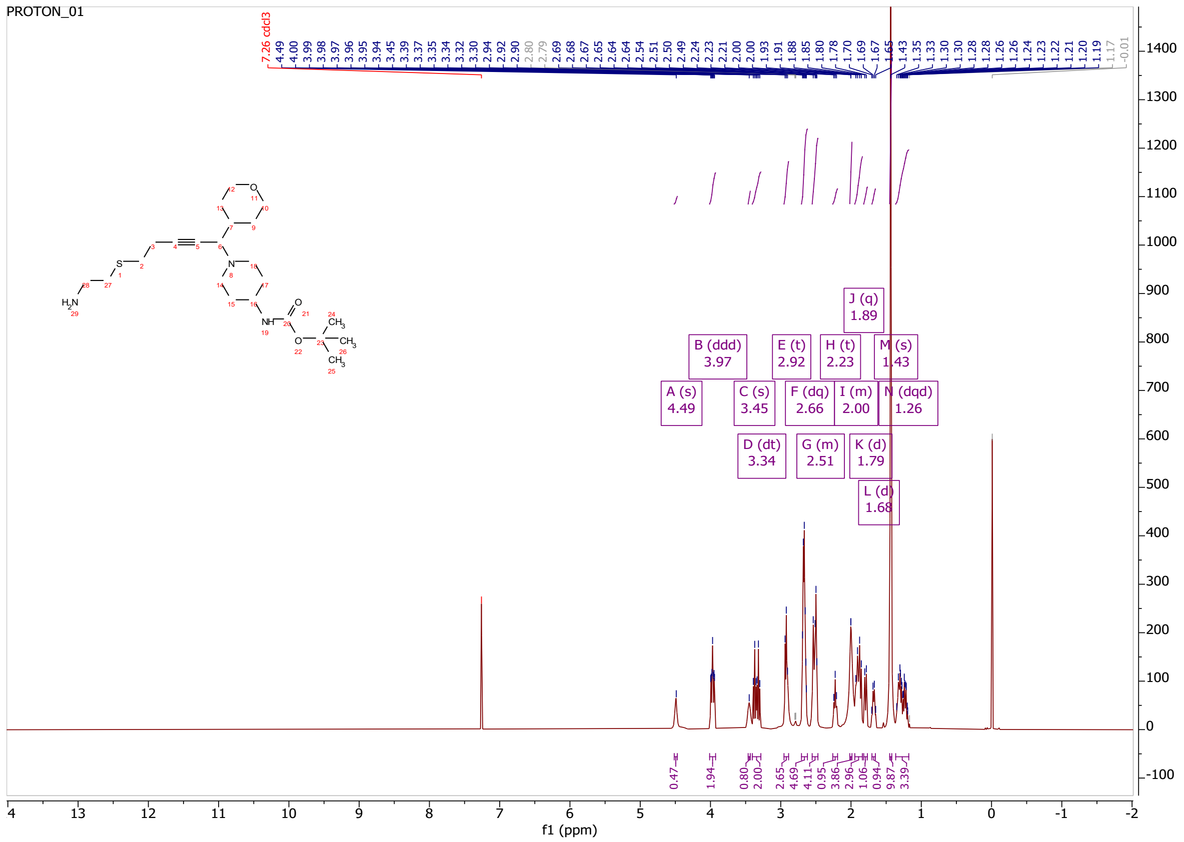

Figure S84 – ^{1}H spectra of compound **5a**.

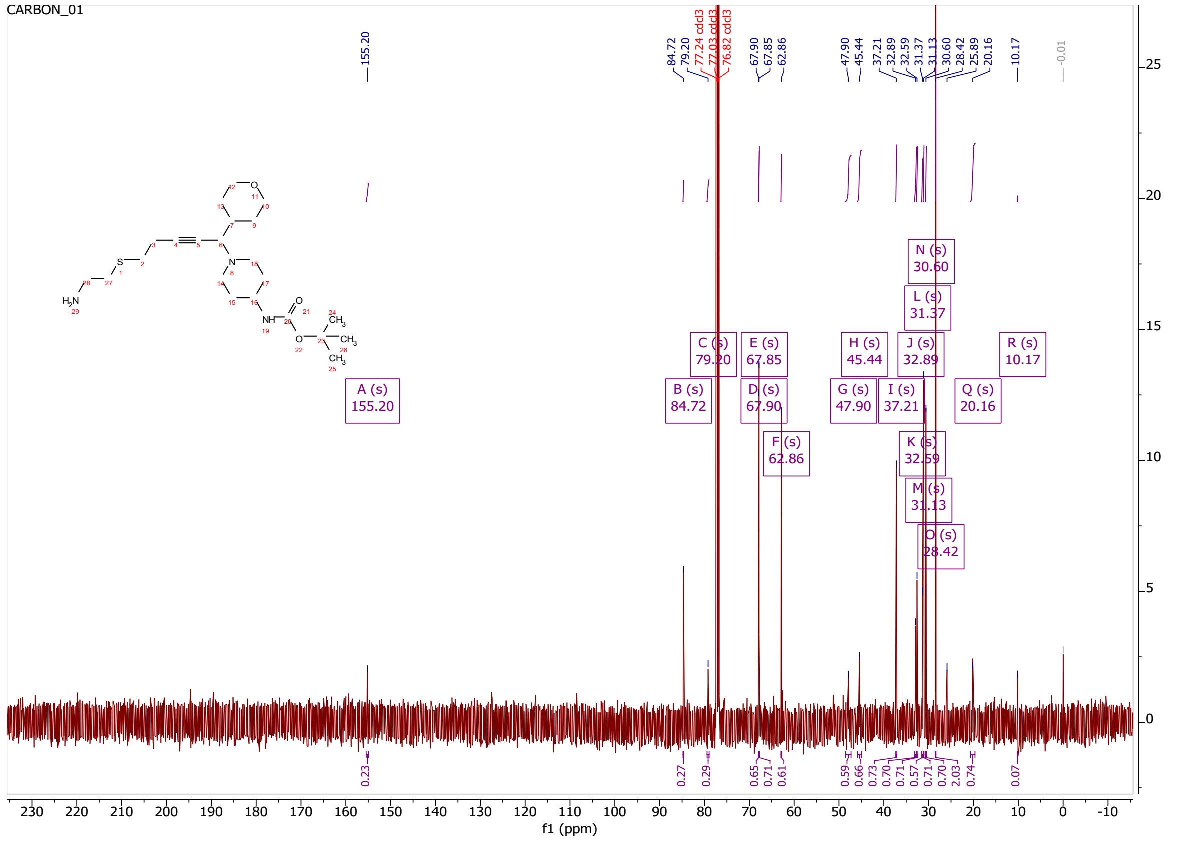

Figure S85 – ^{13}C spectra of compound **5a**.

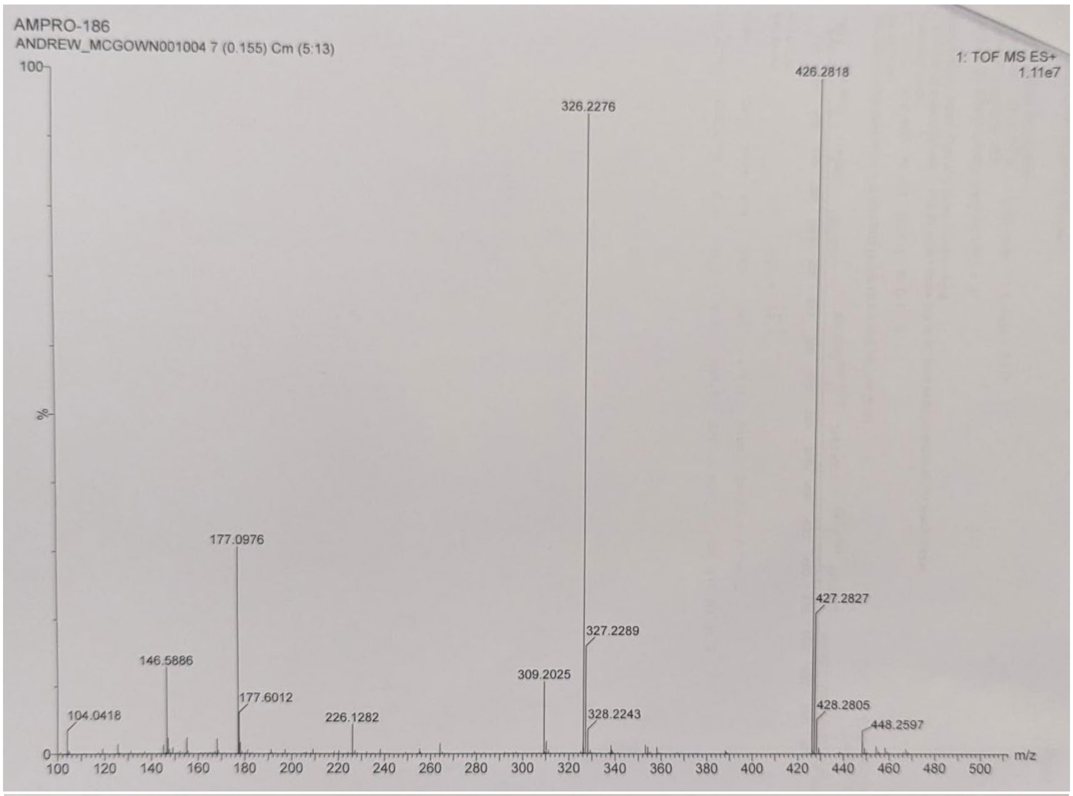

[illegible]ental Composition Report **Page 1**

[illegible]ingle Mass Analysis
Tolerance = 100.0 PPM / DBE: min = -1.5, max = 50.0
Element prediction: Off
Number of isotope peaks used for i-FIT = 3

Monoisotopic Mass, Odd and Even Electron Ions
2 formula(e) evaluated with 1 results within limits (up to 50 best isotopic matches for each mass)
Elements Used:
C: 22-22 H: 0-100 N: 3-3 O: 3-3 P: 0-1 S: 1-1
AMPRO-186
ANDREW_MCGOWN001004 7 (0.155) AM2 (Ar,20000.0,0.00,0.00); Cm (5:13) 1: TOF MS ES+
3.29e+007

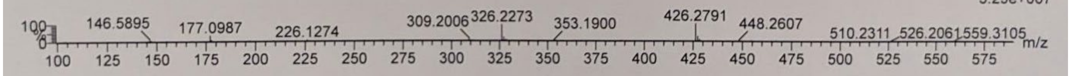

Minimum:				-1.5				
Maximum:		5.0	100.0	50.0				
Mass	Calc. Mass	mDa	PPM	DBE	i-FIT	Norm	Conf(%)	Formula
426.2791	426.2790	0.1	0.2	4.5	632.6	n/a	n/a	C22 H40 N3 O3 S

Figure S86 – HRMS analysis of compound **5a**.

5b AMPRO-222

4-((5-(4-((tert-butoxycarbonyl)amino)piperidin-1-yl)-5-(tetrahydro-2H-pyran-4-yl)pent-3-yn-1-yl)thio)benzoic acid.

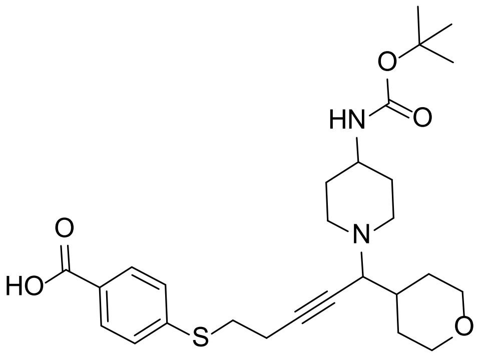

Chemical Formula: $C_{27}H_{38}N_2O_5S$
Exact Mass: 502.2501
Molecular Weight: 502.6660

Using the reported method **5b** was isolated as a pale yellow solid (558mg, 1.11mmol, 95%). ^{1}H NMR (600 MHz, Chloroform-*d*) δ 9.16 (s, 1H), 8.26 (s, 2H), 7.98 (d, *J* = 8.0 Hz, 2H), 7.34 (d, *J* = 8.0 Hz, 2H), 4.01 – 3.94 (m, 2H), 3.64 (s, 1H), 3.40 - 3.30 (m, 2H), 3.25 – 3.22 (m, 2H), 3.16 (t, *J* = 7.0 Hz, 3H), 2.65 (d, *J* = 7.0 Hz, 2H), 2.06 – 2.0 (m, 2H), 1.96 – 1.94 (m, 1H), 1.86 (d, *J* = 12.5 Hz, 2H), 1.82 – 1.75 (m, 3H), 1.51 – 1.46 (m, 2H), 1.43 (s, 9H). ^{13}C NMR (151 MHz, Chloroform-*d*) δ 169.9, 166.0, 142.6, 130.6 (2C), 127.7, 127.2 (2C), 88.6, 79.8, 73.8, 67.3 (2C), 62.9, 46.1 (2C), 36.8 (2C), 31.4, 30.4, 30.1, 29.9, 29.8, 28.4 (3C), 19.3. HRMS $C_{27}H_{38}N_2O_5S$ Calculated [M+H] = 503.2580. Experimental [M+H] = 503.2395 (ppm = -36.8).

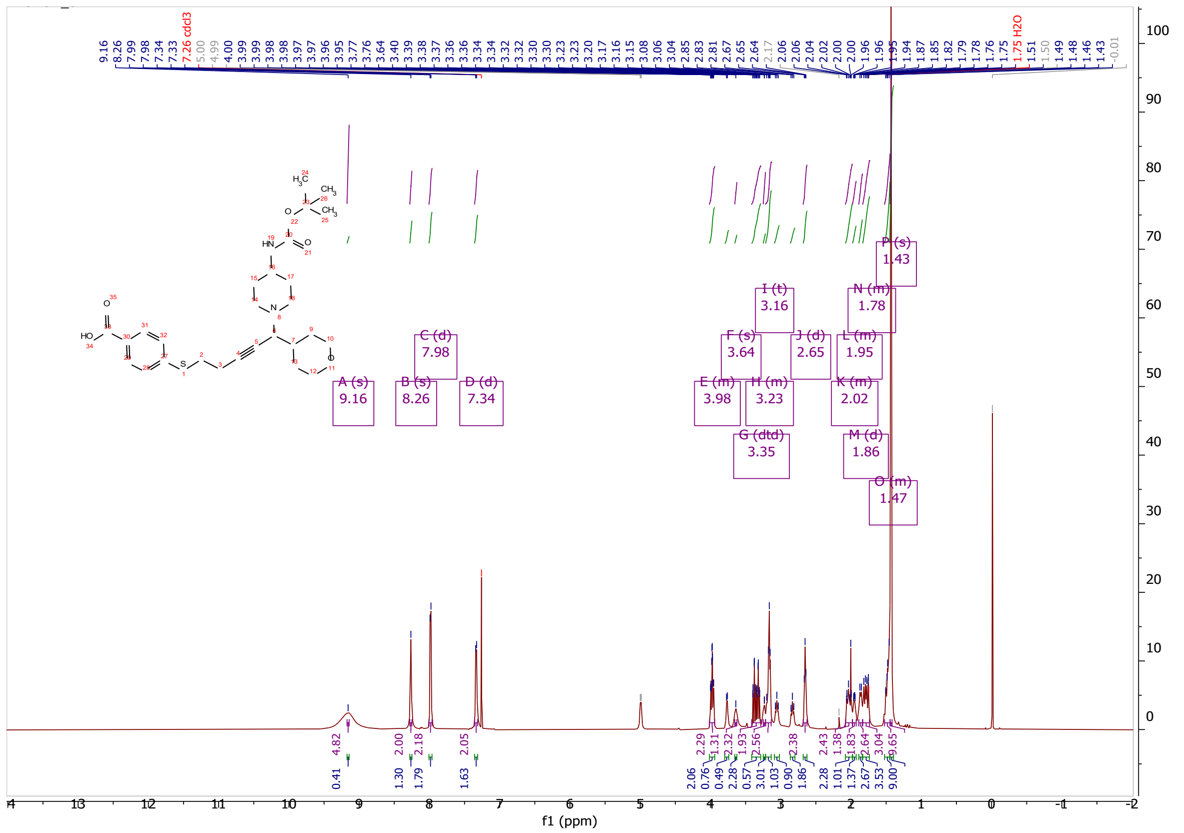

Figure S87 – ^{1}H spectra of compound **5b**.

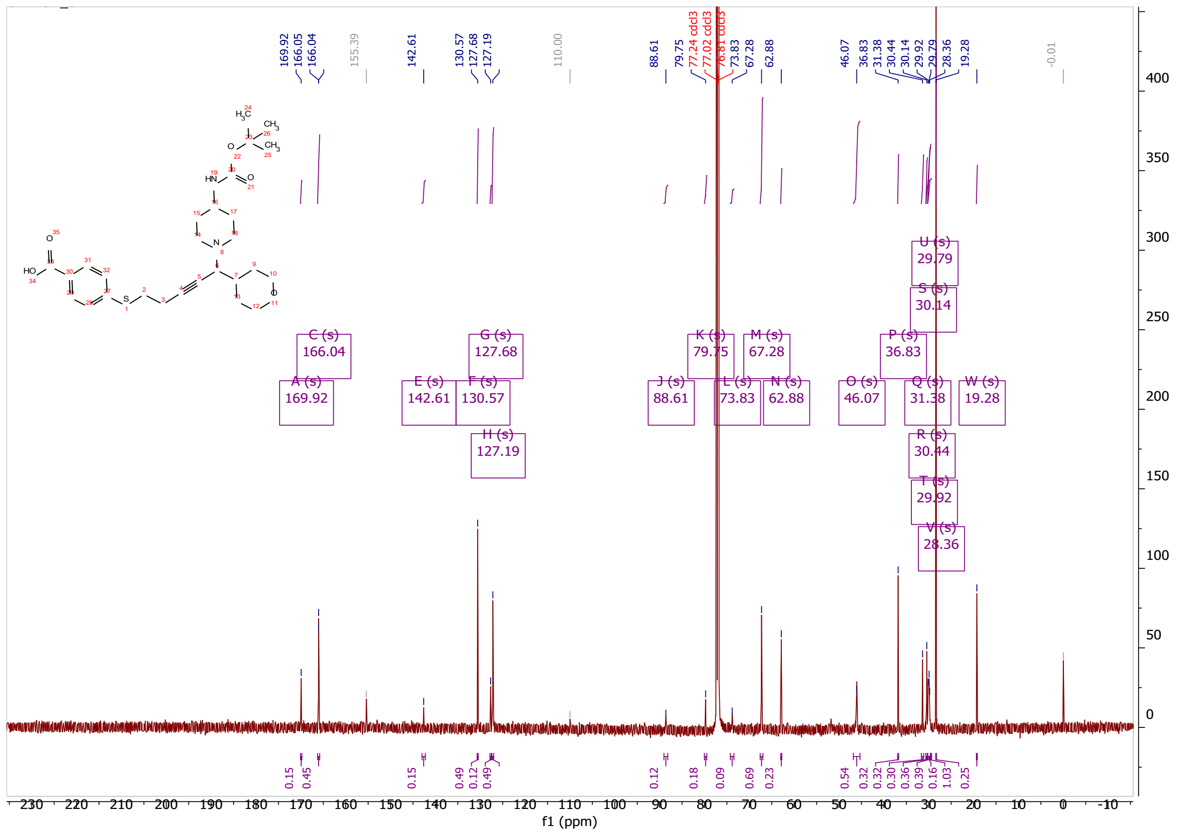

Figure S88 – ^{13}H spectra of compound **5b**.

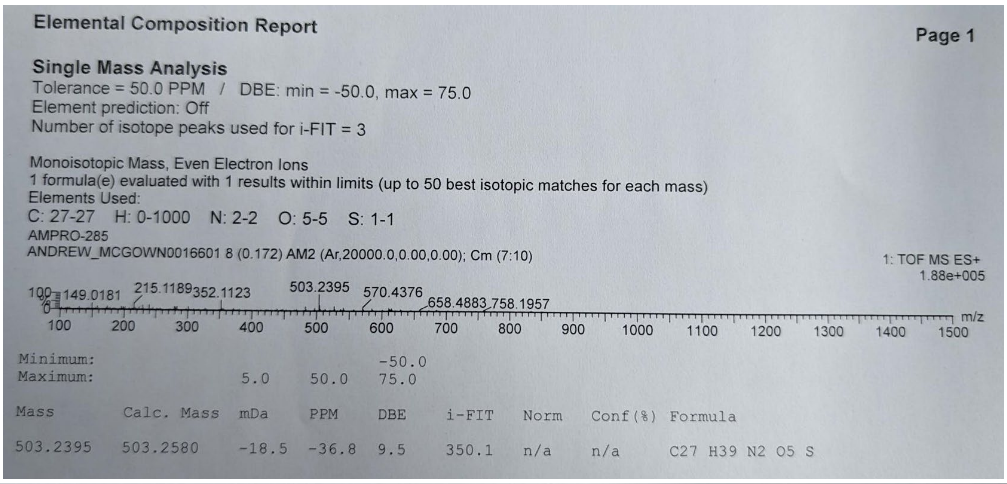

Elemental Composition Report **Page 1**

Single Mass Analysis
Tolerance = 50.0 PPM / DBE: min = -50.0, max = 75.0
Element prediction: Off
Number of isotope peaks used for i-FIT = 3

Monoisotopic Mass, Even Electron Ions
1 formula(e) evaluated with 1 results within limits (up to 50 best isotopic matches for each mass)
Elements Used:
C: 27-27 H: 0-1000 N: 2-2 O: 5-5 S: 1-1
AMPRO-285
ANDREW_MCGOWN0016601 8 (0.172) AM2 (Ar,20000.0,0.00,0.00); Cm (7:10)

1: TOF MS ES+
1.88e+005

Minimum:				-50.0				
Maximum:		5.0	50.0	75.0				
Mass	Calc. Mass	mDa	PPM	DBE	i-FIT	Norm	Conf(%)	Formula
503.2395	503.2580	-18.5	-36.8	9.5	350.1	n/a	n/a	C27 H39 N2 O5 S

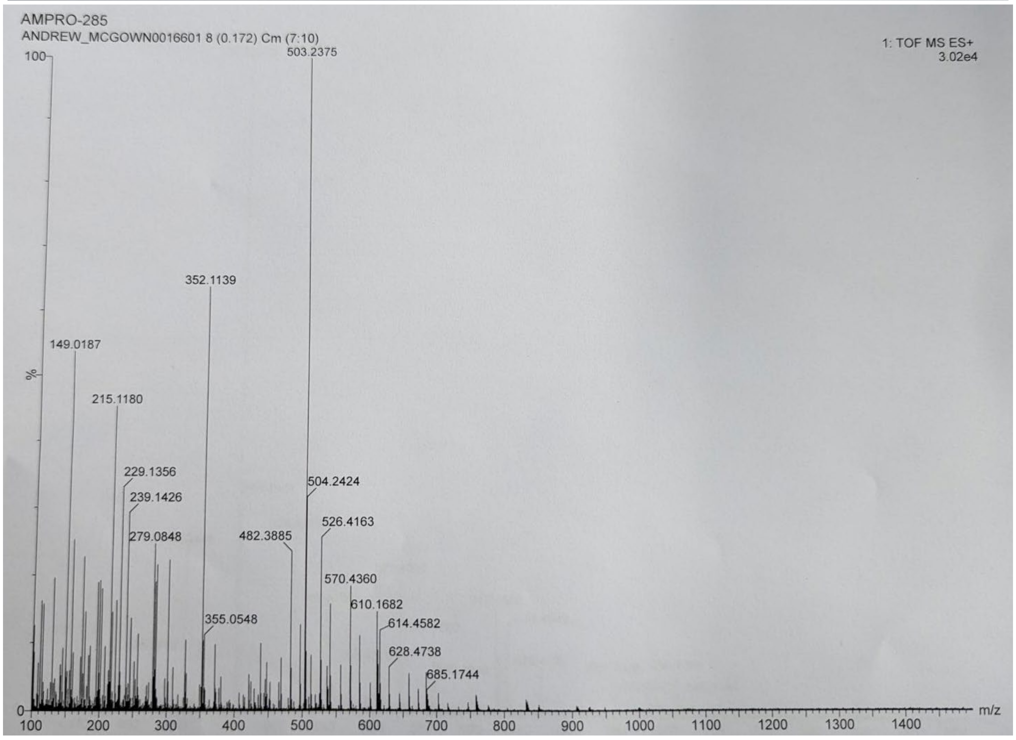

Figure S89 – HRMS analysis of compound **5b**.

6a AMPRO-226

tert-butyl (1-(5-((4-((2-(2-((S)-4-(4-chlorophenyl)-2,3,9-trimethyl-6H-thieno[3,2-f][1,2,4]triazolo[4,3-a][1,4]diazepin-6-yl)acetamido)ethyl)carbamoyl)phenyl)thio)-1-(tetrahydro-2H-pyran-4-yl)pent-2-yn-1-yl)piperidin-4-yl)carbamate.

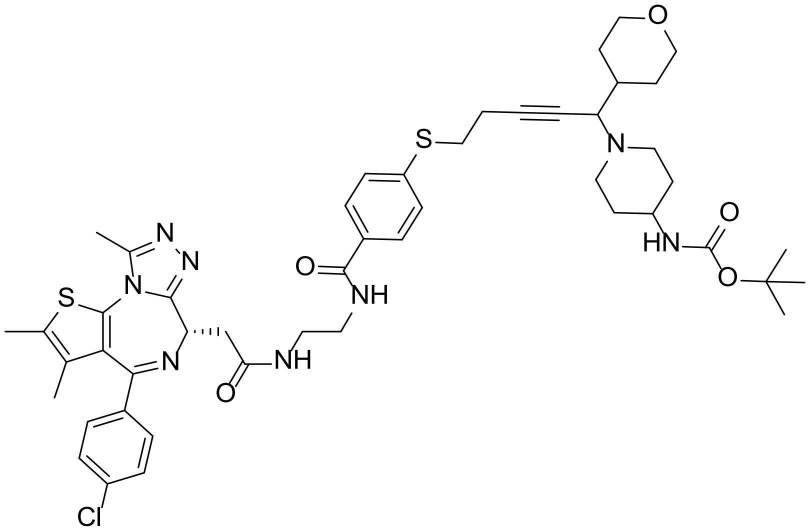

Chemical Formula: $C_{48}H_{59}ClN_8O_5S_2$
Exact Mass: 926.3738
Molecular Weight: 927.6157

5b (57mg, 0.11mmol, 1.0 eq) and HATU (65mg, 0.17mmol, 1.5 eq) were dissolved in a mixture of anhydrous DMF (2mL) and DIPEA (57mg, 77µL, 0.44mmol, 4.0 eq). Upon dissolution **1f** (50mg, 0.11mmol, 1.0 eq) was added and the reaction mixture was stirred at room temperature for 48 hours.

The reaction mixture was monitored by LCMS and upon completion it was diluted with DCM (5mL) and washed with water (5mL) and brine (5mL), dried over $MgSO_4$ and concentrated to residue. This residue was taken on to deprotection without further purification.

LCMS RT 5.293 min, A% = 86 %*, Mw [M+H] = 927.35, [M-H] = 925.20. *Single peak.

Analytical LC-UV/MS Report

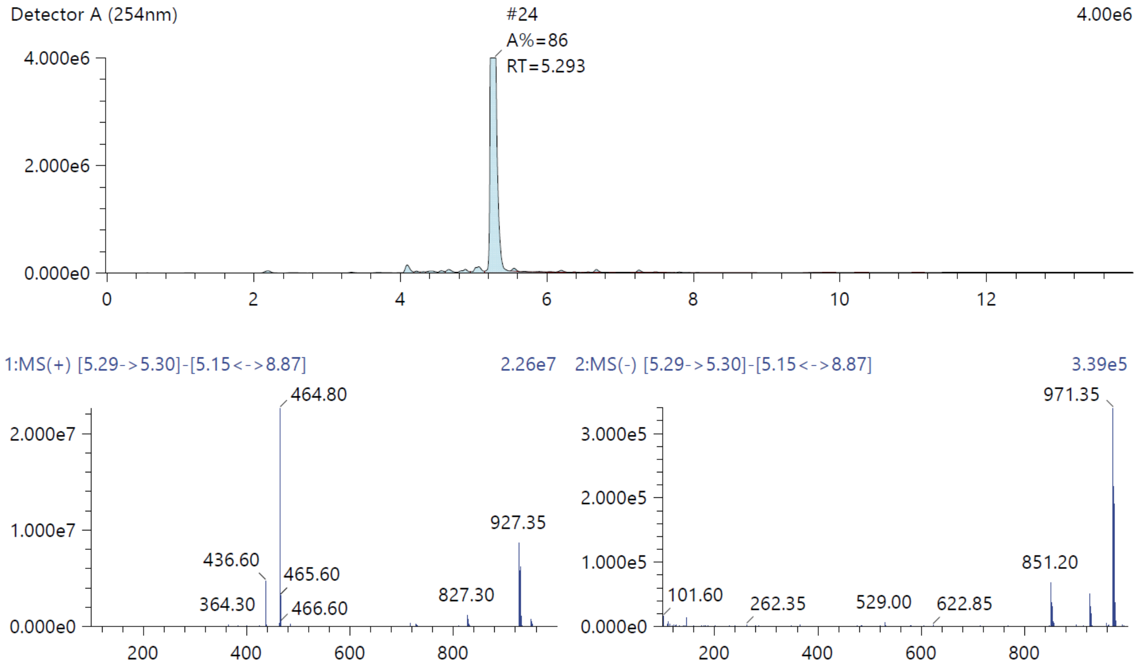

Figure S90 – LCMS analysis of compound **6a**.

6b AMPRO-232

4-((5-(4-aminopiperidin-1-yl)-5-(tetrahydro-2H-pyran-4-yl)pent-3-yn-1-yl)thio)-N-(2-(2-((S)-4-(4-chlorophenyl)-2,3,9-trimethyl-6H-thieno[3,2-f][1,2,4]triazolo[4,3-a][1,4]diazepin-6-yl)acetamido) ethyl)benzamide.

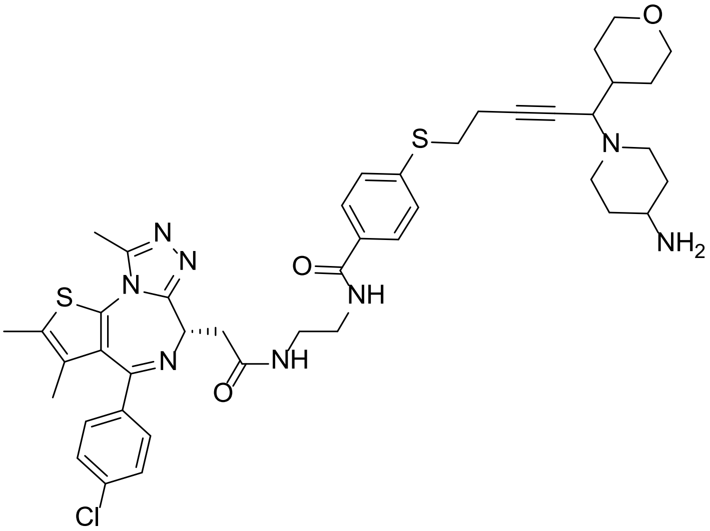

Chemical Formula: $C_{43}H_{51}ClN_8O_3S_2$
Exact Mass: 826.3214
Molecular Weight: 827.4998

6a (102mg, 0.20mmol, 1.0 eq) was dissolved in DCM (2mL) and treated with 4N HCl in 1,4-dioxane (1mL) and stirred at room temperature for 12 hours. Upon completion the reaction mixture was concentrated to residue and passed through a Isolute Si-SCX-2 column to yield **6b** as a white solid (160mg, 0.17mmol, 86%). ^{1}H NMR (600 MHz, DMSO-d_6) δ 8.51 (t, J = 5.5 Hz, 1H), 8.41 (t, J = 5.5 Hz, 1H), 8.05 (s, 2H), 7.77 (d, J = 8.5 Hz, 2H), 7.43 (d, J = 8.5 Hz, 2H), 7.37 (d, J = 8.0 Hz, 2H), 7.35 (d, J = 8.0 Hz, 2H), 4.49 (dd, J = 8.0, 6.0 Hz, 1H), 3.84 – 3.76 (m, 2H), 3.33 – 3.23 (m, 4H), 3.23 – 3.13 (m, 6H), 2.98 (dt, J = 10.5, 2.0 Hz, 1H), 2.89 (m, 1H), 2.63 (d, J = 11.0 Hz, 1H), 2.58 (m, 1H), 2.57 (s, 3H), 2.54 (m, 2H), 2.39 (s, 3H), 2.36 (dd, J = 11.9, 2.5 Hz, 1H), 2.11 – 2.03 (m, 1H), 1.88 – 1.81 (m, 2H), 1.73 (d, J = 13.5 Hz, 1H), 1.67 – 1.59 (m, 2H), 1.57 (s, 3H), 1.48 (m, 1H), 1.39 (m, 1H), 1.12 (m, 1H), 1.08 – 1.00 (m, 1H). ^{13}C NMR (151 MHz, DMSO-d_6) δ 170.5, 166.2, 163.5, 155.5, 150.3, 140.5, 137.2, 135.6, 134.9, 132.7, 131.8, 131.1, 130.6 (2C), 130.3, 130.0, 128.9, 128.3 (2C), 127.4, 127.2, 118.9, 84.9, 78.1, 67.2, 67.1, 62.2, 54.2, 51.3, 49.0, 48.5, 44.2, 38.7, 38.1, 37.0, 31.5, 31.2, 30.8, 30.6, 19.1, 14.5, 13.1, 11.8. HRMS $C_{43}H_{51}ClN_8O_3S_2$ Calculated [M+H] = 827.3292. Experimental [M+H] = 827.3266 (ppm = + 0.2). LCMS RT 4.018 min, A% = 91 % - single peak, Mw [M+H] = 827.30, [M-H] = 825.30.

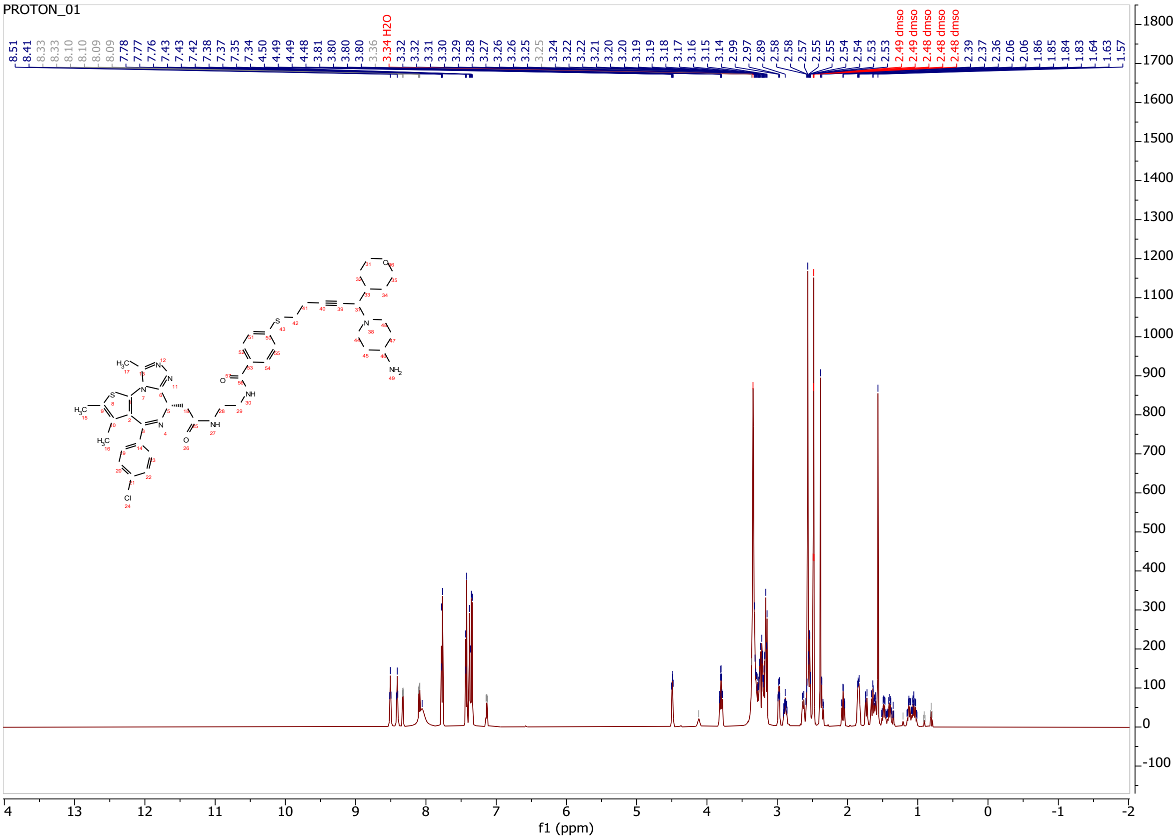

Figure S91 – ^{1}H spectra for compound **6b**.

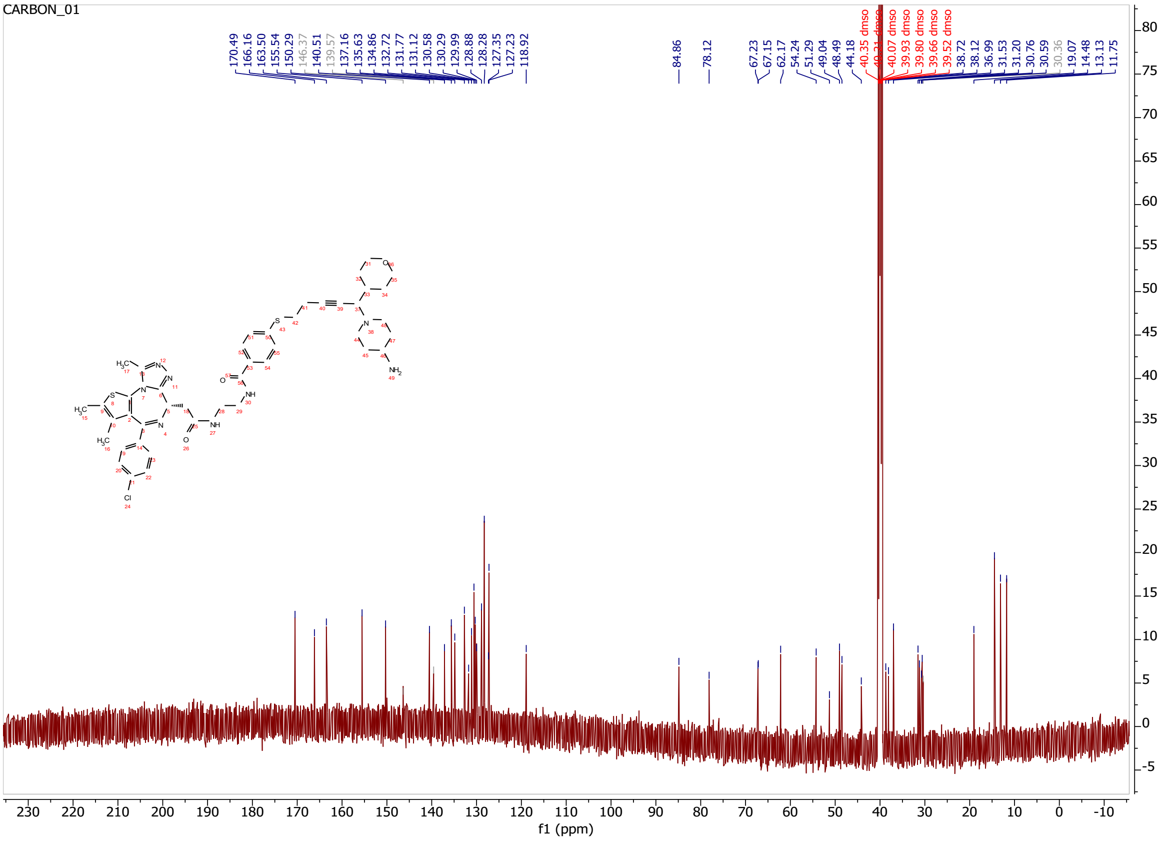

Figure S92 – ^{13}C spectra for compound **6b**.

Analytical LC-UV/MS Report

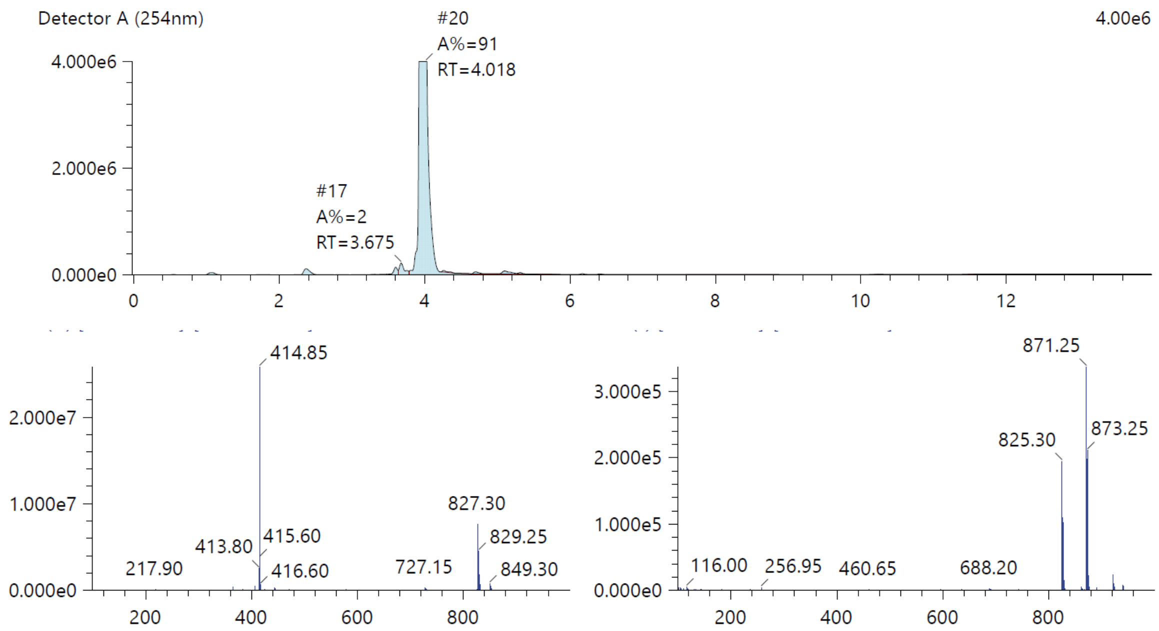

Figure S93 – LCMS analysis of compound **6b**

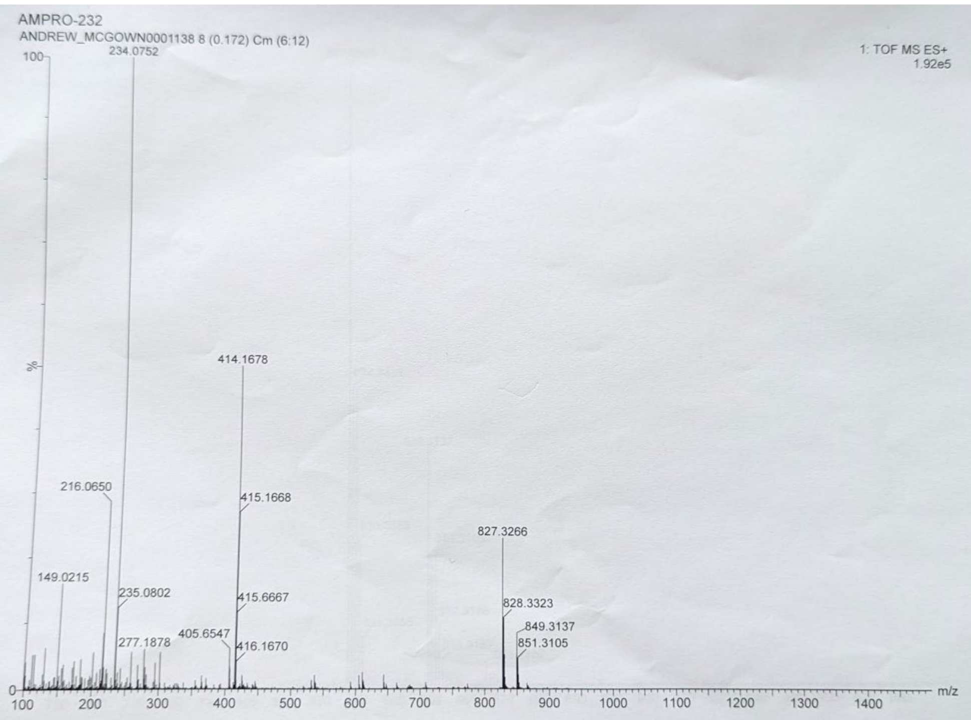

Elemental Composition Report

Page 1

Single Mass Analysis

Tolerance = 100.0 PPM / DBE: min = -50.0, max = 100.0
Element prediction: Off
Number of isotope peaks used for i-FIT = 3

Monoisotopic Mass, Even Electron Ions
2 formula(e) evaluated with 1 results within limits (up to 50 closest results for each mass)
Elements Used:
C: 43-43 H: 0-100 N: 8-8 O: 3-3 P: 0-1 S: 2-2 Cl: 1-1
AMPRO-232
ANDREW_MCGOWN0001138 8 (0.172) AM2 (Ar,20000.0,0.00,0.00); Cm (8:13)

1: TOF MS ES+
4.68e+005

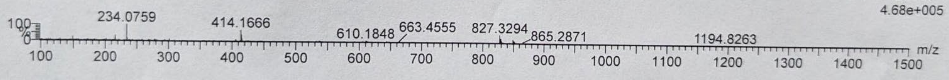

Minimum:				-50.0					
Maximum:		5.0	100.0	100.0					
Mass	Calc. Mass	mDa	PPM	DBE	i-FIT	Norm	Conf(%)	Formula
827.3294	827.3292	0.2	0.2	21.5	256.2	n/a	n/a	C43 H52 N8 O3 S2 Cl

Figure S94 – HRMS analysis of compound **6b**.

7a AMPRO-233

N-(2-(2-((S)-4-(4-chlorophenyl)-2,3,9-trimethyl-6H-thieno[3,2-f][1,2,4]triazolo[4,3-a][1,4]diazepin-6-yl)acetamido)ethyl)-4-((5-(4-(2-((2-(2,6-dioxopiperidin-3-yl)-1,3-dioxoisoindolin-4-yl)oxy)acetamido) piperidin-1-yl)-5-(tetrahydro-2H-pyran-4-yl)pent-3-yn-1-yl)thio)benzamide.

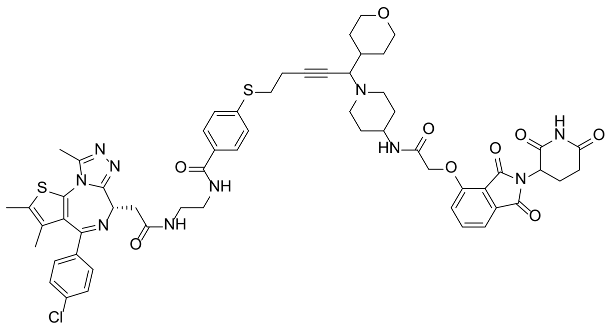

Chemical Formula: $C_{58}H_{61}ClN_{10}O_9S_2$
Exact Mass: 1140.3753
Molecular Weight: 1141.7495

TC E3 5031 (10 mg, 0.03 mmol, 1.0 eq) and HATU (17.1 mg, 0.05 mmol, 1.5 eq) were dissolved in a mixture of anhydrous DMF (1 mL) and DIPEA (16 mg, 21 µL, 0.12 mmol, 4.0 eq). Upon dissolution **6b** (25 mg, 0.03 mmol, 1.0 eq) was added and the reaction mixture was stirred at room temperature for 48 hours. The reaction mixture was monitored by LCMS and upon completion it was diluted with DCM (5 mL) and washed with water (5 mL) and brine (5 mL), dried over $MgSO_4$, and concentrated to residue. The residue was purified by column chromatography (SiO2, 4g, DCM: MeOH). **7a** is isolated as a white solid (15.8 mg, 0.014mmol 46%). ^{1}H NMR (600 MHz, DMSO-d_6) δ 11.11 (s, 1H), 8.46 (t, *J* = 5.5 Hz, 1H), 8.38 (t, *J* = 5.5 Hz, 1H), 7.84 (d, *J* = 8.0 Hz, 1H), 7.79 (dd, *J* = 8.5, 7.0 Hz, 1H), 7.75 (d, *J* = 8.5 Hz, 2H), 7.47 (d, *J* = 7.0 Hz, 1H), 7.42 (d, *J* = 8.5 Hz, 2H), 7.37 (dd, *J* = 8.5, 2.5 Hz, 3H), 7.35 (d, *J* = 8.5 Hz, 2H), 4.74 (s, 2H), 4.49 (dd, *J* = 8.0, 6.0 Hz, 1H), 3.84 – 3.77 (m, 2H), 3.62 – 3.55 (m, 1H), 3.34 (s, 1H), 3.31 (s, 2H), 3.28 – 3.19 (m, 4H), 3.16 (t, *J* = 7.0 Hz, 2H), 2.98 (d, *J* = 10.0 Hz, 1H), 2.88 (m, 1H), 2.62 (d, *J* = 10.0 Hz, 1H), 2.59 – 2.57 (m, 1H), 2.56 (s, 3H), 2.55 – 2.50 (m, 5H), 2.46 – 2.40 (m, 1H), 2.39 (s, 3H), 2.14 (t, *J* = 11.0 Hz, 1H), 2.02 (m, 1H), 1.79 – 1.72 (m, 4H), 1.69 (d, *J* = 13.0 Hz, 1H), 1.63 (d, *J* = 11.0 Hz, 1H), 1.56 (s, 3H), 1.44 (m, 1H), 1.35 (d, *J* = 10.0 Hz, 1H), 1.17 – 1.02 (m, 2H). ^{13}C NMR (151 MHz, DMSO-d_6) δ 170.5, 170.4, 167.2, 166.5, 166.2, 166.1, 163.5 (2C), 155.5, 155.4, 150.3, 140.6, 137.4, 137.2, 135.6, 133.4, 132.7, 131.7, 131.1, 130.6 (2C), 130.3, 130.0, 128.9 (2C), 128.3 (2C), 127.2 (2C), 120.9, 117.2, 116.5, 78.3, 67.3, 67.2, 62.4, 54.3 (2C), 49.2, 45.1, 40.5, 38.7, 38.1, 37.0 (2C), 32.4, 31.5 (2C), 31.4, 30.7, 22.4, 19.1 (2C), 14.5, 13.1, 11.8. HRMS $C_{58}H_{61}ClN_{10}O_9S_2$ Calculated [M+H] = 1141.3831. Experimental [M+H] = 1141.3461 (ppm = - 32.4). LCMS RT 5.129 min, A% = 90 %* single peak, Mw [M+H/2] = 571.65 – weak sample and full mass outside m/z range of LCMS.

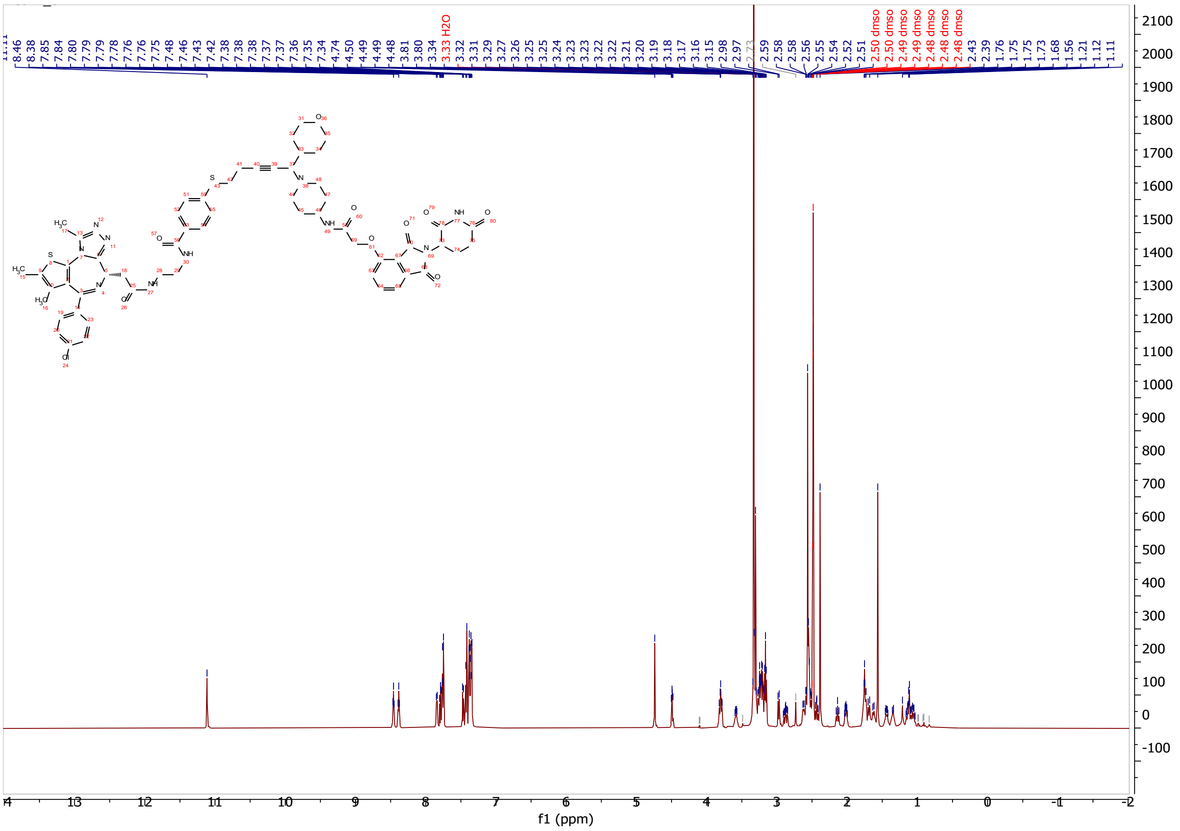

Figure S95 – ^{1}H spectra of compound **7a**.

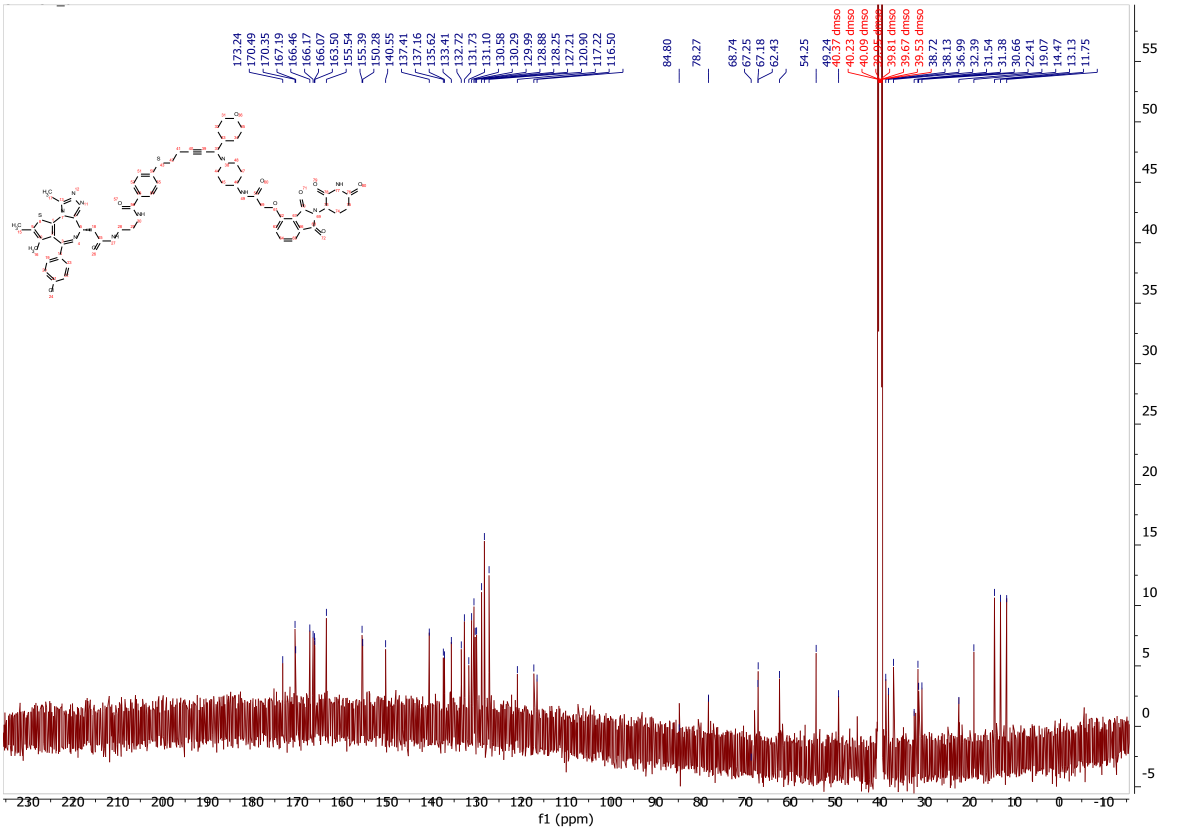

Figure S96 – ^{13}C spectra of compound **7a**.

Analytical LC-UV/MS Report

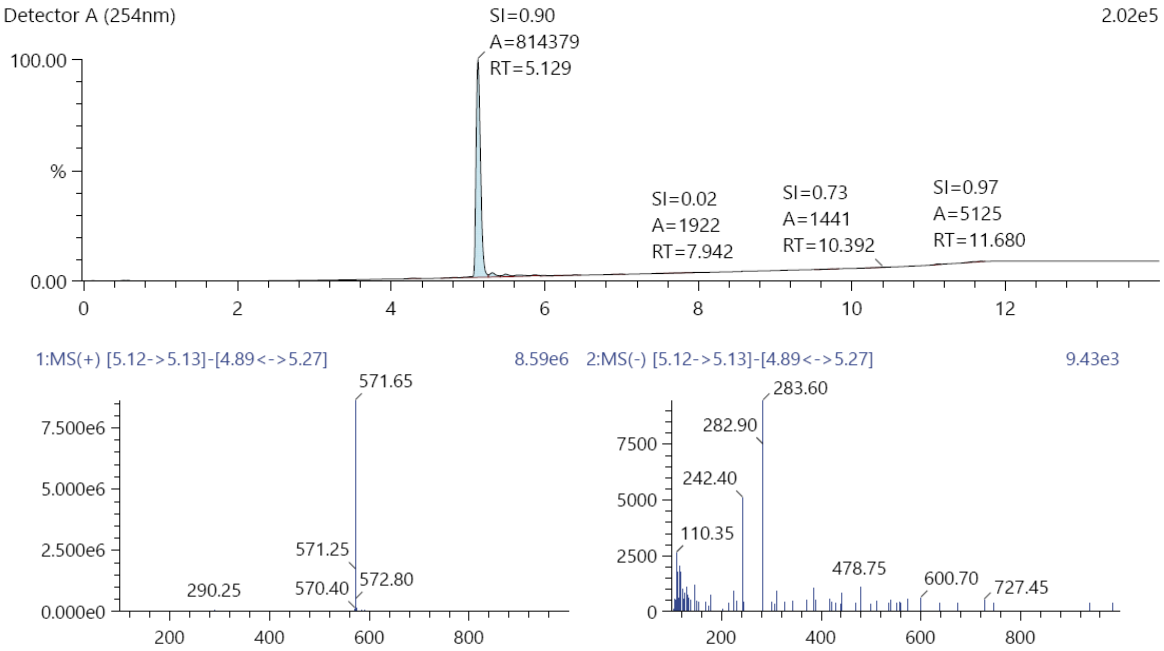

Figure S97 – LCMS analysis of compound **7a**.

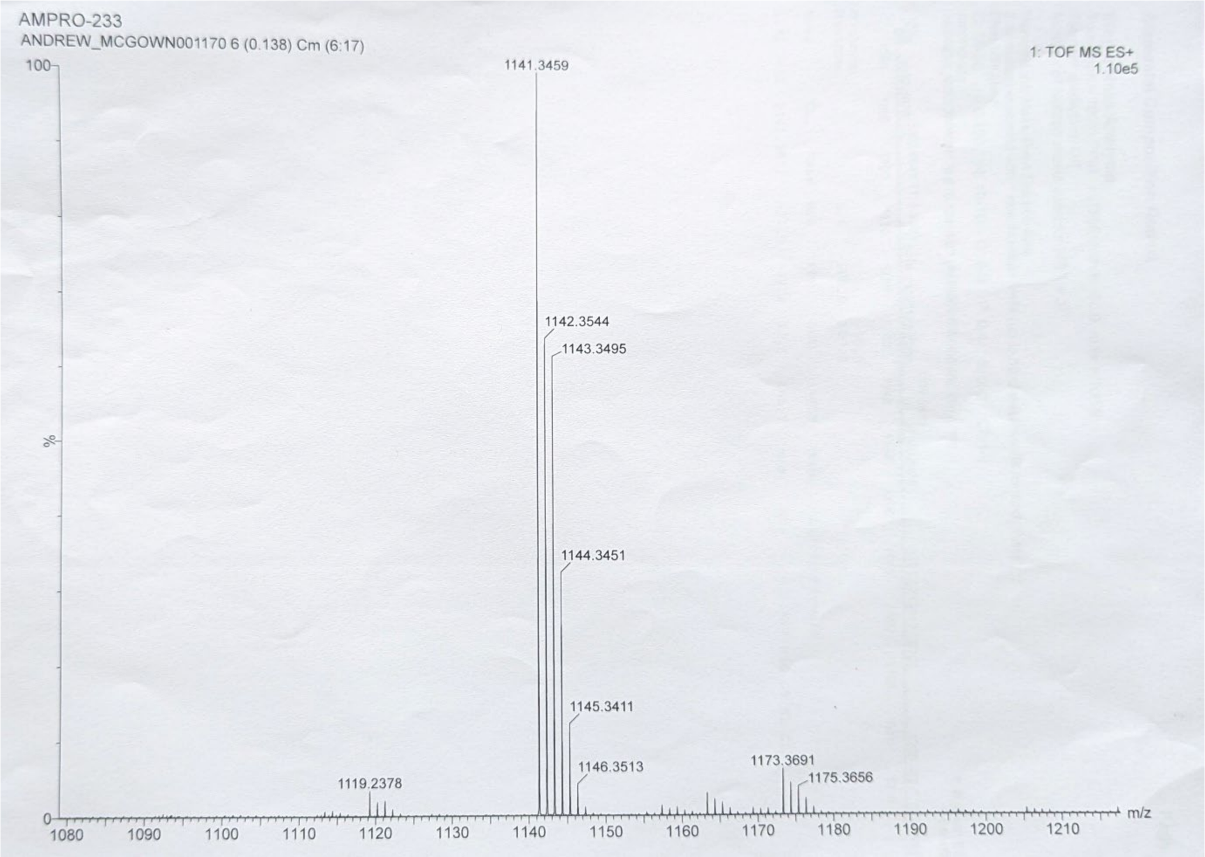

Elemental Composition Report

Page 1

Single Mass Analysis

Tolerance = 100.0 PPM / DBE: min = -50.0, max = 100.0
Element prediction: Off
Number of isotope peaks used for i-FIT = 3

Monoisotopic Mass, Even Electron Ions
2 formula(e) evaluated with 1 results within limits (up to 50 closest results for each mass)
Elements Used:
C: 58-58 H: 0-100 N: 10-10 O: 9-9 P: 0-1 S: 2-2 Cl: 1-1
AMPRO-233
ANDREW_MCGOWN001170 6 (0.138) AM2 (Ar,20000.0,0.00,0.00); Cm (6:17)

1: TOF MS ES+
8.64e+005

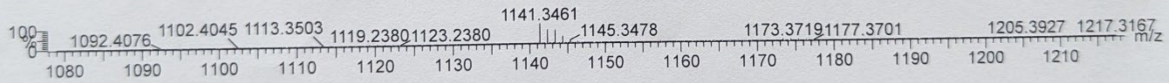

Minimum:				-50.0				
Maximum:		5.0	100.0	100.0				
Mass	Calc. Mass	mDa	PPM	DBE	i-FIT	Norm	Conf(%)	Formula
1141.3461	1141.3831	-37.0	-32.4	32.5	165.7	n/a	n/a	C58 H62 N10 O9 S2 Cl

Figure S98 – HRMS analysis of compound **7a.**

7b AMPRO-234

N1-(1-(5-((4-((2-(2-((S)-4-(4-chlorophenyl)-2,3,9-trimethyl-6H-thieno[3,2-f][1,2,4]triazolo[4,3-a][1,4]diazepin-6-yl)acetamido)ethyl)carbamoyl)phenyl)thio)-1-(tetrahydro-2H-pyran-4-yl)pent-2-yn-1-yl)piperidin-4-yl)-N6-((S)-1-((2S,4R)-4-hydroxy-2-((4-(4-methylthiazol-5-yl)benzyl)carbamoyl)pyrrolidin-1-yl)-3,3-dimethyl-1-oxobutan-2-yl)adipamide

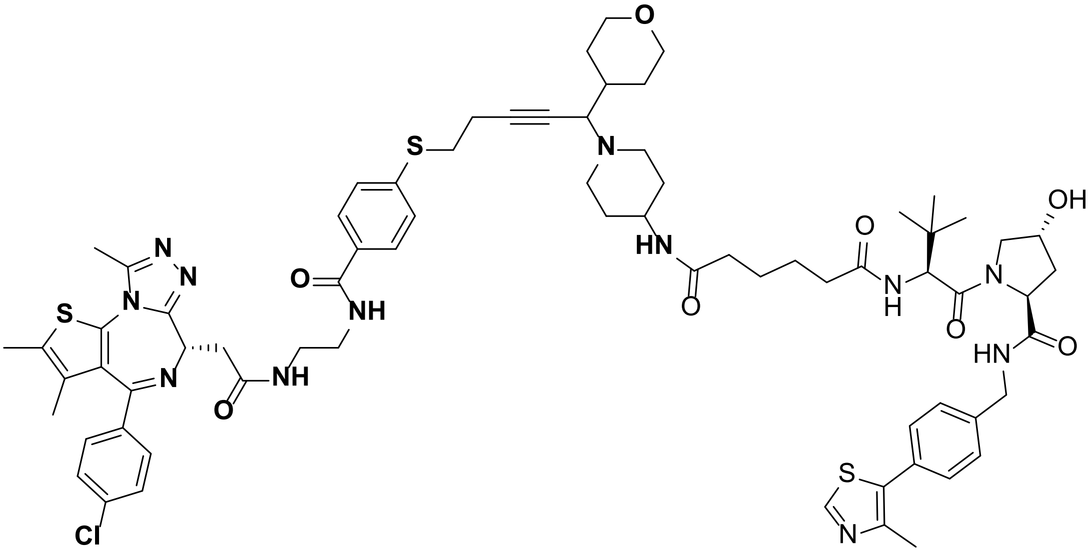

Chemical Formula: $C_{71}H_{87}ClN_{12}O_8S_3$
Exact Mass: 1366.5620
Elemental Analysis: C, 62.33; H, 6.41; Cl, 2.59; N, 12.29; O, 9.35; S, 7.03

7b was synthesised using the method outlined in **7a** on a 0.03mmol scale. **7b** was isolated as a white solid (11.7mg, 0.01mmol, 29%). ^{1}H NMR (600 MHz, DMSO-d_6) δ 8.97 (s, 1H), 8.61 (t, *J* = 6.0 Hz, 1H), 8.54 (t, *J* = 5.5 Hz, 1H), 8.45 (m, 2H), 8.27 (d, *J* = 8.5 Hz, 1H), 7.86 (d, *J* = 9.5 Hz, 1H), 7.78 (d, *J* = 8.0 Hz, 2H), 7.72 (d, *J* = 8.0 Hz, 1H), 7.45 – 7.36 (m, 4H), 7.35 (dd, *J* = 8.5, 2.5 Hz, 5H), 4.53 – 4.47 (m, 2H), 4.43 – 4.37 (m, 2H), 4.32 (s, 1H), 4.19 (dd, *J* = 16.0, 5.5 Hz, 1H), 3.79 (t, *J* = 13.0 Hz, 2H), 3.67 – 3.58 (m, 2H), 3.35 – 3.26 (m, 1H), 3.24 (m, 1H), 3.21 (d, *J* = 5.0 Hz, 1H), 3.16 (m, 4H), 2.95 (d, *J* = 10.0 Hz, 1H), 2.58 (d, *J* = 6.0 Hz, 1H), 2.55 (d, *J* = 8.5 Hz, 5H), 2.42 (s, 3H), 2.38 (s, 3H), 2.38 (d, *J* = 13.5 Hz, 1H), 2.25 (dd, *J* = 14.5, 7.5 Hz, 1H), 2.08 (t, *J* = 12.0 Hz, 2H), 2.07 (s, 1H), 2.00 (t, *J* = 6.0 Hz, 3H), 1.87 (m, 1H), 1.77 – 1.70 (m, 1H), 1.67 (d, *J* = 13.0 Hz, 4H), 1.63 – 1.58 (m, 1H), 1.56 (s, 2H), 1.42 (t, *J* = 7.5 Hz, 5H), 1.40 – 1.31 (m, 1H), 1.30 – 1.18 (m, 3H), 1.11 (m, 1H), 1.04 (m, 1H), 0.90 (s, 9H). ^{13}C NMR (151 MHz, DMSO-d_6) δ 172.4, 171.7, 170.5, 170.1, 166.2, 163.5, 155.6, 151.9, 150.3, 148.1, 140.5, 140.0, 137.2, 135.6, 132.7, 131.7, 131.6, 131.1, 130.6, 130.3, 130.1, 130.0, 129.1 (3C), 128.9, 128.3 (2C), 128.1, 127.9 (2C), 127.2, 119.8, 84.7, 78.3, 69.3 (2C), 67.2, 62.4, 59.1 (2C), 56.7, 54.2, 42.1, 38.8, 38.4, 38.1, 37.0, 35.7 (2C), 35.2, 32.5, 31.6, 31.3, 30.7, 26.8 (3C), 25.6 (2C), 19.1, 16.4, 14.5, 13.1, 11.8. Some carbon signals hidden under DMSO-d_6 peak. HRMS $C_{71}H_{87}ClN_{12}O_8S_3$ Calculated [M+H] = 1367.5699, [M+Na] = 1389.5518. Experimental [M+H] = 1367.5762, [M+Na] = 1389.5559 (ppm = - 3.4). LCMS RT 0.656 min, A% = 98 %, Mw [M+H/2] = 684.85 - full mass outside m/z range of LCMS.

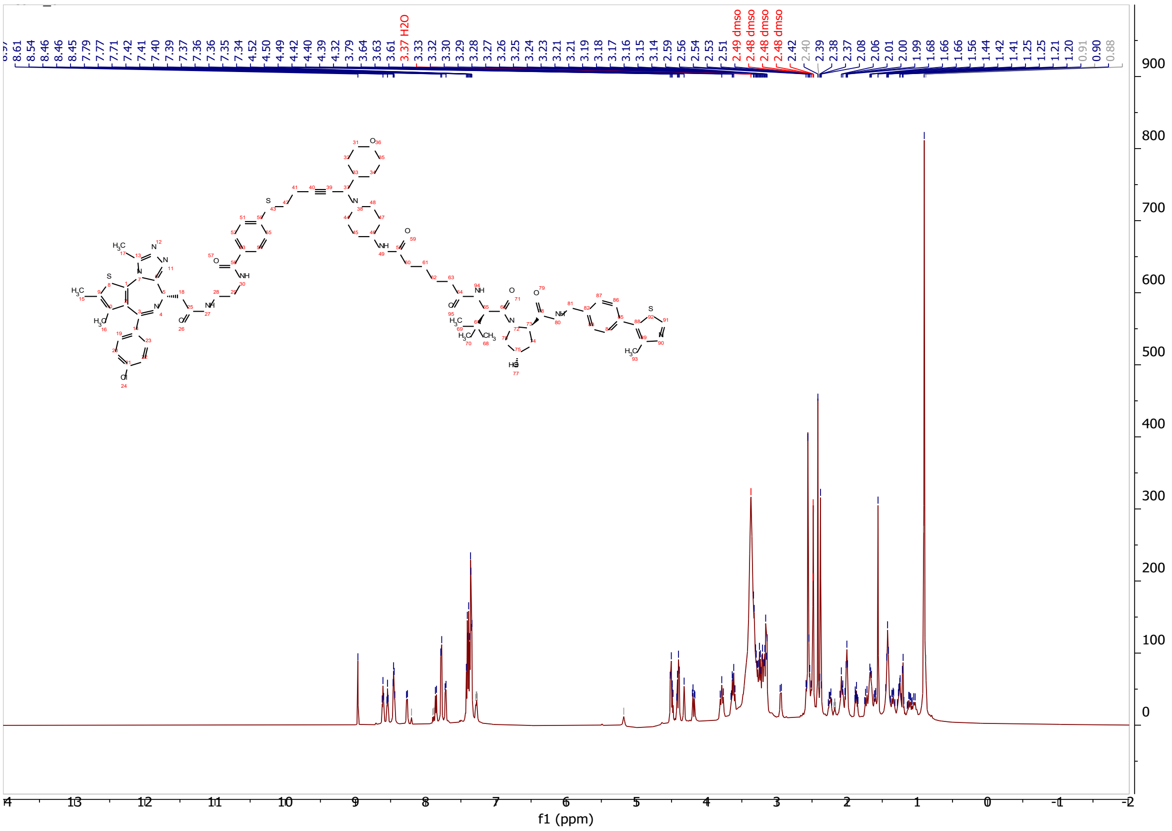

Figure S99 – ^{1}H spectra of compound **7b.**

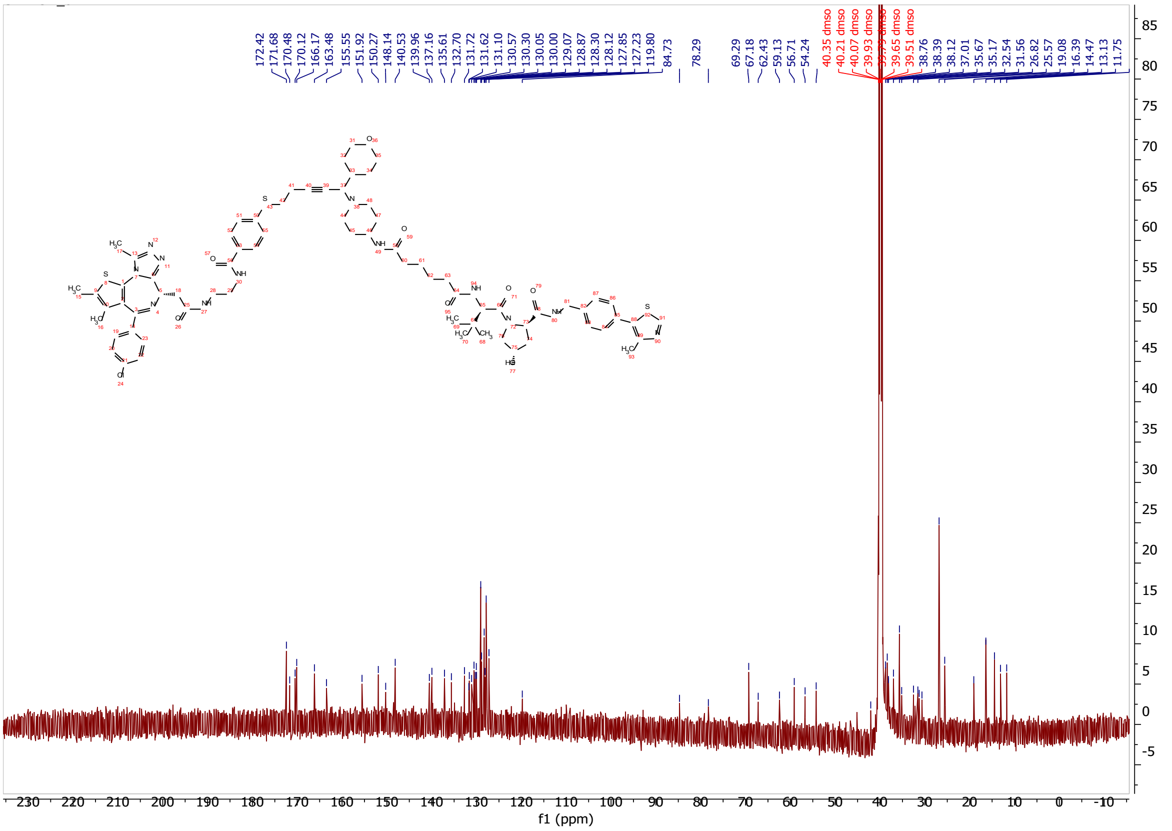

Figure S100 – ^{13}C spectra of compound **7b.**

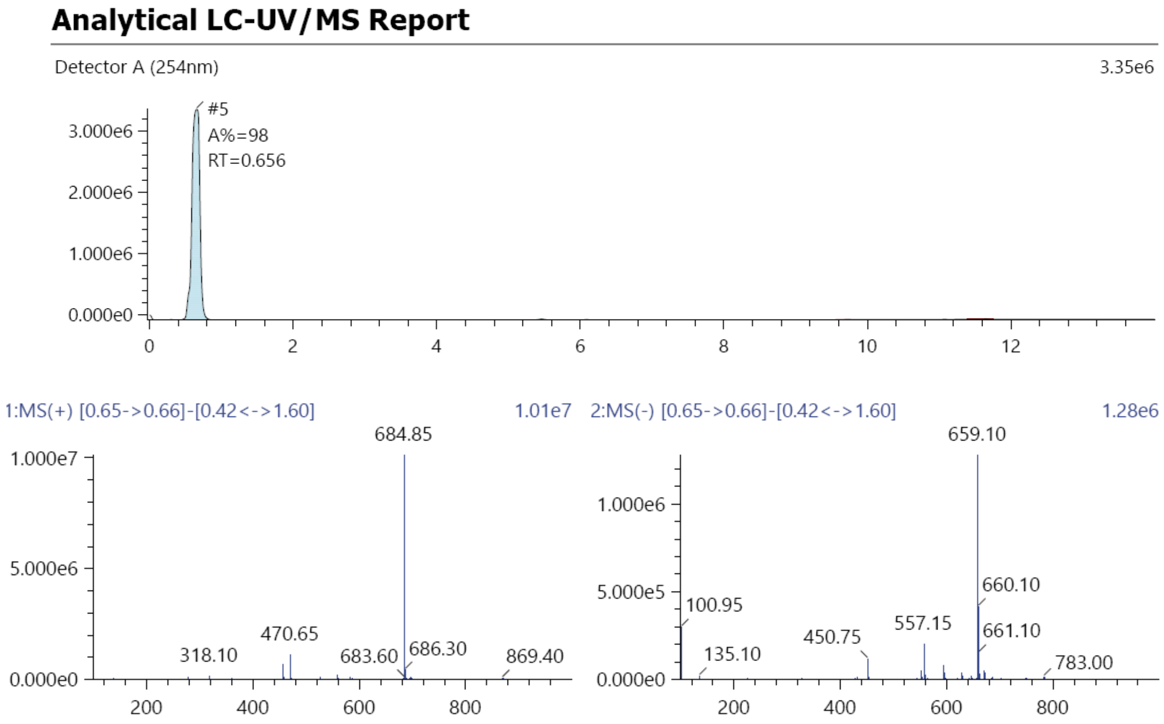

Figure S101 – LCMS analysis of compound **7b.**

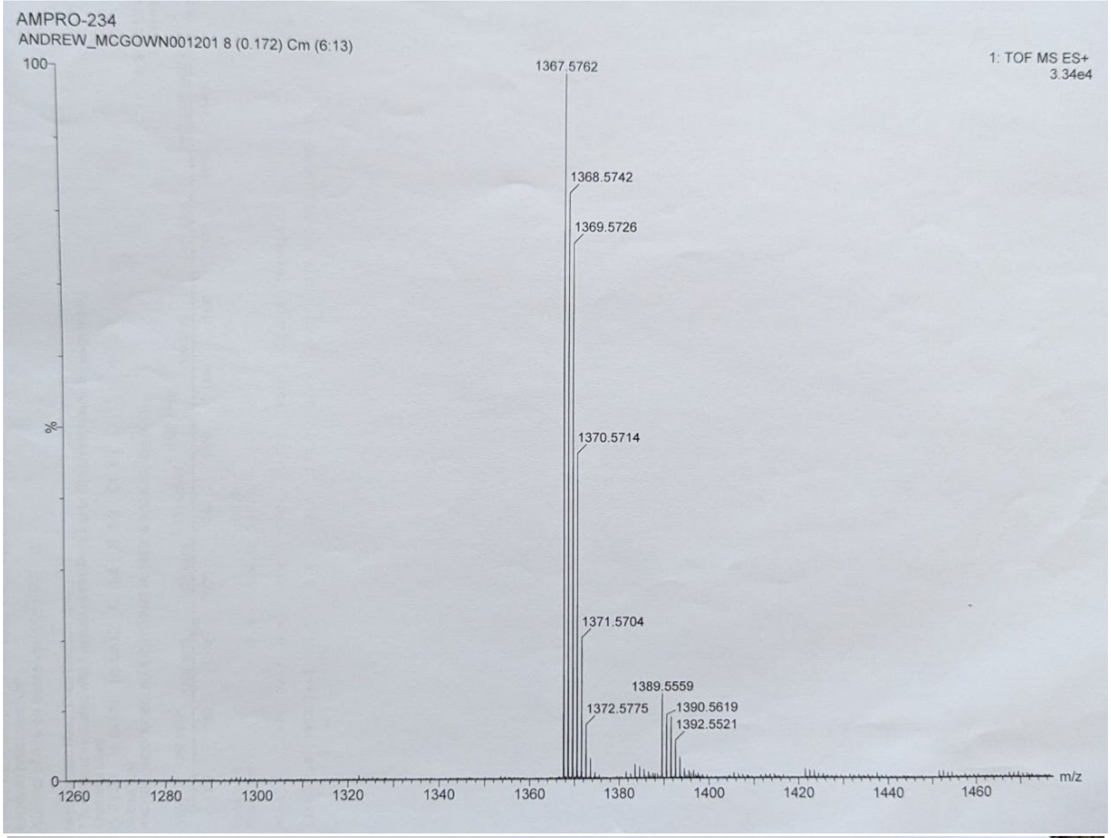

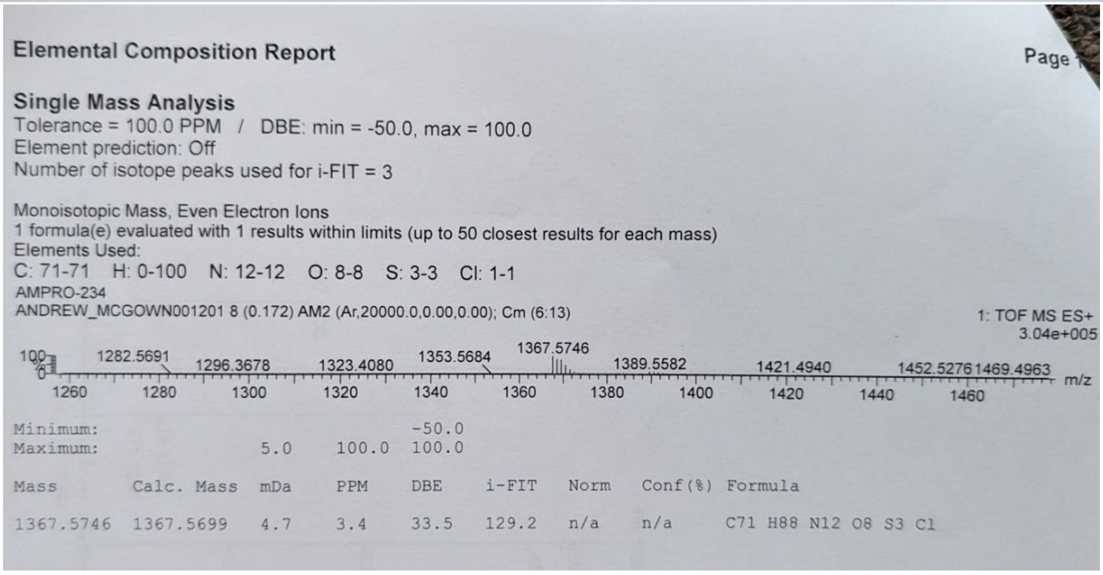

Elemental Composition Report

Page 1

Single Mass Analysis
Tolerance = 100.0 PPM / DBE: min = -50.0, max = 100.0
Element prediction: Off
Number of isotope peaks used for i-FIT = 3

Monoisotopic Mass, Even Electron Ions
1 formula(e) evaluated with 1 results within limits (up to 50 closest results for each mass)
Elements Used:
C: 71-71 H: 0-100 N: 12-12 O: 8-8 S: 3-3 Cl: 1-1
AMPRO-234
ANDREW_MCGOWN001201 8 (0.172) AM2 (Ar,20000.0,0.00,0.00); Cm (6:13)

1: TOF MS ES+
3.04e+005

Minimum:				-50.0				
Maximum:		5.0	100.0	100.0				
Mass	Calc. Mass	mDa	PPM	DBE	i-FIT	Norm	Conf(%)	Formula
1367.5746	1367.5699	4.7	3.4	33.5	129.2	n/a	n/a	C71 H88 N12 O8 S3 Cl

Figure S102 – HRMS analysis of compound **7b.**

7c N2261-048

2-((S)-4-(4-chlorophenyl)-2,3,9-trimethyl-6H-thieno[3,2-f][1,2,4]triazolo[4,3-a][1,4]diazepin-6-yl)-N-(4-(4-(2-((2-(2,6-dioxopiperidin-3-yl)-1,3-dioxoisoindolin-4-yl)oxy)acetyl)piperazin-1-yl)-4-(1-(2-((2-(2,6-dioxopiperidin-3-yl)-1,3-dioxoisoindolin-4-yl)oxy)acetyl)piperidin-4-yl)but-2-yn-1-yl)acetamide.

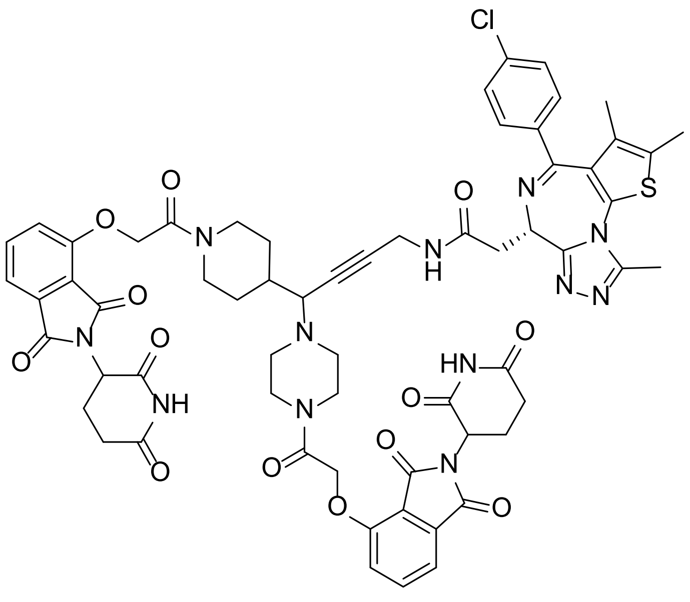

Chemical Formula: $C_{62}H_{59}ClN_{12}O_{13}S$
Exact Mass: 1246.3734
Molecular Weight: 1247.7225

At 0°C propylphosphonic anhydride (50 wt% in EtOAc, 114.5mg, 214µL, 0.18mmol, 3.0 eq) and DIPEA (38.8mg, 52µL, 0.3mmol, 5.0 eq) were added to a solution of 2-((2-(2,6-dioxopiperidin-3-yl)-1,3-dioxoisoindolin-4-yl)oxy)acetic acid (42.3mg, 0.13mmol, 2.1 eq) and **2k** (37.2mg, 0.06mmol, 1.0 eq) in 1,2-dichloroethane (600µL). The reaction mixture was stirred at 50°C for 3 hours and then at room temperature for 12 hours.

Upon completion the reaction was diluted with water (5mL) and a mixture of DCM and MeOH (9:1, 15mL). Using aq HCl (2N) the mixture was acidified to pH 2, the layers were separated and the aqueous was re-extracted with the mixture of DCM and MeOH (9:1, 2 x 5mL). The combined organics were passed through a phase separator and concentrated to dryness.

The material was purified by column chromatography (4g, SiO_2, DCM: MeOH) to yield a white solid of ~80% purity by LCMS. This was dissolved in a mixture of MeOH: DMSO (8:2, 1mL) and purified by preparative HPLC (C18, 20 x 150mm, 5µM) using a gradient from 15 to 80 % MeCN in water (modified by 0.1% formic acid). **7c** (mixt. of diast.) was isolated as a white solid (10 mg, 0.01mmol, 13% yield). ^{1}H NMR (600 MHz, DMSO-d_6) δ 11.12 (d, *J*= 3.0 Hz, 2H), 8.70 – 8.62 (m, 1H), 7.81 – 7.72 (m, 2H), 7.48 (dd, *J*= 8.5, 3.5 Hz, 2H), 7.46 – 7.36 (m, 4H), 7.32 – 7.25 (m, 2H), 5.26 – 5.14 (m, 4H), 5.14 – 5.07 (m, 2H), 4.56 – 4.49 (m, 1H), 4.34 – 4.25 (m, 1H), 4.11 – 4.00 (m, 1H), 3.98 – 3.89 (m, 1H), 3.87 – 3.75 (m, 1H), 3.56 – 3.40 (m, 4H), 3.30 – 3.22 (m, 1H), 3.18 – 3.12 (m, 1H), 3.09 – 2.98 (m, 1H), 2.94 – 2.83 (m, 2H), 2.68 – 2.59 (m, 3H), 2.58 (s, 3H), 2.52 (s, 4H), 2.47 – 2.40 (m, 3H), 2.38 (s, 3H), 2.07 – 2.00 (m, 2H), 1.99 – 1.89 (m, 2H), 1.86 – 1.76 (m, 1H), 1.60 (s, 3H), 1.32 – 1.16 (m, 1H), 1.09 – 0.96 (m, 1H). Insufficient material to record a useable ^{13}C NMR. HRMS $C_{62}H_{59}ClN_{12}O_{13}S$ Calculated [M+H] = 1247.3812. Experimental [M+H] = 1247.3793 (ppm = - 1.5). RT 5.635 min, A% = 92 %*, Mw [M+H/2] = 624.70 - *single peak - full mass outside m/z range of LCMS.

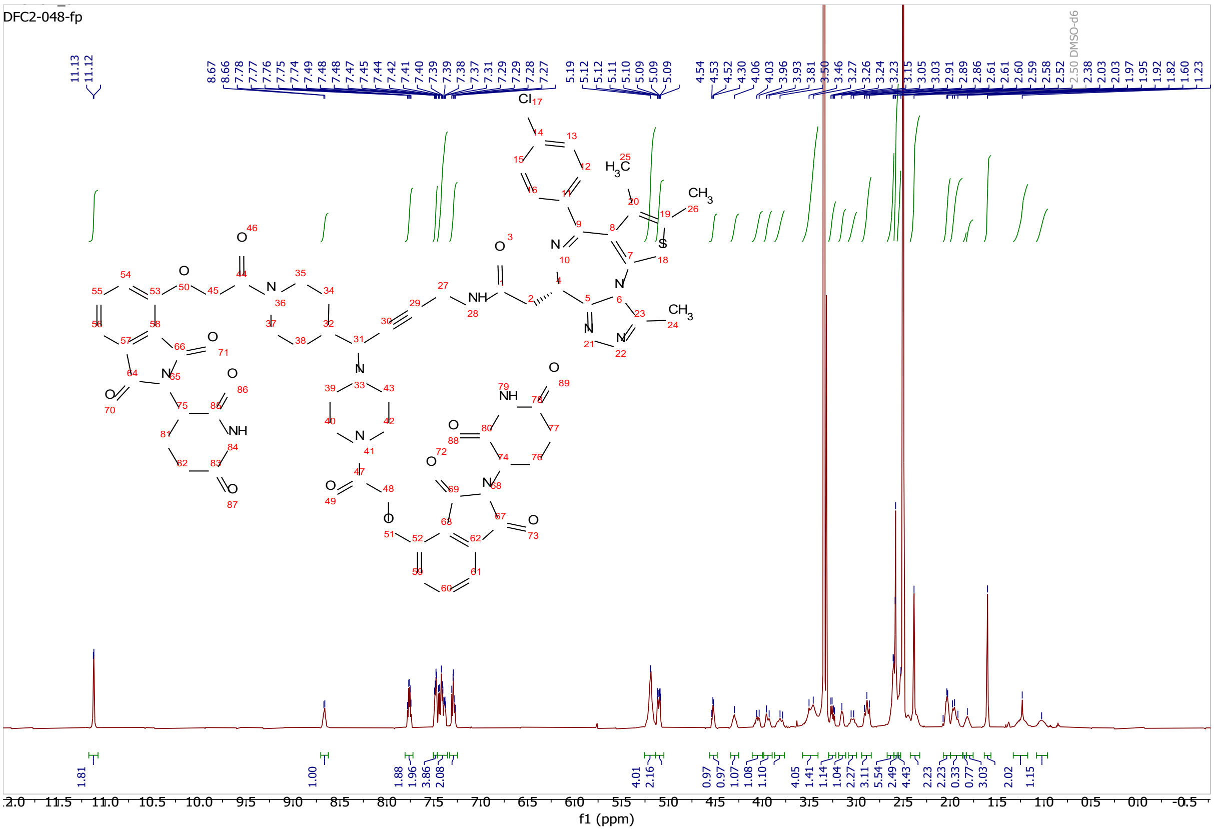

Figure S103 – ^{1}H spectra of compound **7c**.

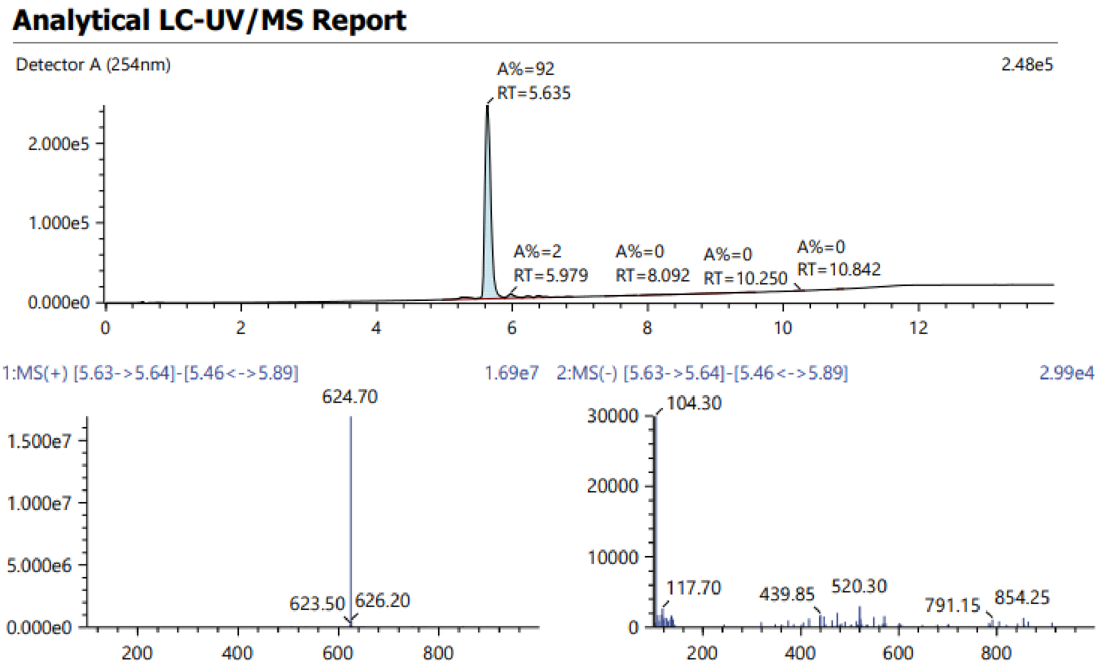

Figure S104 – LCMS analysis of compound **7c**.

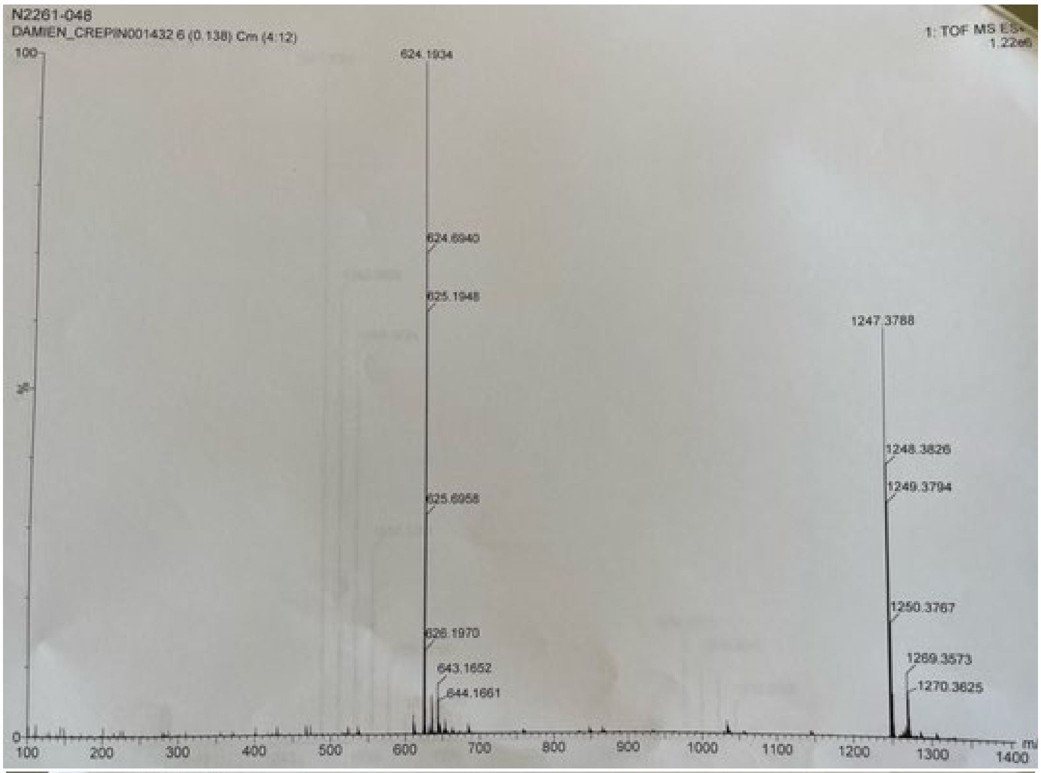

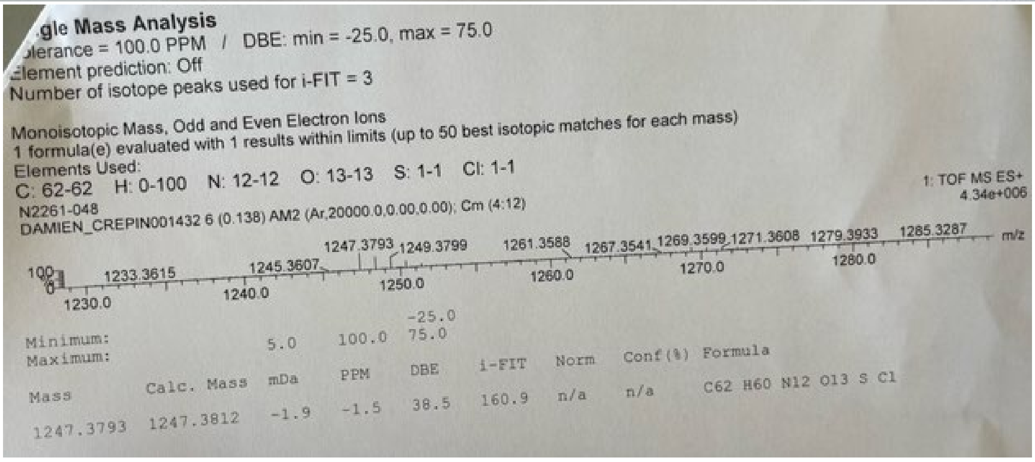
[illegible]gle Mass Analysis
[illegible]lerance = 100.0 PPM / DBE: min = -25.0, max = 75.0
[illegible]lement prediction: Off
Number of isotope peaks used for i-FIT = 3

Monoisotopic Mass, Odd and Even Electron Ions
1 formula(e) evaluated with 1 results within limits (up to 50 best isotopic matches for each mass)
Elements Used:
C: 62-62 H: 0-100 N: 12-12 O: 13-13 S: 1-1 Cl: 1-1

N2261-048
DAMIEN_CREPIN001432 6 (0.138) AM2 (Ar,20000.0,0.00,0.00); Cm (4:12)
1: TOF MS ES+
4.34e+006

Mass	Calc. Mass	mDa	PPM	DBE	i-FIT	Norm	Conf(%)	Formula
Minimum:				-25.0				
Maximum:		5.0	100.0	75.0				
1247.3793	1247.3812	-1.9	-1.5	38.5	160.9	n/a	n/a	C62 H60 N12 O13 S Cl

Figure S105 – HRMS analysis of compound **7c**.

1. Mhlongo, N.Z., et al., *Microwave-assisted synthesis of meso-carboxyalkyl-BODIPYs and an application to fluorescence imaging.* Org Biomol Chem, 2020. **18**(39): p. 7876-7883.
